# Global patterns of antibiotic resistance in group B *Streptococcus*: a systematic review and meta-analysis

**DOI:** 10.3389/fmicb.2025.1541524

**Published:** 2025-04-16

**Authors:** Chou-Yi Hsu, Safoura Moradkasani, Muath Suliman, Subasini Uthirapathy, Ahmed Hussein Zwamel, Ahmed Hjazi, Raghav Vashishth, Masoumeh Beig

**Affiliations:** ^1^Thunderbird School of Global Management, Arizona State University Tempe Campus, Phoenix, AZ, United States; ^2^Department of Bacteriology, Pasteur Institute of Iran, Tehran, Iran; ^3^Department of Laboratory Medicine, School of Medical Sciences, King Khalid University, Abha, Saudi Arabia; ^4^Pharmacy Department Tishk International University Erbil, Kurdistan Region, Iraq; ^5^Medical Laboratory Technique College, The Islamic University, Najaf, Iraq; ^6^Medical Laboratory Technique College, The Islamic University of Al Diwaniyah, Al Diwaniyah, Iraq; ^7^Medical Laboratory Technique College, The Islamic University of Babylon, Babylon, Iraq; ^8^Department of Medical Laboratory, College of Applied Medical Sciences, Prince Sattam bin Abdulaziz University, Al-Kharj, Saudi Arabia; ^9^Department of Surgery, National Institute of Medical Sciences, NIMS University Rajasthan, Jaipur, India

**Keywords:** antibiotic resistance, group B *Streptococcus*, meta-analysis, antibiotic susceptibility, Joanna Briggs Institute tool

## Abstract

**Objectives:**

*Streptococcus agalactiae*, or group B *Streptococcus* (GBS), is a significant pathogen associated with severe infections in neonates, particularly sepsis and meningitis. The increasing prevalence of antibiotic resistance among GBS strains is a growing public health concern, necessitating a comprehensive meta-analysis to evaluate the prevalence of this resistance globally.

**Methods:**

We conducted a comprehensive systematic search across four major scientific databases: Scopus, PubMed, Web of Science, and EMBASE, targeting articles published until December 13, 2023. This meta-analysis focused on studies that examined antibiotic resistance in GBS strains. The Joanna Briggs Institute tool was employed to assess the quality of the included studies. This meta-analysis applied a random-effects model to synthesize data on antibiotic resistance in GBS, incorporating subgroup analyses and regression techniques to explore heterogeneity and trends in resistance rates over time. Outliers and influential studies were identified using statistical methods such as Cook’s distance, and funnel plot asymmetry was assessed to evaluate potential publication bias. All analyses were conducted using R software (version 4.2.1) and the metafor package (version 3.8.1).

**Results:**

This study included 266 studies from 57 countries, revealing significant variability in GBS antibiotic resistance rates. The highest resistance rates were observed for tetracycline (80.1, 95% CI: 77.1–82.8%), while tedizolid (0.1, 95% CI: 0.0–0.8%) showed the lowest resistance rates. Significant heterogeneity in resistance rates was observed, particularly for antibiotics such as azithromycin and gentamicin (*I*^2^ = 97.29%), variability across studies. On the other hand, tigecycline and ceftaroline exhibited no heterogeneity (*I*^2^ = 0%), suggesting consistent resistance patterns. Subgroup analyses revealed disparities in resistance rates based on country, continent, and methodological categories. Significant increase in resistance rates for several antibiotics over time, including clindamycin, erythromycin, ceftriaxone, cefuroxime, ciprofloxacin, levofloxacin, moxifloxacin, chloramphenicol, and ofloxacin. Ofloxacin and cefuroxime showed particularly steep trends. Conversely, a declining resistance trend was observed for oxacillin.

**Conclusion:**

This study emphasizes the growing issue of antibiotic resistance in GBS strains. Notable resistance to older and newer antibiotics, increasing resistance over time, regional disparities, and methodological variations are noted. Rising resistance trends for multiple antibiotics underscore the urgent need for global surveillance and improved antibiotic stewardship.

**Systematic Review Registration:**

https://www.crd.york.ac.uk/PROSPERO/view/CRD42024566269, CRD42024566269.

## 1 Introduction

The increasing prevalence of antibiotic resistance has emerged as a primary global public health concern, significantly complicating the management of infectious diseases across diverse populations (Murray et al., 2022; Heath and Jardine, 2014; Imperi et al., 2024; Aijaz et al., 2023). Among the myriad pathogens contributing to this crisis, group B *Streptococcus* (GBS) stands out due to its substantial impact on vulnerable groups, including newborns, pregnant women, and the elderly. GBS is a leading cause of severe infections in neonates, often resulting in life-threatening conditions such as sepsis, pneumonia, and meningitis (Alshamlan and Anumakonda, 2024). The timely and effective administration of antibiotics is crucial for mitigating these risks; however, the emergence of resistance to commonly prescribed antibiotics poses a formidable challenge to healthcare systems worldwide (Muteeb et al., 2023).

The treatment of GBS infections has evolved significantly with the introduction of antibiotics, shaping both clinical outcomes and bacterial resistance patterns (Sabroske et al., 2023). The first antibiotic used against GBS was penicillin, introduced in the 1940s, which revolutionized bacterial infection treatment due to its broad-spectrum activity and sustained efficacy (Aminov, 2010). Despite its continued effectiveness, alternative antibiotics such as erythromycin and clindamycin were introduced in the 1960s and 1970s to provide treatment options for penicillin-allergic patients (Solensky et al., 2000). Over time, additional drugs, including cephalosporins, vancomycin, and linezolid, were developed to combat GBS infections, particularly in cases of emerging multidrug-resistant (MDR) (Li et al., 2020). However, these antibiotics’ widespread and prolonged use has led to the gradual emergence of resistance. While GBS has largely remained susceptible to penicillin, resistance has been increasingly reported against macrolides, lincosamides, and fluoroquinolones (Oliveira et al., 2022). Notably, the growing resistance to erythromycin and clindamycin, first reported in the late 20th century, coincided with their expanded clinical use (Sabroske et al., 2023). The resistance profile of GBS has continued to evolve, highlighting a clear correlation between antibiotic pressure and bacterial adaptation (Sabroske et al., 2023). This trend highlights the need for ongoing resistance monitoring and historical analysis of antibiotic use in GBS to address treatment challenges (Laitin et al., 2024; Muteeb et al., 2023; Hooshiar et al., 2024).

Previous studies have provided valuable insights into GBS antibiotic resistance (Sabroske et al., 2023; Verma et al., 2023); however, gaps still need to be found in understanding its global epidemiology and trends. Studies on antibiotic resistance in GBS have often been limited to specific regions or antibiotics, resulting in fragmented data that fails to capture the global scope of the issue. However, recent research has uncovered a concerning pattern: GBS is increasingly resistant to widely used antibiotics like erythromycin and clindamycin. This trend has been observed across diverse regions, including Europe, North America, and South America, highlighting the growing challenge of managing GBS infections effectively (Al-Subol et al., 2022; Jones et al., 2022). Moreover, earlier meta-analyses often require more granularity to explore potential variations in resistance rates across different geographic regions and periods, which is crucial for understanding the dynamic nature of antibiotic resistance. The mechanisms underlying antibiotic resistance in GBS involve genetic mutations, horizontal gene transfer, and the acquisition of resistance genes from other bacterial species (Liu and Liu, 2022; Arnold et al., 2022). This has led to a rise in strains resistant to traditional therapies, complicating treatment protocols and threatening patient outcomes (Terreni et al., 2021). Reliance on empirical antibiotic therapy, often based on historical susceptibility patterns, may need to be revised in the face of evolving resistance profiles (Merker et al., 2020). Consequently, healthcare providers must effectively adapt their strategies to manage GBS infections (Verma et al., 2023) and use these gaps by conducting a systematic review and meta-analysis to synthesize data from diverse global sources. This study aimed to document the current resistance landscape by analyzing relevant published literature to address the lack of statistical evaluations on antibiotic resistance in GBS. Additionally, we conducted subgroup analyses based on continents, countries, antimicrobial susceptibility testing (AST) categories, bacterial diagnostic methods, and year groups to identify factors influencing resistance variations.

## 2 Methods

This investigation, implemented following PRISMA guidelines, integrated a meta-analysis to strengthen the outcomes. It was registered in the PROSPERO registry with the assigned code CRD42024566269.

### 2.1 Eligibility criteria

The eligibility criteria for incorporating articles into the meta-analysis were studies that investigated GBS, reported the proportion of resistance, determined the sample size, and published full-text articles in English. The exclusion criteria were languages other than English, case reports, single-arm studies, cohort studies, and pharmacokinetic studies.

### 2.2 Information sources

We extensively searched several major online databases, including Scopus, PubMed, Web of Science, and EMBASE, focusing on studies published up to December 2023. These databases were chosen for their extensive and comprehensive coverage of the biomedical literature, ensuring a broad scope for our systematic review.

### 2.3 Search strategy

The search syntax used for PubMed and other databases was as follows: ((“*Streptococcus agalactiae* OR group B strep* OR GBS OR *S. agalactiae*)).

The search syntax was adjusted according to each database’s guidelines (see the Supplementary material for detailed search syntax used for each database). This meticulous methodological approach aimed to cover all the necessary research topics.

### 2.4 Selection process

The systematic online database search results were imported into EndNote (version 20), and duplicates were removed. Two authors (SK and MB) independently searched and analyzed relevant publications to prevent bias. A third author (MH) investigated these disparities.

### 2.5 Data collection process

The extracted data included the first author(s), publication year, country, diagnostic method, sample source, number of positive tests, and the total number of individuals (sample size). To avoid errors in data extraction, the two authors independently extracted the necessary data and agreed on discrepant data.

### 2.6 Quality assessment and subgroup analysis

The Joanna Briggs Institute (JBI) tool was used to evaluate the quality of the included articles. Two authors (MB and SK) independently assessed their quality, and a third author (MH) investigated these disparities.

To assess the quality of the included studies, we performed a comprehensive risk of bias assessment based on key methodological criteria. These criteria included the clarity of sample inclusion, detailed description of study subjects and settings, standard and objective criteria for measuring the condition, identification, and management of confounding factors, validity and reliability of outcome measurements, and the appropriateness of statistical analyses.

In addition, to validate the robustness of our findings, we conducted subgroup analyses based on study quality. The studies were categorized into three quality groups: low risk (L), some concerns (S), and high risk (H).

This approach allowed us to assess the potential influence of study quality on the overall results and ensure that our conclusions were not unduly affected by studies with a higher risk of bias.

### 2.7 Effect measures

This meta-analysis investigated the prevalence of antibiotic resistance by analyzing the proportion of resistant isolates across various research studies. Subgroup analyses and meta-regression were employed to understand the factors contributing to the differences in resistance rates, considering variables such as country of origin. In addition, this study explored changing trends in antibiotic resistance over time.

### 2.8 Synthesis methods

The analysis was performed using proportions as outcome measures. This study’s primary objective was to determine the prevalence of antibiotic resistance in bacterial strains. Its secondary goal was to identify the sources of heterogeneity between the groups through subgroup analysis and regression based on country. Additionally, we investigated the trends in antibiotic resistance rates across the years.

### 2.9 Statistics

A random effects model was used to fit the data. The amount of heterogeneity (i.e., *τ*^2^) was estimated using the DerSimonian–Laird estimator. In addition to the estimate of *τ*^2^, the *Q*-test for heterogeneity and *I*^2^ statistic were reported. Any heterogeneity was detected (i.e., *τ*^2^ > 0, regardless of the results of the *Q*-test). Meta-regression analysis was conducted using moderator analysis to investigate the trends in antibiotic resistance rates over time. Studentized residuals and Cook’s distance were used to examine whether the studies were outliers or influential in the model context. Studies with a studentized residual larger than the 100 × (1–0.05/(2 × *k*))th percentile of a standard normal distribution were considered potential outliers (i.e., using a Bonferroni correction with two-sided *α* = 0.05, for *k* studies included in the meta-analysis). Studies with Cook’s distance more extensive than the median plus six times the interquartile range of Cook’s distances were considered influential. Rank correlation and regression tests using the standard error of the observed outcomes as predictors were used to check for funnel plot asymmetry. The analysis used R (version 4.2.1) and the metafor package (version 3.8.1) (DerSimonian and Laird, 1986; Cochran, 1954; Higgins and Thompson, 2002; Viechtbauer and Cheung, 2010; Begg and Mazumdar, 1994; Sterne and Egger, 2005; Viechtbauer, 2010; Kuhn et al., 2020).

### 2.10 Reporting bias assessment and certainty assessment

We used rank correlation and Egger’s regression tests to evaluate funnel plot asymmetry and to detect potential reporting bias. To enhance the reliability of our findings, we also applied Fail-Safe N and Trim-and-Fill methods, ensuring that our conclusions remain robust and credible despite publication biases.

## 3 Results

### 3.1 Study selection

**Figure 1:**
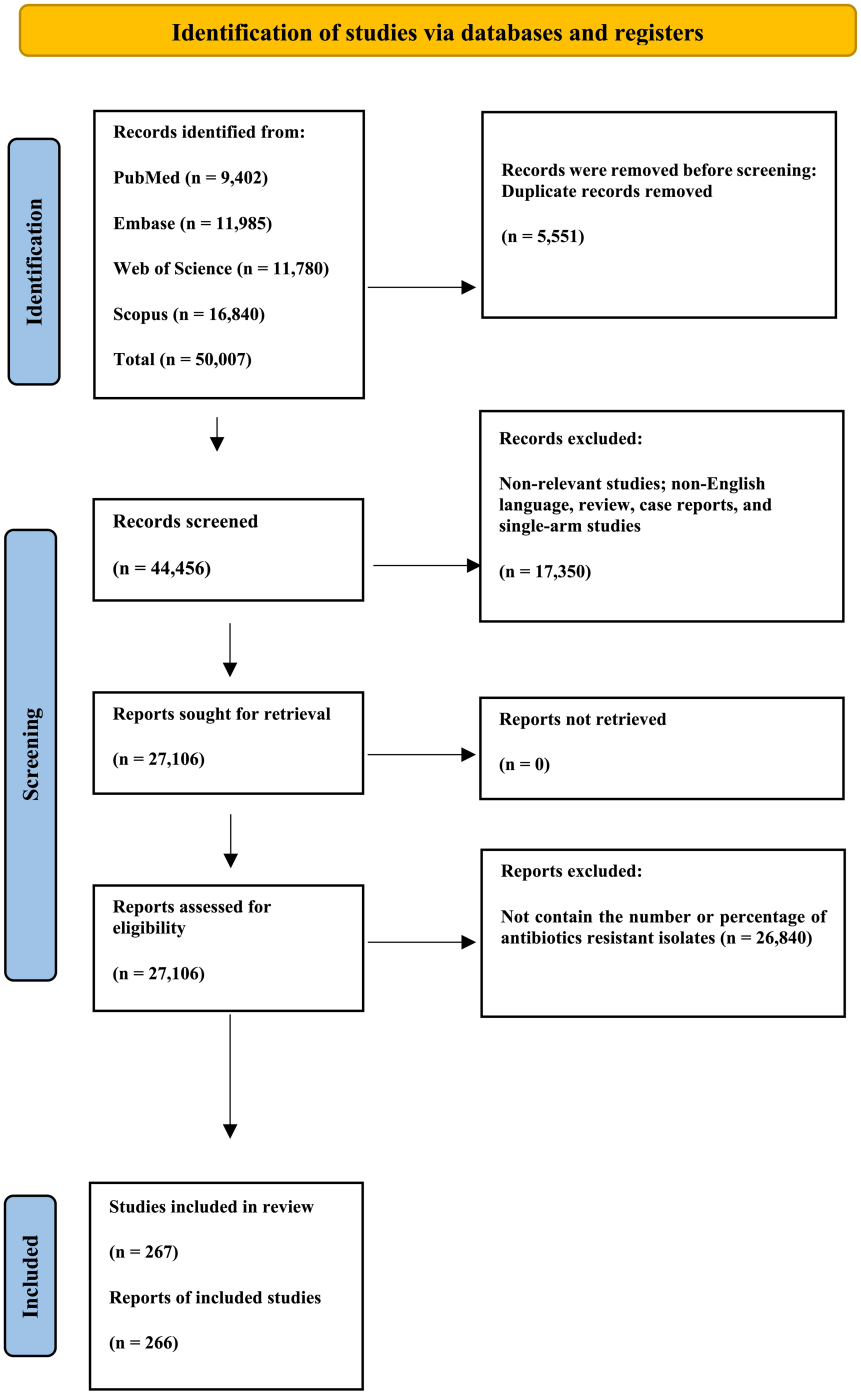
PRISMA flow chart of the article selection procedure. This PRISMA flow diagram illustrates identifying and selecting relevant studies for inclusion in the review. Studies were identified through a comprehensive search of PubMed, EMBASE, Web of Science, and Scopus databases. After removing duplicates and excluding irrelevant studies, 267 were included in the final review.

The present investigation involved compiling 50,007 studies from four prominent online databases: Scopus, PubMed, EMBASE, and Web of Science. Subsequently, 5,551 duplicate studies were excluded from the dataset. Additionally, 7,560 studies of non-relevant study types were systematically removed to ensure precision and relevance in the analytical framework. Furthermore, an exhaustive review excluded studies explicitly related to animal subjects. Ultimately, the assessment focused on the removed titles and abstracts of 9,790 animal studies. The 27,106 remaining studies were conducted to refine the dataset and ensure its appropriateness for subsequent analysis. Subsequently, 26,840 studies that did not include the number or percentage of antibiotic-resistant isolates were excluded from the meta-analysis. The present systematic review and meta-analysis included 266 eligible studies (Abdallah et al., 2018; Abotorabi et al., 2023; Ábrók et al., 2019; Ahmad, 2015; Akpaka et al., 2022; Al Abbas et al., 2022; Al Benwan and Al Banwan, 2024; Al Romaihi et al., 2018; Al-Matary et al., 2019; Al-Subol et al., 2022; Al-Tulaibawi, 2019; Alani and AlMeani, 2022; Alemán et al., 2022; Alhhazmi et al., 2016; Ali et al., 2022; Ali et al., 2020; Alp et al., 2016; Alzayer et al., 2023; AlZuheiri et al., 2021; Asghar et al., 2020; Bae et al., 2022; Baldan et al., 2021; Balkhi et al., 2018; Belard et al., 2015; Bergal et al., 2015; Bhola et al., 2020; Biedenbach et al., 2015; Biobaku Oluwafunmilola et al., 2017; Bitew et al., 2021; Björnsdóttir et al., 2019; Bob-Manuel et al., 2021; Bolukaoto et al., 2015; Brigtsen et al., 2015; Burcham et al., 2019; Campisi et al., 2016; Carvalhaes et al., 2022; Chang et al., 2014; Cheng et al., 2020; Choi et al., 2021; Cooper et al., 2016; del Pilar Crespo-Ortiz et al., 2014; Creti et al., 2017; Dashtizade et al., 2020; de Figueiredo et al., 2021; Dehbashi et al., 2015; Dehdashtian et al., 2021; Dilrukshi et al., 2021; Dilrukshi et al., 2023; Dobrut et al., 2022; Dong et al., 2017; Doumith et al., 2017; Dube et al., 2022; Duncan et al., 2017; Dutra et al., 2014; Ebrahem et al., 2023; El Shahaway et al., 2019; El-Gendy et al., 2021; Emaneini et al., 2016; Emaneini et al., 2014; Eskandarian et al., 2015; Evangelia et al., 2015; Fahim et al., 2022; Felemban et al., 2019; Feuerschuette et al., 2022; Flamm et al., 2014; Florindo et al., 2014; Foster-Nyarko et al., 2016; Frej-Mądrzak et al., 2020; Fröhlicher et al., 2014; Fujiya et al., 2019; Gajic et al., 2019; Gao et al., 2019; Ge et al., 2021; Ghamari et al., 2022; Gharabeigi et al., 2023; Gherardi et al., 2014; Girma et al., 2020; Gizachew et al., 2018; Gizachew et al., 2020; Gogoi et al., 2021; Gomi et al., 2019; Goudarzi et al., 2015; Goudarzi and Navidinia, 2019; Graux et al., 2021; Guan et al., 2018; Guo D. et al., 2018; Guo et al., 2019; Guo et al., 2016; Guo Y. et al., 2018; Hadavand et al., 2015; Haimbodi et al., 2021; Hamad et al., 2023; Hayes et al., 2017; Hays et al., 2016; Hernandez et al., 2022; Hirai et al., 2020; Hiriote et al., 2017; Hon et al., 2020; Horn et al., 2021; Houri et al., 2017; Hsu et al., 2023a, 2023b; Hsueh, 2015; Husen et al., 2023; Ikebe et al., 2015; Ikebe et al., 2023; Iweriebor et al., 2023; Jalalifar et al., 2019; Jamrozy et al., 2020; Ji et al., 2017; Jiang et al., 2016; Jiang et al., 2017; Jiao et al., 2022; Jisuvei et al., 2020; Jones et al., 2022; Kaminska et al., 2020; Kang et al., 2017; Kao et al., 2019; Kardos et al., 2019; Karim et al., 2019; Karlowsky et al., 2014; Karlowsky et al., 2016; Karlowsky et al., 2015; Karlowsky et al., 2017; Kawaguchiya et al., 2022; Kekic et al., 2021; Kernéis et al., 2017; Khan et al., 2015; Khan et al., 2023; Khodaei et al., 2018; Kitamura et al., 2019; Ko et al., 2021; Kumalo et al., 2023; Laczeski et al., 2014; Lagunas-Rangel, 2018; Lee et al., 2022; Lee and Lai, 2015; Leykun et al., 2021; Li et al., 2018; Li G. et al., 2022; Li J. et al., 2019; Li X. et al., 2019; Li X. et al., 2022; Li et al., 2023; Li Y. et al., 2019; Liu et al., 2021; Liu et al., 2022; Liu et al., 2023; Lopes et al., 2017; Lu et al., 2016; Lu et al., 2018; Ma et al., 2021; Madrid et al., 2018; Majigo et al., 2023; Majigo et al., 2022; Malek-Jafarian et al., 2015; Malița et al., 2023; Martins et al., 2017; Matani et al., 2016; Mathur et al., 2014; de Melo et al., 2016; Mendes et al., 2015a, 2015b; Metcalf et al., 2017; Miloshevski and Miloshevska, 2015; Minotti et al., 2023; Mišić et al., 2018; Mohamed, 2023; Mohamed et al., 2020; Mohamed et al., 2024; Moltó-García et al., 2016; Morfin-Otero et al., 2015; Moroi et al., 2019; Morozumi et al., 2014; Motallebirad et al., 2021a, 2021b; Mousavi et al., 2016; Mubanga et al., 2015; Mudzana et al., 2021; Mudzikati and Dramowski, 2015; Mukesi et al., 2019; Mulu et al., 2015; Mwei et al., 2018; Nabavinia et al., 2020; Nagano et al., 2019; Newland et al., 2020; Ngom et al., 2023; Ngonzi et al., 2018; Njoku et al., 2017; Nkembe et al., 2018; Novosak et al., 2020; Numanović et al., 2017; de Oliveira Luiz et al., 2019; Ojo et al., 2019; Palacios-Saucedo et al., 2022; Panahi et al., 2023; Perim et al., 2015; Perme et al., 2020; Pfaller et al., 2016; Piccinelli et al., 2015; Piérard and Stone, 2021; Pimentel et al., 2016; Proudmore et al., 2023; Qadi et al., 2021; Qiu et al., 2021; Rasamiravaka et al., 2016; Renteria et al., 2014; Rostami et al., 2021; Saad et al., 2018; Safari et al., 2021; Saffar et al., 2016; Sahraee et al., 2019; Said et al., 2019; Santana et al., 2020; Sapugahawatte et al., 2022; Schuab et al., 2015; Shabayek and Abdalla, 2014; Shadbad et al., 2020; Shen et al., 2019; Shipitsyna et al., 2020; Shrestha et al., 2020; Sigaúque et al., 2018; Simoni et al., 2018; Slotved et al., 2021; Soares et al., 2013; Stewart et al., 2020; Størdal et al., 2022; Suhaimi et al., 2017; Sulung et al., 2023; Swann et al., 2014; Tan et al., 2022; Tang et al., 2020; Teatero et al., 2015a; Teatero et al., 2017; Teatero et al., 2015b; Tesfaye et al., 2022; Tsai et al., 2019; Tulyaprawat et al., 2021; Van Du et al., 2021; Venkatnarayan et al., 2014; Vuillemin et al., 2021; Wang et al., 2015a, 2015b; Wang S. et al., 2018; Wang X. et al., 2018; Wang Y-H. et al., 2015; Wang et al., 2014; Warrier et al., 2022; Wataradee et al., 2023; Wilkie et al., 2019; Williams et al., 2023; Woldu et al., 2014; Wu et al., 2019; Yan et al., 2016; Yayan et al., 2015; Yong et al., 2015; Yoon et al., 2015; Yu et al., 2021; Zakerifar et al., 2023; Zeng et al., 2016; Zhang et al., 2015; Zhang et al., 2021; Zhang et al., 2022; Zhang et al., 2023; Zhou et al., 2022; Zhou et al., 2023). Supplementary Table 1 presents the detailed characteristics of these studies and extracted data. The PRISMA flowchart, presented in Figure 1, summarizes the screening and selection process for the included presagers.

### 3.2 Study characteristics

The reports came from 57 countries (Australia, Nigeria, Iran, Japan, Serbia, Brazil, Cameroon, Portugal, Argentina, Poland, Malaysia, Taiwan, Switzerland, Norway, Indonesia, Kenya, Tanzania, Ethiopia, Bahrain, Palestinian Territories, Namibia, Bosnia & Herzegovina, Canada, Hong Kong SAR China, Unknown, Germany, Botswana, Nepal, Saudi Arabia, Italy, Ireland, Kuwait, Qatar, Thailand, Mozambique, Egypt, Syria, United States, Hungary, India, Sri Lanka, Iceland, Slovenia, Mexico, Colombia, China, France, Pakistan, Trinidad & Tobago, United Arab Emirates, Malawi, Gambia, Lesotho, South Africa, Iraq, Vietnam, Zimbabwe). Six continents [Oceania, Africa, Asia, Europe, North America, South America, and NA (Not Applicable)]. NA indicates studies that include data from multiple continents rather than being confined to a single geographic region. These studies were included in this meta-analysis. Reports cover the years from 2013 to 2023.

### 3.3 Results of syntheses

#### 3.3.1 Comprehensive antibiotic-specific meta-analysis results

The proportion of penicillin resistance in 108 reports, with 979 resistant isolates among 68,461 investigated isolates, was 0.017 (0.013, 0.024), and heterogeneity between reports was not significant (*I*^2^ = 0.00%, *p* > 0.999). The proportion of ampicillin resistance in 63 reports, with 622 resistant isolates among 15,558 investigated isolates, was 0.031 (0.02, 0.046), and heterogeneity between reports was insignificant (*I*^2^ = 0.00%, *p* = 0.993). The proportion of SAM resistance through six reports, with four resistant isolates among 229 investigated isolates, was 0.043 (0.012, 0.14), and heterogeneity between reports was insignificant (*I*^2^ = 49.25%, *p* = 0.080). The proportion of cefazolin-resistant isolates in 11 reports, with 39 resistant isolates among the 1744 isolates, was 0.013 (0.002, 0.079), and the heterogeneity between reports was insignificant (*I*^2^ = 0.00%, *p* = 0.694). The proportion of clindamycin-resistant isolates among 108 reports, with 14,263 resistant isolates among 51,066 investigated isolates, was 0.293 (0.269, 0.319), and heterogeneity between reports was significant (*I*^2^ = 83.33%, *p* = 0.001). The proportion of erythromycin resistance in 217 reports, with 15,548 resistant isolates among 47,934 investigated isolates, was 0.35 (0.324, 0.378), and the heterogeneity between reports was significant (*I*^2^ = 95.76%, *p* = 0.001). The proportion of vancomycin resistance in 93 reports, with 604 resistant isolates among 45,009 investigated isolates, was 0.014 (0.01, 0.02), and the heterogeneity between reports was significant (*I*^2^ = 87.35%, *p* = 0.001). The proportion of ceftriaxone resistance in 77 reports, with 724 resistant isolates among 30,196 investigated isolates, was 0.062 (0.039, 0.097), and the heterogeneity between reports was significant (*I*^2^ = 91.54%, *p* = 0.001). The proportion of amoxicillin-resistant isolates in 10 reports, with 43 resistant isolates among the 9,837 investigated isolates, was 0.035 (0.006, 0.178), and the heterogeneity between reports was significant (*I*^2^ = 91.79%, *p* = 0.001). The proportion of cefuroxime resistance through 17 reports, with 53.3 resistant isolates among 4,806 investigated isolates, was 0.03 (0.012, 0.07), and heterogeneity between reports was significant (*I*^2^ = 82.72%, *p* = 0.001). The proportion of cefotaxime resistance through 33 reports, with 367 resistant isolates among 6,595 investigated isolates, was 0.032 (0.017, 0.06), and heterogeneity between reports was not significant (*I*^2^ = 0.00%, *p* > 0.999). The proportion of meropenem resistance in 13 reports, with 24 resistant isolates among 26,329 investigated isolates, was 0.007 (0.003, 0.017), and heterogeneity between reports was not significant (*I*^2^ = 0.00%, *p* > 0.999). The proportion of imipenem resistance through 11 reports, with 26 resistant isolates among 384 investigated isolates, was 0.065 (0.023, 0.166), and heterogeneity between reports was significant (*I*^2^ = 58.66%, *p* = 0.007). The proportion of azithromycin resistance in 21 reports, with 3,580 resistant isolates among 21,334 investigated isolates, was 0.41 (0.28, 0.554), and the heterogeneity between reports was significant (*I*^2^ = 97.29%, *p* = 0.001). The proportion of clarithromycin-resistant isolates in 11 reports, with 480 resistant isolates among the 1,468 investigated isolates, was 0.434 (0.303, 0.575), and the heterogeneity between reports was significant (*I*^2^ = 93.05%, *p* = 0.001). The proportion of erythromycin resistance through seven reports, with 300 resistant isolates among 554 investigated isolates, was 0.597 (0.31, 0.829), and heterogeneity between reports was significant (*I*^2^ = 96.27%, *p* = 0.001). The proportion of tetracycline resistance in 62 reports, with 21,931 resistant isolates among 28,322 investigated isolates, was 0.801 (0.771, 0.828), and heterogeneity between reports was significant (*I*^2^ = 80.79%, *p* = 0.001). The proportion of doxycycline resistance through five reports, with 188 resistant isolates among 372 investigated isolates, was 0.649 (0.371, 0.853), and heterogeneity between reports was significant (*I*^2^ = 93.66%, *p* = 0.001). The proportion of TMP-SMX resistant isolates in 29 reports, with 371 resistant isolates among the 5,705 investigated isolates, was 0.213 (0.107, 0.378), and the heterogeneity between reports was significant (*I*^2^ = 93.33%, *p* = 0.001). The proportion of ciprofloxacin resistance through 42 reports, with 502.9 resistant isolates among 3,558 investigated isolates, was 0.179 (0.127, 0.246), and heterogeneity between reports was significant (*I*^2^ = 87.16%, *p* = 0.001). The proportion of levofloxacin resistance in 62 reports, with 3,756 resistant isolates among 46,465 investigated isolates, was 0.086 (0.068, 0.108), and heterogeneity between reports was significant (*I*^2^ = 51.53%, *p* = 0.001). The proportion of gentamicin resistance through 32 reports, with 649 resistant isolates among 12,155 investigated isolates, was 0.19 (0.08, 0.389), and heterogeneity between reports was significant (*I*^2^ = 97.29%, *p* = 0.001). The proportion of linezolid resistance in 45 reports, with 17 resistant isolates among 18,117 investigated isolates, was 0.008 (0.006, 0.011), and heterogeneity between reports was not significant (*I*^2^ = 0.00%, *p* > 0.999). The proportion of daptomycin resistance in 22 reports, with four resistant isolates among 10,690 investigated isolates, was 0.003 (0.002, 0.007), and the heterogeneity between reports was insignificant (*I*^2^ = 5.23%, *p* = 0.390). The proportion of tigecycline-resistant isolates among the 30 reports, with zero resistant isolates among the 3,066 investigated isolates, was 0.007 (0.004, 0.012), and heterogeneity between reports was insignificant (*I*^2^ = 0.00%, *p* = 0.996). The proportion of nitrofurantoin-resistant isolates in the 12 reports, with 73 resistant isolates among the 627 investigated isolates, was 0.124 (0.055, 0.258), and the heterogeneity between reports was significant (*I*^2^ = 83.52%, *p* = 0.001). The proportion of ceftaroline resistance through four reports, with zero resistant isolates among 176 investigated isolates, was 0.012 (0.003, 0.048), and heterogeneity between reports was insignificant (*I*^2^ = 0.00%, *p* = 0.933). The proportion of tedizolid resistance through four reports, with zero resistant isolates among 5,213 investigated isolates, was 0.001 (0, 0.008), and heterogeneity between reports was insignificant (*I*^2^ = 49.62%, *p* = 0.114). The proportion of cefepime resistance in 25 reports, with 179 resistant isolates among the 5,231 investigated isolates, was 0.063 (0.027, 0.142), and heterogeneity between reports was significant (*I*^2^ = 89.76%, *p* = 0.001). The proportion of moxifloxacin resistance in 20 reports, with 110 resistant isolates among the 1,431 investigated isolates, was 0.063 (0.033, 0.12), and heterogeneity between reports was significant (*I*^2^ = 86.10%, *p* = 0.001). The proportion of oxacillin resistance in nine reports, with 41 resistant isolates among the 1,069 investigated isolates, was 0.062 (0.012, 0.261), and heterogeneity between reports was significant (*I*^2^ = 85.61%, *p* = 0.001). The proportion of teicoplanin resistance in eight reports, with three resistant isolates among the 1,274 investigated isolates, was 0.007 (0.003, 0.021), and heterogeneity between reports was insignificant (*I*^2^ = 0.00%, *p* = 0.882). The proportion of Q/D resistance in 15 reports, with 67 resistant isolates among the 1,260 investigated isolates, was 0.014 (0.003, 0.065), and heterogeneity between reports was insignificant (*I*^2^ = 0.00%, *p* = 0.472). The proportion of chloramphenicol resistance in 57 reports, with 500 resistant isolates among 10,245 investigated isolates, was 0.072 (0.048, 0.107), and the heterogeneity between reports was significant (*I*^2^ = 93.25%, *p* = 0.001). The proportion of cefditoren resistance in four reports, with 44 resistant isolates among 20,636 investigated isolates, was 0.003 (0, 0.238), and heterogeneity between reports was significant (*I*^2^ = 95.23%, *p* = 0.001). The proportion of norfloxacin resistance in nine reports, with 168 resistant isolates among the 865 investigated isolates, was 0.157 (0.084, 0.274), and the heterogeneity between reports was significant (*I*^2^ = 87.99%, *p* = 0.001). The proportion of AMC resistance in six reports, with 31 resistant isolates among the 2033 investigated isolates, was 0.196 (0.023, 0.713), and heterogeneity between reports was significant (*I*^2^ = 75.79%, *p* = 0.001). The proportion of cefoxitin resistance through four reports, with 14 resistant isolates among 184 investigated isolates, was 0.186 (0.031, 0.622), and heterogeneity between reports was significant (*I*^2^ = 81.58%, *p* = 0.001). The proportion of norfloxacin resistance in four reports, with 18 resistant isolates among the 499 investigated isolates, was 0.096 (0.006, 0.648), and the heterogeneity between reports was significant (*I*^2^ = 91.42%, *p* = 0.001). The proportion of ofloxacin resistance through six reports, with 98 resistant isolates among 292 investigated isolates, was 0.273 (0.049, 0.731), and heterogeneity between reports was significant (*I*^2^ = 94.61%, *p* = 0.001). The proportion of amikacin resistance in nine reports, with 652 resistant isolates among the 9,033 investigated isolates, was 0.196 (0.076, 0.422), and heterogeneity between reports was significant (*I*^2^ = 73.85%, *p* = 0.001). The proportion of nalidixic acid resistance through three reports, with 102 resistant isolates among 135 investigated isolates, was 0.749 (0.42, 0.925), and heterogeneity between reports was insignificant (*I*^2^ = 3.10%, *p* = 0.356).

**Figure 2:**
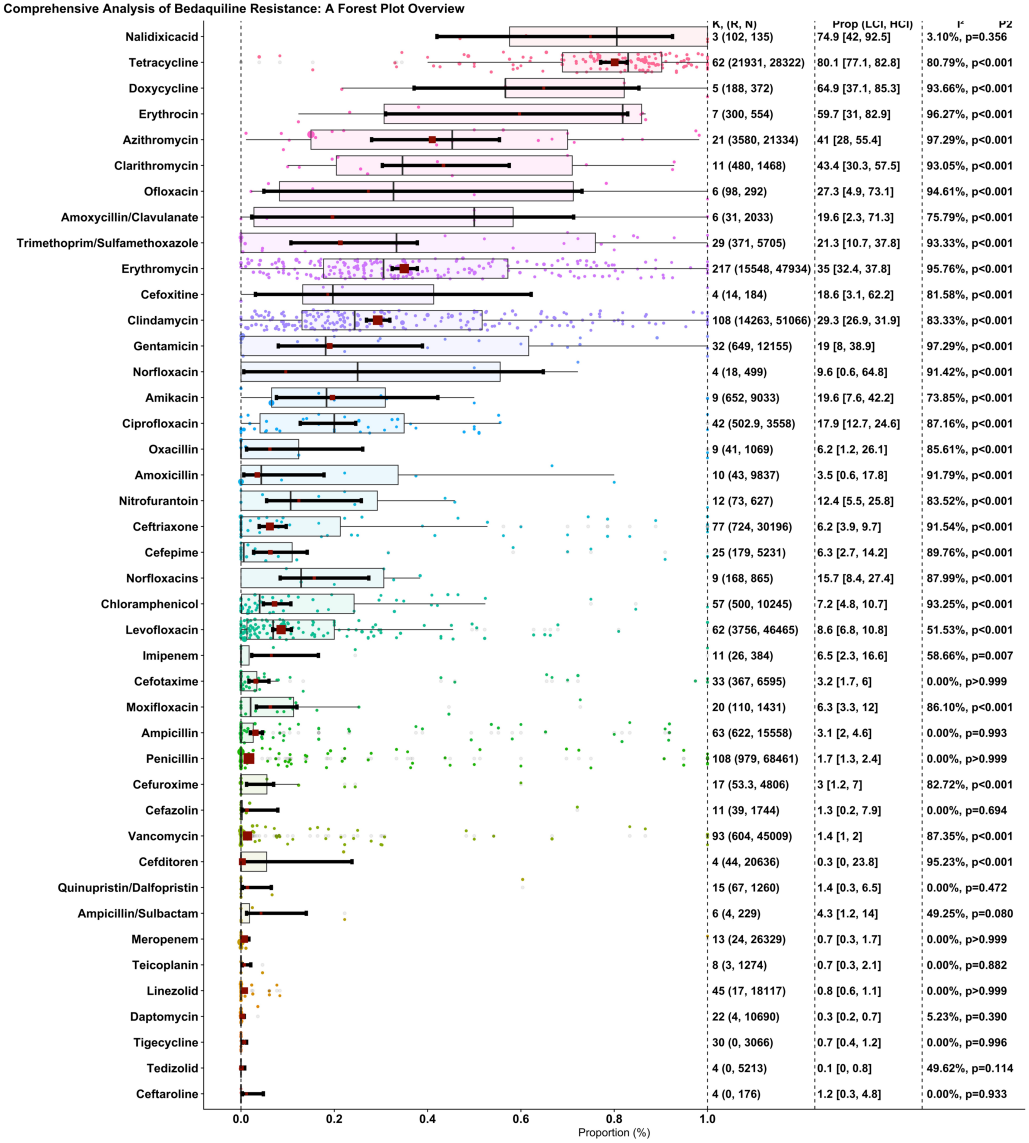
Overall, forest plot of the proportion of antibiotic resistance of GBS in individual studies. Each dot represents the resistance rate in a single study, with the size of the dot reflecting the sample size. The red square and error bars indicate the pooled estimate and 95% confidence interval.

Figure 2 shows a forest plot of the observed outcomes and the estimate based on the random-effects model. Table 1 details the antibiotic resistance patterns among GBS spp.

**Table 1:** Meta-analysis statistics of worldwide antibiotic resistance in GBS.

Antibiotic	*K* (*n*, *N*)	Proportion 95% CI (LCI, HCI)	*I* ^2^	*p*1	*p*2
Penicillin	216 (979, 68,461)	0.017 (0.013, 0.024)	89.08%	*p* < 0.001	*p* < 0.001
Ampicillin	126 (622, 15,558)	0.031 (0.020, 0.046)	90.80%	*p* < 0.001	*p* < 0.001
SAM	6 (4, 229)	0.043 (0.012, 0.140)	49.25%	*p* < 0.001	*p* = 0.080
Cefazolin	12 (39, 1744)	0.013 (0.002, 0.079)	92.99%	*p* < 0.001	*p* < 0.001
Clindamycin	216 (14,263, 51,066)	0.293 (0.269, 0.319)	96.10%	*p* < 0.001	*p* < 0.001
Erythromycin	221 (15,548, 47,934)	0.350 (0.324, 0.378)	96.02%	*p* < 0.001	*p* < 0.001
Vancomycin	186 (604, 45,009)	0.014 (0.010, 0.020)	88.77%	*p* < 0.001	*p* < 0.001
Ceftriaxone	78 (724, 30,196)	0.062 (0.039, 0.097)	91.66%	*p* < 0.001	*p* < 0.001
Amoxicillin	10 (43, 9,837)	0.035 (0.006, 0.178)	91.79%	*p* < 0.001	*p* < 0.001
Cefuroxime	17 (53.3, 4,806)	0.030 (0.012, 0.070)	82.72%	*p* < 0.001	*p* < 0.001
Cefotaxime	67 (367, 6,595)	0.032 (0.017, 0.060)	91.90%	*p* < 0.001	*p* < 0.001
Meropenem	27 (24, 26,329)	0.007 (0.003, 0.017)	64.91%	*p* < 0.001	*p* < 0.001
Imipenem	12 (26, 384)	0.065 (0.023, 0.166)	69.46%	*p* < 0.001	*p* < 0.001
Azithromycin	21 (3,580, 21,334)	0.410 (0.280, 0.554)	97.29%	*p* = 0.218	*p* < 0.001
Clarithromycin	12 (480, 1,468)	0.434 (0.303, 0.575)	94.11%	*p* = 0.357	*p* < 0.001
Erythrocin	7 (300, 554)	0.597 (0.310, 0.829)	96.27%	*p* = 0.519	*p* < 0.001
Tetracycline	124 (21,931, 28,322)	0.801 (0.771, 0.828)	95.82%	*p* < 0.001	*p* < 0.001
Doxycycline	5 (188, 372)	0.649 (0.371, 0.853)	93.66%	*p* = 0.293	*p* < 0.001
TMP-SMX	30 (371, 5,705)	0.213 (0.107, 0.378)	93.67%	*p* = 0.002	*p* < 0.001
Ciprofloxacin	43 (502.9, 3,558)	0.179 (0.127, 0.246)	90.42%	*p* < 0.001	*p* < 0.001
Levofloxacin	125 (3,756, 46,465)	0.086 (0.068, 0.108)	96.32%	*p* < 0.001	*p* < 0.001
Gentamicin	32 (649, 12,155)	0.190 (0.080, 0.389)	97.29%	*p* = 0.004	*p* < 0.001
Linezolid	90 (17, 18,117)	0.008 (0.006, 0.011)	20.92%	*p* < 0.001	*p* = 0.047
Daptomycin	23 (4, 10,690)	0.003 (0.002, 0.007)	42.57%	*p* < 0.001	*p* = 0.017
Tigecycline	30 (0, 3,066)	0.007 (0.004, 0.012)	0.00%	*p* < 0.001	*p* = 0.996
Nitrofurantoin	12 (73, 627)	0.124 (0.055, 0.258)	83.52%	*p* < 0.001	*p* < 0.001
Ceftaroline	4 (0, 176)	0.012 (0.003, 0.048)	0.00%	*p* < 0.001	*p* = 0.933
Tedizolid	4 (0, 5,213)	0.001 (0.000, 0.008)	49.62%	*p* < 0.001	*p* = 0.114
Cefepime	26 (179, 5,231)	0.063 (0.027, 0.142)	90.32%	*p* < 0.001	*p* < 0.001
Moxifloxacin	20 (110, 1,431)	0.063 (0.033, 0.120)	86.10%	*p* < 0.001	*p* < 0.001
Oxacillin	9 (41, 1,069)	0.062 (0.012, 0.261)	85.61%	*p* = 0.001	*p* < 0.001
Teicoplanin	9 (3, 1,274)	0.007 (0.003, 0.021)	37.75%	*p* < 0.001	*p* = 0.117
QD	16 (67, 1,260)	0.014 (0.003, 0.065)	90.81%	*p* < 0.001	*p* < 0.001
Chloramphenicol	57 (500, 10,245)	0.072 (0.048, 0.107)	93.25%	*p* < 0.001	*p* < 0.001
Cefditoren	4 (44, 20,636)	0.003 (0.000, 0.238)	95.23%	*p* = 0.014	*p* < 0.001
Norfloxacins	9 (168, 865)	0.157 (0.084, 0.274)	87.99%	*p* < 0.001	*p* < 0.001
AMC	7 (31, 2033)	0.196 (0.023, 0.713)	88.08%	*p* = 0.234	*p* < 0.001
Cefoxitine	4 (14, 184)	0.186 (0.031, 0.622)	81.58%	*p* = 0.142	*p* < 0.001
Norfloxacin	4 (18, 499)	0.096 (0.006, 0.648)	91.42%	*p* = 0.123	*p* < 0.001
Ofloxacin	6 (98, 292)	0.273 (0.049, 0.731)	94.61%	*p* = 0.332	*p* < 0.001
Amikacin	10 (652, 9,033)	0.196 (0.076, 0.422)	84.66%	*p* = 0.012	*p* < 0.001
Nalidixicacid	4 (102, 135)	0.749 (0.420, 0.925)	77.42%	*p* = 0.130	*p* = 0.004

*K, number of reports; n, number of resistant isolates; N, number of total isolates; LCI, 95% lower limit confidence interval; HCI, 95% higher limit confidence interval; p1, p-value of difference from zero resistance rate; p2, p-value of heterogeneity between reports; QD, quinupristin/dalfopristin; AMC, amoxicillin-clavulanic acid; SAM, ampicillin/sulbactam; TMP-SMX, Trimethoprim/sulfamethoxazole.*

### 3.4 Subgroup analysis

**Figure 3:**
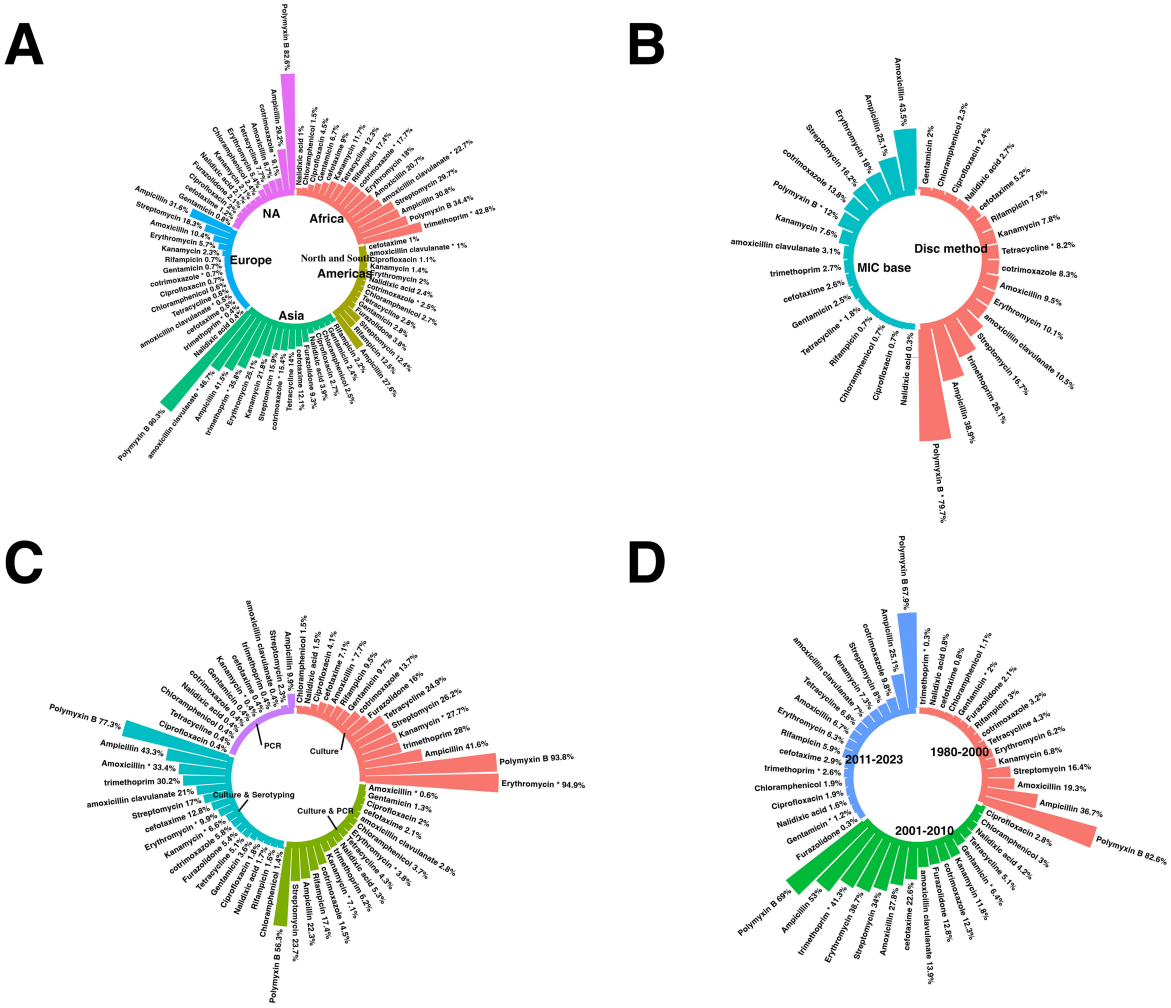
Worldwide map for prevalence of antibiotics resistance in GBS.

**Figure 4:**
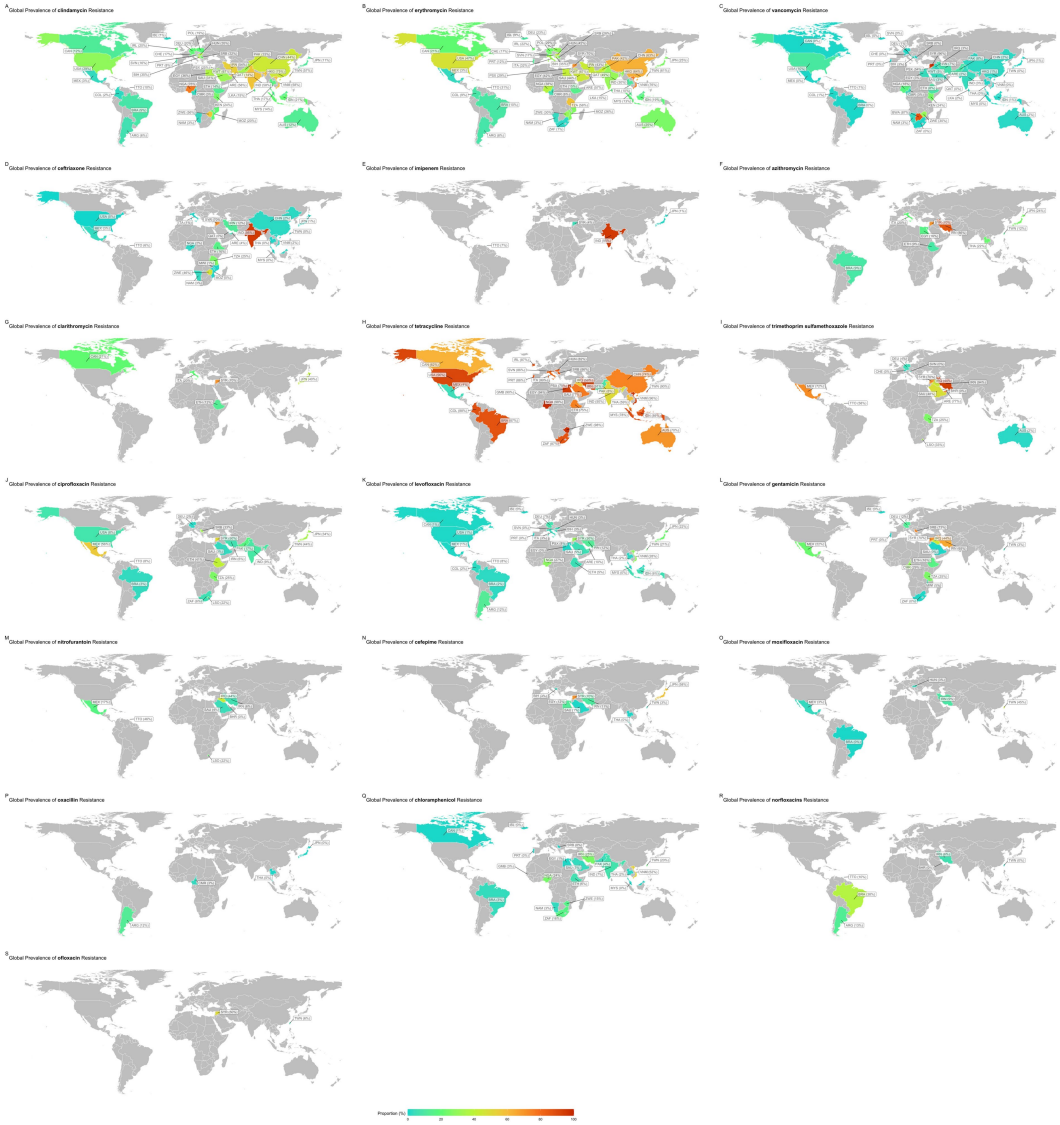
Subgroup analysis results of GBS isolates. **(A)** Compression of the prevalence of antibiotic-resistant in GBS isolates between continents. **(B)** Compression of the prevalence of antibiotic-resistant in GBS isolates between AST methods. **(C)** Compression of the prevalence of antibiotic-resistant in GBS isolates based on different bacterial diagnostic methods. **(D)** Compression of the prevalence of antibiotic-resistant in GBS based on years.

This passage offers a comprehensive overview of the subgroup analyses of antibiotic resistance. Supplementary Table 2 and Figures 3, 4 present detailed data on the subgroups, offering a complete view of resistance patterns and trends. The analyses investigated differences in resistance rates based on geography, AST methods, trends over time, and study quality.

#### 3.4.1 Subgroup analysis based on countries

The subgroup analysis revealed a statistically significant disparity in the prevalence of antibiotic resistance, including resistance to chloramphenicol, ciprofloxacin, clindamycin, erythromycin, gentamicin, imipenem, levofloxacin, moxifloxacin, norfloxacin, ofloxacin, tetracycline, and vancomycin. Serbia had the lowest chloramphenicol resistance rate, with a prevalence of 0.1%. Conversely, Vietnam had the highest resistance rate, with a prevalence of 52.4%.

Germany had the lowest prevalence rate of ciprofloxacin resistance, 1.5%. Conversely, Mexico had the highest resistance rate, 55.6%.

Iceland had the lowest rate of resistance to clindamycin, with a prevalence rate of 1%. Conversely, Nigeria showed the highest resistance rate (76.2%).

South Africa had the lowest erythromycin resistance rate, at 1.4%. Conversely, the country with the highest resistance rate, at 88.9%, was unknown.

Portugal has the lowest prevalence of gentamicin (0.3%). In contrast, Serbia showed the highest resistance rate (72.7%).

Japan had the lowest imipenem resistance rate, at 1.1%. Conversely, India had the highest resistance rate, at 95%.

Portugal had a 0.1% prevalence of resistance to the antibiotic levofloxacin. Conversely, Syria had the highest resistance rate, at 30%.

Brazil has the lowest resistance rate to moxifloxacin, with a prevalence rate of 0.4%. Conversely, Taiwan had the highest resistance rate, with a prevalence of 44.6%.

Bahrain had the lowest resistance rate for the antibiotic norfloxacin, with a prevalence rate of 0.4%. Conversely, Brazil showed the highest rate of resistance (37.5%).

Taiwan had the lowest prevalence rate of resistance to the antibiotic ofloxacin, at 5.8%. Conversely, Syria had the highest resistance, at 50%. Mexico had the lowest rate of resistance to tetracycline prevalence, at 4.3%. Conversely, Nigeria had the highest resistance rate, with a prevalence of 98.5%. Switzerland had the lowest vancomycin resistance rate at 0%. Conversely, Syria had the highest resistance rate, with a prevalence of 95.5% (Figure 3).

Supplementary Table 4 provides a subgroup analysis highlighting the countries with the highest and lowest resistance patterns for each antibiotic.

#### 3.4.2 Subgroup analysis based on continents

The subgroup analysis revealed a statistically significant disparity in the prevalence of antibiotic resistance, including resistance to azithromycin, cefuroxime, chloramphenicol, clindamycin, erythromycin, levofloxacin, moxifloxacin, norfloxacin, ofloxacin, TMP-SMX, and vancomycin. For the antibiotic azithromycin, the continent with the lowest resistance rate was the Americas, with a prevalence of 9.1%. Conversely, the continent with the highest resistance rate was NA, with a prevalence of 72.2%.

Europe had the lowest resistance rate to cefuroxime, with a prevalence of 0.2%. Conversely, the continent with the highest resistance rate was the Americas, which had a prevalence of 11.7%.

Europe had the lowest prevalence of chloramphenicol use (0.4%), while Asia had the highest prevalence (14.1%).

The Americas had the lowest resistance rate to clindamycin, at 11.5%. Conversely, Asia had the highest resistance rate, at 37.7%.

For the antibiotic erythromycin, the continent with the lowest resistance rate was the Americas, which had a prevalence of 14.9%. Conversely, Asia had the highest resistance rate, with a prevalence of 43.4%. Europe had the lowest prevalence of levofloxacin (1.7%), while Asia had the highest prevalence (12.7%). The continent with the lowest resistance rate to moxifloxacin was the Americas, with a prevalence of 0.8%. Conversely, the continents with the highest resistance rates were observed in Asia, with a prevalence of 22.6%. Asia had the lowest prevalence of norfloxacin (1.1%).

Conversely, NA had the highest prevalence (29%). Asia had the lowest resistance rate to the antibiotic ofloxacin, with a prevalence rate of 11.2%. Conversely, the continent with the highest resistance rate was NA, with a prevalence of 78.4%. Europe had the lowest resistance rate to TMP-SMX, with a prevalence rate of 0.8%. Conversely, the continent with the highest resistance rate was the Americas, with a prevalence rate of 61.5%.

Europe had the lowest vancomycin prevalence (0.4%), and Africa had the highest (8.5%) (Figure 4A).

#### 3.4.3 Subgroup analysis based on AST category

The subgroup analysis revealed a statistically significant disparity in the prevalence of antibiotic resistance to amikacin and amoxicillin. Among the various AST categories are clavulanate, ceftriaxone, ciprofloxacin, clindamycin, doxycycline, erythromycin, imipenem, TMP-SMX, and vancomycin. The disc method was the AST category with the lowest resistance to amikacin, with a prevalence rate of 11.1%. Conversely, the AST category with the highest resistance rate (99.3%) was observed in the MIC database. For the AMC, the AST category with the lowest resistance rate was the disc method, which exhibited a prevalence rate of 27.6%. Conversely, the AST category with the highest resistance rate was observed in other categories, with a prevalence rate of 97.6%.

The combination method had the lowest resistance rate for ceftriaxone, with a prevalence of 0.6%. Conversely, the disc method had the highest resistance rate, with a prevalence of 14.1%.

For ciprofloxacin, the AST category with the lowest resistance rate was MIC base, with a prevalence rate of 4.7%. Conversely, the AST category with the highest resistance rate was the disc method, with a prevalence of 24.2%.

For clindamycin, the AST category with the lowest resistance rate was MIC base, with a prevalence rate of 27.2%. Conversely, the AST category with the highest resistance rate was observed in other categories, with a prevalence rate of 46.8%.

For doxycycline, the AST category with the lowest resistance rate was the MIC, with a prevalence of 52.9%. Conversely, the AST category with the highest resistance rate (99.2%) was observed for the disc method.

For erythromycin, the AST category with the lowest resistance rate was the combination method, with a prevalence of 25.5%. Conversely, the AST category with the highest resistance rate was observed in other categories, with a prevalence rate of 52.8%.

For imipenem, the AST category with the lowest resistance rate was MIC base, with a prevalence rate of 1.1%. Conversely, the AST category with the highest resistance rate was the disc method, with a prevalence of 10.5%.

For TMP-SMX, the AST category with the lowest resistance rate was MIC, with a prevalence rate of 0.9%. Conversely, the AST category with the highest resistance rate was observed in other categories, with a prevalence rate of 74.8%.

For vancomycin, the AST category with the lowest resistance rate was MIC base, with a prevalence rate of 0.7%. Conversely, the AST category with the highest resistance rate was observed for the disc method, with a prevalence of 2.6% (Figure 4B).

#### 3.4.4 Subgroup analysis based on different bacterial diagnostic methods

The data highlights variability in resistance rates depending on the diagnostic technique used. When culture methods were employed, resistance was notably higher for antibiotics such as ampicillin (43.7%) and amoxicillin (33.8%). Trimethoprim (30.2%) and amoxicillin-clavulanate (21%) showed significant resistance in culture-based approaches.

PCR-based diagnostics, however, revealed differing resistance rates, with certain antibiotics demonstrating lower or less reported resistance levels. When culture and serotyping or PCR were combined, resistance patterns diversified further, reflecting the sensitivity and specificity of these diagnostic techniques. The distribution underscores the influence of diagnostic methodologies on reported antibiotic resistance rates, emphasizing the importance of standardized approaches for reliable assessments (Figure 4C).

#### 3.4.5 Subgroup analysis based on year-group

Subgroup analysis revealed a statistically significant disparity in the prevalence of antibiotic resistance, including resistance to cefditoren, ceftriaxone, clindamycin, erythromycin, levofloxacin, and moxifloxacin. For cefditoren, the year with the lowest resistance rate was 2020–2023, with a prevalence rate of 0%. Conversely, the year with the highest resistance rate was 2013–2019, with a prevalence rate of 6%.

For the antibiotic ceftriaxone, the year with the lowest resistance rate was 2013–2019, with a prevalence rate of 3.1%. Conversely, the year with the highest resistance rate was 2020–2023, with a prevalence rate of 9.7%.

For the antibiotic clindamycin, the year group with the lowest resistance rate was 2013–2019, with a prevalence rate of 24.6%. Conversely, the year with the highest resistance rate was 2020–2023, with a prevalence rate of 32.3%.

For the antibiotic erythromycin, the year group with the lowest resistance rate was 2013–2019, with a prevalence rate of 29.9%. Conversely, the year group with the highest resistance rate was observed in 2020–2023, with a prevalence rate of 38.8%.

For the antibiotic levofloxacin, the year group with the lowest resistance rate was 2013–2019, with a prevalence rate of 4.1%. Conversely, the year group with the highest resistance rate was observed in 2020–2023, with a prevalence rate of 13.1%.

For the antibiotic moxifloxacin, the year group with the lowest resistance rate was 2013–2019, with a prevalence rate of 1.8%. Conversely, the year group with the highest resistance rate was observed in 2020–2023, with a prevalence rate of 10.9% (Figure 4D).

#### 3.4.6 Subgroup analysis based on quality group

Subgroup analysis revealed a statistically significant disparity in the prevalence of antibiotic resistance, including amikacin, cefditoren, cefepime, chloramphenicol, clindamycin, norfloxacin, and tetracycline. For amikacin, the quality group with the lowest resistance rate was low, with a prevalence rate of 9.7%. Conversely, the quality group with the highest resistance rate was observed to be at some risk, with a prevalence rate of 77.1%.

The group with the lowest resistance rate to cefditoren was at risk, with a prevalence rate of 0%. Conversely, the high-risk group had the highest resistance rate observed in high risk, with a prevalence rate of 22%. For cefepime, the quality group with the lowest resistance rate was in the low-risk category, with a prevalence rate of 4.4%. Conversely, the quality group with the highest resistance rate was observed in the Some-Risk category, with a prevalence rate of 59%.

For the antibiotic chloramphenicol, the quality group with the lowest resistance rate was low-risk, with a prevalence rate of 6%. Conversely, the quality group with the highest resistance rate was observed to be at some risk, with a prevalence of 32.9%.

For the antibiotic clindamycin, the quality group with the lowest resistance rate was at high risk, with a prevalence rate of 9.9%. Conversely, the quality group with the highest resistance rate was observed to be at some risk, with a prevalence of 34.6%.

For norfloxacin, the quality group with the lowest resistance rate was at risk, with a prevalence rate of 0.5%. Conversely, the low-risk group had the highest resistance rate observed in low risk, with a prevalence rate of 18.5%.

For the antibiotic tetracycline, the quality group with the lowest resistance rate was at high risk, with a prevalence of 7.8%. Conversely, the low-risk group had the highest resistance rate observed in low risk, with a prevalence rate of 81.8%.

### 3.5 Risk of bias assessment

Overall, 266 studies were assessed for methodological quality using the JBI checklist. Of the included studies, 209 (78.6%) were classified as low risk, demonstrating strong methodological rigor with minimal risk of bias. Forty-two studies (15.8%) were identified as having some concerns, indicating minor methodological limitations that may affect the reliability of findings. Additionally, 15 studies (5.6%) were classified as high risk, highlighting significant methodological weaknesses that could impact study validity and interpretation. Overall, the majority of studies exhibited a low risk of bias, ensuring a high level of confidence in the synthesized evidence. However, the presence of studies with some concerns or a high risk of bias suggests the need for careful interpretation of findings, particularly in areas where methodological limitations may have influenced results. The results of this assessment are summarized in Supplementary Table 3, which provides a detailed risk of bias evaluation for each included study.

### 3.6 Meta-regression

**Figure 5:**
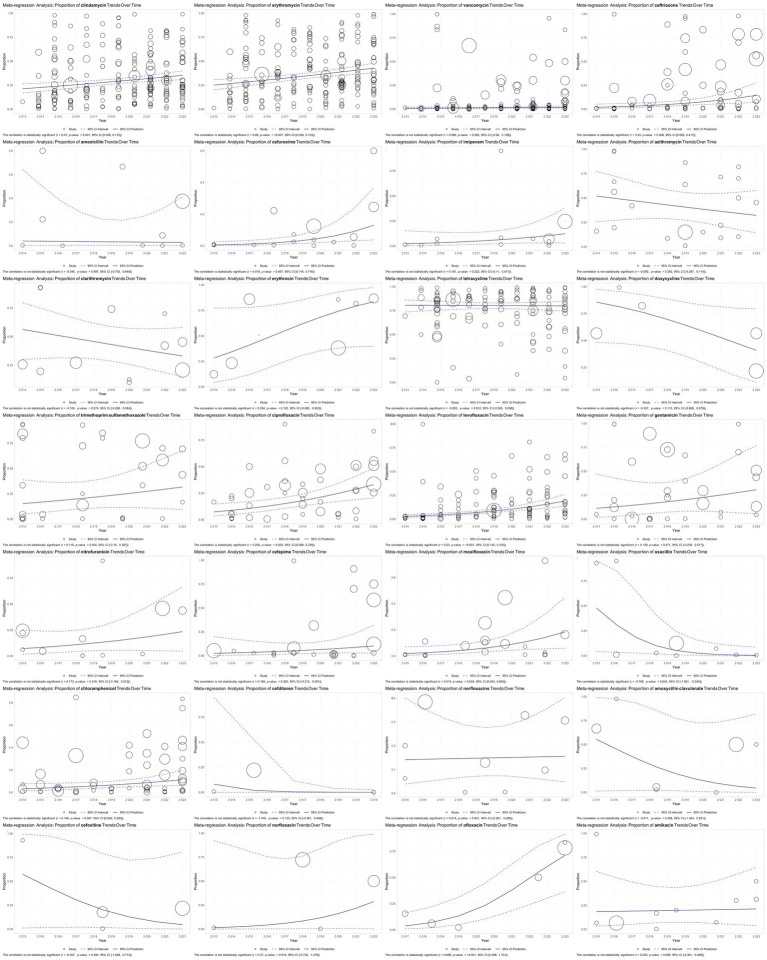
Meta-regression analysis of antibiotic resistance in GBS isolates over time. The results are visualized in scatter plots, illustrating the trend in the proportion of antibiotic-resistant GBS isolates over the years. The analysis revealed significant increases in resistance for several antibiotics, including clindamycin, erythromycin, ceftriaxone, cefuroxime, ciprofloxacin, levofloxacin, moxifloxacin, chloramphenicol, and ofloxacin, with notably steep increases in cefuroxime, moxifloxacin, and ofloxacin. Conversely, oxacillin showed a significant decrease in resistance over time.

The meta-regression analysis revealed a statistically significant positive correlation between resistance rates over the years for several antibiotics, indicating an increasing trend in resistance. Notable findings include clindamycin [*r* = 0.07, *p* = 0.001, 95% CI (0.028, 0.112)], erythromycin [*r* = 0.08, *p* < 0.001, 95% CI (0.038, 0.123)], ceftriaxone [*r* = 0.24, *p* = 0.008, 95% CI (0.063, 0.417)], cefuroxime [*r* = 0.416, *p* = 0.007, 95% CI (0.116, 0.716)], ciprofloxacin [*r* = 0.204, *p* = 0.003, 95% CI (0.068, 0.339)], levofloxacin [*r* = 0.23, *p* < 0.001, 95% CI (0.145, 0.316)], moxifloxacin [*r* = 0.315, *p* = 0.034, 95% CI (0.024, 0.605)], chloramphenicol [*r* = 0.148, *p* = 0.047, 95% CI (0.002, 0.293)], and ofloxacin [*r* = 0.698, *p* < 0.001, 95% CI (0.296, 1.101)]. Among these, ofloxacin, cefuroxime, and moxifloxacin exhibited particularly steep increases in resistance over time. In contrast, oxacillin showed a statistically significant negative correlation with resistance rates over the years [*r* = −0.78, *p* = 0.040, 95% CI (−1.381, −0.034)], indicating a declining trend in resistance. These findings underscore the growing threat of antibiotic resistance, particularly for commonly used antibiotics while highlighting a rare positive trend in the decreasing resistance to oxacillin (Figure 5).

### 3.7 Publication bias assessment

The Egger and Begg tests were employed to assess funnel plot asymmetry and rank correlation, which indicate publication bias.

For antibiotics such as tetracycline (*p* = 0.055) and doxycycline (*p* < 0.001), the Egger test indicated varying levels of potential publication bias, with doxycycline suggesting more substantial evidence of bias due to its *p*-value below 0.05. The Begg test, however, yielded *p*-values above 0.05 for both antibiotics, suggesting less consistent evidence of bias. Similarly, ciprofloxacin (*p* = 0.040) and levofloxacin (*p* < 0.001) showed low *p*-values in the Egger test, supporting the likelihood of bias, while the Begg test was more mixed in its findings. The “Fail-Safe N” values were substantial for tetracycline (141,092) and levofloxacin (185,807), highlighting robust findings, whereas ciprofloxacin (5,542) and doxycycline (0) demonstrated lower thresholds.

For macrolides like azithromycin (*p* = 0.047) and clarithromycin (*p* = 0.084), the Egger test suggested borderline evidence of publication bias, while the Begg test returned higher *p*-values, indicating weaker support for bias. The “Fail-Safe N” values were moderate for azithromycin (Aijaz et al., 2023; Mohamed et al., 2024) and clarithromycin (Piccinelli et al., 2015), suggesting fewer studies would be needed to overturn the observed results. The “Trim and Fill” analysis adjusted the effect sizes to 0.410 for azithromycin and 0.434 for clarithromycin, reflecting the estimated true effect sizes after correcting for potential bias.

Other antibiotics, such as tigecycline (*p* = 0.003) and daptomycin (*p* = 0.025), also showed significant Egger test results, suggesting potential bias, with moderate “Fail-Safe N” values of 4,002 and 3,588, respectively. Their “Trim and Fill” adjusted effect sizes were 0.007 and 0.011, indicating minimal impact after correcting for bias.

Lastly, for agents like nitrofurantoin (*p* = 0.006) and cefepime (*p* = 0.007), the Egger test revealed significant results, while the Begg test returned mixed findings. The “Fail-Safe N” values were relatively low for nitrofurantoin (Mwambia, 2021) and moderate for cefepime (2,095), reflecting varying levels of robustness. Their adjusted effect sizes under Trim and Fill were 0.150 and 0.118, providing insights into the corrected estimates after addressing bias. Table 2 and Supplementary Figure 1 summarize antibiotic resistance trends in GBS isolates, combining detailed statistical analysis with visual representation to highlight resistance patterns and assess the robustness of the findings.

**Table 2:** Evaluation of publication bias in meta-analysis.

Antibiotic	Egger test	Begg test	Fail and Safe	Trim and Fill
Penicillin	*p* < 0.001	*p* < 0.001	192,532	0.047 (0.035, 0.062)
Ampicillin	*p* < 0.001	*p* < 0.001	50,472	0.065 (0.045, 0.092)
SAM	*p* = 0.588	*p* = 0.719	97	0.043 (0.012, 0.140)
Cefazolin	*p* = 0.090	*p* = 0.002	757	0.035 (0.008, 0.135)
Clindamycin	*p* < 0.001	*p* = 0.401	215,655	0.337 (0.309, 0.365)
Erythromycin	*p* = 0.008	*p* = 0.209	116,614	0.390 (0.361, 0.420)
Vancomycin	*p* < 0.001	*p* < 0.001	165,823	0.033 (0.024, 0.045)
Ceftriaxone	*p* < 0.001	*p* = 0.129	15,590	0.136 (0.090, 0.200)
Amoxicillin	*p* < 0.001	*p* = 0.156	285	0.035 (0.006, 0.178)
Cefuroxime	*p* < 0.001	*p* = 0.490	1,408	0.107 (0.047, 0.225)
Cefotaxime	*p* < 0.001	*p* < 0.001	14,741	0.065 (0.037, 0.110)
Meropenem	*p* = 0.984	*p* < 0.001	3,456	0.020 (0.008, 0.048)
Imipenem	*p* = 0.333	*p* = 0.046	314	0.134 (0.055, 0.293)
Azithromycin	*p* = 0.047	*p* = 0.242	4,199	0.410 (0.280, 0.554)
Clarithromycin	*p* = 0.084	*p* = 0.638	229	0.434 (0.303, 0.575)
Erythrocin	*p* = 0.326	*p* > 0.999	0	0.597 (0.310, 0.829)
Tetracycline	*p* = 0.055	*p* = 0.509	141,092	0.753 (0.719, 0.784)
Doxycycline	*p* < 0.001	*p* = 0.069	0	0.649 (0.371, 0.853)
TMP-SMX	*p* = 0.002	*p* = 0.479	442	0.277 (0.146, 0.463)
Ciprofloxacin	*p* = 0.040	*p* = 0.191	5,542	0.179 (0.127, 0.246)
Levofloxacin	*p* < 0.001	*p* < 0.001	185,807	0.141 (0.113, 0.176)
Gentamicin	*p* = 0.361	*p* = 0.570	1,782	0.190 (0.080, 0.389)
Linezolid	*p* < 0.001	*p* < 0.001	40,937	0.018 (0.013, 0.025)
Daptomycin	*p* = 0.025	*p* < 0.001	3,588	0.011 (0.005, 0.025)
Tigecycline	*p* = 0.003	*p* < 0.001	4,002	0.007 (0.004, 0.012)
Nitrofurantoin	*p* = 0.006	*p* = 0.737	301	0.150 (0.068, 0.300)
Ceftaroline	*p* = 0.518	*p* = 0.083	52	0.012 (0.003, 0.048)
Tedizolid	*p* = 0.024	*p* = 0.056	134	0.001 (0.000, 0.008)
Cefepime	*p* = 0.007	*p* = 0.480	2,095	0.118 (0.053, 0.242)
Moxifloxacin	*p* < 0.001	*p* = 0.537	1,857	0.115 (0.063, 0.200)
Oxacillin	*p* = 0.841	*p* = 0.046	278	0.062 (0.012, 0.261)
Teicoplanin	*p* = 0.002	*p* = 0.045	467	0.016 (0.006, 0.039)
Q/D	*p* < 0.001	*p* < 0.001	898	0.025 (0.007, 0.083)
Chloramphenicol	*p* < 0.001	*p* = 0.203	24,616	0.121 (0.083, 0.175)
Cefditoren	*p* = 0.048	*p* > 0.999	194	0.015 (0.000, 0.410)
Norfloxacins	*p* < 0.001	*p* = 0.477	433	0.226 (0.126, 0.372)
AMC	*p* = 0.505	*p* = 0.773	15	0.196 (0.023, 0.713)
Cefoxitine	*p* = 0.995	*p* > 0.999	16	0.186 (0.031, 0.622)
Norfloxacin	*p* < 0.001	*p* = 0.333	10	0.096 (0.006, 0.648)
Ofloxacin	*p* = 0.747	*p* > 0.999	10	0.273 (0.049, 0.731)
Amikacin	*p* = 0.490	*p* > 0.999	1,951	0.196 (0.076, 0.422)
Nalidixicacid	*p* = 0.005	*p* = 0.333	6	0.749 (0.420, 0.925)

This table provides a comprehensive assessment of potential publication bias in the meta-analysis using a range of statistical techniques. Included are statistics generated from Egger’s method, Begg’s method, the Fail-Safe N (NFS), and the Trim-and-Fill method. These methods are applied to investigate the presence of bias and its impact on the meta-analysis results, ensuring the robustness and reliability of the findings. QD, quinupristin/dalfopristin; AMC, amoxicillin-clavulanic acid; SAM, ampicillin/sulbactam; TMP-SMX, Trimethoprim/sulfamethoxazole.

## 4 Discussion

GBS, known as *Streptococcus agalactiae*, is a significant pathogen in neonates, pregnant women, and immunocompromised individuals (Shrestha et al., 2020). It can cause severe infections such as sepsis, pneumonia, meningitis in newborns and various invasive diseases in adults (Lin et al., 2021). Antibiotic resistance in GBS infections is an increasing concern in clinical settings (Hayes et al., 2020). Penicillin and ampicillin are the primary antibiotics used to treat GBS infections (Alotaibi et al., 2023). Although these drugs are highly effective, resistance to them remains rare. However, alternative antibiotics, such as erythromycin and clindamycin, are used for individuals allergic to penicillin (Nadeau and Edwards, 2019).

The resistance profile of GBS to various antibiotics is a critical consideration in treatment strategies (Di Renzo et al., 2015). Studies have shown that while GBS is resistant to certain antibiotics, such as gentamicin, erythromycin, and clindamycin, it remains susceptible to essential drugs, such as penicillin, cefuroxime, cefotaxime, and vancomycin (Mwambia, 2021; Kasem et al., 2024). This information is crucial for selecting appropriate antibiotics for managing GBS infections, ensuring effective treatment, and reducing the risk of complications (Alotaibi et al., 2023). The emergence of antibiotic-resistant strains, especially pathogens such as GBS, underscores the need for continuous monitoring and surveillance to track resistance patterns and inform clinical practice (Yang et al., 2024). This systematic review and meta-analysis provide a comprehensive overview of the antibiotic resistance rates of GBS isolates to various antibiotics, highlighting significant findings and trends.

First, this systematic review and meta-analysis successfully identified 334 eligible studies from 57 countries across six continents, representing a substantial and diverse dataset. Comprehensive geographical coverage underscores the global nature of GBS. The variations in antibiotic resistance rates across different regions and countries highlight the importance of localized strategies for combating antibiotic resistance, considering regional factors such as healthcare practices, antibiotic use, and socioeconomic conditions (Coque et al., 2023). The use of standards for antimicrobial susceptibility testing is crucial for this discussion.

Key findings indicated that resistance rates for penicillin and ampicillin were meager at 1.7 and 3.1%, respectively, with no significant heterogeneity observed. This finding reinforces the continued effectiveness of antibiotics for GBS. Similarly, vancomycin and linezolid showed low resistance rates (1.4 and 0.8%, respectively), suggesting their reliability for treating GBS infections. Meropenem and daptomycin also demonstrated resistance rates of 0.7 and 0.3%, respectively, indicating their strong efficacy against GBS.

Moderate resistance rates of 6.2 and 6.3% were observed for ceftriaxone and cefepime, respectively, along with significant heterogeneity, indicating varied efficacy across different regions or settings. Clindamycin and erythromycin, however, showed notably high resistance rates of 29.3 and 35%, respectively, with significant heterogeneity, raising concerns regarding their reliability in treating GBS infections. Tetracycline and doxycycline exhibited extremely high resistance rates of 80.1 and 64.9%, respectively, with significant heterogeneity, suggesting the limited utility of these antibiotics in treating GBS infections. Similarly, azithromycin and clarithromycin showed high resistance rates (41 and 43.4%, respectively), indicating widespread resistance.

Subgroup analyses have provided valuable insights into the evolving landscape of antibiotic resistance in GBS. The variations observed based on year groups, continental locations, countries, detection methods, antimicrobial susceptibility test guidelines, and risk of bias assessment demonstrated the dynamic nature of the resistance problem.

This comprehensive review and subgroup analysis emphasizes the complex and multifaceted nature of antibiotic resistance in GBS. These findings highlight the importance of localized data, standardized testing methods, and high-quality research in understanding and addressing antibiotic resistance. Effective management of GBS infections requires ongoing surveillance, tailored antibiotic stewardship programs, and continuous adaptation of treatment guidelines based on the latest evidence. By addressing these challenges, healthcare providers can better manage GBS infections and mitigate the impact of antibiotic resistance on public health.

Subgroup analysis revealed significant geographical variations in antibiotic resistance rates for GBS, underscoring the importance of local epidemiological data in informing treatment guidelines and antibiotic stewardship programs. The resistance rates for clindamycin, erythromycin, and tetracycline showed marked differences across regions.

The significant geographical variations in antibiotic resistance rates suggest that local factors, such as antibiotic prescription practices, public health policies, and access to healthcare resources, play crucial roles in shaping resistance patterns (Felemban et al., 2019; Iweriebor et al., 2023). Studies have shown that differences in antibiotic prescribing practices across regions contribute to varying resistance rates; for instance, countries with stringent antibiotic regulations and robust healthcare infrastructure often report lower resistance levels, whereas those with lax policies and limited healthcare resources face higher resistance challenges (Felemban et al., 2019; Iweriebor et al., 2023).

Significant heterogeneity was observed for several antibiotics, including clindamycin, erythromycin, and tetracycline. This variation suggests that regional antibiotic use policies and differences in bacterial strains may significantly influence resistance patterns. For example, research has demonstrated that GBS strains exhibit varying resistance levels depending on local prescribing habits and public health policies, which can contribute to increased resistance rates (Wang S. et al., 2018; Hsu et al., 2021). Furthermore, antibiotic resistance tends to be higher in regions with poor healthcare access and inadequate regulation of antibiotic sales, leading to self-medication and misuse (Chen et al., 2023; Campisi et al., 2016). Therefore, addressing local antibiotic use policies and strengthening healthcare infrastructure is essential for controlling GBS resistance and ensuring effective treatment options.

Furthermore, the high heterogeneity (*I*^2^ values) observed across multiple antibiotics indicates substantial variability among the included studies, reflecting differences in study methodologies, geographic distribution, and temporal trends in GBS resistance. One key source of heterogeneity stems from variations in AST methods, including differences in breakpoints, testing protocols, and regional laboratory standards. Furthermore, microbiological diagnostic approaches may vary, with some studies relying solely on culture-based methods while others incorporate molecular techniques such as PCR for pathogen identification and resistance gene detection. Studies that combine culture with serotyping or culture with PCR-based detection may introduce further variability in resistance estimates. Additionally, geographic differences in antibiotic prescribing practices and selective pressure, along with temporal shifts in resistance trends, likely contribute to the observed heterogeneity. Addressing these methodological discrepancies through standardized protocols and region-specific analyses is crucial for improving the comparability and interpretability of resistance data in GBS research.

Subgroup analysis revealed geographical variations in resistance rates, with clindamycin resistance being the lowest in Iceland (1%) and highest in Nigeria (76.2%), which could be attributed to higher usage rates, over-the-counter availability, and lack of stringent antibiotic policies, emphasizing the importance of local surveillance and tailored antibiotic stewardship programs. Erythromycin resistance also varied, being the lowest in South Africa (1.4%), possibly reflecting stringent control measures and targeted antibiotic use. An unspecified region reported a resistance rate as high as 88.9%, indicating severe misuse or overuse of erythromycin and the necessity for immediate public health interventions and highest in the unspecified regions (88.9%).

Continental differences were noted, with Asia showing the highest resistance rates to several antibiotics, including erythromycin (43.4%) and clindamycin (37.7%), compared to lower rates in the Americas and Europe. This suggests that broader regional factors, such as healthcare infrastructure and antibiotic use policies, might affect resistance patterns and the need for enhanced antibiotic stewardship and surveillance programs to curb the rise of resistance.

The observed differences in resistance rates based on AST methods highlight the importance of methodological standardization. Using reliable and validated testing methods consistently ensures accurate resistance data, critical for effective clinical decision-making and public health strategies.

MIC-based methods reported an extremely high resistance rate to amikacin (99.3%) compared to disc methods (11.1%), highlighting the importance of standardized testing methods to ensure consistent and reliable data. These results show that MIC methods might be more sensitive for detecting resistant strains.

Similar variations were noted for other antibiotics, underscoring the need for standardized testing protocols to ensure consistent and reliable resistance data across different laboratories and studies.

Recent studies (2020–2023) have generally reported higher resistance rates for several antibiotics, including ceftriaxone (9.7%) and clindamycin (32.3%). This indicates a potentially increasing trend in the resistance.

Temporal trends indicate an increase in resistance rates for several antibiotics over recent years (2020–2023), including ceftriaxone (9.7%) and clindamycin (32.3%). This reflects a worrying upward trend in GBS resistance and underscores the need for ongoing surveillance and updated treatment guidelines.

The quality of the studies also affected the reported resistance rates. Studies categorized as “high risk” often reported higher resistance rates. For instance, tetracycline resistance was highest in low-risk studies (81.8%), suggesting that study quality can impact the reported resistance rates.

Variations in study quality underscore the need for high methodological standards in the research. High-quality studies provide reliable data for accurately assessing the burden of antibiotic resistance and informing effective interventions.

Given the observed antibiotic resistance trends in GBS, it is crucial to consider the clinical implications for treatment guidelines and healthcare policies. The emergence of MDR strains underscores the need for updated treatment protocols, particularly for high-risk populations such as neonates, pregnant women, and the elderly. Clinicians should be guided by more dynamic, region-specific susceptibility data rather than relying solely on historical resistance patterns. Additionally, antibiotic stewardship programs should be strengthened to reduce unnecessary antibiotic use and slow the progression of resistance. Policies should focus on regular surveillance and implementing rapid diagnostic tools, including PCR-based methods, to ensure timely and appropriate treatment. Developing new antibiotics and alternatives to current therapies should also be prioritized to address the growing challenge of multidrug resistance in GBS infections.

Despite the rigorous methodology employed in this study, several limitations should be acknowledged. One key limitation is the potential influence of publication bias, even though we assessed it using funnel plots and statistical tests. Studies with negative or inconclusive results are less likely to be published, which may lead to overestimating antibiotic resistance rates in GBS. This selective reporting bias could distort the true resistance landscape, as studies showing lower resistance rates or no significant trends might be underrepresented.

Additionally, missing or unpublished data may result in geographic disparities, particularly in regions with limited surveillance studies or where only certain resistance profiles are reported. The exclusion of smaller studies or those published in non-indexed journals further contributes to potential data gaps. Variability in study methodologies, including differences in AST protocols and diagnostic approaches (culture-based vs. PCR-based methods), may also introduce heterogeneity in the findings.

Furthermore, while we performed subgroup analyses based on study quality, the risk of bias in included studies may still affect the overall conclusions. Some studies lacked detailed reporting on key methodological aspects, such as sample selection criteria and control for confounding variables, which could influence resistance estimates. Future research should prioritize standardized surveillance methodologies and explore strategies such as gray literature searches, data sharing initiatives, and the inclusion of preprint studies to mitigate publication bias. Expanding resistance monitoring programs across diverse geographic regions and clinical settings will be essential to ensure a more comprehensive and globally representative understanding of GBS antibiotic resistance trends.

The variability of GBS resistance patterns across different continents and even within various regions of the same country is an essential limitation of this study. Several factors, including differences in local antimicrobial use, regional healthcare practices, and pathogen characteristics, may influence this variability. Our data represent a broad overview of global resistance patterns, but these patterns may not be universally applicable. We recommend that future studies explore regional variations in more detail, as this could provide valuable insights into the local drivers of resistance and support the development of region-specific treatment strategies.

## 5 Conclusion

In conclusion, this study highlighted the varying resistance patterns of GBS across different antibiotics, regions, and testing methods. The low resistance rates to penicillin, ampicillin, vancomycin, and linezolid suggest that these remain effective treatments for GBS infection. However, the high resistance rates to clindamycin, erythromycin, and tetracycline necessitate cautious use and consideration of local resistance patterns when choosing treatment options. This systematic review and meta-analysis, encompassing data from 334 studies across 57 countries, revealed significant geographical variations and trends, underscoring the need for localized antibiotic stewardship programs and continuous global surveillance to combat antibiotic resistance effectively. Increasing antibiotic resistance in GBS infections poses a significant challenge, particularly for neonates, pregnant women, and immunocompromised individuals. In recent years, the increasing trend in resistance rates has highlighted the urgent need for continuous surveillance, high-quality research, and adaptive treatment guidelines. Future research should focus on standardizing AST methods and improving study quality to ensure more reliable and comparable data across regions and periods, ultimately enhancing our ability to manage GBS infections and mitigate the impact of antibiotic resistance on public health.

## Data Availability

The original contributions presented in the study are included in the article/Supplementary material. Further inquiries can be directed to the corresponding authors.
