# Supplementary material for: Global patterns of antibiotic resistance in group B Streptococcus: a systematic review and meta-analysis
Source: Front Microbiol. 2025 Apr 16;16:1541524. doi: 10.3389/fmicb.2025.1541524 (PMC12060732; doi:10.3389/fmicb.2025.1541524)
Supplement: Supplementary file 1 [file Table_1.DOCX]

[1. The search syntax used for literature review in each online database. 3](#_Toc182816286)

[2. Comprehensive Antibiotic-Specific Meta-analysis Results 3](#_Toc182816287)

[Prevalence of ampicillin resistance 3](#_Toc182816288)

[Prevalence of ampicillin. sulbactam resistance 4](#_Toc182816289)

[Prevalence of cefazolin resistance 4](#_Toc182816290)

[Prevalence of clindamycin resistance 5](#_Toc182816291)

[Prevalence of erythromycin resistance 5](#_Toc182816292)

[Prevalence of vancomycin resistance 5](#_Toc182816293)

[Prevalence of ceftriaxone resistance 6](#_Toc182816294)

[Prevalence of amoxicillin resistance 6](#_Toc182816295)

[Prevalence of cefuroxime resistance 6](#_Toc182816296)

[Prevalence of cefotaxime resistance 7](#_Toc182816297)

[Prevalence of meropenem resistance 7](#_Toc182816298)

[Prevalence of imipenem resistance 8](#_Toc182816299)

[Prevalence of azithromycin resistance 8](#_Toc182816300)

[Prevalence of clarithromycin resistance 8](#_Toc182816301)

[Prevalence of erythrocin resistance 9](#_Toc182816302)

[Prevalence of tetracycline resistance 9](#_Toc182816303)

[Prevalence of doxycycline resistance 9](#_Toc182816304)

[Prevalence of trimethoprim.sulfamethoxazole resistance 10](#_Toc182816305)

[Prevalence of ciprofloxacin resistance 10](#_Toc182816306)

[Prevalence of levofloxacin resistance 11](#_Toc182816307)

[Prevalence of gentamicin resistance 11](#_Toc182816308)

[Prevalence of linezolid resistance 11](#_Toc182816309)

[Prevalence of daptomycin resistance 12](#_Toc182816310)

[Prevalence of tigecycline resistance 12](#_Toc182816311)

[Prevalence of nitrofurantoin resistance 12](#_Toc182816312)

[Prevalence of ceftaroline resistance 13](#_Toc182816313)

[Prevalence of tedizolid resistance 13](#_Toc182816314)

[Prevalence of cefepime resistance 13](#_Toc182816315)

[Prevalence of moxifloxacin resistance 14](#_Toc182816316)

[Prevalence of oxacillin resistance 14](#_Toc182816317)

[Prevalence of teicoplanin resistance 15](#_Toc182816318)

[Prevalence of quinupristin.dalfopristin resistance 15](#_Toc182816319)

[Prevalence of chloramphenicol resistance 15](#_Toc182816320)

[Prevalence of cefditoren resistance 16](#_Toc182816321)

[Prevalence of norfloxacin resistance 16](#_Toc182816322)

[Prevalence of amoxicillin.clavulanate resistance 16](#_Toc182816323)

[Prevalence of cefoxitin resistance 17](#_Toc182816324)

[Prevalence of norfloxacin resistance 17](#_Toc182816325)

[Prevalence of ofloxacin resistance 17](#_Toc182816326)

[Prevalence of amikacin resistance 18](#_Toc182816327)

[Prevalence of nalidixic acid resistance 18](#_Toc182816328)

[Supplementary Table 1: Characteristics and Extracted Data of Studies Included in the Meta-analysis 19](#_Toc182816329)

[Supplementary Table 2: Prevalence of Antibiotic Resistance 64](#_Toc182816330)

[References 97](#_Toc182816331)

# 1. The search syntax used for literature review in each online database.

**Web of Science query:**

(“Streptococcus agalactiae” OR “group B strep*” OR GBS OR “S. agalactiae”)

**PubMed** **query:**

(“Streptococcus agalactiae” [Title/Abstract] OR “group B strep*” [Title/Abstract] OR

GBS[Title/Abstract] OR “S. agalactiae” [Title/Abstract])

**Embase query:**

(“streptococcus agalactiae”:ab,ti OR “group b strep*”:ab,ti OR GBS:ab,ti OR “s)

agalactiae”: ab,ti

**Scopus query:**

(“Streptococcus agalactiae” OR “Group B Streptococcus” OR GBS)

# 2. Comprehensive Antibiotic-Specific Meta-analysis Results

## Prevalence of ampicillin resistance

A total of 15558 isolates investigated in 126 studies were included in the analysis of ampicillin resistance. The estimated average proportion based on the random-effects model was 0.031 (95%CI, 0.020, 0.046). Therefore, the average outcome differed significantly from zero (z=-15.720, p<0.001). According to the Q-test, the outcomes appear to be heterogeneous Q(125) = 1358.862, I²=90.80%, p<0.001. A forest plot showing the observed outcomes and the estimate based on the random-effects model is shown in Figure 2. With the fill and trim method implementation, the proportion changed to 0.065 (95%CI, 0.045, 0.092). Examining the student’s residuals revealed that none of the studies had a value larger than 3.542. Hence, there was no indication of outliers in the context of this model. According to Cook's distances, several studies (M. M. Majigo.1; M. A. Qadi; S. N. A. Alani; M. M. Majigo.2) could be considered overly influential. Post-removal of the potential outlier, the proportion equalled 0.065 (95%CI, 0.045, 0.092). The rank correlation and the regression test indicated potential funnel plot asymmetry (p<0.001 and p=0.007, respectively).

## Prevalence of ampicillin. sulbactam resistance

Two hundred twenty-nine isolates were investigated in 6 studies included in the ampicillin analysis. Sulbactam resistance. The estimated average proportion based on the random-effects model was 0.043 (95%CI, 0.012, 0.140). Therefore, the average outcome differed significantly from zero (z=-4.736, p<0.001). The Q-test for heterogeneity was insignificant, but some may still be present in the outcomes. A forest plot showing the observed outcomes and the estimate based on the random-effects model is shown in Figure 2. With the fill and trim method implementation, the proportion changed to 0.043 (95%CI, 0.012, 0.140). Examining the studentised residuals revealed that none of the studies had a value larger than 2.638. Hence, there was no indication of outliers in the context of this model. According to Cook's distances, one study (G. F. A.-S. Karim) could be considered overly influential. Neither the rank correlation nor the regression test indicated any funnel plot asymmetry (p=0.719 and p=0.588, respectively).

## Prevalence of cefazolin resistance

One thousand seven hundred forty-four isolates were investigated in 12 studies included in the cefazolin resistance analysis. The estimated average proportion based on the random-effects model was 0.013 (95%CI, 0.002, 0.079). Therefore, the average outcome differed significantly from zero (z=-4.548, p<0.001). According to the Q-test, the outcomes appear to be heterogeneous Q(11) = 156.809, I²=92.99%, p<0.001. A forest plot showing the observed outcomes and the estimate based on the random-effects model is shown in Figure 2. With the implementation of the fill and trim method, the proportion changed to 0.035 (95%CI, 0.008, 0.135). An examination of the studentized residuals revealed that one study (R. M. Mudzana) had a value larger than 2.865 and maybe a potential outlier in the context of this model. Upon exclusion of this potential outlier, the proportion was found to be 0.035 (95%CI, 0.008, 0.135). According to Cook's distances, none of the studies could be considered overly influential. Neither the rank correlation nor the regression test indicated any funnel plot asymmetry (p=0.060 and p=0.119, respectively).

## Prevalence of clindamycin resistance

A total of 51066 isolates investigated in 216 studies were included in the analysis of clindamycin resistance. The estimated average proportion based on the random-effects model was 0.293 (95%CI, 0.269, 0.319). Therefore, the average outcome differed significantly from zero (z=-14.125, p<0.001). According to the Q-test, the outcomes appear to be heterogeneous Q(215) = 5511.774, I²=96.10%, p<0.001. A forest plot showing the observed outcomes and the estimate based on the random-effects model is shown in Figure 2. With the fill and trim method implementation, the proportion changed to 0.337 (95%CI, 0.309, 0.365). An examination of the studentized residuals revealed that several studies (M. A. K. Shadbad; E. F. Lopes; R. O. A. Biobaku Oluwafunmilola; A. H. Alhhazmi) had values more significant than 3.682 and may be potential outliers in the context of this model. Upon exclusion of this potential outlier, the proportion was found to be 0.337 (95%CI, 0.309, 0.365). According to Cook's distances, two studies (R. O. A. Biobaku Oluwafunmilola and A. H. Alhhazmi) could be considered overly influential. Post-removal of the potential outlier, the proportion equaled 0.337 (95%CI, 0.309, 0.365). The regression test indicated funnel plot asymmetry (p=0.013) but not the rank correlation test (p=0.669).

## Prevalence of erythromycin resistance

A total of 47934 isolates investigated in 221 studies were included in the analysis of erythromycin resistance. The estimated average proportion based on the random-effects model was 0.350 (95%CI, 0.324, 0.378). Therefore, the average outcome differed significantly from zero (z=-10.059, p<0.001). According to the Q-test, the outcomes appear to be heterogeneous Q(220) = 5527.737, I²=96.02%, p<0.001. A forest plot showing the observed outcomes and the estimate based on the random-effects model is shown in Figure 2. With the fill and trim method implementation, the proportion changed to 0.390 (95%CI, 0.361, 0.420). An examination of the studentized residuals revealed that several studies (Z. J. Liu; H. K. Moroi; E. F. Lopes; A. H. Alhhazmi) had values more significant than 3.688 and may be potential outliers in the context of this model. Upon exclusion of this potential outlier, the proportion was found to be 0.390 (95%CI, 0.361, 0.420). According to Cook's distances, one study (A. H. Alhhazmi) could be considered overly influential. Neither the rank correlation nor the regression test indicated any funnel plot asymmetry (p=0.263 and p=0.067, respectively).

## Prevalence of vancomycin resistance

A total of 45009 isolates investigated in 186 studies were included in the analysis of vancomycin resistance. The estimated average proportion based on the random-effects model was 0.014 (95%CI, 0.010, 0.020). Therefore, the average outcome differed significantly from zero (z=-22.784, p<0.001). According to the Q-test, the outcomes appear to be heterogeneous Q(185) = 1647.329, I²=88.77%, p<0.001. A forest plot showing the observed outcomes and the estimate based on the random-effects model is shown in Figure 2. With the fill and trim method implementation, the proportion changed to 0.033 (95%CI, 0.024, 0.045). An examination of the studentized residuals revealed that one study (R. B. B. A. Schaub) had a value larger than 3.644 and may be a potential outlier in the context of this model. Upon exclusion of this potential outlier, the proportion was found to be 0.033 (95%CI, 0.024, 0.045).According to the Cook's distances, several studies (S. R. Abotorabi; M. A. Qadi; L. D. Mudzikati; N. B. Gharabeigi; R. B. B. A. Schuab; A. M. J. Ebrahim; L. A. K. Al Abbas; N. A. E. R. Fahim; L. R. S. Burcham; C. L. Hays; R. M. Mudzana; M. C. B. J. Perim) could be considered to be overly influential. Post-removal of the potential outlier, the proportion equalled 0.033 (95%CI, 0.024, 0.045). The rank correlation and the regression test indicated potential funnel plot asymmetry (p<0.001 and p=0.041, respectively).

## Prevalence of ceftriaxone resistance

A total of 30196 isolates investigated in 78 studies were included in the analysis of ceftriaxone resistance. The estimated average proportion based on the random-effects model was 0.062 (95%CI, 0.039, 0.097). Therefore, the average outcome differed significantly from zero (z=-10.890, p<0.001). According to the Q-test, the outcomes appear to be heterogeneous Q(77) = 923.210, I²=91.66%, p<0.001. A forest plot showing the observed outcomes and the estimate based on the random-effects model is shown in Figure 2. With the implementation of the fill and trim method, the proportion changed to 0.136 (95%CI, 0.090, 0.200). An examination of the studentized residuals revealed that one study (R. B. B. A. Schaub) had a value larger than 3.414 and may be a potential outlier in the context of this model. Upon exclusion of this potential outlier, the proportion was found to be 0.136 (95%CI, 0.090, 0.200). According to Cook's distances, none of the studies could be considered overly influential. The regression test indicated funnel plot asymmetry (p<0.001) but not the rank correlation test (p=0.118).

## Prevalence of amoxicillin resistance

A total of 9837 isolates investigated in 10 studies were included in the analysis of amoxicillin resistance. The estimated average proportion based on the random-effects model was 0.035 (95%CI, 0.006, 0.178). Therefore, the average outcome differed significantly from zero (z=-3.640, p<0.001). According to the Q-test, the outcomes appear to be heterogeneous Q(9) = 109.563, I²=91.79%, p<0.001. A forest plot showing the observed outcomes and the estimate based on the random-effects model is shown in Figure 2. With the fill and trim method implementation, the proportion changed to 0.035 (95%CI, 0.006, 0.178). Examining the studentized residuals revealed that none of the studies had a value larger than 2.807. Hence, there was no indication of outliers in the context of this model. According to Cook's distances, none of the studies could be considered overly influential. The regression test indicated funnel plot asymmetry (p<0.001) but not the rank correlation test (p=0.156).

## Prevalence of cefuroxime resistance

A total of 4806 isolates investigated in 17 studies were included in the analysis of cefuroxime resistance. The estimated average proportion based on the random-effects model was 0.030 (95%CI, 0.012, 0.070). Therefore, the average outcome differed significantly from zero (z=-7.633, p<0.001). According to the Q-test, the outcomes appear to be heterogeneous Q(16) = 92.618, I²=82.72%, p<0.001. A forest plot showing the observed outcomes and the estimate based on the random-effects model is shown in Figure 2. With the implementation of the fill and trim method, the proportion changed to 0.107 (95%CI, 0.047, 0.225). Examining the studentized residuals revealed that none of the studies had a value larger than 2.974. Hence, there was no indication of outliers in the context of this model. According to Cook's distances, none of the studies could be considered overly influential. The regression test indicated funnel plot asymmetry (p<0.001) but not the rank correlation test (p=0.490).

## Prevalence of cefotaxime resistance

A total of 6595 isolates investigated in 67 studies were included in the analysis of cefotaxime resistance. The estimated average proportion based on the random-effects model was 0.032 (95%CI, 0.017, 0.060). Therefore, the average outcome differed significantly from zero (z=-10.130, p<0.001). According to the Q-test, the outcomes appear to be heterogeneous Q(66) = 814.671, I²=91.90%, p<0.001. A forest plot showing the observed outcomes and the estimate based on the random-effects model is shown in Figure 2. With the implementation of the fill and trim method, the proportion changed to 0.065 (95%CI, 0.037, 0.110). Examining the studentized residuals revealed that none of the studies had a value larger than 3.372. Hence, there was no indication of outliers in the context of this model. According to Cook's distances, several studies (S. R. Abotorabi; N. K. Nagano; S. N. A. Alani) could be considered overly influential. Post-removal of the potential outlier, the proportion equalled 0.065 (95%CI, 0.037, 0.110). The rank correlation test indicated funnel plot asymmetry (p=0.001) but not the regression test (p=0.375).

## Prevalence of meropenem resistance

A total of 26329 isolates investigated in 27 studies were included in the analysis of meropenem resistance. The estimated average proportion based on the random-effects model was 0.007 (95%CI, 0.003, 0.017). Therefore, the average outcome differed significantly from zero (z=-11.093, p<0.001). According to the Q-test, the outcomes appear to be heterogeneous Q(26) = 74.104, I²=64.91%, p<0.001. A forest plot showing the observed outcomes and the estimate based on the random-effects model is shown in Figure 2. With the implementation of the fill and trim method, the proportion changed to 0.020 (95%CI, 0.008, 0.048). An examination of the studentized residuals revealed that one study (S. N. A. Alani) had a value larger than 3.113 and maybe a potential outlier in the context of this model. Upon exclusion of this potential outlier, the proportion was found to be 0.020 (95%CI, 0.008, 0.048). According to Cook's distances, two studies (Y. W. Gomi and S. N. A. Alani) could be considered overly influential. Post-removal of the potential outlier, the proportion equalled 0.020 (95%CI, 0.008, 0.048). The rank correlation test indicated funnel plot asymmetry (p<0.001) but not the regression test (p=0.569).

## Prevalence of imipenem resistance

A total of 384 isolates investigated in 12 studies were included in the analysis of imipenem resistance. The estimated average proportion based on the random-effects model was 0.065 (95%CI, 0.023, 0.166). Therefore, the average outcome differed significantly from zero (z=-4.939, p<0.001). According to the Q-test, the outcomes appear to be heterogeneous Q(11) = 36.020, I²=69.46%, p<0.001. A forest plot showing the observed outcomes and the estimate based on the random-effects model is shown in Figure 2. With the implementation of the fill and trim method, the proportion changed to 0.134 (95%CI, 0.055, 0.293). An examination of the studentized residuals revealed that one study (G. F. A.-S. Karim) had a value larger than 2.865 and maybe a potential outlier in the context of this model. Upon exclusion of this potential outlier, the proportion was found to be 0.134 (95%CI, 0.055, 0.293). According to Cook's distances, none of the studies could be considered overly influential. The rank correlation and the regression test indicated potential funnel plot asymmetry (p=0.042 and p<0.001, respectively).

## Prevalence of azithromycin resistance

Twenty-one thousand three hundred thirty-four isolates investigated in 21 studies were included in analysing azithromycin resistance. The estimated average proportion based on the random-effects model was 0.410 (95%CI, 0.280, 0.554). Therefore, the average outcome was not significantly different from zero (z=-1.233, p=0.218). According to the Q-test, the outcomes appear to be heterogeneous Q(20) = 737.262, I²=97.29%, p<0.001. A forest plot showing the observed outcomes and the estimate based on the random-effects model is shown in Figure 2. With the fill and trim method implementation, the proportion changed to 0.410 (95%CI, 0.280, 0.554). An examination of the studentized residuals revealed that none of the studies had a value larger than 3.038. Hence, there was no indication of outliers in the context of this model. According to Cook's distances, none of the studies could be considered overly influential. The regression test indicated funnel plot asymmetry (p=0.047) but not the rank correlation test (p=0.242).

## Prevalence of clarithromycin resistance

A total of 1468 isolates investigated in 12 studies were included in the analysis of clarithromycin resistance. The estimated average proportion based on the random-effects model was 0.434 (95%CI, 0.303, 0.575). Therefore, the average outcome was not significantly different from zero (z=-0.920, p=0.357). According to the Q-test, the outcomes appear to be heterogeneous Q(11) = 186.907, I²=94.11%, p<0.001. A forest plot showing the observed outcomes and the estimate based on the random-effects model is shown in Figure 2. With the fill and trim method implementation, the proportion changed to 0.434 (95%CI, 0.303, 0.575). An examination of the studentized residuals revealed that one study (P. T. Wang) had a value larger than 2.865 and maybe a potential outlier in the context of this model. Upon exclusion of this potential outlier, the proportion was found to be 0.434 (95%CI, 0.303, 0.575). According to Cook's distances, none of the studies could be considered overly influential. Neither the rank correlation nor the regression test indicated any funnel plot asymmetry (p=0.648 and p=0.303, respectively).

## Prevalence of erythrocin resistance

A total of 554 isolates investigated in 7 studies were included in the analysis of erythrocin resistance. The estimated average proportion based on the random-effects model was 0.597 (95%CI, 0.310, 0.829). Therefore, the average outcome was not significantly different from zero (z=0.645, p=0.519). According to the Q-test, the outcomes appear to be heterogeneous Q(6) = 160.723, I²=96.27%, p<0.001. A forest plot showing the observed outcomes and the estimate based on the random-effects model is shown in Figure 2. With the fill and trim method implementation, the proportion changed to 0.597 (95%CI, 0.310, 0.829). Examining the studentized residuals revealed that none of the studies had a value larger than 2.690. Hence, there was no indication of outliers in the context of this model. According to Cook's distances, none of the studies could be considered overly influential. Neither the rank correlation nor the regression test indicated any funnel plot asymmetry (p>0.999 and p=0.326, respectively).

## Prevalence of tetracycline resistance

A total of 28322 isolates investigated in 124 studies were included in the analysis of tetracycline resistance. The estimated average proportion based on the random-effects model was 0.801 (95%CI, 0.771, 0.828). Therefore, the average outcome differed significantly from zero (z=15.311, p<0.001). According to the Q-test, the outcomes appear to be heterogeneous Q(123) = 2944.944, I²=95.82%, p<0.001. A forest plot showing the observed outcomes and the estimate based on the random-effects model is shown in Figure 2. With the fill and trim method implementation, the proportion changed to 0.753 (95%CI, 0.719, 0.784). An examination of the studentized residuals revealed that two studies (R. I. Creti and R. N. Morfin-Otero) had values more significant than 3.538 and may be potential outliers in the context of this model. Upon exclusion of this potential outlier, the proportion was found to be 0.753 (95%CI, 0.719, 0.784). According to Cook's distances, several studies (C. T. Matani; R. I. Creti; R. N. Morfin-Otero) could be considered overly influential. Post-removal of the potential outlier, the proportion equalled 0.753 (95%CI, 0.719, 0.784). Neither the rank correlation nor the regression test indicated any funnel plot asymmetry (p=0.808 and p=0.648, respectively).

## Prevalence of doxycycline resistance

A total of 372 isolates investigated in 5 studies were included in the analysis of doxycycline resistance. The estimated average proportion based on the random-effects model was 0.649 (95%CI, 0.371, 0.853). Therefore, the average outcome was not significantly different from zero (z=1.052, p=0.293). According to the Q-test, the outcomes appear to be heterogeneous Q(4) = 63.083, I²=93.66%, p<0.001. A forest plot showing the observed outcomes and the estimate based on the random-effects model is shown in Figure 2. With the fill and trim method implementation, the proportion changed to 0.649 (95%CI, 0.371, 0.853). Examining the studentized residuals revealed that none of the studies had a value larger than 2.576. Hence, there was no indication of outliers in the context of this model. According to Cook's distances, none of the studies could be considered overly influential. The regression test indicated funnel plot asymmetry (p<0.001) but not the rank correlation test (p=0.069).

## Prevalence of trimethoprim.sulfamethoxazole resistance

A total of 5705 isolates investigated in 30 studies were included in the trimethoprim-sulfamethoxazole resistance analysis. The estimated average proportion based on the random-effects model was 0.213 (95%CI, 0.107, 0.378). Therefore, the average outcome differed significantly from zero (z=-3.162, p=0.002). According to the Q-test, the outcomes appear to be heterogeneous Q(29) = 458.185, I²=93.67%, p<0.001. A forest plot showing the observed outcomes and the estimate based on the random-effects model is shown in Figure 2. With the implementation of the fill and trim method, the proportion changed to 0.277 (95%CI, 0.146, 0.463). An examination of the studentized residuals revealed that one study (M. T. Ábrók) had a value larger than 3.144 and maybe a potential outlier in the context of this model. Upon exclusion of this potential outlier, the proportion was found to be 0.277 (95%CI, 0.146, 0.463). According to Cook's distances, none of the studies could be considered overly influential. The regression test indicated funnel plot asymmetry (p=0.011) but not the rank correlation test (p=0.539).

## Prevalence of ciprofloxacin resistance

A total of 3558 isolates investigated in 43 studies were included in the analysis of ciprofloxacin resistance. The estimated average proportion based on the random-effects model was 0.179 (95%CI, 0.127, 0.246). Therefore, the average outcome differed significantly from zero (z=-7.391, p<0.001). According to the Q-test, the outcomes appear to be heterogeneous Q(42) = 438.622, I²=90.42%, p<0.001. A forest plot showing the observed outcomes and the estimate based on the random-effects model is shown in Figure 2. With the implementation of the fill and trim method, the proportion changed to 0.179 (95%CI, 0.127, 0.246). An examination of the studentized residuals revealed that one study (M. M. Doumith) had a value larger than 3.248 and maybe a potential outlier in the context of this model. Upon exclusion of this potential outlier, the proportion was found to be 0.179 (95%CI, 0.127, 0.246). According to Cook's distances, one study (M. M. Doumith) could be considered overly influential. The regression test indicated funnel plot asymmetry (p=0.008) but not the rank correlation test (p=0.213).

## Prevalence of levofloxacin resistance

A total of 46465 isolates investigated in 125 studies were included in the analysis of levofloxacin resistance. The estimated average proportion based on the random-effects model was 0.086 (95%CI, 0.068, 0.108). Therefore, the average outcome differed significantly from zero (z=-18.020, p<0.001). According to the Q-test, the outcomes appear to be heterogeneous Q(124) = 3373.187, I²=96.32%, p<0.001. A forest plot showing the observed outcomes and the estimate based on the random-effects model is shown in Figure 2. With the implementation of the fill and trim method, the proportion changed to 0.141 (95%CI, 0.113, 0.176). An examination of the studentized residuals revealed that one study (R. B. B. A. Schaub) had a value larger than 3.540 and may be a potential outlier in the context of this model. Upon exclusion of this potential outlier, the proportion was found to be 0.141 (95%CI, 0.113, 0.176). According to Cook's distances, several studies (Y. W. Gomi; R. B. B. A. Schuab; C. L. Hays) could be considered overly influential. Post-removal of the potential outlier, the proportion equalled 0.141 (95%CI, 0.113, 0.176). The regression test indicated funnel plot asymmetry (p=0.001) but not the rank correlation test (p=0.082).

## Prevalence of gentamicin resistance

A total of 12155 isolates investigated in 32 studies were included in the analysis of gentamicin resistance. The estimated average proportion based on the random-effects model was 0.190 (95%CI, 0.080, 0.389). Therefore, the average outcome differed significantly from zero (z=-2.846, p=0.004). According to the Q-test, the outcomes appear to be heterogeneous Q(31) = 1143.547, I²=97.29%, p<0.001. A forest plot showing the observed outcomes and the estimate based on the random-effects model is shown in Figure 2. With the fill and trim method implementation, the proportion changed to 0.190 (95%CI, 0.080, 0.389). Examining the studentized residuals revealed that none of the studies had a value larger than 3.163. Hence, there was no indication of outliers in the context of this model. According to Cook's distances, none of the studies could be considered overly influential. Neither the rank correlation nor the regression test indicated any funnel plot asymmetry (p=0.570 and p=0.361, respectively).

## Prevalence of linezolid resistance

A total of 18117 isolates investigated in 90 studies were included in the analysis of linezolid resistance. The estimated average proportion based on the random-effects model was 0.008 (95%CI, 0.006, 0.011). Therefore, the average outcome differed significantly from zero (z=-30.956, p<0.001). According to the Q-test, the outcomes appear to be heterogeneous Q(89) = 112.539, I²=20.92%, p=0.047. A forest plot showing the observed outcomes and the estimate based on the random-effects model is shown in Figure 2. With the fill and trim method implementation, the proportion changed to 0.018 (95%CI, 0.013, 0.025). An examination of the studentized residuals revealed that one study (S. Ahmad) had a value larger than 3.452 and maybe a potential outlier in the context of this model. Upon exclusion of this potential outlier, the proportion was found to be 0.018 (95%CI, 0.013, 0.025). According to Cook's distances, several studies (R. E. F. Mendes.1; R. E. F. Mendes.2; C. G. S. Carvalhaes.1; C. G. S. Carvalhaes.2) could be considered overly influential. Post-removal of the potential outlier, the proportion equalled 0.018 (95%CI, 0.013, 0.025). The rank correlation test indicated funnel plot asymmetry (p<0.001) but not the regression test (p=0.816).

## Prevalence of daptomycin resistance

A total of 10690 isolates investigated in 23 studies were included in the analysis of daptomycin resistance. The estimated average proportion based on the random-effects model was 0.003 (95%CI, 0.002, 0.007). Therefore, the average outcome differed significantly from zero (z=-15.639, p<0.001). According to the Q-test, the outcomes appear to be heterogeneous Q(22) = 38.308, I²=42.57%, p=0.017. A forest plot showing the observed outcomes and the estimate based on the random-effects model is shown in Figure 2. With the implementation of the fill and trim method, the proportion changed to 0.011 (95%CI, 0.005, 0.025). An examination of the studentized residuals revealed that one study (M. M. W. Ali) had a value larger than 3.065 and maybe a potential outlier in the context of this model. Upon exclusion of this potential outlier, the proportion was found to be 0.011 (95%CI, 0.005, 0.025). According to Cook's distances, one study (M. M. W. Ali) could be considered overly influential. The rank correlation test indicated funnel plot asymmetry (p<0.001) but not the regression test (p=0.334).

## Prevalence of tigecycline resistance

A total of 3066 isolates investigated in 30 studies were included in the analysis of tigecycline resistance. The estimated average proportion based on the random-effects model was 0.007 (95%CI, 0.004, 0.012). Therefore, the average outcome differed significantly from zero (z=-19.093, p<0.001). According to the Q-test, the actual outcomes had no significant heterogeneity. A forest plot showing the observed outcomes and the estimate based on the random-effects model is shown in Figure 2. With the implementation of the fill and trim method, the proportion changed to 0.007 (95%CI, 0.004, 0.012). Examining the studentised residuals revealed that none of the studies had a value larger than 3.144. Hence, there was no indication of outliers in the context of this model. According to Cook's distances, one study (X. D. Li) could be overly influential. The rank correlation and the regression test indicated potential funnel plot asymmetry (p<0.001 and p=0.003, respectively).

## Prevalence of nitrofurantoin resistance

A total of 627 isolates investigated in 12 studies were included in the analysis of nitrofurantoin resistance. The estimated average proportion based on the random-effects model was 0.124 (95%CI, 0.055, 0.258). Therefore, the average outcome differed significantly from zero (z=-4.271, p<0.001). According to the Q-test, the outcomes appear to be heterogeneous Q(11) = 66.752, I²=83.52%, p<0.001. A forest plot showing the observed outcomes and the estimate based on the random-effects model is shown in Figure 2. With the fill and trim method implementation, the proportion changed to 0.150 (95%CI, 0.068, 0.300). Examining the studentized residuals revealed that none of the studies had a value larger than 2.865. Hence, there was no indication of outliers in the context of this model. According to Cook's distances, none of the studies could be considered overly influential. The regression test indicated funnel plot asymmetry (p=0.006) but not the rank correlation test (p=0.737).

## Prevalence of ceftaroline resistance

A total of 176 isolates investigated in 4 studies were included in the analysis of ceftaroline resistance. The estimated average proportion based on the random-effects model was 0.012 (95%CI, 0.003, 0.048). Therefore, the average outcome differed significantly from zero (z=-6.152, p<0.001). According to the Q-test, the actual outcomes had no significant heterogeneity. A forest plot showing the observed outcomes and the estimate based on the random-effects model is shown in Figure 2. With the implementation of the fill and trim method, the proportion changed to 0.012 (95%CI, 0.003, 0.048). An examination of the studentised residuals revealed that the studies had a value of 2.498; hence, there was no indication of outliers in the context of this model. According to Cook's distances, none of the studies could be considered overly influential. Neither the rank correlation nor the regression test indicated any funnel plot asymmetry (p=0.083 and p=0.518, respectively).

## Prevalence of tedizolid resistance

A total of 5213 isolates were investigated in 4 studies included in the tedizolid resistance analysis. The estimated average proportion based on the random-effects model was 0.001 (95%CI, 0.000, 0.008). Therefore, the average outcome differed significantly from zero (z=-6.850, p<0.001). According to the Q-test, the actual outcomes had no significant heterogeneity. A forest plot showing the observed outcomes and the estimate based on the random-effects model is shown in Figure 2. With the fill and trim method implementation, the proportion changed to 0.001 (95%CI, 0.000, 0.008). Examining the studentized residuals revealed that none of the studies had a value larger than 2.498. Hence, there was no indication of outliers in the context of this model. According to Cook's distances, none of the studies could be considered overly influential. The regression test indicated funnel plot asymmetry (p=0.024) but not the rank correlation test (p=0.056).

## Prevalence of cefepime resistance

A total of 5231 isolates investigated in 26 studies were included in the analysis of cefepime resistance. The estimated average proportion based on the random-effects model was 0.063 (95%CI, 0.027, 0.142). Therefore, the average outcome differed significantly from zero (z=-5.874, p<0.001). According to the Q-test, the outcomes appear to be heterogeneous Q(25) = 258.276, I²=90.32%, p<0.001. A forest plot showing the observed outcomes and the estimate based on the random-effects model is shown in Figure 2. With the fill and trim method implementation, the proportion changed to 0.118 (95%CI, 0.053, 0.242). An examination of the studentized residuals revealed that one study (N. K. Nagano) had a value larger than 3.102 and maybe a potential outlier in the context of this model. Upon exclusion of this potential outlier, the proportion was found to be 0.118 (95%CI, 0.053, 0.242). According to Cook's distances, none of the studies could be considered overly influential. The regression test indicated funnel plot asymmetry (p<0.001) but not the rank correlation test (p=0.559).

## Prevalence of moxifloxacin resistance

A total of 1431 isolates investigated in 20 studies were included in the analysis of moxifloxacin resistance. The estimated average proportion based on the random-effects model was 0.063 (95%CI, 0.033, 0.120). Therefore, the average outcome differed significantly from zero (z=-7.529, p<0.001). According to the Q-test, the outcomes appear to be heterogeneous Q(19) = 136.665, I²=86.10%, p<0.001. A forest plot showing the observed outcomes and the estimate based on the random-effects model is shown in Figure 2. With the fill and trim method implementation, the proportion changed to 0.115 (95%CI, 0.063, 0.200). An examination of the studentized residuals revealed that none of the studies had a value larger than 3.023. Hence, there was no indication of outliers in the context of this model. According to Cook's distances, none of the studies could be considered overly influential. The regression test indicated funnel plot asymmetry (p<0.001) but not the rank correlation test (p=0.537).

## Prevalence of oxacillin resistance

A total of 1069 isolates investigated in 9 studies were included in the analysis of oxacillin resistance. The estimated average proportion based on the random-effects model was 0.062 (95%CI, 0.012, 0.261). Therefore, the average outcome differed significantly from zero (z=-3.176, p=0.001). According to the Q-test, the outcomes appear to be heterogeneous Q(8) = 55.611, I²=85.61%, p<0.001. A forest plot showing the observed outcomes and the estimate based on the random-effects model is shown in Figure 2. With the fill and trim method implementation, the proportion changed to 0.062 (95%CI, 0.012, 0.261). Examining the studentized residuals revealed that none of the studies had a value larger than 2.773. Hence, there was no indication of outliers in the context of this model. According to Cook's distances, none of the studies could be considered overly influential. The rank correlation test indicated funnel plot asymmetry (p=0.046) but not the regression test (p=0.841).

## Prevalence of teicoplanin resistance

One thousand two hundred seventy-four isolates were investigated in 9 studies included in the teicoplanin resistance analysis. The estimated average proportion based on the random-effects model was 0.007 (95%CI, 0.003, 0.021). Therefore, the average outcome differed significantly from zero (z=-9.229, p<0.001). According to the Q-test, the actual outcomes had no significant heterogeneity. A forest plot showing the observed outcomes and the estimate based on the random-effects model is shown in Figure 2. With the implementation of the fill and trim method, the proportion changed to 0.016 (95%CI, 0.006, 0.039). An examination of the studentized residuals revealed that one study (C. T. Matani) had a value larger than 2.773 and maybe a potential outlier in the context of this model. Upon exclusion of this potential outlier, the proportion was found to be 0.016 (95%CI, 0.006, 0.039).According to Cook's distances, one study (C. T. Matani) could be considered overly influential. The rank correlation test indicated funnel plot asymmetry (p<0.001) but not the regression test (p=0.095).

## Prevalence of quinupristin.dalfopristin resistance

A total of 1260 isolates were investigated in 16 studies included in the quinupristin analysis—Dalfopristin resistance. The estimated average proportion based on the random-effects model was 0.014 (95%CI, 0.003, 0.065). Therefore, the average outcome differed significantly from zero (z=-5.200, p<0.001). According to the Q-test, the outcomes appear to be heterogeneous Q(15) = 163.262, I²=90.81%, p<0.001. A forest plot showing the observed outcomes and the estimate based on the random-effects model is shown in Figure 2. With the fill and trim method implementation, the proportion changed to 0.025 (95%CI, 0.007, 0.083). An examination of the studentized residuals revealed that one study (M. K. Zakerifar) had a value larger than 2.955 and maybe a potential outlier in the context of this model. Upon exclusion of this potential outlier, the proportion was found to be 0.025 (95%CI, 0.007, 0.083).According to Cook's distances, none of the studies could be considered overly influential. The rank correlation and the regression test indicated potential funnel plot asymmetry (p<0.001 and p=0.002, respectively).

## Prevalence of chloramphenicol resistance

A total of 10245 isolates investigated in 57 studies were included in the analysis of chloramphenicol resistance. The estimated average proportion based on the random-effects model was 0.072 (95%CI, 0.048, 0.107). Therefore, the average outcome differed significantly from zero (z=-11.536, p<0.001). According to the Q-test, the outcomes appear to be heterogeneous Q(56) = 830.146, I²=93.25%, p<0.001. A forest plot showing the observed outcomes and the estimate based on the random-effects model is shown in Figure 2. With the implementation of the fill and trim method, the proportion changed to 0.121 (95%CI, 0.083, 0.175). Examining the studentised residuals revealed that none of the studies had a value larger than 3.327. Hence, there was no indication of outliers in the context of this model. According to Cook's distances, none of the studies could be considered overly influential. The regression test indicated funnel plot asymmetry (p<0.001) but not the rank correlation test (p=0.203).

## Prevalence of cefditoren resistance

A total of 20636 isolates were investigated in 4 studies and included in the cefditoren resistance analysis. The estimated average proportion based on the random-effects model was 0.003 (95%CI, 0.000, 0.238). Therefore, the average outcome differed significantly from zero (z=-2.456, p=0.014). According to the Q-test, the outcomes appear to be heterogeneous Q(3) = 62.939, I²=95.23%, p<0.001. A forest plot showing the observed outcomes and the estimate based on the random-effects model is shown in Figure 2. With the fill and trim method implementation, the proportion changed to 0.015 (95%CI, 0.000, 0.410). Examining the studentized residuals revealed that none of the studies had a value larger than 2.498. Hence, there was no indication of outliers in the context of this model. According to Cook's distances, none of the studies could be considered overly influential. The regression test indicated funnel plot asymmetry (p=0.048) but not the rank correlation test (p>0.999).

## Prevalence of norfloxacin resistance

A total of 865 isolates that were investigated in 9 studies were included in the analysis of norfloxacin resistance. The estimated average proportion based on the random-effects model was 0.157 (95%CI, 0.084, 0.274). Therefore, the average outcome differed significantly from zero (z=-4.657, p<0.001). According to the Q-test, the outcomes appear to be heterogeneous Q(8) = 66.612, I²=87.99%, p<0.001. A forest plot showing the observed outcomes and the estimate based on the random-effects model is shown in Figure 2. With the fill and trim method implementation, the proportion changed to 0.226 (95%CI, 0.126, 0.372). Examining the studentized residuals revealed that none of the studies had a value larger than 2.773. Hence, there was no indication of outliers in the context of this model. According to Cook's distances, none of the studies could be considered overly influential. The regression test indicated funnel plot asymmetry (p<0.001) but not the rank correlation test (p=0.477).

## Prevalence of amoxicillin.clavulanate resistance

The 2033 isolates investigated in 7 studies were included in the amoxicillin-clavulanate resistance analysis. The estimated average proportion based on the random-effects model was 0.196 (95%CI, 0.023, 0.713). Therefore, the average outcome was not significantly different from zero (z=-1.190, p=0.234). According to the Q-test, the outcomes appear to be heterogeneous Q(6) = 50.331, I²=88.08%, p<0.001. A forest plot showing the observed outcomes and the estimate based on the random-effects model is shown in Figure 2. With the implementation of the fill and trim method, the proportion changed to 0.196 (95%CI, 0.023, 0.713). An examination of the studentized residuals revealed that one study (X. H. Vuillemin) had a value larger than 2.690 and maybe a potential outlier in the context of this model. Upon exclusion of this potential outlier, the proportion was found to be 0.196 (95%CI, 0.023, 0.713).According to Cook's distances, none of the studies could be considered overly influential. Neither the rank correlation nor the regression test indicated any funnel plot asymmetry (p>0.999 and p=0.972, respectively).

## Prevalence of cefoxitin resistance

A total of 184 isolates were investigated in 4 studies included in the cefoxitin resistance analysis. The estimated average proportion based on the random-effects model was 0.186 (95%CI, 0.031, 0.622). Therefore, the average outcome was not significantly different from zero (z=-1.467, p=0.142). According to the Q-test, the outcomes appear to be heterogeneous Q(3) = 16.289, I²=81.58%, p<0.001. A forest plot showing the observed outcomes and the estimate based on the random-effects model is shown in Figure 2. With the fill and trim method implementation, the proportion changed to 0.186 (95%CI, 0.031, 0.622). Examining the studentized residuals revealed that none of the studies had a value larger than 2.498. Hence, there was no indication of outliers in the context of this model. According to Cook's distances, none of the studies could be considered overly influential. Neither the rank correlation nor the regression test indicated any funnel plot asymmetry (p>0.999 and p=0.995, respectively).

## Prevalence of norfloxacin resistance

Four hundred ninety-nine isolates investigated in 4 studies were included in the analysis of norfloxacin resistance. The estimated average proportion based on the random-effects model was 0.096 (95%CI, 0.006, 0.648). Therefore, the average outcome was not significantly different from zero (z=-1.542, p=0.123). According to the Q-test, the outcomes appear to be heterogeneous Q(3) = 34.953, I²=91.42%, p<0.001. A forest plot showing the observed outcomes and the estimate based on the random-effects model is shown in Figure 2. With the implementation of the fill and trim method, the proportion changed to 0.096 (95%CI, 0.006, 0.648). Examining the studentized residuals revealed that none of the studies had a value larger than 2.498. Hence, there was no indication of outliers in the context of this model. According to Cook's distances, none of the studies could be considered overly influential. The regression test indicated funnel plot asymmetry (p<0.001) but not the rank correlation test (p=0.333).

## Prevalence of ofloxacin resistance

A total of 292 isolates investigated in 6 studies were included in the analysis of ofloxacin resistance. The estimated average proportion based on the random-effects model was 0.273 (95%CI, 0.049, 0.731). Therefore, the average outcome was not significantly different from zero (z=-0.970, p=0.332). According to the Q-test, the outcomes appear to be heterogeneous Q(5) = 92.807, I²=94.61%, p<0.001. A forest plot showing the observed outcomes and the estimate based on the random-effects model is shown in Figure 2. With the fill and trim method implementation, the proportion changed to 0.273 (95%CI, 0.049, 0.731). Examining the studentized residuals revealed that none of the studies had a value larger than 2.638. Hence, there was no indication of outliers in the context of this model. According to Cook's distances, none of the studies could be considered overly influential. Neither the rank correlation nor the regression test indicated any funnel plot asymmetry (p>0.999 and p=0.747, respectively).

## Prevalence of amikacin resistance

A total of 9033 isolates investigated in 10 studies were included in the analysis of amikacin resistance. The estimated average proportion based on the random-effects model was 0.196 (95%CI, 0.076, 0.422). Therefore, the average outcome differed significantly from zero (z=-2.524, p=0.012). According to the Q-test, the outcomes appear to be heterogeneous Q(9) = 58.676, I²=84.66%, p<0.001. A forest plot showing the observed outcomes and the estimate based on the random-effects model is shown in Figure 2. With the implementation of the fill and trim method, the proportion changed to 0.196 (95%CI, 0.076, 0.422). An examination of the studentized residuals revealed that one study (M. H. Malek-Jafarian) had a value larger than 2.807 and maybe a potential outlier in the context of this model. Upon exclusion of this potential outlier, the proportion was found to be 0.196 (95%CI, 0.076, 0.422).According to Cook's distances, one study (M. H. Malek-Jafarian) could be considered overly influential. Neither the rank correlation nor the regression test indicated any funnel plot asymmetry (p=0.612 and p=0.748, respectively).

## Prevalence of nalidixic acid resistance

A total of 135 isolates investigated in 4 studies were included in the analysis of nalidixic acid resistance. The estimated average proportion based on the random-effects model was 0.749 (95%CI, 0.420, 0.925). Therefore, the average outcome was not significantly different from zero (z=1.515, p=0.130). According to the Q-test, the outcomes appear to be heterogeneous Q(3) = 13.287, I²=77.42%, p=0.004. A forest plot showing the observed outcomes and the estimate based on the random-effects model is shown in Figure 2. With the fill and trim method implementation, the proportion changed to 0.749 (95%CI, 0.420, 0.925). An examination of the studentized residuals revealed that one study (M. H. Malek-Jafarian) had a value larger than 2.498 and maybe a potential outlier in the context of this model. Upon exclusion of this potential outlier, the proportion was found to be 0.749 (95%CI, 0.420, 0.925). According to Cook's distances, none of the studies could be considered overly influential. Neither the rank correlation nor the regression test indicated any funnel plot asymmetry (p=0.333 and p=0.180, respectively).

# Supplementary Table 1: Characteristics and Extracted Data of Studies Included in the Meta-analysis

| Author | year | countries | AST category | Quality group | penicillin | ampicillin | SAM | cefazolin | clindamycin | erythromycin | vancomycin | ceftriaxone | amoxicillin | cefuroxime | cefotaxime | meropenem | imipenem | azithromycin | clarithromycin | erythrocin | tetracycline | doxycycline | TMP/SMX | ciprofloxacin | levofloxacin | gentamicin | linezolid | daptomycin | tigecycline | nitrofurantoin | ceftaroline | tedizolid | cefepime | moxifloxacin | oxacillin | teicoplanin | Q/D | chloramphenicol | cefditoren | norfloxacins | AMC | cefoxitine | norfloxacin | ofloxacin | amikacin | nalidixicacid |
| --- | --- | --- | --- | --- | --- | --- | --- | --- | --- | --- | --- | --- | --- | --- | --- | --- | --- | --- | --- | --- | --- | --- | --- | --- | --- | --- | --- | --- | --- | --- | --- | --- | --- | --- | --- | --- | --- | --- | --- | --- | --- | --- | --- | --- | --- | --- |
| A. G. B. Stewart (1) | 2020 | Australia | D | L | 0 | NA | NA | NA | 4 | 8 | 0 | NA | NA | NA | NA | NA | NA | NA | NA | NA | 23 | NA | 0 | NA | NA | NA | NA | NA | NA | NA | NA | NA | NA | NA | NA | NA | NA | NA | NA | NA | NA | NA | NA | NA | NA | NA |
| A. G. B. Stewart (1) | 2020 | Australia | Other | L | NA | NA | NA | NA | NA | NA | NA | NA | NA | NA | NA | NA | NA | NA | NA | NA | 22 | NA | NA | NA | NA | NA | NA | NA | NA | NA | NA | NA | NA | NA | NA | NA | NA | NA | NA | NA | NA | NA | NA | NA | NA | NA |
| M. M. Bob-Manuel (2) | 2021 | Nigeria | M | L | 0 | 0 | NA | NA | 8 | 10 | 0 | 0 | NA | NA | 0 | 0 | NA | NA | NA | NA | 33 | NA | NA | NA | 9 | NA | 0 | NA | NA | NA | 0 | NA | NA | NA | NA | NA | 0 | 8 | NA | NA | NA | NA | NA | NA | NA | NA |
| S. R. Abotorabi (3) | 2023 | Iran | D | S | 12 | 6 | NA | NA | 12 | 12 | 8 | 9 | NA | NA | 16 | NA | NA | NA | NA | NA | 12 | NA | NA | NA | NA | NA | NA | NA | NA | NA | NA | NA | 12 | NA | NA | NA | NA | 12 | NA | NA | NA | NA | NA | NA | NA | NA |
| Y. W. Gomi (4) | 2019 | Japan | M | S | 0 | NA | NA | NA | NA | NA | NA | 0 | NA | NA | NA | 0 | NA | 2985 | NA | NA | NA | NA | NA | NA | 1790 | NA | NA | NA | NA | NA | NA | NA | NA | NA | NA | NA | NA | NA | 0 | NA | NA | NA | NA | NA | NA | NA |
| D. S. Miloshevski (5) | 2015 | NA | NA | L | 0 | NA | NA | NA | 0 | 9 | 0 | NA | NA | NA | 0 | NA | NA | NA | NA | NA | 35 | 34 | NA | 0.0 | 0 | 60 | 0 | NA | NA | NA | NA | NA | NA | 0 | NA | NA | NA | 0 | 0 | NA | NA | NA | NA | NA | NA | NA |
| N. S. G. Ngom (6) | 2023 | NA | M | L | 12 | NA | NA | NA | 11 | 23 | 0 | NA | NA | NA | NA | NA | NA | NA | NA | NA | NA | NA | NA | NA | 8 | NA | 0 | NA | 0 | NA | NA | NA | NA | 7 | NA | 0 | NA | 1 | NA | NA | NA | NA | NA | NA | NA | NA |
| R. E. F. Mendes (7) | 2015 | NA | M | S | 0 | NA | NA | NA | 147 | 278 | 0 | NA | NA | NA | NA | NA | NA | NA | NA | NA | 495 | NA | NA | NA | 5 | NA | 0 | 0 | NA | NA | NA | NA | NA | NA | NA | NA | NA | NA | NA | NA | NA | NA | NA | NA | NA | NA |
| R. E. F. Mendes (7) | 2015 | NA | M | S | 0 | NA | NA | NA | 147 | 278 | 0 | NA | NA | NA | NA | NA | NA | NA | NA | NA | 515 | NA | NA | NA | 7 | NA | 0 | 0 | NA | NA | NA | NA | NA | NA | NA | NA | NA | NA | NA | NA | NA | NA | NA | NA | NA | NA |
| D. G. Kekic (8) | 2021 | Serbia | D | L | 0 | NA | NA | NA | 237 | 286 | NA | NA | NA | NA | NA | NA | NA | NA | NA | NA | 912 | NA | NA | NA | NA | NA | NA | NA | NA | NA | NA | NA | NA | NA | NA | NA | NA | NA | NA | NA | NA | NA | NA | NA | NA | NA |
| J. A. N. Karlowsky (9) | 2015 | NA | M | S | NA | NA | NA | NA | NA | NA | 0 | NA | NA | NA | NA | NA | NA | NA | NA | NA | NA | NA | NA | NA | NA | NA | 0 | 0 | NA | NA | NA | NA | NA | NA | NA | NA | NA | NA | NA | NA | NA | NA | NA | NA | NA | NA |
| L. R. S. Duncan (10) | 2017 | NA | M | L | 0 | NA | NA | NA | 96 | 177 | 0 | NA | NA | NA | NA | NA | NA | NA | NA | NA | 305 | NA | NA | NA | 9 | NA | 0 | 0 | NA | NA | NA | NA | NA | NA | NA | NA | NA | NA | NA | NA | NA | NA | NA | NA | NA | NA |
| L. R. S. Duncan (10) | 2017 | NA | M | L | 0 | NA | NA | NA | 97 | 178 | 0 | NA | NA | NA | NA | NA | NA | NA | NA | NA | 321 | NA | NA | NA | 9 | NA | 0 | 0 | NA | NA | NA | NA | NA | NA | NA | NA | NA | NA | NA | NA | NA | NA | NA | NA | NA | NA |
| S. M. N. Mousavi (11) | 2016 | NA | D | L | 0 | 0 | NA | 0 | 0 | 22 | NA | NA | NA | NA | NA | NA | NA | 26 | NA | NA | NA | 62 | NA | NA | 3 | NA | 0 | NA | NA | NA | NA | NA | NA | NA | NA | NA | 0 | 2 | NA | NA | NA | NA | NA | NA | NA | NA |
| C. T. Matani (12) | 2016 | NA | NA | H | 7 | 8 | NA | NA | 28 | 19 | 5 | NA | NA | NA | NA | NA | NA | NA | NA | NA | 10 | NA | 3 | NA | 6 | NA | 0 | NA | 0 | 3 | NA | NA | NA | 0 | NA | 3 | NA | NA | NA | NA | NA | NA | NA | NA | NA | NA |
| F. A. F. D. O. Santana (13) | 2020 | Brazil | D | L | 0 | 0 | NA | NA | 6 | 8 | 0 | NA | NA | NA | 0 | NA | NA | NA | NA | NA | NA | NA | NA | NA | NA | NA | NA | NA | NA | NA | NA | NA | NA | NA | NA | NA | NA | NA | NA | NA | NA | NA | NA | NA | NA | NA |
| G. L.-C. De Figueiredo Sanches (14) | 2021 | Brazil | M | L | 0 | 0 | NA | NA | 3 | 5 | 0 | NA | NA | NA | NA | NA | NA | 5 | NA | NA | 47 | NA | NA | 3.0 | NA | NA | 0 | NA | NA | NA | NA | NA | NA | 0 | NA | NA | NA | NA | NA | 18 | NA | NA | NA | NA | NA | NA |
| M. N. Laczeski (15) | 2014 | NA | M | L | NA | NA | NA | NA | 0 | 0 | NA | NA | NA | NA | NA | NA | NA | NA | NA | NA | NA | NA | NA | NA | NA | NA | NA | NA | NA | NA | NA | NA | NA | NA | NA | NA | NA | NA | NA | NA | NA | NA | NA | NA | NA | NA |
| S. T. Belard (16) | 2015 | NA | D | L | 0 | NA | NA | 0 | 0 | 0 | 0 | NA | NA | 0.0 | NA | NA | NA | NA | NA | NA | NA | NA | NA | NA | NA | NA | 0 | NA | NA | NA | NA | NA | NA | NA | NA | NA | NA | NA | NA | NA | NA | NA | NA | NA | NA | NA |
| N. M. K. Nkembe (17) | 2018 | Cameroon | D | S | NA | 0 | NA | NA | 0 | 1 | 0 | NA | NA | NA | 0 | NA | NA | NA | NA | NA | NA | NA | NA | NA | NA | 5 | NA | NA | NA | NA | NA | NA | NA | NA | 0 | NA | NA | NA | NA | NA | 0 | 3 | NA | NA | NA | NA |
| B. A. S. M. Pimentel (18) | 2016 | Brazil | NA | L | 0 | 0 | NA | 3 | 23 | 7 | 0 | NA | NA | NA | NA | NA | NA | NA | NA | NA | 250 | NA | NA | 5.0 | NA | NA | 0 | NA | NA | NA | NA | NA | NA | 0 | NA | NA | NA | NA | NA | 101 | NA | NA | NA | NA | NA | NA |
| V. G. A. Dutra (19) | 2014 | Brazil | D | S | NA | 13 | NA | NA | 13 | 18 | NA | NA | NA | NA | 13 | NA | NA | NA | NA | NA | NA | NA | NA | NA | 13 | NA | NA | NA | NA | NA | NA | NA | NA | NA | NA | NA | NA | 13 | NA | NA | NA | NA | NA | NA | NA | NA |
| M. A. K. Shadbad (20) | 2020 | Iran | D | L | NA | NA | NA | NA | 86 | 26 | NA | NA | NA | NA | NA | NA | NA | NA | NA | NA | 86 | NA | NA | NA | 6 | NA | NA | NA | NA | NA | NA | NA | NA | 8 | NA | NA | NA | NA | NA | NA | NA | NA | NA | NA | NA | NA |
| E. R. P.-R. Martins (21) | 2017 | Portugal | D | L | 0 | NA | NA | NA | 31 | 35 | 0 | NA | NA | NA | NA | NA | NA | NA | NA | NA | NA | NA | NA | NA | 0 | 1 | NA | NA | NA | NA | NA | NA | NA | NA | NA | NA | NA | 3 | NA | NA | NA | NA | NA | NA | NA | NA |
| E. J. B. Saad (22) | 2018 | Argentina | NA | H | NA | 0 | NA | NA | 2 | 2 | NA | NA | NA | NA | NA | NA | NA | NA | NA | NA | NA | NA | NA | NA | NA | NA | NA | NA | NA | NA | NA | NA | NA | NA | NA | NA | NA | NA | NA | NA | NA | NA | NA | NA | NA | NA |
| M. J.-K. Frej-Madrzak (23) | 2020 | Poland | D | S | 0 | NA | NA | NA | 8 | 12 | NA | NA | NA | NA | NA | NA | NA | NA | NA | NA | NA | NA | NA | NA | NA | NA | NA | NA | NA | NA | NA | NA | NA | NA | NA | NA | NA | NA | NA | NA | NA | NA | NA | NA | NA | NA |
| M. M. Majigo (24) | 2022 | NA | D | L | 93 | 90 | NA | NA | 29 | 85 | NA | 77 | NA | NA | NA | NA | NA | NA | NA | NA | NA | NA | NA | 50.0 | NA | NA | NA | NA | NA | NA | NA | NA | NA | NA | NA | NA | NA | 29 | NA | NA | NA | NA | NA | NA | NA | NA |
| E. S. Shipitsyna (25) | 2020 | NA | D | H | 0 | NA | NA | 0 | NA | 86 | 0 | NA | NA | NA | NA | NA | NA | NA | NA | NA | NA | NA | NA | NA | NA | NA | NA | NA | NA | NA | NA | NA | NA | NA | NA | NA | NA | NA | NA | NA | NA | NA | NA | NA | NA | NA |
| B. J. C. Metcalf (26) | 2017 | NA | M | L | NA | NA | NA | NA | NA | 235 | NA | NA | NA | NA | NA | NA | NA | NA | NA | NA | NA | NA | NA | NA | NA | NA | NA | NA | NA | NA | NA | NA | NA | NA | NA | NA | NA | NA | NA | NA | NA | NA | NA | NA | NA | NA |
| B. J. C. Metcalf (26) | 2017 | NA | Other | L | NA | NA | NA | NA | NA | NA | 2 | NA | NA | NA | NA | NA | NA | NA | NA | NA | NA | NA | NA | NA | NA | NA | NA | NA | NA | NA | NA | NA | NA | NA | NA | NA | NA | 2 | NA | NA | NA | NA | NA | NA | NA | NA |
| K. L. C. Hon (27) | 2020 | NA | M | L | 0 | NA | NA | NA | 4 | 4 | NA | NA | NA | NA | NA | NA | NA | NA | NA | NA | NA | NA | NA | NA | NA | NA | NA | NA | NA | NA | NA | NA | NA | NA | NA | NA | NA | NA | NA | NA | NA | NA | NA | NA | NA | NA |
| A. E. A. A. H. El-Gendy (28) | 2021 | NA | D | L | 0 | 0 | NA | NA | 50 | 20 | 0 | NA | 0 | NA | NA | NA | NA | NA | NA | NA | 470 | NA | NA | NA | NA | NA | 0 | NA | NA | NA | NA | NA | NA | NA | NA | NA | NA | NA | NA | NA | NA | NA | NA | NA | NA | NA |
| F. N. Z. Sulung (29) | 2023 | Malaysia | D | L | 1 | NA | NA | NA | 7 | 5 | NA | NA | NA | NA | NA | NA | NA | NA | NA | NA | 54 | NA | NA | NA | NA | NA | NA | NA | NA | NA | NA | NA | NA | NA | NA | NA | NA | NA | NA | NA | NA | NA | NA | NA | NA | NA |
| P. T. Wang (30) | 2015 | NA | M | L | 0 | NA | NA | NA | 41 | 48 | NA | 0 | NA | NA | NA | NA | NA | 55 | 52 | NA | 51 | NA | NA | NA | 22 | NA | NA | NA | NA | NA | NA | NA | NA | NA | NA | NA | NA | NA | NA | NA | NA | NA | NA | NA | NA | NA |
| S. L. Wang (31) | 2018 | Iran | M | L | 0 | NA | NA | NA | NA | 21 | 0 | 0 | NA | NA | NA | 0 | NA | NA | NA | NA | 26 | NA | NA | NA | 4 | NA | NA | NA | NA | NA | NA | NA | 0 | NA | NA | NA | NA | 0 | NA | NA | NA | NA | NA | NA | NA | NA |
| S. F. Teatero (32) | 2017 | NA | M | L | 0 | 0 | NA | NA | 25 | 37 | 0 | NA | NA | NA | NA | NA | NA | NA | NA | NA | 91 | NA | NA | NA | NA | NA | NA | NA | NA | NA | NA | NA | NA | NA | NA | NA | NA | NA | NA | NA | NA | NA | NA | NA | NA | NA |
| P. M. Wang (33) | 2015 | NA | M | L | 0 | NA | NA | NA | 35 | 37 | NA | 0 | NA | NA | NA | NA | NA | 39 | 37 | NA | 40 | NA | NA | NA | 0 | NA | NA | NA | NA | NA | NA | NA | NA | NA | NA | NA | NA | NA | NA | NA | NA | NA | NA | NA | NA | NA |
| H. C. M. Slotved (34) | 2021 | NA | D | S | 0 | NA | NA | NA | 15 | 23 | NA | NA | NA | NA | NA | NA | NA | NA | NA | NA | NA | NA | NA | NA | NA | NA | NA | NA | NA | NA | NA | NA | NA | NA | NA | NA | NA | NA | NA | NA | NA | NA | NA | NA | NA | NA |
| Y. H. C. Wang (35) | 2015 | Taiwan | D | L | NA | NA | NA | NA | NA | NA | NA | 0 | NA | NA | NA | NA | NA | NA | NA | NA | NA | NA | NA | NA | NA | NA | NA | NA | NA | NA | NA | NA | NA | NA | NA | NA | NA | NA | NA | NA | NA | NA | NA | NA | NA | NA |
| Y. H. C. Wang (35) | 2015 | Taiwan | Other | L | NA | NA | NA | NA | 169 | 171 | NA | NA | NA | NA | NA | NA | NA | 181 | NA | NA | NA | NA | NA | NA | NA | NA | NA | NA | NA | NA | NA | NA | NA | NA | NA | NA | NA | NA | NA | NA | NA | NA | NA | NA | NA | NA |
| S. R.-F. Frohlicher (36) | 2014 | Switzerland | D | S | 0 | NA | NA | NA | 27 | 27 | NA | NA | NA | NA | NA | NA | NA | NA | NA | NA | NA | NA | NA | NA | NA | NA | NA | NA | NA | NA | NA | NA | NA | NA | NA | NA | NA | NA | NA | NA | NA | NA | NA | NA | NA | NA |
| T. O. Ikebe (37) | 2023 | Japan | M | L | 7 | 4 | NA | 5 | 48 | 81 | 0 | NA | NA | NA | 1 | 0 | NA | NA | NA | NA | NA | NA | NA | 90.0 | NA | NA | 0 | 0 | NA | NA | NA | NA | NA | NA | NA | NA | NA | NA | NA | NA | NA | NA | NA | NA | NA | NA |
| E. H. S. Stordal (38) | 2022 | Norway | NA | L | 0 | 0 | NA | NA | NA | NA | 0 | NA | NA | NA | 0 | 0 | NA | NA | NA | NA | NA | NA | NA | NA | NA | NA | NA | NA | NA | NA | NA | NA | NA | NA | NA | NA | NA | NA | NA | NA | NA | NA | NA | NA | NA | NA |
| F. F. Alp (39) | 2016 | NA | D | L | 0 | NA | NA | NA | 6 | 16 | 0 | 0 | NA | NA | NA | NA | NA | NA | NA | NA | 55 | NA | NA | NA | 13 | NA | 0 | NA | NA | NA | NA | NA | NA | NA | NA | NA | NA | NA | NA | NA | NA | NA | NA | NA | NA | NA |
| J. B. Ngonzi (40) | 2018 | NA | D | L | 5 | NA | NA | NA | NA | 3 | NA | NA | NA | NA | NA | NA | NA | NA | NA | NA | 12 | NA | 13 | NA | NA | NA | NA | NA | NA | NA | NA | NA | NA | NA | NA | NA | NA | NA | NA | NA | NA | NA | NA | NA | NA | NA |
| H. G. H. Bae (41) | 2022 | NA | Other | L | 0 | 0 | NA | NA | NA | NA | 0 | 0 | NA | NA | NA | 0 | NA | NA | NA | NA | NA | NA | NA | NA | NA | NA | NA | NA | NA | NA | NA | NA | 0 | NA | NA | NA | NA | NA | NA | NA | NA | NA | NA | NA | NA | NA |
| M. K. Kitamura (42) | 2019 | Japan | M | L | NA | 0 | NA | NA | NA | NA | 0 | 0 | NA | NA | 0 | 0 | NA | NA | NA | NA | NA | NA | NA | NA | 24 | NA | 0 | 0 | NA | NA | NA | NA | NA | NA | NA | NA | 0 | NA | NA | NA | NA | NA | NA | NA | NA | NA |
| K. A. Cooper (43) | 2016 | Japan | M | H | 0 | NA | NA | NA | NA | NA | NA | NA | NA | NA | 3 | NA | NA | NA | NA | NA | NA | NA | NA | NA | NA | NA | NA | NA | NA | NA | NA | NA | NA | NA | 2 | NA | NA | NA | 44 | NA | NA | NA | NA | NA | NA | NA |
| T. V. Aleman (44) | 2022 | NA | C | L | 0 | NA | NA | NA | 27 | 19 | 0 | 0 | NA | NA | NA | 0 | NA | NA | NA | NA | NA | NA | NA | NA | 1 | NA | 0 | NA | NA | NA | NA | NA | NA | NA | NA | NA | NA | NA | NA | NA | NA | NA | NA | NA | NA | NA |
| S. J. K. Choi (45) | 2021 | NA | M | L | 0 | 0 | NA | NA | 12 | 21 | 0 | 0 | NA | NA | 0 | 0 | NA | NA | NA | NA | 32 | NA | NA | NA | 15 | NA | NA | NA | NA | NA | NA | NA | 0 | NA | NA | NA | NA | 3 | NA | NA | NA | NA | NA | NA | NA | NA |
| T. B. Rasamiravaka (46) | 2016 | NA | NA | H | 9 | NA | NA | NA | NA | NA | 1 | NA | NA | NA | NA | NA | NA | NA | NA | NA | NA | NA | NA | NA | NA | NA | NA | NA | NA | NA | NA | NA | NA | NA | 9 | NA | NA | NA | NA | NA | NA | NA | NA | NA | NA | NA |
| D. G. Safari (47) | 2021 | Indonesia | M | L | 0 | 0 | NA | NA | 11 | 10 | 0 | NA | NA | NA | 0 | NA | NA | NA | NA | NA | 47 | NA | NA | NA | 3 | NA | 0 | 0 | NA | NA | NA | NA | NA | NA | NA | NA | NA | NA | NA | NA | NA | NA | NA | NA | NA | NA |
| T. F. Motallebirad (48) | 2021 | Iran | D | L | 0 | NA | NA | NA | 22 | 30 | 0 | 0 | NA | NA | NA | NA | NA | NA | NA | NA | 65 | NA | NA | NA | 7 | NA | NA | NA | NA | NA | NA | NA | 0 | NA | NA | NA | NA | NA | NA | NA | NA | NA | NA | NA | NA | NA |
| R. A.-Z. Dube (49) | 2023 | NA | Other | L | 0 | 3 | NA | NA | 7 | 7 | NA | 1 | NA | NA | 1 | NA | NA | NA | NA | NA | NA | NA | 9 | NA | 2 | NA | 1 | NA | NA | NA | NA | NA | NA | NA | NA | NA | NA | NA | NA | NA | NA | NA | NA | NA | NA | NA |
| S. C. O. Jisuvei (50) | 2020 | Kenya | D | L | 42 | 32 | NA | NA | 14 | NA | 14 | NA | NA | NA | NA | NA | NA | NA | NA | NA | NA | NA | NA | NA | NA | NA | NA | NA | NA | NA | NA | NA | NA | NA | NA | NA | NA | NA | NA | NA | NA | NA | NA | NA | NA | NA |
| O. K. A. Husen (51) | 2023 | NA | D | L | 5 | 3 | NA | NA | 6 | 10 | 1 | NA | NA | NA | NA | NA | NA | NA | NA | NA | NA | NA | NA | NA | NA | NA | NA | NA | NA | NA | NA | NA | 21 | NA | NA | NA | NA | 7 | NA | NA | NA | NA | NA | NA | NA | NA |
| A. M. Bitew (52) | 2021 | NA | D | L | NA | NA | NA | NA | 1 | 13 | NA | 2 | 2 | NA | NA | NA | NA | NA | NA | NA | NA | NA | 12 | 0.0 | NA | 0 | NA | NA | NA | 0 | NA | NA | NA | NA | NA | NA | NA | NA | NA | NA | NA | 5 | NA | NA | NA | NA |
| M. S. P. Abdallah (53) | 2018 | Tanzania | NA | L | NA | 0 | NA | NA | NA | 0 | NA | 0 | NA | NA | NA | NA | NA | NA | NA | NA | NA | NA | 0 | 0.0 | NA | 0 | NA | NA | NA | NA | NA | NA | NA | NA | NA | NA | NA | NA | NA | NA | NA | NA | NA | NA | NA | NA |
| M. A. Misic (54) | 2018 | Serbia | D | L | 0 | NA | NA | NA | 30 | 45 | NA | NA | NA | NA | NA | NA | NA | NA | NA | NA | NA | NA | NA | 45.0 | NA | 101 | NA | NA | NA | NA | NA | NA | NA | NA | NA | NA | NA | NA | NA | NA | NA | NA | NA | NA | NA | NA |
| M. A. Misic (54) | 2018 | Serbia | D | L | 0 | NA | NA | NA | 1 | 2 | NA | NA | NA | NA | NA | NA | NA | NA | NA | NA | NA | NA | NA | 4.0 | NA | 8 | NA | NA | NA | NA | NA | NA | NA | NA | NA | NA | NA | NA | NA | NA | NA | NA | NA | NA | NA | NA |
| S. M. Sahraee (55) | 2019 | Iran | D | L | 39 | 10 | NA | NA | 41 | 20 | 12 | 32 | NA | NA | NA | NA | NA | 37 | NA | NA | 26 | NA | NA | NA | 12 | NA | NA | NA | NA | NA | NA | NA | NA | NA | NA | NA | NA | NA | NA | NA | NA | NA | NA | NA | NA | NA |
| Z. L. T. Woldu (56) | 2014 | Ethiopia | M | H | 8 | 1 | NA | NA | 2 | 2 | NA | NA | NA | NA | NA | NA | NA | NA | NA | NA | NA | NA | NA | NA | NA | NA | NA | NA | NA | NA | NA | NA | NA | NA | NA | NA | NA | NA | NA | NA | NA | NA | NA | NA | NA | NA |
| G. C. A. Soares (57) | 2013 | NA | D | L | 0 | 0 | NA | 0 | 3 | 0 | 0 | NA | NA | NA | NA | NA | 0 | NA | NA | NA | 27 | NA | NA | NA | NA | NA | NA | NA | NA | NA | NA | NA | NA | NA | NA | NA | NA | NA | NA | NA | NA | NA | 0 | NA | NA | NA |
| Y. P. Ge (58) | 2021 | Taiwan | D | L | NA | NA | NA | NA | NA | NA | NA | NA | NA | NA | NA | NA | NA | NA | NA | NA | NA | NA | NA | NA | 117 | NA | NA | NA | NA | NA | NA | NA | NA | NA | NA | NA | NA | NA | NA | NA | NA | NA | NA | NA | NA | NA |
| Y. P. Ge (58) | 2021 | Taiwan | D | L | NA | NA | NA | NA | NA | NA | NA | NA | NA | NA | 2 | NA | NA | NA | NA | NA | NA | NA | NA | NA | NA | NA | NA | NA | NA | NA | NA | NA | NA | NA | NA | NA | NA | NA | NA | NA | NA | NA | NA | NA | NA | NA |
| Y. P. Ge (58) | 2021 | Taiwan | D | L | NA | 3 | NA | NA | NA | NA | 0 | NA | NA | NA | NA | NA | NA | NA | NA | NA | NA | NA | NA | NA | NA | NA | NA | NA | NA | NA | NA | NA | NA | NA | NA | NA | NA | NA | NA | NA | NA | NA | NA | NA | NA | NA |
| Y. P. Ge (58) | 2021 | Taiwan | D | L | 0 | NA | NA | NA | 170 | 193 | NA | NA | NA | NA | NA | NA | NA | NA | NA | NA | NA | NA | NA | NA | NA | NA | 0 | NA | NA | NA | NA | NA | NA | NA | NA | NA | NA | NA | NA | NA | NA | NA | NA | NA | NA | NA |
| Y. P. Ge (58) | 2021 | Taiwan | D | L | NA | NA | NA | NA | NA | NA | NA | NA | NA | NA | NA | NA | NA | NA | NA | NA | NA | NA | NA | NA | NA | NA | NA | NA | NA | NA | NA | NA | 3 | NA | NA | NA | NA | 17 | NA | NA | NA | NA | NA | NA | NA | NA |
| O. O. A. Ojo (59) | 2019 | NA | D | L | NA | 5 | NA | NA | NA | 1 | NA | 1 | 4 | 0.0 | NA | NA | NA | NA | NA | NA | NA | NA | NA | 2.0 | NA | 4 | NA | NA | NA | NA | NA | NA | NA | NA | NA | NA | NA | NA | NA | NA | NA | NA | NA | NA | NA | NA |
| E. A. K. Al Romaihi (60) | 2018 | Bahrain | D | L | NA | NA | NA | NA | NA | NA | NA | NA | NA | NA | NA | NA | NA | NA | NA | NA | NA | NA | 0 | NA | NA | NA | NA | NA | NA | 0 | NA | NA | NA | NA | NA | NA | NA | NA | NA | 0 | NA | NA | NA | NA | NA | NA |
| N. A. J. Al-Tulaibawi (61) | 2019 | NA | D | L | NA | NA | NA | NA | NA | NA | NA | 3 | NA | NA | NA | NA | 0 | NA | NA | NA | 2 | NA | 4 | 1.0 | NA | 2 | NA | NA | NA | 5 | NA | NA | NA | NA | NA | NA | NA | NA | NA | NA | NA | NA | NA | NA | 1 | NA |
| M. A. Qadi (62) | 2021 | Palestinian Territories | D | L | NA | 22 | NA | NA | 6 | 7 | 13 | NA | NA | NA | 11 | NA | NA | NA | NA | NA | 18 | NA | NA | NA | 2 | NA | 0 | NA | NA | NA | NA | NA | NA | NA | NA | NA | NA | NA | NA | NA | NA | NA | NA | NA | NA | NA |
| E. L. M. Haimbodi (63) | 2021 | Namibia | D | L | 0 | 0 | NA | NA | 0 | 0 | 0 | 0 | NA | NA | NA | NA | NA | NA | NA | NA | NA | NA | NA | NA | NA | NA | NA | NA | NA | NA | NA | NA | NA | NA | NA | NA | NA | 0 | NA | NA | NA | NA | NA | NA | NA | NA |
| K. B. B. O. Luiz Fb Alves (64) | 2021 | NA | D | L | 0 | NA | NA | NA | NA | NA | 0 | 0 | NA | NA | NA | NA | NA | NA | NA | NA | 2 | NA | NA | NA | 0 | NA | NA | NA | NA | NA | NA | NA | NA | NA | NA | NA | NA | NA | NA | NA | NA | NA | NA | NA | NA | NA |
| C. E. Njoku (65) | 2018 | NA | NA | L | 4 | 3 | NA | NA | 8 | 13 | NA | 16 | NA | NA | NA | NA | NA | NA | NA | NA | NA | NA | NA | NA | NA | NA | NA | NA | NA | NA | NA | NA | NA | NA | NA | NA | NA | NA | NA | NA | NA | NA | NA | NA | NA | NA |
| H. S. Jiang (66) | 2017 | Taiwan | D | L | NA | 0 | NA | NA | 10 | 12 | 0 | NA | NA | NA | NA | NA | NA | NA | NA | NA | 13 | NA | NA | 4.0 | 2 | NA | 0 | NA | NA | NA | NA | NA | NA | NA | NA | NA | NA | 11 | NA | NA | NA | NA | NA | 2 | NA | NA |
| F. S. Numanovic (67) | 2017 | Bosnia & Herzegovina | C | L | NA | 0 | NA | NA | 6 | 6 | 0 | NA | NA | NA | 0 | NA | NA | NA | NA | NA | NA | NA | NA | NA | 0 | NA | 0 | NA | NA | NA | NA | NA | 0 | NA | NA | NA | NA | NA | NA | NA | NA | NA | NA | NA | NA | NA |
| N. K. Nagano (68) | 2019 | Japan | M | L | 77 | 38 | NA | NA | 18 | 57 | 0 | 32 | NA | NA | 75 | 0 | NA | NA | 57 | NA | NA | NA | NA | NA | 35 | NA | NA | NA | NA | NA | NA | NA | 77 | NA | NA | NA | NA | NA | NA | NA | NA | NA | NA | NA | NA | NA |
| S. M. Teatero (69) | 2015 | Canada | M | L | 0 | 0 | NA | NA | 19 | 20 | 0 | NA | NA | NA | 0 | NA | NA | NA | NA | NA | 23 | NA | NA | NA | 0 | NA | NA | NA | NA | NA | NA | NA | NA | NA | NA | NA | NA | NA | NA | NA | NA | NA | NA | NA | NA | NA |
| D. G. R. Jamrozy (70) | 2023 | Canada | Other | H | NA | NA | NA | NA | 13 | NA | NA | NA | NA | NA | NA | NA | NA | NA | 111 | NA | NA | NA | NA | NA | NA | NA | NA | NA | NA | NA | NA | NA | NA | NA | NA | NA | NA | NA | NA | NA | NA | NA | NA | NA | NA | NA |
| F. N. Khodaei (71) | 2018 | NA | M | L | 0 | NA | NA | NA | 80 | 24 | 0 | NA | NA | NA | NA | NA | NA | NA | NA | NA | 80 | NA | NA | NA | 5 | NA | NA | NA | NA | NA | NA | NA | NA | 7 | NA | NA | NA | NA | NA | NA | NA | NA | NA | NA | NA | NA |
| X. C. Wang (72) | 2018 | NA | D | L | NA | NA | NA | NA | 70 | 75 | NA | NA | NA | NA | NA | NA | NA | NA | NA | NA | 80 | NA | NA | NA | 30 | NA | NA | NA | NA | NA | NA | NA | NA | NA | NA | NA | NA | 9 | NA | NA | NA | NA | NA | NA | NA | NA |
| M. B. Novosak (73) | 2020 | NA | M | L | NA | NA | NA | NA | 10 | 12 | NA | NA | NA | NA | NA | NA | NA | NA | NA | NA | NA | NA | NA | NA | NA | NA | NA | NA | NA | NA | NA | NA | NA | NA | NA | NA | NA | NA | NA | NA | NA | NA | NA | NA | NA | NA |
| B. S. Wu (74) | 2019 | NA | NA | L | 0 | NA | NA | NA | 86 | 78 | 0 | NA | NA | NA | NA | NA | NA | NA | NA | NA | 91 | NA | NA | NA | 28 | NA | NA | NA | NA | NA | NA | NA | NA | NA | NA | NA | NA | NA | NA | NA | NA | NA | NA | NA | NA | NA |
| M. H. Malek-Jafarian (75) | 2015 | NA | M | S | 18 | 0 | NA | NA | 13 | 16 | 2 | 0 | NA | NA | NA | NA | NA | NA | NA | NA | NA | NA | NA | NA | NA | 66 | NA | NA | NA | NA | NA | NA | NA | NA | NA | NA | NA | NA | NA | NA | NA | NA | NA | NA | 66 | 66 |
| Y. Y. K. Li (76) | 2019 | Hong Kong SAR China | NA | S | 0 | NA | NA | NA | NA | 19 | NA | NA | NA | NA | NA | NA | NA | NA | NA | NA | NA | NA | NA | NA | NA | NA | NA | NA | NA | NA | NA | NA | NA | NA | NA | NA | NA | NA | NA | NA | NA | NA | NA | NA | NA | NA |
| J. F. Liu (77) | 2021 | Hong Kong SAR China | D | L | NA | NA | NA | NA | 49 | NA | 0 | NA | NA | NA | NA | NA | NA | NA | NA | NA | NA | NA | NA | NA | NA | NA | NA | NA | NA | NA | NA | NA | NA | NA | NA | NA | NA | NA | NA | NA | NA | NA | NA | NA | NA | NA |
| J. F. Liu (77) | 2021 | Hong Kong SAR China | D | L | NA | NA | NA | NA | NA | 54 | NA | NA | NA | NA | NA | NA | NA | NA | NA | NA | NA | NA | NA | NA | NA | NA | NA | NA | NA | NA | NA | NA | NA | NA | NA | NA | NA | NA | NA | NA | NA | NA | NA | NA | NA | NA |
| D. L. R. Horn (78) | 2021 | Unknown | D | L | NA | NA | NA | NA | 9 | NA | NA | NA | NA | NA | NA | NA | NA | NA | NA | NA | NA | NA | NA | NA | NA | NA | NA | NA | NA | NA | NA | NA | NA | NA | NA | NA | NA | NA | NA | NA | NA | NA | NA | NA | NA | NA |
| D. L. R. Horn (79) | 2021 | Unknown | D | L | NA | NA | NA | NA | NA | 8 | NA | NA | NA | NA | NA | NA | NA | NA | NA | NA | NA | NA | NA | NA | NA | NA | NA | NA | NA | NA | NA | NA | NA | NA | NA | NA | NA | NA | NA | NA | NA | NA | NA | NA | NA | NA |
| X. O. G. Li (80) | 2023 | NA | NA | L | 0 | 0 | NA | NA | 50 | 72 | 0 | 0 | NA | NA | NA | 0 | NA | NA | NA | NA | 80 | NA | NA | NA | 46 | NA | 0 | NA | NA | NA | NA | NA | NA | NA | NA | NA | NA | NA | NA | NA | NA | NA | NA | NA | NA | NA |
| G. W. Li (81) | 2022 | NA | M | L | NA | NA | NA | NA | 131 | 75 | NA | NA | NA | NA | NA | NA | NA | NA | NA | NA | NA | 35 | NA | NA | 40 | NA | NA | NA | 0 | NA | NA | NA | NA | NA | NA | NA | NA | 25 | NA | NA | NA | NA | NA | NA | NA | NA |
| A. F. Ali Hamad (82) | 2023 | NA | D | L | 8 | NA | NA | NA | 30 | 43 | NA | NA | NA | NA | NA | NA | NA | NA | NA | NA | 35 | NA | NA | 11.0 | NA | NA | NA | NA | NA | NA | NA | NA | NA | NA | NA | NA | NA | 20 | NA | NA | NA | NA | NA | NA | NA | NA |
| J. G. Yayan (83) | 2015 | Germany | M | L | 0 | 0 | 0 | NA | 6 | 8 | 0 | NA | NA | 0.0 | 0 | NA | NA | NA | NA | NA | NA | NA | 3 | 1.0 | 1 | 8 | NA | NA | NA | NA | NA | NA | NA | NA | NA | NA | NA | NA | NA | NA | NA | NA | NA | NA | NA | NA |
| M. T. Gizachew (84) | 2018 | Ethiopia | D | L | 8 | 4 | NA | NA | 14 | 16 | 12 | 21 | NA | NA | NA | NA | NA | 7 | NA | NA | 48 | NA | NA | NA | NA | NA | NA | NA | NA | NA | NA | NA | NA | NA | NA | NA | NA | 5 | NA | NA | NA | NA | NA | NA | NA | NA |
| L. D. Mudzikati (85) | 2015 | Botswana | D | L | 9 | 9 | NA | NA | NA | NA | 13 | NA | NA | NA | 2 | NA | NA | NA | NA | NA | NA | NA | NA | NA | NA | NA | NA | NA | NA | NA | NA | NA | NA | NA | NA | NA | NA | NA | NA | NA | NA | NA | NA | NA | 1 | NA |
| K. B. Venkatnarayan (86) | 2014 | Nepal | NA | H | 0 | 0 | NA | NA | NA | NA | NA | NA | NA | NA | NA | NA | NA | NA | NA | NA | NA | NA | NA | NA | NA | NA | NA | NA | NA | NA | NA | NA | NA | NA | NA | NA | NA | NA | NA | NA | NA | NA | NA | NA | NA | NA |
| M. M. Dehdashtian (87) | 2021 | NA | D | L | NA | 1 | NA | NA | NA | NA | 0 | NA | NA | NA | 0 | NA | 0 | NA | NA | NA | NA | NA | 2 | NA | NA | 0 | NA | NA | NA | NA | NA | NA | NA | 0 | 0 | NA | NA | NA | NA | NA | NA | NA | NA | NA | 0 | NA |
| R. I. Creti (88) | 2017 | NA | NA | H | NA | NA | NA | NA | 14 | 17 | NA | NA | NA | NA | NA | NA | NA | NA | NA | NA | 17 | NA | NA | NA | 9 | NA | NA | NA | NA | NA | NA | NA | NA | NA | NA | NA | NA | NA | NA | NA | NA | NA | NA | NA | NA | NA |
| D. C. Guo (89) | 2018 | NA | D | L | 0 | NA | NA | NA | NA | NA | 0 | 0 | NA | NA | NA | NA | NA | NA | NA | NA | NA | NA | NA | NA | NA | NA | 0 | NA | NA | NA | NA | NA | NA | NA | NA | NA | NA | NA | NA | NA | NA | NA | NA | NA | NA | NA |
| M. J. Ghamari (90) | 2022 | Iran | D | L | 0 | NA | NA | NA | 24 | 41 | 0 | NA | NA | NA | NA | NA | NA | NA | NA | NA | 141 | NA | NA | NA | NA | NA | 0 | NA | NA | NA | NA | NA | NA | NA | NA | NA | 0 | NA | NA | NA | NA | NA | NA | NA | NA | NA |
| M. A. Alzayer (91) | 2023 | Saudi Arabia | D | L | NA | NA | NA | NA | 52 | 75 | NA | NA | NA | NA | NA | NA | NA | NA | NA | NA | 156 | NA | NA | NA | 13 | NA | NA | NA | NA | NA | NA | NA | NA | NA | NA | NA | NA | NA | NA | NA | NA | NA | NA | NA | NA | NA |
| N. B. Gharabeigi (92) | 2023 | Iran | D | L | 11 | 49 | NA | NA | 14 | 10 | 18 | NA | NA | NA | NA | NA | NA | NA | NA | NA | 52 | NA | NA | NA | 5 | NA | NA | NA | NA | NA | NA | NA | NA | NA | NA | NA | NA | 1 | NA | NA | NA | NA | NA | NA | NA | NA |
| S. P. Dehbashi (93) | 2015 | NA | NA | L | 18 | 21 | NA | NA | 9 | 8 | 12 | 12 | NA | NA | NA | NA | NA | NA | NA | NA | NA | NA | NA | NA | NA | NA | NA | NA | NA | NA | NA | NA | NA | NA | NA | NA | NA | NA | NA | NA | NA | NA | NA | NA | NA | NA |
| R. B. B. A. Schuab (94) | 2015 | NA | D | L | 194 | NA | NA | NA | 18 | 15 | 194 | 194 | NA | NA | NA | NA | NA | NA | NA | NA | 147 | NA | NA | NA | 194 | NA | NA | NA | NA | NA | NA | NA | NA | NA | NA | NA | NA | NA | NA | NA | NA | NA | NA | NA | NA | NA |
| I. P. Gajic (95) | 2019 | Serbia | D | L | 0 | NA | NA | NA | 92 | 100 | 0 | NA | NA | NA | NA | NA | NA | NA | NA | NA | 374 | NA | NA | NA | NA | NA | NA | NA | NA | NA | NA | NA | NA | NA | NA | NA | NA | 0 | NA | NA | NA | NA | 0 | NA | NA | NA |
| A. L. Bergal (96) | 2015 | NA | M | L | 0 | NA | NA | NA | 35 | 74 | NA | NA | NA | NA | NA | NA | NA | NA | NA | NA | NA | NA | NA | NA | NA | NA | NA | NA | NA | NA | NA | NA | NA | NA | NA | NA | NA | NA | NA | NA | NA | NA | NA | NA | NA | NA |
| K. S. Shrestha (97) | 2020 | NA | D | L | 12 | NA | NA | NA | 10 | 8 | 6 | 20 | NA | NA | NA | NA | NA | NA | NA | NA | 21 | NA | NA | NA | 4 | NA | 0 | NA | NA | NA | NA | NA | NA | NA | NA | NA | NA | 8 | NA | NA | NA | NA | NA | NA | NA | NA |
| M. T. Gizachew (98) | 2020 | Ethiopia | D | L | 1 | 0 | NA | NA | 1 | 2 | 3 | 4 | NA | NA | NA | NA | NA | NA | 2 | NA | 14 | NA | NA | 6.0 | NA | NA | NA | NA | NA | NA | NA | NA | NA | NA | NA | NA | NA | 3 | NA | NA | NA | NA | NA | NA | NA | NA |
| H. C. Jiang (99) | 2016 | NA | D | L | NA | NA | NA | NA | 44 | 60 | NA | 7 | NA | NA | 1 | NA | NA | NA | NA | NA | NA | NA | NA | 22.0 | NA | NA | 2 | NA | NA | NA | NA | NA | NA | NA | NA | NA | NA | NA | NA | NA | NA | NA | NA | NA | NA | NA |
| S. V. Simoni (100) | 2018 | Italy | M | L | NA | NA | NA | NA | NA | NA | NA | NA | NA | NA | NA | NA | NA | NA | NA | NA | NA | NA | NA | NA | 11 | NA | NA | NA | NA | NA | NA | NA | NA | NA | NA | NA | NA | NA | NA | NA | NA | NA | NA | NA | NA | NA |
| B. L.-M. Molto-Garcia (101) | 2016 | Ireland | D | L | 0 | NA | NA | NA | 20 | 25 | NA | NA | NA | NA | NA | NA | NA | NA | NA | NA | NA | NA | NA | NA | NA | NA | NA | NA | NA | NA | NA | NA | NA | NA | NA | NA | NA | NA | NA | NA | NA | NA | NA | NA | NA | NA |
| M. U. Kawaguchiya (102) | 2022 | Japan | M | S | 0 | 0 | NA | NA | 23 | 28 | 0 | NA | NA | NA | 0 | 0 | 0 | 38 | 32 | NA | NA | NA | NA | NA | 12 | NA | NA | NA | NA | NA | NA | NA | NA | NA | NA | NA | NA | NA | NA | NA | NA | NA | NA | NA | NA | NA |
| J. J. Li (103) | 2019 | Taiwan | Other | L | 0 | 0 | NA | NA | 61 | 57 | NA | NA | NA | NA | NA | NA | NA | 1 | NA | NA | 87 | NA | NA | NA | 8 | NA | NA | NA | NA | NA | NA | NA | NA | NA | NA | NA | NA | NA | NA | NA | NA | NA | NA | 2 | NA | NA |
| M. H. H. Tsai (104) | 2019 | Taiwan | M | L | 0 | 0 | NA | NA | 158 | 166 | 0 | NA | NA | NA | 0 | NA | NA | NA | NA | NA | NA | NA | NA | NA | NA | NA | NA | NA | NA | NA | NA | NA | NA | NA | NA | NA | NA | NA | NA | NA | NA | NA | NA | NA | NA | NA |
| B. C. Lu (105) | 2016 | NA | M | S | 0 | 0 | NA | NA | 124 | 143 | 0 | NA | NA | NA | 1 | NA | NA | NA | NA | NA | 133 | NA | NA | NA | NA | NA | NA | NA | NA | NA | NA | NA | NA | NA | NA | NA | NA | NA | NA | NA | NA | NA | NA | NA | NA | NA |
| Z. J. Liu (106) | 2023 | NA | D | L | 0 | 0 | NA | NA | NA | 106 | 0 | NA | NA | NA | NA | NA | NA | NA | NA | NA | 59 | NA | NA | NA | 69 | NA | 0 | NA | 0 | NA | NA | NA | NA | NA | NA | NA | 0 | 10 | NA | NA | NA | NA | NA | NA | NA | NA |
| A. O. Dobrut (107) | 2022 | NA | D | L | NA | NA | NA | NA | 17 | 21 | NA | NA | NA | NA | NA | NA | NA | NA | NA | NA | NA | NA | NA | NA | NA | NA | NA | NA | NA | NA | NA | NA | NA | NA | NA | NA | NA | NA | NA | NA | NA | NA | NA | NA | NA | NA |
| K. A. B. Al Benwan (108) | 2023 | Kuwait | D | L | 0 | 0 | NA | NA | 6 | 6 | 0 | NA | NA | NA | 0 | NA | NA | NA | NA | NA | NA | NA | NA | NA | NA | NA | 0 | NA | NA | NA | NA | NA | NA | NA | NA | NA | NA | NA | NA | NA | NA | NA | NA | NA | NA | NA |
| G. L. P. Wilkie (109) | 2019 | NA | NA | S | NA | 0 | NA | NA | 9 | NA | NA | NA | NA | NA | NA | NA | NA | NA | NA | NA | NA | NA | NA | NA | NA | NA | NA | NA | NA | NA | NA | NA | NA | NA | NA | NA | NA | NA | NA | NA | NA | NA | NA | NA | NA | NA |
| Y. Y. Qiu (110) | 2019 | NA | D | L | 0 | NA | NA | NA | NA | NA | NA | NA | NA | NA | NA | NA | NA | NA | NA | NA | NA | NA | NA | NA | NA | NA | NA | NA | NA | NA | NA | NA | NA | NA | NA | NA | NA | NA | NA | NA | NA | NA | NA | NA | NA | NA |
| Y. Y. Qiu (110) | 2019 | NA | D | L | NA | NA | NA | NA | NA | NA | NA | NA | NA | NA | NA | NA | NA | NA | NA | NA | NA | NA | NA | NA | NA | NA | NA | NA | NA | NA | NA | NA | 1 | NA | NA | NA | NA | NA | NA | NA | NA | NA | NA | NA | NA | NA |
| Y. Y. Qiu (110) | 2019 | NA | D | L | NA | NA | NA | NA | NA | NA | NA | 17 | NA | NA | NA | NA | NA | NA | NA | NA | NA | NA | NA | NA | NA | NA | NA | NA | NA | NA | NA | NA | NA | NA | NA | NA | NA | NA | NA | NA | NA | NA | NA | NA | NA | NA |
| Y. Y. Qiu (110) | 2019 | NA | D | L | NA | NA | NA | NA | NA | NA | 0 | NA | NA | NA | NA | NA | NA | NA | NA | NA | NA | NA | NA | NA | NA | NA | NA | NA | NA | NA | NA | NA | NA | NA | NA | NA | NA | NA | NA | NA | NA | NA | NA | NA | NA | NA |
| Y. Y. Qiu (110) | 2019 | NA | D | L | NA | NA | NA | NA | NA | NA | NA | NA | NA | NA | NA | NA | NA | NA | NA | NA | NA | NA | NA | NA | NA | NA | 0 | NA | NA | NA | NA | NA | NA | NA | NA | NA | NA | NA | NA | NA | NA | NA | NA | NA | NA | NA |
| Y. Y. Qiu (110) | 2019 | NA | D | L | NA | NA | NA | NA | 114 | NA | NA | NA | NA | NA | NA | NA | NA | NA | NA | NA | NA | NA | NA | NA | NA | NA | NA | NA | NA | NA | NA | NA | NA | NA | NA | NA | NA | NA | NA | NA | NA | NA | NA | NA | NA | NA |
| Y. Y. Qiu (110) | 2019 | NA | D | L | NA | NA | NA | NA | NA | 75 | NA | NA | NA | NA | NA | NA | NA | NA | NA | NA | NA | NA | NA | NA | NA | NA | NA | NA | NA | NA | NA | NA | NA | NA | NA | NA | NA | NA | NA | NA | NA | NA | NA | NA | NA | NA |
| Y. Y. Qiu (110) | 2019 | NA | D | L | NA | NA | NA | NA | NA | NA | NA | NA | NA | NA | NA | NA | NA | NA | NA | NA | NA | NA | NA | NA | 55 | NA | NA | NA | NA | NA | NA | NA | NA | NA | NA | NA | NA | NA | NA | NA | NA | NA | NA | NA | NA | NA |
| Y. Y. Qiu (110) | 2019 | NA | D | L | NA | NA | NA | NA | NA | NA | NA | NA | NA | NA | NA | NA | NA | NA | NA | NA | NA | NA | NA | NA | NA | NA | NA | NA | NA | NA | NA | NA | NA | NA | NA | NA | NA | 2 | NA | NA | NA | NA | NA | NA | NA | NA |
| Y. Y. Qiu (110) | 2019 | NA | D | L | NA | NA | NA | NA | NA | NA | NA | NA | NA | NA | NA | NA | NA | NA | NA | NA | 126 | NA | NA | NA | NA | NA | NA | NA | NA | NA | NA | NA | NA | NA | NA | NA | NA | NA | NA | NA | NA | NA | NA | NA | NA | NA |
| Y. Y. Qiu (110) | 2019 | NA | D | L | NA | NA | NA | NA | NA | NA | NA | NA | NA | NA | NA | NA | 0 | NA | NA | NA | NA | NA | NA | NA | NA | NA | NA | NA | NA | NA | NA | NA | NA | NA | NA | NA | NA | NA | NA | NA | NA | NA | NA | NA | NA | NA |
| M. A. Ali (111) | 2022 | Qatar | M | L | 0 | NA | NA | NA | 27 | 96 | 0 | 0 | NA | NA | NA | NA | NA | NA | NA | NA | NA | NA | NA | NA | NA | NA | NA | NA | NA | NA | NA | NA | NA | NA | NA | NA | NA | NA | NA | NA | NA | NA | NA | NA | NA | NA |
| B. W. Lu (112) | 2018 | NA | M | L | 0 | 0 | NA | NA | 73 | 75 | 0 | 0 | NA | NA | NA | NA | NA | NA | NA | NA | 77 | NA | NA | NA | 48 | NA | NA | NA | NA | NA | NA | NA | NA | NA | NA | NA | NA | NA | NA | NA | NA | NA | NA | NA | NA | NA |
| W. T.-U. Hiriote (113) | 2017 | Thailand | D | L | 0 | 0 | NA | NA | 10 | 10 | 0 | 0 | NA | 0.0 | NA | NA | NA | NA | NA | NA | 75 | NA | NA | NA | NA | NA | 0 | NA | NA | NA | NA | NA | NA | NA | NA | NA | NA | NA | NA | NA | NA | NA | NA | NA | NA | NA |
| P. F. Liu (114) | 2022 | NA | M | L | 0 | 0 | NA | NA | 10 | 18 | 0 | NA | NA | NA | NA | NA | NA | NA | NA | NA | 40 | NA | NA | NA | 7 | NA | 0 | NA | NA | NA | NA | NA | NA | NA | NA | NA | 0 | NA | NA | NA | NA | NA | NA | NA | NA | NA |
| L. M. Madrid (115) | 2018 | Mozambique | D | L | 0 | 0 | NA | NA | 20 | 34 | NA | 0 | NA | NA | NA | NA | NA | NA | NA | NA | NA | NA | NA | NA | NA | NA | NA | NA | NA | NA | NA | NA | NA | NA | NA | NA | NA | NA | NA | NA | NA | NA | NA | NA | NA | NA |
| S. M. Rostami (116) | 2021 | NA | D | L | NA | NA | NA | NA | 8 | 12 | 0 | 5 | NA | NA | NA | NA | NA | NA | NA | NA | NA | NA | NA | NA | 3 | NA | NA | NA | NA | NA | NA | NA | NA | NA | NA | NA | NA | NA | NA | NA | NA | NA | NA | NA | NA | NA |
| S. A. Shabayek (117) | 2014 | Egypt | D | L | 0 | 0 | NA | NA | 14 | 17 | 0 | NA | NA | NA | 0 | NA | NA | 16 | NA | NA | 98 | NA | NA | NA | 0 | NA | NA | NA | NA | NA | NA | NA | NA | NA | NA | NA | NA | 1 | NA | NA | NA | NA | NA | NA | NA | NA |
| H. K. Hou (118) | 2017 | Iran | M | L | NA | NA | NA | NA | 6 | 9 | 0 | NA | NA | NA | NA | NA | NA | NA | NA | NA | NA | 23 | NA | NA | 3 | NA | 0 | NA | NA | NA | NA | NA | NA | NA | NA | NA | NA | NA | NA | NA | NA | NA | NA | NA | NA | NA |
| H. K. Moroi (119) | 2019 | Japan | NA | L | 0 | 0 | NA | 0 | 0 | 0 | NA | NA | NA | NA | NA | 0 | NA | NA | NA | NA | NA | NA | NA | NA | NA | NA | NA | NA | NA | NA | NA | NA | NA | NA | 0 | NA | NA | NA | 0 | NA | NA | NA | NA | NA | NA | NA |
| A. M. Shafi (120) | 2016 | NA | Other | H | NA | NA | NA | NA | NA | NA | NA | NA | NA | NA | NA | NA | NA | NA | NA | NA | NA | NA | NA | 10.0 | NA | 12 | NA | NA | NA | NA | NA | NA | NA | NA | NA | NA | NA | NA | NA | NA | 20 | NA | NA | NA | NA | NA |
| Y. Z. H. Yan \ (121) | 2016 | NA | D | H | NA | NA | NA | NA | 115 | 137 | NA | NA | NA | NA | NA | NA | NA | NA | NA | NA | NA | NA | NA | NA | 79 | NA | NA | NA | NA | NA | NA | NA | NA | NA | NA | NA | NA | NA | NA | NA | NA | NA | NA | NA | NA | NA |
| A. M. J. Ebrahem (122) | 2023 | NA | D | S | NA | 0 | NA | NA | 2 | NA | 2 | NA | NA | NA | 0 | NA | NA | NA | NA | NA | 0 | NA | 0 | 2.0 | NA | NA | NA | NA | NA | NA | NA | NA | NA | NA | NA | NA | NA | 2 | NA | NA | 1 | NA | NA | 2 | 1 | 2 |
| L. A. K. Al Abbas (123) | 2022 | Syria | D | L | NA | NA | NA | NA | NA | 7 | 10 | 7 | NA | NA | 7 | NA | 0 | 7 | 7 | NA | NA | NA | 7 | 5.0 | 3 | 7 | 0 | NA | NA | NA | NA | NA | 7 | NA | NA | NA | NA | NA | NA | NA | 5 | NA | 5 | 5 | 3 | NA |
| X. H. Vuillemin (124) | 2021 | NA | NA | L | 0 | NA | NA | NA | 784 | 490 | 0 | NA | NA | NA | NA | NA | NA | NA | NA | NA | 1470 | NA | NA | NA | NA | NA | NA | NA | NA | NA | NA | NA | NA | NA | NA | NA | NA | NA | NA | NA | 0 | NA | NA | NA | NA | NA |
| E. H. Graux (125) | 2021 | Germany | D | L | 0 | NA | NA | NA | NA | NA | NA | NA | NA | NA | NA | NA | NA | NA | NA | NA | NA | NA | NA | NA | NA | NA | NA | NA | NA | NA | NA | NA | NA | NA | NA | NA | NA | NA | NA | NA | NA | NA | NA | NA | NA | NA |
| E. H. Graux (125) | 2021 | Germany | D | L | NA | NA | NA | NA | NA | 93 | 0 | NA | NA | NA | NA | NA | NA | NA | NA | NA | NA | NA | NA | NA | NA | NA | NA | NA | NA | NA | NA | NA | NA | NA | NA | NA | NA | NA | NA | NA | NA | NA | NA | NA | NA | NA |
| E. H. Graux (125) | 2021 | Germany | D | L | NA | NA | NA | NA | 72 | NA | NA | NA | NA | NA | NA | NA | NA | NA | NA | NA | NA | NA | NA | NA | NA | NA | NA | NA | NA | NA | NA | NA | NA | NA | NA | NA | NA | NA | NA | NA | NA | NA | NA | NA | NA | NA |
| E. H. Graux (125) | 2021 | Germany | D | L | NA | NA | NA | NA | NA | NA | NA | NA | NA | NA | NA | NA | NA | NA | NA | NA | NA | NA | NA | NA | 7 | NA | NA | NA | NA | NA | NA | NA | NA | NA | NA | NA | NA | NA | NA | NA | NA | NA | NA | NA | NA | NA |
| J. Z. Zhang (126) | 2015 | NA | Other | L | 0 | 0 | NA | NA | 29 | 19 | 0 | NA | NA | NA | NA | NA | NA | NA | NA | NA | 42 | NA | NA | 0.0 | 0 | NA | 0 | NA | 0 | NA | NA | NA | NA | 0 | NA | NA | NA | NA | NA | NA | NA | NA | NA | NA | NA | NA |
| B. K. Sigauque (127) | 2018 | Mozambique | Other | L | 2 | NA | NA | NA | NA | 6 | NA | NA | NA | NA | NA | NA | NA | NA | NA | NA | NA | NA | NA | NA | NA | NA | NA | NA | NA | NA | NA | NA | NA | NA | NA | NA | NA | NA | NA | NA | NA | NA | NA | NA | NA | NA |
| I. A.-A. Al-Subol (128) | 2022 | NA | D | L | 0 | 0 | NA | NA | 2 | NA | 0 | NA | NA | NA | 0 | NA | NA | NA | NA | NA | 11 | NA | NA | NA | 0 | NA | NA | NA | NA | NA | NA | NA | NA | NA | NA | NA | NA | NA | NA | NA | NA | NA | NA | NA | NA | NA |
| S. N. A. Alani (129) | 2022 | NA | Other | S | 22 | 20 | NA | NA | 18 | 22 | NA | 22 | NA | NA | 22 | 22 | NA | 18 | NA | NA | 21 | NA | NA | NA | 5 | NA | NA | NA | NA | NA | NA | NA | 20 | NA | NA | NA | NA | 9 | NA | NA | NA | NA | NA | NA | NA | NA |
| D. R. Kaminska (130) | 2020 | NA | M | L | 0 | NA | NA | NA | NA | NA | NA | NA | NA | NA | NA | NA | NA | NA | NA | NA | 154 | NA | NA | NA | 0 | NA | NA | NA | 0 | NA | NA | NA | NA | NA | NA | NA | NA | NA | NA | NA | NA | NA | NA | NA | NA | NA |
| E. F. Lopes (131) | 2017 | Portugal | D | L | 0 | NA | NA | NA | 0 | 0 | 0 | NA | NA | NA | NA | NA | NA | NA | NA | NA | 478 | NA | NA | NA | 0 | 0 | NA | NA | NA | NA | NA | NA | NA | NA | NA | NA | NA | 0 | NA | NA | NA | NA | NA | NA | NA | NA |
| K. K. F. Gao (132) | 2019 | NA | D | L | 0 | NA | NA | NA | NA | NA | 0 | NA | NA | NA | NA | NA | NA | NA | NA | NA | NA | NA | NA | NA | NA | NA | NA | NA | NA | NA | NA | NA | NA | NA | NA | NA | NA | NA | NA | NA | NA | NA | NA | NA | NA | NA |
| K. K. F. Gao (132) | 2019 | NA | D | L | NA | 0 | NA | NA | NA | NA | NA | NA | NA | NA | NA | NA | NA | NA | NA | NA | NA | NA | NA | NA | NA | NA | NA | NA | NA | NA | NA | NA | NA | NA | NA | NA | NA | NA | NA | NA | NA | NA | NA | NA | NA | NA |
| K. K. F. Gao (132) | 2019 | NA | D | L | NA | NA | NA | NA | NA | 61 | NA | NA | NA | NA | NA | NA | NA | NA | NA | NA | NA | NA | NA | NA | NA | NA | NA | NA | NA | NA | NA | NA | NA | NA | NA | NA | NA | NA | NA | NA | NA | NA | NA | NA | NA | NA |
| K. K. F. Gao (132) | 2019 | NA | D | L | NA | NA | NA | NA | NA | NA | NA | NA | NA | NA | NA | NA | NA | NA | NA | NA | NA | NA | NA | 10.0 | NA | NA | NA | NA | NA | NA | NA | NA | NA | NA | NA | NA | 0 | NA | NA | NA | NA | NA | NA | NA | NA | NA |
| K. K. F. Gao (132) | 2019 | NA | D | L | NA | NA | NA | NA | NA | NA | NA | NA | NA | NA | NA | NA | NA | NA | NA | NA | NA | NA | NA | NA | NA | NA | NA | NA | NA | 0 | NA | NA | NA | NA | NA | NA | NA | NA | NA | NA | NA | NA | NA | NA | NA | NA |
| K. K. F. Gao (132) | 2019 | NA | D | L | NA | NA | NA | NA | NA | NA | NA | NA | NA | NA | NA | NA | NA | NA | NA | NA | NA | NA | NA | NA | NA | NA | NA | NA | NA | NA | NA | NA | NA | 8 | NA | NA | NA | NA | NA | NA | NA | NA | NA | NA | NA | NA |
| K. K. F. Gao (132) | 2019 | NA | D | L | NA | NA | NA | NA | NA | NA | NA | NA | NA | NA | NA | NA | NA | NA | NA | NA | NA | NA | NA | NA | NA | NA | NA | NA | 0 | NA | NA | NA | NA | NA | NA | NA | NA | NA | NA | NA | NA | NA | NA | NA | NA | NA |
| K. K. F. Gao (132) | 2019 | NA | D | L | NA | NA | NA | NA | NA | NA | NA | NA | NA | NA | NA | NA | NA | NA | NA | NA | NA | NA | NA | NA | 9 | NA | NA | NA | NA | NA | NA | NA | NA | NA | NA | NA | NA | NA | NA | NA | NA | NA | NA | NA | NA | NA |
| R. R. A. El-Lakany (133) | 2023 | Egypt | D | L | 0 | 0 | NA | NA | 50 | 63 | 0 | NA | NA | NA | 0 | NA | NA | NA | NA | NA | 77 | NA | NA | NA | NA | NA | NA | NA | NA | NA | NA | NA | 11 | NA | NA | NA | NA | NA | NA | NA | NA | NA | NA | NA | NA | NA |
| M. I. B. Renteria (134) | 2014 | NA | M | L | 0 | 0 | NA | NA | NA | NA | 0 | 0 | NA | NA | NA | 0 | NA | NA | NA | NA | NA | NA | NA | NA | 0 | NA | 0 | NA | 0 | NA | NA | NA | NA | NA | NA | NA | NA | NA | NA | NA | NA | NA | NA | NA | NA | NA |
| D. J. A. Biedenbach (135) | 2015 | United States | M | L | 0 | NA | NA | NA | 202 | 382 | NA | 0 | NA | NA | NA | NA | NA | NA | NA | NA | 691 | NA | NA | NA | 7 | NA | 0 | 1 | NA | NA | NA | NA | NA | NA | NA | NA | NA | NA | NA | NA | NA | NA | NA | NA | NA | NA |
| J. A. W. Karlowsky (136) | 2017 | NA | M | L | NA | NA | NA | NA | 49 | NA | 0 | NA | NA | NA | NA | NA | NA | NA | 73 | NA | NA | NA | NA | NA | NA | NA | 0 | 0 | 0 | NA | NA | NA | NA | NA | NA | NA | NA | NA | NA | NA | NA | NA | NA | NA | NA | NA |
| J. A. W. Karlowsky (137) | 2014 | NA | M | L | NA | NA | NA | NA | 25 | NA | 0 | NA | NA | NA | NA | NA | NA | NA | 40 | NA | NA | NA | NA | NA | NA | NA | 0 | 0 | 0 | NA | NA | NA | NA | NA | NA | NA | NA | NA | NA | NA | NA | NA | NA | NA | NA | NA |
| D. S. Pierard (138) | 2021 | NA | M | L | 0 | NA | NA | NA | 13 | 21 | 0 | NA | NA | NA | NA | NA | NA | NA | NA | NA | NA | NA | NA | NA | 5 | NA | 0 | NA | 0 | NA | NA | NA | NA | NA | NA | NA | NA | NA | NA | NA | NA | NA | NA | NA | NA | NA |
| D. S. Pierard (138) | 2021 | NA | M | L | 0 | NA | NA | NA | 20 | 26 | 0 | NA | NA | NA | NA | NA | NA | NA | NA | NA | NA | NA | NA | NA | 4 | NA | 0 | NA | 0 | NA | NA | NA | NA | NA | NA | NA | NA | NA | NA | NA | NA | NA | NA | NA | NA | NA |
| D. S. Pierard (138) | 2021 | NA | M | L | 0 | NA | NA | NA | 120 | 146 | 11 | NA | NA | NA | NA | NA | NA | NA | NA | NA | NA | NA | NA | NA | 0 | NA | 0 | NA | 0 | NA | NA | NA | NA | NA | NA | NA | NA | NA | NA | NA | NA | NA | NA | NA | NA | NA |
| D. S. Pierard (138) | 2021 | NA | M | L | 0 | NA | NA | NA | 19 | 33 | 0 | NA | NA | NA | NA | NA | NA | NA | NA | NA | NA | NA | NA | NA | 7 | NA | 0 | NA | 0 | NA | NA | NA | NA | NA | NA | NA | NA | NA | NA | NA | NA | NA | NA | NA | NA | NA |
| J. A. B. Karlowsky (139) | 2016 | NA | M | L | 0 | NA | NA | NA | 3 | 4 | 0 | NA | NA | NA | NA | NA | NA | NA | NA | NA | NA | NA | NA | NA | 2 | NA | 0 | 0 | 0 | NA | 0 | NA | NA | NA | NA | NA | NA | NA | NA | NA | NA | NA | NA | NA | NA | NA |
| N. V. Mohamed (140) | 2023 | NA | M | L | 0 | NA | NA | NA | 9 | 17 | 0 | NA | NA | NA | NA | NA | NA | NA | NA | NA | NA | NA | NA | NA | 3 | NA | 0 | NA | 0 | NA | NA | NA | NA | NA | NA | NA | NA | NA | NA | NA | NA | NA | NA | NA | NA | NA |
| N. V. Mohamed (140) | 2023 | NA | NA | L | 0 | NA | NA | NA | 24 | 32 | 0 | NA | NA | NA | NA | NA | NA | NA | NA | NA | NA | NA | NA | NA | 10 | NA | 0 | NA | 0 | NA | NA | NA | NA | NA | NA | NA | NA | NA | NA | NA | NA | NA | NA | NA | NA | NA |
| N. V. Mohamed (140) | 2023 | NA | NA | L | 0 | NA | NA | NA | 67 | 94 | 0 | NA | NA | NA | NA | NA | NA | NA | NA | NA | NA | NA | NA | NA | 6 | NA | 0 | NA | 0 | NA | NA | NA | NA | NA | NA | NA | NA | NA | NA | NA | NA | NA | NA | NA | NA | NA |
| N. V. Mohamed (140) | 2023 | NA | NA | L | 0 | NA | NA | NA | 11 | 14 | 0 | NA | NA | NA | NA | NA | NA | NA | NA | NA | NA | NA | NA | NA | 4 | NA | 0 | NA | 0 | NA | NA | NA | NA | NA | NA | NA | NA | NA | NA | NA | NA | NA | NA | NA | NA | NA |
| P. R. Hsueh (141) | 2015 | Taiwan | M | L | NA | NA | NA | NA | NA | NA | NA | NA | NA | NA | NA | NA | NA | NA | NA | NA | NA | NA | NA | NA | NA | NA | 0 | NA | NA | NA | NA | 0 | NA | NA | NA | NA | NA | NA | NA | NA | NA | NA | NA | NA | NA | NA |
| W. T. L. Lee (142) | 2015 | Taiwan | M | L | 0 | NA | NA | NA | 74 | 92 | 0 | NA | NA | NA | NA | 2 | NA | NA | NA | NA | 162 | NA | NA | NA | 7 | NA | NA | NA | NA | NA | NA | NA | 10 | NA | NA | NA | NA | 13 | NA | NA | NA | NA | NA | NA | NA | NA |
| S. T. Kardos (143) | 2019 | Hungary | M | L | 0 | NA | NA | NA | 34 | 40 | NA | NA | NA | NA | NA | NA | NA | NA | NA | NA | 79 | NA | NA | NA | 3 | NA | NA | NA | NA | NA | NA | NA | NA | 3 | NA | NA | NA | NA | NA | NA | NA | NA | NA | NA | NA | NA |
| M. M. Emanei (144)ni | 2014 | NA | D | S | 0 | NA | NA | NA | 40 | 40 | NA | NA | NA | NA | NA | NA | NA | NA | NA | NA | 110 | NA | NA | NA | NA | NA | 1 | NA | NA | NA | NA | NA | NA | NA | NA | NA | 0 | 51 | NA | NA | NA | NA | NA | NA | NA | NA |
| W. Y. Girma (145) | 2020 | Ethiopia | D | L | 2 | 1 | NA | NA | 9 | 11 | NA | NA | NA | NA | NA | NA | NA | NA | NA | NA | NA | NA | NA | NA | NA | 22 | NA | NA | NA | NA | NA | NA | NA | NA | NA | NA | NA | NA | NA | NA | NA | NA | NA | NA | NA | NA |
| K. E. S. Proudmore (146) | 2023 | Ethiopia | D | S | 0 | NA | NA | NA | 34 | 45 | 0 | NA | NA | NA | NA | NA | NA | NA | NA | NA | NA | NA | NA | NA | NA | NA | NA | NA | NA | NA | NA | NA | NA | NA | NA | NA | NA | NA | NA | NA | NA | NA | NA | NA | NA | NA |
| A. M. K. Mohamed (147) | 2020 | Saudi Arabia | D | S | 0 | 0 | 0 | NA | 9 | 10 | 0 | NA | NA | NA | NA | NA | NA | NA | NA | NA | NA | NA | NA | NA | 2 | NA | 0 | 0 | NA | NA | NA | NA | NA | NA | NA | NA | NA | NA | NA | NA | NA | NA | NA | NA | NA | NA |
| R. O. A. Biobaku Oluwafunmilola (148) | 2017 | Nigeria | D | L | 38 | 33 | NA | NA | 192 | 77 | 132 | NA | NA | NA | 143 | NA | NA | NA | NA | NA | NA | NA | NA | NA | NA | NA | NA | NA | NA | NA | NA | NA | NA | NA | NA | NA | NA | NA | NA | NA | NA | NA | NA | NA | NA | NA |
| M. D. Gogoi (149) | 2021 | India | D | L | 0 | 0 | NA | NA | 12 | 5 | 1 | NA | NA | NA | 2 | NA | NA | NA | NA | NA | NA | NA | NA | 1.0 | NA | NA | NA | NA | NA | NA | NA | NA | NA | NA | NA | NA | NA | NA | NA | NA | NA | NA | NA | NA | NA | NA |
| G. N. K. Dilrukshi (150) | 2021 | Sri Lanka | M | S | 0 | 0 | NA | NA | 1 | 1 | 0 | NA | NA | NA | 0 | NA | NA | NA | NA | NA | NA | NA | NA | NA | NA | NA | NA | NA | NA | NA | NA | NA | NA | NA | NA | NA | NA | NA | NA | NA | NA | NA | NA | NA | NA | NA |
| Y. J. Dong (151) | 2017 | NA | M | S | 0 | NA | NA | 0 | 21 | 22 | 0 | 1 | NA | NA | 1 | 0 | NA | NA | NA | NA | NA | NA | NA | NA | 5 | NA | NA | NA | 0 | NA | NA | NA | NA | NA | NA | NA | NA | NA | NA | NA | NA | NA | NA | NA | NA | NA |
| B. C. A. Iweriebor (152) | 2023 | NA | D | L | 5 | NA | NA | NA | 49 | 49 | 4 | NA | NA | 12.0 | 21 | NA | 12 | NA | NA | NA | 49 | NA | NA | NA | NA | 25 | NA | NA | NA | NA | NA | NA | NA | NA | NA | NA | NA | 20 | NA | 15 | NA | NA | NA | NA | NA | 23 |
| Y. G. Leykun (153) | 2021 | NA | D | S | 8 | 7 | NA | NA | 25 | 19 | 3 | 18 | NA | NA | NA | NA | NA | NA | NA | NA | 62 | NA | NA | NA | NA | NA | NA | NA | NA | NA | NA | NA | NA | NA | NA | NA | NA | NA | NA | NA | NA | NA | NA | NA | NA | NA |
| A. G. Kumalo (154) | 2023 | Ethiopia | D | L | 5 | 7 | NA | NA | NA | 10 | 11 | 7 | NA | NA | NA | NA | NA | NA | NA | NA | 59 | NA | NA | 37.0 | NA | NA | NA | NA | NA | NA | NA | NA | NA | NA | NA | NA | NA | 0 | NA | NA | NA | NA | NA | NA | NA | NA |
| E. S. M. Bjornsdottir (155) | 2019 | Iceland | NA | L | 0 | NA | NA | NA | 1 | 9 | 0 | NA | NA | NA | NA | NA | NA | NA | NA | NA | NA | NA | NA | NA | 0 | 0 | NA | NA | NA | NA | NA | NA | NA | NA | NA | NA | NA | 0 | NA | NA | NA | NA | NA | NA | NA | NA |
| R. D. Baldan (156) | 2021 | NA | M | L | 0 | NA | NA | NA | 8 | 8 | NA | NA | NA | NA | NA | NA | NA | NA | NA | NA | NA | NA | NA | NA | NA | NA | NA | NA | NA | NA | NA | NA | NA | NA | NA | NA | NA | NA | NA | NA | NA | NA | NA | NA | NA | NA |
| L. M. J. Warrier (157) | 2022 | India | D | S | 0 | 0 | NA | NA | 8 | NA | 0 | NA | NA | NA | 0 | NA | NA | NA | NA | NA | NA | NA | NA | NA | NA | NA | NA | NA | NA | NA | NA | NA | NA | NA | NA | NA | NA | NA | NA | NA | NA | NA | NA | NA | NA | NA |
| S. S. Wataradee (158) | 2023 | Thailand | M | L | 0 | 0 | NA | NA | NA | 1 | NA | NA | NA | NA | NA | NA | NA | NA | NA | NA | 33 | NA | NA | NA | NA | NA | NA | NA | NA | NA | NA | NA | NA | NA | 0 | NA | NA | NA | NA | NA | NA | NA | NA | NA | NA | NA |
| H. M. L. Kang (159) | 2017 | NA | M | L | 8 | NA | NA | NA | NA | 42 | NA | NA | NA | NA | NA | NA | NA | NA | NA | NA | NA | NA | NA | NA | NA | NA | NA | NA | NA | NA | NA | NA | NA | NA | NA | NA | NA | NA | NA | NA | NA | NA | NA | NA | NA | NA |
| M. M. Doumith (160) | 2017 | NA | M | H | 0 | 0 | NA | NA | NA | NA | 0 | NA | NA | NA | 0 | NA | NA | NA | NA | NA | NA | NA | NA | 7.0 | NA | 2 | NA | NA | NA | NA | NA | NA | NA | NA | NA | NA | NA | NA | NA | NA | NA | NA | NA | NA | NA | NA |
| A. N. C. Williams (161) | 2023 | Germany | D | L | 0 | NA | NA | NA | 0 | 0 | 1 | NA | NA | NA | NA | NA | NA | NA | NA | NA | NA | NA | NA | NA | NA | NA | NA | NA | NA | NA | NA | NA | NA | NA | NA | NA | NA | NA | NA | NA | NA | NA | NA | NA | NA | NA |
| T. G. Perme (162) | 2020 | Slovenia | D | L | 0 | 0 | NA | NA | 27 | 29 | 0 | NA | NA | NA | NA | NA | NA | NA | NA | NA | 148 | NA | 0 | NA | 0 | NA | NA | NA | NA | NA | NA | NA | NA | NA | NA | NA | NA | NA | NA | NA | NA | NA | NA | NA | NA | NA |
| U. B. P. Khan (163) | 2023 | NA | D | L | NA | 0 | NA | NA | 23 | 39 | 0 | NA | NA | NA | NA | NA | NA | NA | NA | NA | 174 | NA | NA | NA | 4 | NA | NA | NA | NA | NA | NA | NA | NA | NA | NA | NA | NA | 3 | NA | NA | NA | NA | NA | NA | NA | NA |
| E. R. Campisi (164) | 2016 | Taiwan | M | H | 0 | NA | NA | NA | NA | NA | 0 | 0 | NA | NA | NA | NA | NA | NA | NA | NA | NA | NA | NA | NA | 0 | 0 | NA | NA | NA | NA | NA | NA | NA | NA | NA | NA | NA | NA | NA | NA | NA | NA | NA | NA | NA | NA |
| G. D. C. R.-M. Palacios-Saucedo (165) | 2022 | Mexico | D | L | NA | 0 | NA | NA | 0 | 0 | 0 | NA | NA | NA | NA | NA | NA | NA | NA | NA | 11 | NA | NA | NA | 0 | NA | 0 | NA | 0 | NA | NA | NA | NA | 0 | NA | NA | NA | NA | NA | NA | NA | NA | NA | NA | NA | NA |
| Y. W. Zhou (166) | 2022 | Taiwan | M | L | 0 | 0 | NA | NA | NA | NA | NA | NA | NA | NA | NA | NA | NA | NA | NA | NA | NA | NA | NA | NA | NA | NA | NA | NA | NA | NA | NA | NA | NA | NA | NA | NA | NA | NA | NA | NA | NA | NA | NA | NA | NA | NA |
| Y. W. Zhou (166) | 2022 | Taiwan | Other | L | 0 | 0 | NA | NA | 112 | 114 | 0 | 0 | NA | 0.0 | NA | NA | NA | NA | NA | NA | 226 | NA | NA | NA | NA | NA | NA | NA | NA | NA | NA | NA | NA | NA | NA | 0 | NA | NA | NA | NA | NA | NA | NA | NA | NA | NA |
| G. I. Gherardi (167) | 2014 | Italy | M | H | NA | NA | NA | NA | NA | 17 | NA | NA | NA | NA | NA | NA | NA | NA | NA | NA | NA | NA | NA | NA | NA | NA | NA | NA | NA | NA | NA | NA | NA | NA | NA | NA | NA | NA | NA | NA | NA | NA | NA | NA | NA | NA |
| O. H. M. A. Feuerschuette (168) | 2022 | Brazil | D | L | 0 | NA | NA | NA | 54 | 92 | 0 | NA | NA | NA | NA | NA | NA | NA | NA | NA | 257 | NA | NA | NA | NA | NA | NA | NA | NA | NA | NA | NA | NA | NA | NA | NA | NA | NA | NA | NA | NA | NA | NA | NA | NA | NA |
| H. R. Saffar (169) | 2016 | NA | D | L | NA | NA | NA | NA | 1 | NA | NA | NA | NA | NA | NA | NA | NA | NA | NA | NA | NA | NA | NA | NA | NA | NA | NA | NA | NA | NA | NA | NA | NA | NA | NA | NA | NA | NA | NA | NA | NA | NA | NA | NA | NA | NA |
| S. G. Hadavand (170) | 2015 | Iran | D | L | 0 | 0 | NA | NA | NA | NA | NA | NA | NA | NA | NA | NA | NA | NA | NA | NA | 7 | NA | 7 | 0.0 | NA | NA | NA | NA | NA | 0 | NA | NA | NA | NA | NA | NA | NA | NA | NA | 0 | NA | NA | NA | NA | NA | NA |
| C. G. S. Carvalhaes (171) | 2022 | United States | M | L | NA | NA | NA | NA | 755 | NA | NA | NA | NA | NA | NA | NA | NA | NA | NA | NA | NA | NA | NA | NA | NA | NA | 0 | 0 | NA | NA | NA | 0 | NA | NA | NA | NA | NA | NA | NA | NA | NA | NA | NA | NA | NA | NA |
| C. G. S. Carvalhaes (171) | 2022 | United States | M | L | NA | NA | NA | NA | 755 | NA | NA | NA | NA | NA | NA | NA | NA | NA | NA | NA | NA | NA | NA | NA | NA | NA | 0 | 0 | NA | NA | NA | 0 | NA | NA | NA | NA | NA | NA | NA | NA | NA | NA | NA | NA | NA | NA |
| M. M. W. Ali (172) | 2020 | Ethiopia | M | L | NA | 0 | NA | NA | 5 | 4 | 0 | 0 | NA | NA | NA | NA | NA | NA | NA | NA | 73 | NA | NA | NA | 4 | NA | 0 | 3 | NA | NA | NA | NA | NA | NA | NA | NA | NA | NA | NA | NA | NA | NA | NA | NA | NA | NA |
| M. T. Abrok (173) | 2020 | Switzerland | D | L | 0 | NA | NA | NA | 1230 | 1239 | 0 | NA | NA | 0.0 | NA | NA | NA | NA | NA | NA | NA | NA | 0 | NA | NA | NA | NA | NA | NA | NA | NA | NA | NA | NA | NA | NA | NA | NA | NA | NA | NA | NA | NA | NA | NA | NA |
| T. C. Ikebe (174) | 2015 | Japan | M | L | 0 | 0 | NA | 0 | 1 | 3 | NA | NA | NA | NA | 0 | NA | 0 | NA | NA | NA | NA | NA | NA | NA | NA | NA | 0 | NA | NA | NA | NA | NA | NA | NA | NA | NA | NA | NA | NA | NA | NA | NA | NA | NA | NA | NA |
| H. Z. Shen (175) | 2019 | NA | D | L | 0 | 0 | NA | NA | 9 | NA | 0 | NA | NA | NA | NA | NA | NA | NA | NA | NA | 9 | NA | NA | 0.0 | 0 | NA | NA | NA | NA | NA | NA | NA | NA | NA | NA | NA | NA | NA | NA | NA | NA | NA | NA | NA | NA | NA |
| S. N. Jones (176) | 2022 | NA | D | L | 0 | NA | NA | NA | 32 | 32 | 0 | 2 | NA | NA | NA | NA | NA | NA | NA | NA | 77 | NA | 0 | NA | 4 | NA | NA | NA | NA | NA | NA | NA | NA | 1 | NA | NA | NA | 0 | NA | NA | NA | NA | NA | NA | NA | NA |
| X. S. M. Guan (177) | 2018 | Taiwan | D | L | 25 | NA | NA | NA | 35 | 39 | 0 | 0 | NA | NA | NA | NA | NA | NA | NA | NA | 65 | NA | NA | NA | NA | NA | 0 | NA | NA | NA | NA | NA | NA | NA | NA | NA | NA | NA | NA | NA | NA | NA | NA | 4 | NA | NA |
| A. H. Alhhazmi (178) | 2016 | Canada | D | L | 0 | NA | NA | NA | 24 | 27 | 23 | NA | NA | NA | NA | NA | NA | NA | NA | NA | NA | NA | NA | NA | NA | NA | NA | NA | NA | NA | NA | NA | NA | NA | NA | NA | NA | 26 | NA | NA | NA | NA | NA | NA | NA | NA |
| X. L. Zhang (179) | 2022 | NA | D | L | 0 | NA | NA | NA | 36 | 40 | 0 | NA | NA | NA | NA | NA | NA | NA | NA | NA | 33 | NA | NA | NA | 15 | NA | 0 | NA | NA | NA | NA | NA | NA | NA | NA | NA | 3 | NA | NA | NA | NA | NA | NA | NA | NA | NA |
| C. D. Florindo (180) | 2014 | Portugal | M | L | NA | NA | NA | NA | NA | 43 | NA | NA | NA | NA | NA | NA | NA | NA | NA | NA | NA | NA | NA | NA | NA | NA | NA | NA | NA | NA | NA | NA | NA | NA | NA | NA | NA | NA | NA | NA | NA | NA | NA | NA | NA | NA |
| C. D. Florindo (180) | 2014 | Portugal | M | L | NA | NA | NA | NA | 24 | NA | NA | NA | NA | NA | NA | NA | NA | NA | NA | NA | NA | NA | NA | NA | NA | NA | NA | NA | NA | NA | NA | NA | NA | NA | NA | NA | NA | NA | NA | NA | NA | NA | NA | NA | NA | NA |
| A. T. Ma (181) | 2021 | Canada | D | L | 0 | NA | NA | NA | 702 | 778 | 0 | NA | NA | NA | NA | NA | NA | NA | NA | NA | NA | NA | NA | NA | NA | NA | NA | NA | NA | NA | NA | NA | NA | NA | NA | NA | NA | 4 | NA | NA | NA | NA | NA | NA | NA | NA |
| M. D. P. C.-R. Crespo-Ortiz (182) | 2014 | Colombia | M | L | NA | NA | NA | NA | 16 | 60 | 4 | NA | NA | NA | NA | NA | NA | NA | NA | NA | 593 | NA | NA | NA | 17 | NA | 0 | NA | NA | NA | NA | NA | NA | NA | NA | NA | NA | NA | NA | NA | NA | NA | NA | NA | NA | NA |
| Y. T. Kao (183) | 2019 | Taiwan | D | L | 0 | 0 | NA | NA | 117 | 124 | 0 | NA | NA | NA | 0 | NA | NA | NA | NA | NA | NA | NA | NA | NA | NA | NA | NA | NA | NA | NA | NA | NA | NA | NA | NA | NA | NA | NA | NA | NA | NA | NA | NA | NA | NA | NA |
| K. C. Hayes (184) | 2017 | Ireland | D | L | 0 | NA | NA | NA | 48 | 50 | NA | NA | NA | NA | NA | NA | NA | NA | NA | NA | 205 | NA | NA | NA | NA | NA | NA | NA | NA | NA | NA | NA | NA | NA | NA | NA | NA | NA | NA | NA | NA | NA | NA | NA | NA | NA |
| S. A. Teatero (185) | 2015 | NA | M | L | 0 | 0 | NA | NA | 77 | 77 | 0 | NA | NA | NA | 0 | NA | NA | NA | NA | NA | 71 | NA | NA | NA | NA | NA | NA | NA | NA | NA | NA | NA | NA | NA | NA | NA | NA | NA | NA | NA | NA | NA | NA | NA | NA | NA |
| O. P. Tulyaprawat (186) | 2021 | Thailand | M | L | 1 | NA | NA | NA | 24 | 24 | 0 | 0 | NA | NA | 0 | 0 | NA | 24 | NA | NA | 45 | NA | NA | NA | 2 | NA | 0 | 0 | NA | NA | NA | NA | 0 | NA | NA | NA | NA | 2 | NA | NA | NA | NA | NA | NA | NA | NA |
| L. K. Zhang (187) | 2021 | NA | D | L | 0 | NA | NA | NA | 7 | 19 | 0 | NA | NA | 0.0 | 0 | NA | NA | NA | NA | NA | 7 | NA | NA | NA | 17 | NA | NA | NA | NA | NA | 0 | NA | 0 | NA | NA | NA | NA | NA | NA | NA | NA | NA | NA | NA | NA | NA |
| Y. D. Guo (188) | 2018 | China | D | L | 0 | NA | NA | NA | 12 | 17 | 0 | 0 | NA | NA | NA | NA | NA | NA | NA | NA | 20 | NA | NA | NA | NA | NA | 0 | NA | 0 | NA | NA | NA | NA | NA | NA | NA | 0 | NA | NA | NA | NA | NA | NA | NA | NA | NA |
| M. K. Nabavinia  (189) | 2020 | Iran | D | L | 6 | NA | NA | NA | 12 | 8 | NA | NA | NA | NA | NA | NA | NA | NA | NA | NA | 55 | NA | NA | NA | 9 | 17 | NA | NA | NA | NA | NA | NA | NA | NA | NA | NA | NA | NA | NA | NA | NA | NA | NA | NA | NA | NA |
| X. M. D. Li (190) | 2022 | NA | M | L | 0 | 0 | NA | NA | 9 | NA | 0 | NA | NA | NA | NA | NA | NA | NA | NA | 9 | 9 | NA | NA | 3.0 | 3 | NA | 0 | 0 | 0 | NA | NA | NA | NA | 8 | NA | NA | 0 | NA | NA | NA | NA | NA | NA | NA | NA | NA |
| N. A. E. R. Fahim (191) | 2022 | Egypt | D | L | 13 | NA | NA | NA | 14 | 13 | 14 | NA | NA | NA | 19 | NA | NA | NA | NA | NA | NA | NA | NA | NA | NA | NA | NA | NA | NA | NA | NA | NA | NA | NA | NA | NA | NA | NA | NA | NA | NA | NA | NA | NA | NA | NA |
| A. A. E. M. El Shahaway (192) | 2019 | NA | D | L | 0 | 0 | NA | NA | 6 | 10 | 0 | 0 | NA | NA | NA | NA | NA | NA | NA | NA | NA | NA | NA | NA | NA | NA | NA | NA | NA | NA | NA | NA | NA | NA | NA | NA | NA | NA | NA | NA | NA | NA | NA | NA | NA | NA |
| S. H. Jalalifar (193) | 2019 | NA | Other | L | 8 | NA | NA | NA | 47 | 52 | 1 | 8 | NA | NA | 8 | NA | NA | NA | NA | NA | NA | NA | NA | NA | 9 | NA | NA | NA | NA | NA | NA | NA | 8 | NA | NA | NA | NA | NA | NA | NA | NA | NA | NA | NA | NA | NA |
| S. K. Panahi (194) | 2023 | Iran | D | L | NA | NA | NA | NA | 8 | 7 | 6 | 0 | NA | NA | 0 | NA | NA | NA | NA | NA | 33 | NA | NA | NA | 5 | NA | 2 | NA | NA | NA | NA | NA | 1 | NA | NA | NA | NA | NA | NA | NA | NA | NA | NA | NA | NA | NA |
| T. F. Motallebirad (195) | 2021 | NA | D | L | 0 | NA | NA | NA | 42 | NA | 0 | 2 | NA | NA | 0 | NA | NA | NA | NA | 51 | 122 | NA | NA | NA | 15 | NA | NA | NA | NA | NA | NA | NA | 2 | NA | NA | NA | NA | NA | NA | NA | NA | NA | NA | NA | NA | NA |
| L. R. S. Burcham (196) | 2019 | United States | D | L | 11 | NA | NA | NA | 24 | 34 | 24 | NA | NA | NA | NA | NA | NA | NA | NA | NA | 74 | NA | NA | NA | NA | NA | NA | NA | NA | NA | NA | NA | NA | NA | NA | NA | NA | NA | NA | NA | NA | NA | NA | NA | NA | NA |
| K. E. Evangelia (197) | 2015 | France | D | L | 0 | NA | NA | NA | 8 | 11 | 0 | NA | NA | NA | NA | NA | NA | NA | NA | NA | 112 | NA | NA | NA | 0 | NA | NA | NA | NA | NA | NA | NA | NA | NA | NA | NA | NA | NA | NA | NA | NA | NA | NA | NA | NA | NA |
| M. A. M. Malita (198) | 2023 | NA | D | L | 0 | 0 | 0 | NA | 0 | 0 | 1 | NA | NA | 6.0 | NA | NA | NA | NA | NA | NA | 0 | NA | NA | NA | NA | 0 | 0 | NA | NA | NA | NA | NA | NA | NA | NA | NA | NA | 3 | NA | NA | NA | NA | NA | NA | NA | NA |
| G. G. Goudarzi (199) | 2015 | Iran | D | L | 1 | NA | NA | NA | 22 | 22 | 0 | NA | NA | NA | NA | NA | NA | NA | NA | NA | NA | NA | NA | NA | NA | NA | NA | NA | NA | NA | NA | NA | NA | NA | NA | NA | NA | NA | NA | NA | NA | NA | NA | NA | NA | NA |
| S. K. Asghar (200) | 2020 | Pakistan | D | L | 0 | 1 | NA | NA | 4 | 5 | 1 | NA | NA | 0.0 | NA | NA | NA | NA | NA | NA | 1 | NA | NA | 2.0 | NA | NA | 1 | NA | NA | NA | NA | NA | NA | NA | NA | NA | NA | 0 | NA | NA | NA | NA | NA | NA | NA | NA |
| E. M. A. J. Felemban (201) | 2019 | Saudi Arabia | D | L | 0 | 0 | NA | NA | 4 | 10 | 3 | NA | NA | NA | 0 | 0 | NA | NA | NA | NA | 12 | NA | NA | NA | 0 | NA | 0 | 0 | NA | NA | NA | NA | 0 | NA | NA | NA | NA | 0 | NA | NA | NA | NA | NA | NA | NA | NA |
| A. K. D. Brigtsen (202) | 2015 | NA | D | L | 0 | NA | NA | NA | 43 | 46 | NA | NA | NA | NA | NA | NA | NA | NA | NA | NA | 344 | NA | 4 | NA | NA | NA | NA | NA | NA | NA | NA | NA | NA | NA | NA | NA | NA | NA | NA | NA | NA | NA | NA | NA | NA | NA |
| W. Y. Mulu (203) | 2015 | NA | D | L | NA | 8 | NA | NA | 2 | 2 | NA | NA | 8 | NA | NA | NA | NA | NA | NA | NA | 6 | NA | 8 | 2.0 | NA | 2 | NA | NA | NA | NA | NA | NA | NA | NA | NA | NA | NA | NA | NA | 2 | NA | NA | NA | NA | NA | NA |
| W. Z. Ji (204) | 2017 | Taiwan | D | L | 0 | NA | NA | NA | 333 | 413 | 0 | 0 | NA | NA | NA | NA | NA | NA | NA | NA | NA | NA | NA | NA | 165 | NA | 0 | NA | NA | NA | NA | NA | NA | NA | NA | NA | NA | NA | NA | NA | NA | NA | NA | NA | NA | NA |
| P. E. H. Akpaka (205) | 2022 | Trinidad & Tobago | D | L | NA | 47 | NA | NA | 7 | 22 | 0 | 4 | 27 | 4.0 | NA | 0 | 5 | NA | NA | NA | NA | NA | 42 | 6.0 | 6 | NA | NA | NA | NA | 33 | NA | NA | NA | NA | NA | NA | NA | NA | NA | 7 | NA | NA | NA | NA | NA | NA |
| D. N. L. Sapugahawatte (206) | 2022 | NA | M | L | 0 | NA | NA | NA | 0 | 9 | 0 | NA | NA | NA | 0 | NA | NA | NA | NA | NA | 35 | 34 | NA | 0.0 | 0 | 60 | 0 | NA | NA | NA | NA | NA | NA | 0 | 0 | NA | NA | 0 | NA | NA | NA | NA | NA | NA | NA | NA |
| J. F. C. Hsu (207) | 2023 | Taiwan | D | L | 0 | NA | NA | NA | 92 | 93 | 0 | NA | NA | NA | 0 | NA | NA | NA | NA | NA | NA | NA | NA | NA | NA | NA | NA | NA | NA | NA | NA | NA | NA | NA | NA | 0 | NA | NA | NA | NA | NA | NA | NA | NA | NA | NA |
| I. A. J. Yoon (208) | 2015 | NA | M | L | 0 | NA | NA | NA | 31 | 29 | NA | NA | NA | NA | NA | NA | NA | NA | NA | NA | NA | NA | NA | NA | NA | NA | NA | NA | NA | NA | NA | NA | NA | NA | NA | NA | NA | NA | NA | NA | NA | NA | NA | NA | NA | NA |
| J. W. Jiao (209) | 2022 | NA | NA | L | 0 | NA | NA | NA | 129 | 138 | 0 | NA | 0 | NA | 0 | 0 | NA | NA | NA | NA | 99 | NA | NA | NA | 112 | NA | 0 | NA | NA | NA | NA | NA | 0 | NA | NA | NA | NA | 0 | NA | NA | NA | NA | NA | NA | NA | NA |
| S. T. S. D. Alzuheiri (210) | 2021 | United Arab Emirates | Other | L | 15 | 7 | NA | NA | 91 | 90 | 4 | 6 | NA | NA | 6 | NA | NA | NA | NA | NA | NA | NA | 122 | NA | 16 | NA | 4 | NA | NA | NA | NA | NA | NA | NA | NA | NA | NA | NA | NA | NA | NA | NA | NA | NA | NA | NA |
| X. D. Li (211) | 2019 | NA | D | L | NA | NA | NA | NA | NA | NA | NA | NA | NA | NA | NA | NA | NA | NA | NA | NA | NA | NA | NA | NA | NA | NA | NA | NA | 0 | NA | NA | NA | NA | NA | NA | NA | NA | NA | NA | NA | NA | NA | NA | NA | NA | NA |
| X. D. Li (211) | 2019 | NA | D | L | NA | NA | NA | NA | NA | NA | NA | 0 | NA | 0.0 | NA | NA | NA | NA | NA | NA | NA | NA | NA | NA | NA | NA | 0 | NA | NA | NA | NA | NA | NA | 2 | NA | NA | NA | NA | NA | NA | NA | NA | NA | NA | NA | NA |
| X. D. Li (211) | 2019 | NA | D | L | 0 | NA | NA | NA | 14 | 17 | 0 | NA | NA | NA | 1 | NA | NA | NA | NA | NA | NA | NA | NA | NA | 2 | NA | NA | NA | NA | NA | NA | NA | NA | NA | NA | NA | NA | NA | NA | NA | NA | NA | NA | NA | NA | NA |
| N. K. Hirai (212) | 2020 | Japan | M | L | 0 | 0 | NA | 0 | 2 | 7 | 0 | 0 | NA | NA | NA | 0 | NA | 6 | 4 | NA | NA | NA | NA | NA | NA | NA | NA | NA | NA | NA | NA | NA | 0 | NA | NA | NA | NA | NA | NA | NA | NA | NA | NA | NA | NA | NA |
| C. F. Li (213) | 2018 | NA | NA | L | 0 | NA | NA | NA | NA | NA | 0 | NA | NA | NA | NA | 0 | NA | NA | NA | NA | NA | NA | NA | NA | 0 | NA | 0 | NA | NA | NA | NA | NA | NA | NA | NA | NA | NA | NA | NA | NA | NA | NA | NA | NA | NA | NA |
| C. F. Li (213) | 2018 | NA | NA | S | NA | NA | NA | NA | NA | NA | NA | 0 | NA | NA | NA | NA | NA | NA | NA | NA | NA | NA | NA | NA | NA | NA | NA | NA | NA | NA | NA | NA | NA | NA | NA | NA | NA | NA | NA | NA | NA | NA | NA | NA | NA | NA |
| C. F. Li (213) | 2018 | NA | NA | S | NA | NA | NA | NA | NA | NA | NA | NA | NA | NA | NA | NA | NA | NA | NA | NA | NA | NA | NA | NA | 6 | NA | NA | NA | NA | NA | NA | NA | 0 | NA | NA | NA | NA | NA | NA | NA | NA | NA | NA | NA | NA | NA |
| C. F. Li (213) | 2018 | NA | NA | S | NA | NA | NA | NA | NA | NA | NA | NA | NA | NA | NA | NA | NA | NA | NA | NA | 10 | NA | NA | NA | NA | NA | NA | NA | NA | NA | NA | NA | NA | NA | NA | NA | NA | NA | NA | NA | NA | NA | NA | NA | NA | NA |
| C. F. Li (213) | 2018 | NA | NA | S | NA | NA | NA | NA | NA | NA | NA | NA | NA | NA | NA | 0 | NA | NA | NA | NA | NA | NA | NA | NA | NA | NA | NA | NA | NA | NA | NA | NA | NA | NA | NA | NA | NA | NA | NA | NA | NA | NA | NA | NA | NA | NA |
| C. F. Li (213) | 2018 | NA | NA | S | NA | NA | NA | NA | NA | NA | NA | NA | NA | NA | 0 | NA | NA | NA | NA | NA | NA | NA | NA | NA | NA | NA | NA | NA | NA | NA | NA | NA | NA | NA | NA | NA | NA | NA | NA | NA | NA | NA | NA | NA | NA | NA |
| C. F. Li (213) | 2018 | NA | NA | S | NA | NA | NA | NA | NA | NA | NA | NA | NA | NA | NA | NA | NA | NA | NA | NA | NA | NA | NA | 4.0 | NA | NA | NA | NA | NA | NA | NA | NA | NA | NA | NA | NA | NA | NA | NA | NA | NA | NA | NA | NA | NA | NA |
| L. Y. Z. Guo (214) | 2016 | NA | M | L | 0 | NA | NA | NA | NA | NA | 0 | NA | NA | NA | NA | NA | NA | NA | NA | NA | NA | NA | NA | NA | NA | NA | NA | NA | NA | NA | NA | NA | NA | NA | NA | NA | NA | NA | NA | NA | NA | NA | NA | NA | NA | NA |
| L. Y. Z. Guo (214) | 2016 | NA | M | L | NA | NA | NA | NA | NA | NA | NA | 0 | NA | NA | NA | NA | NA | NA | NA | NA | NA | NA | NA | NA | NA | NA | NA | NA | NA | NA | NA | NA | NA | NA | NA | NA | NA | NA | NA | NA | NA | NA | NA | NA | NA | NA |
| L. Y. Z. Guo (214) | 2016 | NA | M | L | NA | NA | NA | NA | NA | NA | NA | NA | NA | NA | NA | NA | NA | NA | NA | NA | NA | NA | NA | NA | NA | NA | NA | NA | NA | NA | NA | NA | 0 | NA | NA | NA | NA | NA | NA | NA | NA | NA | NA | NA | NA | NA |
| H. K. Lee (215) | 2022 | Germany | M | L | 0 | NA | NA | NA | 40 | 36 | NA | NA | NA | NA | NA | NA | NA | NA | NA | NA | NA | NA | NA | NA | 53 | NA | NA | NA | NA | NA | NA | NA | NA | NA | NA | NA | NA | NA | NA | NA | NA | NA | NA | NA | NA | NA |
| P. B. Mathur (216) | 2014 | India | D | S | NA | NA | NA | NA | NA | 1 | NA | NA | NA | NA | NA | NA | NA | NA | NA | NA | 3 | NA | NA | 1.0 | NA | NA | NA | NA | NA | NA | NA | NA | NA | NA | NA | NA | NA | 0 | NA | NA | NA | NA | NA | NA | NA | NA |
| S. P. Kerneis (217) | 2017 | NA | D | L | NA | NA | NA | NA | NA | 55 | NA | NA | NA | NA | NA | NA | NA | NA | NA | NA | 132 | NA | NA | NA | NA | NA | NA | NA | NA | NA | NA | NA | NA | NA | NA | NA | NA | NA | NA | NA | NA | NA | NA | NA | NA | NA |
| Y. H. C. Wang (218) | 2014 | Taiwan | D | L | 0 | NA | NA | NA | 227 | 229 | NA | NA | NA | NA | NA | NA | NA | NA | NA | NA | NA | NA | NA | NA | NA | NA | NA | NA | NA | NA | NA | NA | NA | NA | NA | NA | NA | NA | NA | NA | NA | NA | NA | NA | NA | NA |
| J. F. L. Hsu (219) | 2023 | Taiwan | D | L | 0 | 0 | NA | NA | 133 | 136 | 0 | NA | NA | NA | 0 | NA | NA | NA | NA | NA | NA | NA | NA | NA | NA | NA | NA | NA | NA | NA | NA | NA | NA | NA | NA | 0 | NA | NA | NA | NA | NA | NA | NA | NA | NA | NA |
| S. J. T. Zeng (220) | 2016 | NA | NA | S | 0 | 0 | NA | NA | 26 | NA | 0 | NA | NA | NA | NA | NA | NA | NA | NA | NA | 28 | NA | NA | 4.0 | 4 | NA | 0 | NA | 0 | NA | NA | NA | NA | NA | NA | NA | NA | NA | NA | NA | NA | NA | NA | NA | NA | NA |
| S. J. T. Zeng (220) | 2016 | NA | NA | S | NA | NA | NA | NA | NA | NA | NA | NA | NA | NA | NA | NA | NA | NA | NA | NA | NA | NA | NA | NA | NA | NA | NA | NA | NA | NA | NA | NA | NA | 2 | NA | NA | NA | NA | NA | NA | NA | NA | NA | NA | NA | NA |
| M. J. Emaneini (221) | 2016 | NA | D | L | 0 | NA | NA | NA | 6 | 5 | 0 | NA | NA | NA | NA | NA | NA | NA | NA | NA | NA | NA | NA | NA | NA | NA | 0 | NA | NA | NA | NA | NA | NA | NA | NA | NA | 0 | NA | NA | NA | NA | NA | NA | NA | NA | NA |
| Y. Y. Tang (222) | 2020 | Taiwan | NA | S | 0 | 0 | NA | NA | 52 | 60 | NA | NA | NA | NA | NA | NA | NA | NA | NA | NA | 57 | NA | NA | 44.0 | 61 | NA | NA | NA | 0 | NA | NA | NA | NA | 41 | NA | NA | 0 | NA | NA | 0 | NA | NA | NA | NA | NA | NA |
| M. E. S. D. Suhaimi (223) | 2017 | Malaysia | D | L | 1 | NA | NA | NA | 7 | 13 | NA | NA | NA | NA | NA | NA | NA | NA | NA | NA | 54 | NA | NA | NA | NA | NA | NA | NA | NA | NA | NA | NA | NA | NA | NA | NA | NA | NA | NA | NA | NA | NA | NA | NA | NA | NA |
| G. B. Piccinelli (224) | 2015 | Italy | C | L | 0 | NA | NA | NA | NA | NA | NA | 0 | NA | 0.0 | NA | NA | NA | 17 | 17 | NA | 70 | NA | NA | NA | 3 | NA | NA | NA | NA | NA | NA | NA | NA | NA | NA | NA | NA | NA | NA | NA | NA | NA | NA | NA | NA | NA |
| A. H. Al-Matary (225) | 2019 | Saudi Arabia | NA | L | 0 | NA | NA | NA | NA | NA | 0 | NA | NA | NA | NA | NA | NA | NA | NA | NA | NA | NA | NA | NA | NA | NA | NA | NA | NA | NA | NA | NA | NA | NA | NA | NA | NA | NA | NA | NA | NA | NA | NA | NA | NA | NA |
| B. W. Chang (226) | 2014 | Japan | M | L | 0 | 0 | NA | NA | 2 | 14 | NA | NA | NA | NA | 0 | NA | 0 | NA | NA | NA | NA | NA | NA | NA | NA | NA | NA | NA | NA | NA | NA | NA | NA | NA | NA | NA | NA | NA | NA | NA | NA | NA | NA | NA | NA | NA |
| C. L. Hays (227) | 2016 | France | D | L | 0 | NA | NA | NA | 2177 | 3173 | 0 | NA | 0 | NA | NA | NA | NA | NA | NA | NA | 7579 | NA | NA | NA | 66 | 24 | NA | NA | NA | NA | NA | NA | NA | NA | NA | NA | NA | NA | NA | NA | NA | NA | NA | NA | 572 | NA |
| R. K. S. Flamm (228) | 2014 | NA | M | L | 0 | NA | NA | NA | 3 | 9 | 0 | 0 | NA | NA | NA | NA | NA | NA | NA | NA | 61 | NA | NA | NA | 1 | NA | 0 | 0 | 0 | NA | 0 | NA | NA | NA | NA | NA | NA | NA | NA | NA | NA | NA | NA | NA | NA | NA |
| M. R. Navidinia (229) | 2017 | Iran | M | L | 0 | 0 | NA | NA | 149 | 127 | 0 | 20 | NA | NA | 4 | NA | NA | NA | NA | NA | 194 | NA | NA | NA | 11 | 198 | NA | NA | NA | NA | NA | NA | NA | NA | NA | NA | NA | 73 | NA | NA | NA | NA | NA | NA | NA | NA |
| O. E. Swann (230) | 2014 | Malawi | D | L | NA | NA | NA | NA | NA | NA | NA | NA | NA | NA | NA | NA | NA | NA | NA | 8 | NA | NA | NA | NA | NA | NA | NA | NA | NA | NA | NA | NA | NA | NA | NA | NA | NA | NA | NA | NA | NA | NA | NA | NA | NA | NA |
| O. E. Swann (230) | 2014 | Malawi | D | L | 1 | NA | NA | NA | NA | NA | NA | NA | NA | NA | NA | NA | NA | NA | NA | NA | NA | NA | NA | NA | NA | NA | NA | NA | NA | NA | NA | NA | NA | NA | NA | NA | NA | NA | NA | NA | NA | NA | NA | NA | NA | NA |
| O. E. Swann (230) | 2014 | Malawi | D | L | NA | NA | NA | NA | NA | NA | NA | 0 | NA | NA | NA | NA | NA | NA | NA | NA | NA | NA | NA | NA | NA | NA | NA | NA | NA | NA | NA | NA | NA | NA | NA | NA | NA | NA | NA | NA | NA | NA | NA | NA | NA | NA |
| O. E. Swann (230) | 2014 | Malawi | D | L | NA | NA | NA | NA | NA | NA | NA | NA | NA | NA | NA | NA | NA | NA | NA | NA | NA | NA | NA | NA | NA | 0 | NA | NA | NA | NA | NA | NA | NA | NA | NA | NA | NA | NA | NA | NA | NA | NA | NA | NA | NA | NA |
| Y. Z. Zhou (231) | 2023 | NA | M | L | 0 | NA | NA | NA | 113 | 139 | 0 | NA | NA | NA | NA | NA | NA | NA | NA | NA | NA | NA | NA | NA | 34 | NA | 0 | NA | NA | NA | NA | NA | NA | NA | NA | NA | NA | NA | NA | NA | NA | NA | NA | NA | NA | NA |
| M. M. Majigo (232) | 2023 | NA | D | L | 93 | 90 | NA | NA | 29 | NA | NA | 77 | NA | NA | NA | NA | NA | NA | NA | 85 | NA | NA | NA | 50.0 | NA | NA | NA | NA | NA | NA | NA | NA | NA | NA | NA | NA | NA | 10 | NA | NA | NA | NA | NA | NA | NA | NA |
| S. Ahmad (233) | 2015 | Saudi Arabia | Other | L | 42 | 44 | 2 | NA | 51 | 52 | 0 | NA | NA | NA | NA | NA | NA | NA | NA | NA | 62 | NA | 69 | NA | NA | NA | 5 | NA | NA | 20 | NA | NA | NA | NA | NA | NA | NA | NA | NA | NA | NA | NA | NA | NA | NA | NA |
| M. K. M. Mwei (234) | 2018 | Tanzania | NA | L | 0 | 0 | 0 | NA | NA | 2 | NA | NA | NA | NA | NA | NA | NA | NA | NA | NA | NA | NA | NA | NA | NA | NA | NA | NA | NA | NA | NA | NA | NA | NA | NA | NA | NA | NA | NA | NA | NA | NA | NA | NA | NA | NA |
| E. K. Foster-Nyarko (235) | 2016 | Gambia | M | L | 0 | NA | NA | NA | NA | NA | NA | NA | NA | NA | NA | NA | NA | NA | NA | NA | 119 | NA | NA | NA | NA | NA | NA | NA | NA | NA | NA | NA | NA | NA | NA | NA | NA | 4 | NA | NA | NA | NA | NA | NA | NA | NA |
| M. W. Morozumi (236) | 2014 | NA | M | L | 0 | 0 | NA | NA | NA | 0 | 0 | NA | 0 | NA | 0 | 0 | NA | NA | NA | NA | NA | NA | NA | NA | NA | NA | NA | NA | NA | NA | NA | NA | NA | NA | NA | NA | NA | NA | NA | NA | NA | NA | NA | NA | NA | NA |
| L. A. Vigliarolo (237) | 2019 | Argentina | C | L | 0 | NA | NA | NA | NA | NA | NA | NA | NA | NA | NA | NA | NA | NA | NA | NA | NA | NA | NA | NA | 24 | NA | NA | NA | NA | NA | NA | NA | NA | NA | 24 | NA | NA | NA | NA | 25 | NA | NA | NA | NA | NA | NA |
| L. A. Vigliarolo (237) | 2019 | Argentina | M | L | NA | NA | NA | NA | 24 | 42 | NA | NA | NA | NA | NA | NA | NA | NA | NA | NA | NA | NA | NA | NA | NA | NA | NA | NA | NA | NA | NA | NA | NA | NA | NA | NA | NA | NA | NA | NA | NA | NA | NA | NA | NA | NA |
| R. N. Morfin-Otero (238) | 2015 | Mexico | M | L | 0 | 0 | NA | NA | NA | NA | 0 | 0 | NA | NA | NA | 0 | NA | NA | NA | NA | 0 | NA | NA | NA | 6 | NA | 0 | NA | NA | NA | NA | NA | NA | NA | NA | NA | NA | NA | NA | NA | NA | NA | NA | NA | NA | NA |
| N. I. Eskandarian (239) | 2015 | Malaysia | M | L | 0 | 0 | NA | NA | 18 | NA | 0 | 0 | NA | 0.0 | NA | NA | NA | NA | NA | 24 | 74 | NA | NA | NA | 0 | NA | NA | NA | NA | NA | NA | NA | NA | NA | NA | NA | NA | 0 | NA | NA | NA | NA | NA | NA | NA | NA |
| F. A. Lagunas-Rangel (240) | 2018 | Mexico | D | L | NA | 3 | NA | NA | NA | NA | NA | 7 | NA | 4.0 | NA | NA | NA | NA | NA | NA | NA | NA | 13 | 10.0 | NA | 4 | NA | NA | NA | 3 | NA | NA | NA | NA | NA | NA | NA | NA | NA | NA | 1 | NA | 13 | NA | 3 | 11 |
| P. S. Mubanga (241) | 2015 | Lesotho | D | L | NA | NA | NA | NA | NA | NA | NA | NA | 2 | NA | NA | NA | NA | NA | NA | NA | NA | NA | 3 | 2.0 | NA | NA | NA | NA | NA | 2 | NA | NA | NA | NA | NA | NA | NA | NA | NA | NA | NA | NA | NA | NA | NA | NA |
| P. M. Bhola (242) | 2020 | United States | Other | L | 0 | 0 | NA | NA | NA | NA | NA | NA | NA | 27.3 | NA | NA | NA | NA | NA | NA | NA | NA | NA | 16.9 | NA | NA | NA | NA | NA | NA | NA | NA | NA | NA | NA | NA | NA | NA | NA | NA | NA | NA | NA | NA | NA | NA |
| P. M. Bhola (242) | 2020 | United States | Other | L | NA | 0 | NA | NA | NA | NA | NA | NA | 0 | NA | NA | NA | NA | NA | NA | NA | NA | NA | NA | NA | NA | NA | NA | NA | NA | NA | NA | NA | NA | NA | NA | NA | NA | NA | NA | NA | NA | NA | NA | NA | NA | NA |
| P. M. Bhola (242) | 2020 | United States | Other | L | NA | NA | NA | NA | NA | NA | 5 | NA | NA | NA | NA | NA | NA | NA | NA | NA | NA | NA | NA | NA | NA | NA | NA | NA | NA | NA | NA | NA | NA | NA | NA | NA | NA | NA | NA | NA | NA | NA | NA | NA | NA | NA |
| S. C. S. Melo (243) | 2016 | Brazil | D | L | 0 | NA | NA | NA | 11 | 11 | 0 | NA | NA | NA | 0 | NA | NA | NA | NA | 117 | NA | NA | NA | NA | 1 | NA | NA | NA | NA | NA | NA | NA | NA | NA | NA | NA | NA | 6 | NA | NA | NA | NA | NA | NA | NA | NA |
| B. M. Balkhi (244) | 2018 | Saudi Arabia | D | L | NA | 1 | NA | 0 | NA | NA | NA | NA | NA | 0.0 | NA | NA | NA | NA | NA | NA | NA | NA | 19 | 4.0 | 4 | 0 | NA | NA | NA | 0 | NA | NA | 0 | NA | NA | NA | NA | NA | NA | NA | NA | 0 | NA | NA | 0 | NA |
| M. D. Said (245) | 2020 | South Africa | M | L | 0 | NA | NA | NA | NA | 1 | 0 | NA | NA | NA | NA | NA | NA | NA | NA | NA | NA | NA | NA | NA | NA | NA | NA | NA | NA | NA | NA | NA | NA | NA | NA | NA | NA | NA | NA | NA | NA | NA | NA | NA | NA | NA |
| A. A. Mohamed (246) | 2023 | Iraq | Other | S | 7 | NA | NA | NA | NA | NA | 0 | NA | NA | NA | NA | NA | NA | NA | NA | NA | 9 | NA | 7 | NA | NA | 7 | NA | NA | NA | 7 | NA | NA | NA | NA | NA | NA | NA | NA | NA | NA | NA | NA | NA | NA | 5 | NA |
| A. M. Tesfaye (247) | 2022 | Ethiopia | D | L | 4 | 2 | NA | NA | 8 | 6 | 1 | 5 | NA | NA | NA | NA | NA | 3 | NA | NA | 10 | NA | NA | 8.0 | NA | NA | NA | NA | NA | NA | NA | NA | NA | NA | NA | NA | NA | 1 | NA | NA | NA | NA | NA | NA | NA | NA |
| C. D. C. Minotti (248) | 2023 | NA | Other | L | NA | 0 | NA | NA | NA | NA | 0 | NA | NA | NA | 0 | NA | NA | NA | NA | NA | NA | NA | NA | NA | NA | NA | NA | NA | NA | NA | NA | NA | NA | NA | NA | 0 | NA | NA | NA | NA | NA | NA | NA | NA | NA | NA |
| V. D. Van Du (249) | 2021 | Vietnam | M | L | 0 | NA | NA | NA | NA | NA | NA | NA | NA | NA | NA | NA | NA | NA | NA | NA | NA | NA | NA | NA | NA | NA | NA | NA | NA | NA | NA | NA | NA | NA | NA | NA | NA | NA | NA | NA | NA | NA | NA | NA | NA | NA |
| V. D. Van Du (249) | 2021 | Vietnam | M | L | NA | 0 | NA | NA | NA | NA | NA | NA | NA | NA | NA | NA | NA | NA | NA | NA | NA | NA | NA | NA | NA | NA | NA | NA | NA | NA | NA | NA | NA | NA | NA | NA | NA | NA | NA | NA | NA | NA | NA | NA | NA | NA |
| V. D. Van Du(249) | 2021 | Vietnam | M | S | NA | NA | NA | NA | NA | NA | NA | 0 | NA | NA | NA | NA | NA | NA | NA | NA | NA | NA | NA | NA | NA | NA | NA | NA | NA | NA | NA | NA | NA | NA | NA | NA | NA | NA | NA | NA | NA | NA | NA | NA | NA | NA |
| V. D. Van Du (249) | 2021 | Vietnam | M | L | NA | NA | NA | NA | NA | NA | NA | NA | NA | NA | 0 | NA | NA | NA | NA | NA | NA | NA | NA | NA | NA | NA | NA | NA | NA | NA | NA | NA | NA | NA | NA | NA | NA | NA | NA | NA | NA | NA | NA | NA | NA | NA |
| V. D. Van Du (249) | 2021 | Vietnam | M | L | NA | NA | NA | NA | NA | NA | 0 | NA | NA | NA | NA | NA | NA | NA | NA | NA | 234 | NA | NA | NA | NA | NA | NA | NA | NA | NA | NA | NA | NA | NA | NA | NA | NA | NA | NA | NA | NA | NA | NA | NA | NA | NA |
| V. D. Van Du (249) | 2021 | Vietnam | M | S | NA | NA | NA | NA | NA | NA | NA | NA | NA | NA | NA | NA | NA | NA | NA | NA | NA | NA | NA | NA | NA | NA | NA | NA | NA | NA | NA | NA | NA | NA | NA | NA | 0 | NA | NA | NA | NA | NA | NA | NA | NA | NA |
| V. D. Van Du (249) | 2021 | Vietnam | M | L | NA | NA | NA | NA | NA | 202 | NA | NA | NA | NA | NA | NA | NA | NA | NA | NA | NA | NA | NA | NA | NA | NA | NA | NA | NA | NA | NA | NA | NA | NA | NA | NA | NA | NA | NA | NA | NA | NA | NA | NA | NA | NA |
| V. D. Van Du (249) | 2021 | Vietnam | M | L | NA | NA | NA | NA | 156 | NA | NA | NA | NA | NA | NA | NA | NA | NA | NA | NA | NA | NA | NA | NA | NA | NA | NA | NA | NA | NA | NA | NA | NA | NA | NA | NA | NA | NA | NA | NA | NA | NA | NA | NA | NA | NA |
| V. D. Van Du (249) | 2021 | Vietnam | M | L | NA | NA | NA | NA | NA | NA | NA | NA | NA | NA | NA | NA | NA | NA | NA | NA | NA | NA | NA | NA | NA | NA | NA | NA | NA | NA | NA | NA | NA | NA | NA | NA | NA | 22 | NA | NA | NA | NA | NA | NA | NA | NA |
| V. D. Van Du (249) | 2021 | Vietnam | M | L | NA | NA | NA | NA | NA | NA | NA | NA | NA | NA | NA | NA | NA | NA | NA | NA | NA | NA | NA | NA | 74 | NA | NA | NA | NA | NA | NA | NA | NA | NA | NA | NA | NA | NA | NA | NA | NA | NA | NA | NA | NA | NA |
| H. F. Guo (250) | 2019 | NA | Other | L | 0 | 0 | NA | NA | 27 | 42 | 0 | NA | NA | NA | NA | NA | NA | 58 | NA | NA | 77 | NA | NA | 23.0 | 23 | NA | 0 | NA | 0 | NA | NA | NA | NA | 23 | NA | NA | 0 | NA | NA | NA | NA | NA | NA | NA | NA | NA |
| M. Z. Dashtizade (251) | 2020 | Iran | D | L | NA | 0 | NA | NA | 10 | 14 | 0 | NA | NA | NA | NA | NA | NA | NA | NA | NA | NA | NA | NA | NA | 4 | NA | 0 | NA | NA | NA | NA | NA | 6 | NA | NA | NA | NA | 8 | NA | NA | NA | NA | NA | NA | NA | NA |
| N. K. Dilrukshi (252) | 2023 | Sri Lanka | D | L | 0 | 0 | NA | NA | 10 | 11 | 0 | NA | NA | NA | 0 | NA | NA | NA | NA | NA | NA | NA | NA | NA | NA | NA | NA | NA | NA | NA | NA | NA | NA | NA | NA | NA | NA | NA | NA | NA | NA | NA | NA | NA | NA | NA |
| J. Y. M. Bolukaoto (253) | 2015 | South Africa | D | L | NA | 0 | NA | NA | NA | NA | 0 | NA | NA | NA | NA | NA | NA | NA | NA | NA | 111 | NA | NA | 7.0 | NA | 0 | NA | NA | NA | NA | NA | NA | NA | NA | NA | NA | NA | 21 | NA | NA | NA | NA | NA | NA | NA | NA |
| J. W. Tan (254) | 2022 | NA | D | L | 0 | 0 | NA | NA | 114 | 112 | 0 | NA | NA | NA | 0 | 0 | NA | NA | NA | NA | NA | NA | NA | NA | NA | NA | 0 | NA | NA | NA | NA | NA | NA | NA | NA | NA | NA | NA | NA | NA | NA | NA | NA | NA | NA | NA |
| M. K. Zakerifar (255) | 2023 | NA | M | L | 23 | NA | NA | NA | 56 | 72 | 8 | 56 | NA | NA | NA | NA | NA | 48 | 48 | NA | 100 | NA | NA | NA | 72 | 14 | 0 | NA | NA | NA | NA | NA | NA | NA | NA | NA | 64 | 13 | NA | NA | NA | NA | NA | 83 | NA | NA |
| Z. Q. Cheng (256) | 2020 | NA | D | L | 0 | 0 | NA | NA | 34 | 48 | 0 | NA | NA | NA | NA | NA | NA | NA | NA | NA | NA | NA | NA | NA | NA | NA | 0 | NA | NA | NA | NA | NA | NA | NA | NA | NA | NA | NA | NA | NA | NA | NA | NA | NA | NA | NA |
| R. M. Mudzana (257) | 2021 | Zimbabwe | M | L | 30 | 25 | NA | 31 | 24 | 13 | 13 | 20 | NA | NA | NA | NA | NA | NA | NA | NA | 42 | NA | NA | NA | NA | NA | NA | NA | NA | NA | NA | NA | NA | NA | NA | NA | NA | 15 | NA | NA | NA | NA | NA | NA | NA | NA |
| R. M. Mudzana (257) | 2021 | Zimbabwe | M | L | NA | 0 | NA | NA | NA | NA | NA | NA | NA | NA | NA | NA | NA | NA | NA | NA | NA | NA | NA | NA | NA | NA | NA | NA | NA | NA | NA | NA | NA | NA | NA | NA | NA | 2 | NA | NA | NA | NA | NA | NA | NA | NA |
| Y. Y. H. Yu (258) | 2021 | NA | Other | L | 0 | NA | NA | NA | 3 | NA | 0 | NA | NA | NA | NA | NA | NA | 5 | NA | 6 | 5 | NA | NA | NA | 3 | NA | NA | NA | NA | NA | NA | NA | NA | NA | NA | NA | NA | NA | NA | NA | NA | NA | NA | NA | NA | NA |
| X. T. Zhang (259) | 2023 | NA | Other | L | 0 | 0 | NA | NA | NA | NA | 0 | NA | NA | NA | NA | NA | NA | NA | NA | NA | NA | NA | NA | NA | NA | NA | 0 | NA | 0 | NA | NA | NA | NA | NA | NA | NA | NA | NA | NA | NA | NA | NA | NA | NA | NA | NA |
| Y. H. Fujiya (260) | 2019 | NA | M | L | 0 | 0 | NA | NA | 6 | 10 | 0 | NA | NA | NA | 0 | NA | NA | NA | NA | NA | NA | NA | NA | NA | 12 | NA | NA | NA | NA | NA | NA | NA | NA | NA | NA | NA | NA | NA | NA | NA | NA | NA | NA | NA | NA | NA |
| M. C. B. J. Perim (261) | 2015 | NA | D | L | 6 | NA | NA | NA | NA | 6 | 5 | NA | NA | NA | NA | NA | 0 | 4 | NA | NA | NA | NA | 6 | NA | NA | NA | NA | NA | NA | NA | NA | NA | NA | NA | 6 | NA | NA | NA | NA | NA | 4 | 6 | NA | NA | NA | NA |
| G. F. A.-S. Karim (262) | 2019 | India | D | S | 2 | 0 | 2 | NA | 9 | 4 | NA | 9 | NA | NA | 6 | NA | 9 | NA | NA | NA | NA | NA | NA | NA | NA | NA | NA | NA | NA | NA | NA | NA | NA | NA | NA | NA | NA | NA | NA | NA | NA | NA | NA | NA | NA | NA |
| R. E. F. Mendes (263) | 2015 | NA | M | L | 0 | NA | NA | NA | 82 | 136 | 0 | NA | NA | NA | NA | NA | NA | NA | NA | NA | 206 | NA | 1 | NA | 4 | NA | 0 | 0 | NA | NA | NA | NA | NA | NA | NA | 0 | NA | NA | NA | NA | NA | NA | NA | NA | NA | NA |
| R. E. F. Mendes (263) | 2015 | NA | M | L | 0 | NA | NA | NA | 23 | 39 | 0 | NA | NA | NA | NA | NA | NA | NA | NA | NA | 122 | NA | 0 | NA | 2 | NA | 0 | 0 | NA | NA | NA | NA | NA | NA | NA | 0 | NA | NA | NA | NA | NA | NA | NA | NA | NA | NA |
| M. I. Mukesi (264) | 2019 | NA | Other | L | 0 | 0 | NA | NA | NA | 16 | 0 | 0 | NA | NA | 1 | NA | NA | NA | NA | NA | NA | NA | 17 | NA | 0 | NA | 0 | NA | NA | NA | NA | NA | NA | NA | NA | NA | NA | NA | NA | NA | NA | NA | NA | NA | NA | NA |
| M. A. F. Pfaller (265) | 2016 | NA | M | L | NA | NA | NA | NA | NA | 42 | 0 | NA | NA | NA | NA | NA | NA | NA | NA | NA | NA | NA | 0 | NA | NA | NA | 1 | 0 | 0 | NA | NA | 0 | NA | 0 | NA | 0 | NA | NA | NA | NA | NA | NA | NA | NA | NA | NA |
| M. H. J. C. Ko (266) | 2021 | Taiwan | NA | S | NA | 1 | NA | NA | 9 | 8 | NA | NA | NA | NA | NA | NA | NA | NA | NA | NA | NA | NA | NA | NA | NA | NA | NA | NA | NA | NA | NA | NA | NA | NA | NA | NA | NA | NA | NA | NA | NA | NA | NA | NA | NA | NA |

**Abbreviation:** TMP/SMX, trimethoprim.sulfamethoxazole; SAM, ampicillin.sulbactam; Q/D, quinupristin.dalfopristin; AMC, amoxycillin.clavulanate; S, Some Risk; L, Low Risk; H, High Risk; M, MIC-based Method; D, Disk Diffusion; C, Combined Method.

# Supplementary Table 2: Prevalence of Antibiotic Resistance

| Antibiotic | Category | Subgroup | K (n, N) | Proportion 95%CI(LCI, HCI) | I² | P1 | P2 | P3 |
| --- | --- | --- | --- | --- | --- | --- | --- | --- |
| penicillin | Overall | NA | 108 (979, 68461) | 0.017 (0.013, 0.024) | 0.00% | p<0.001 | p>0.999 | NA |
| ampicillin | Overall | NA | 63 (622, 15558) | 0.031 (0.020, 0.046) | 0.00% | p<0.001 | p=0.993 | NA |
| SAM | Overall | NA | 6 (4, 229) | 0.043 (0.012, 0.140) | 49.25% | p<0.001 | p=0.080 | NA |
| cefazolin | Overall | NA | 11 (39, 1744) | 0.013 (0.002, 0.079) | 0.00% | p<0.001 | p=0.694 | NA |
| clindamycin | Overall | NA | 108 (14263, 51066) | 0.293 (0.269, 0.319) | 83.33% | p<0.001 | p<0.001 | NA |
|  | countries | Australia | 1 (4, 32) | 0.125 (0.048, 0.289) | 0.00% | p<0.001 | p>0.999 | p<0.001 |
|  |  | Nigeria | 2 (200, 231) | 0.762 (0.034, 0.997) | 98.41% | p=0.614 | p<0.001 |  |
|  |  | Iran | 12 (406, 805) | 0.538 (0.346, 0.719) | 93.72% | p=0.708 | p<0.001 |  |
|  |  | Serbia | 4 (360, 1653) | 0.218 (0.199, 0.239) | 0.00% | p<0.001 | p=0.779 |  |
|  |  | Brazil | 6 (110, 1268) | 0.086 (0.048, 0.148) | 86.46% | p<0.001 | p<0.001 |  |
|  |  | Cameroon | 1 (0, 17) | 0.028 (0.002, 0.322) | 0.00% | p=0.013 | p>0.999 |  |
|  |  | Portugal | 3 (55, 990) | 0.081 (0.033, 0.187) | 85.48% | p<0.001 | p=0.001 |  |
|  |  | Argentina | 2 (26, 269) | 0.063 (0.011, 0.293) | 83.91% | p=0.004 | p=0.013 |  |
|  |  | Poland | 1 (8, 42) | 0.190 (0.098, 0.337) | 0.00% | p<0.001 | p>0.999 |  |
|  |  | Malaysia | 3 (32, 238) | 0.135 (0.091, 0.195) | 23.89% | p<0.001 | p=0.269 |  |
|  |  | Taiwan | 15 (1752, 3176) | 0.567 (0.518, 0.614) | 84.24% | p=0.007 | p<0.001 |  |
|  |  | Switzerland | 2 (1257, 3918) | 0.172 (0.032, 0.570) | 98.84% | p=0.096 | p<0.001 |  |
|  |  | Japan | 7 (94, 1017) | 0.108 (0.055, 0.201) | 82.94% | p<0.001 | p<0.001 |  |
|  |  | Indonesia | 1 (11, 53) | 0.208 (0.119, 0.337) | 0.00% | p<0.001 | p>0.999 |  |
|  |  | Kenya | 1 (14, 58) | 0.241 (0.148, 0.367) | 0.00% | p<0.001 | p>0.999 |  |
|  |  | Ethiopia | 7 (73, 479) | 0.136 (0.073, 0.238) | 81.66% | p<0.001 | p<0.001 |  |
|  |  | Palestinian Territories | 1 (6, 24) | 0.250 (0.117, 0.456) | 0.00% | p=0.020 | p>0.999 |  |
|  |  | Namibia | 1 (0, 18) | 0.026 (0.002, 0.310) | 0.00% | p=0.012 | p>0.999 |  |
|  |  | Bosnia & Herzegovina | 1 (6, 17) | 0.353 (0.168, 0.596) | 0.00% | p=0.232 | p>0.999 |  |
|  |  | Canada | 4 (758, 3808) | 0.117 (0.012, 0.591) | 99.40% | p=0.098 | p<0.001 |  |
|  |  | Hong Kong SAR China | 1 (49, 65) | 0.754 (0.635, 0.843) | 0.00% | p<0.001 | p>0.999 |  |
|  |  | Unknown | 1 (9, 18) | 0.500 (0.284, 0.716) | 0.00% | p>0.999 | p>0.999 |  |
|  |  | Germany | 4 (118, 519) | 0.196 (0.103, 0.342) | 85.51% | p<0.001 | p<0.001 |  |
|  |  | Saudi Arabia | 4 (116, 360) | 0.314 (0.142, 0.558) | 92.75% | p=0.132 | p<0.001 |  |
|  |  | Ireland | 2 (68, 342) | 0.199 (0.160, 0.245) | 0.00% | p<0.001 | p=0.710 |  |
|  |  | Kuwait | 1 (6, 9) | 0.667 (0.333, 0.889) | 0.00% | p=0.327 | p>0.999 |  |
|  |  | Qatar | 1 (27, 196) | 0.138 (0.096, 0.193) | 0.00% | p<0.001 | p>0.999 |  |
|  |  | Thailand | 2 (34, 192) | 0.170 (0.092, 0.293) | 68.05% | p<0.001 | p=0.077 |  |
|  |  | Mozambique | 1 (20, 101) | 0.198 (0.131, 0.287) | 0.00% | p<0.001 | p>0.999 |  |
|  |  | Egypt | 3 (78, 221) | 0.361 (0.135, 0.673) | 93.82% | p=0.387 | p<0.001 |  |
|  |  | United States | 4 (1736, 5899) | 0.289 (0.267, 0.312) | 63.23% | p<0.001 | p=0.043 |  |
|  |  | Hungary | 1 (34, 96) | 0.354 (0.265, 0.454) | 0.00% | p=0.005 | p>0.999 |  |
|  |  | India | 3 (29, 68) | 0.576 (0.166, 0.902) | 87.02% | p=0.756 | p<0.001 |  |
|  |  | Sri Lanka | 2 (11, 63) | 0.148 (0.040, 0.423) | 52.46% | p=0.017 | p=0.147 |  |
|  |  | Iceland | 1 (1, 98) | 0.010 (0.001, 0.069) | 0.00% | p<0.001 | p>0.999 |  |
|  |  | Slovenia | 1 (27, 171) | 0.158 (0.111, 0.220) | 0.00% | p<0.001 | p>0.999 |  |
|  |  | Mexico | 1 (0, 17) | 0.028 (0.002, 0.322) | 0.00% | p=0.013 | p>0.999 |  |
|  |  | Colombia | 1 (16, 671) | 0.024 (0.015, 0.039) | 0.00% | p<0.001 | p>0.999 |  |
|  |  | China | 1 (12, 27) | 0.444 (0.272, 0.631) | 0.00% | p=0.565 | p>0.999 |  |
|  |  | France | 2 (2185, 8893) | 0.130 (0.028, 0.433) | 95.20% | p=0.022 | p<0.001 |  |
|  |  | Pakistan | 1 (4, 12) | 0.333 (0.131, 0.624) | 0.00% | p=0.258 | p>0.999 |  |
|  |  | Trinidad & Tobago | 1 (7, 72) | 0.097 (0.047, 0.190) | 0.00% | p<0.001 | p>0.999 |  |
|  |  | United Arab Emirates | 1 (91, 158) | 0.576 (0.498, 0.651) | 0.00% | p=0.057 | p>0.999 |  |
|  |  | Vietnam | 1 (156, 268) | 0.582 (0.522, 0.640) | 0.00% | p=0.007 | p>0.999 |  |
|  |  | Zimbabwe | 1 (24, 43) | 0.558 (0.409, 0.698) | 0.00% | p=0.447 | p>0.999 |  |
|  | continents | Oceania | 1 (4, 32) | 0.125 (0.048, 0.289) | 0.00% | p<0.001 | p>0.999 | p<0.001 |
|  |  | Africa | 18 (415, 1204) | 0.239 (0.144, 0.370) | 92.72% | p<0.001 | p<0.001 |  |
|  |  | Asia | 59 (2844, 6806) | 0.377 (0.323, 0.434) | 93.56% | p<0.001 | p<0.001 |  |
|  |  | NA | 95 (4192, 14180) | 0.341 (0.292, 0.393) | 95.97% | p<0.001 | p<0.001 |  |
|  |  | Europe | 24 (4155, 16840) | 0.194 (0.164, 0.228) | 93.51% | p<0.001 | p<0.001 |  |
|  |  | Americas | 19 (2653, 12004) | 0.115 (0.080, 0.162) | 98.08% | p<0.001 | p<0.001 |  |
|  | AST category | Disk Diffusion | 112 (7857, 28385) | 0.287 (0.253, 0.324) | 96.04% | p<0.001 | p<0.001 | p=0.021 |
|  |  | MIC | 70 (4413, 16350) | 0.272 (0.233, 0.315) | 96.00% | p<0.001 | p<0.001 |  |
|  |  | Other | 12 (628, 1706) | 0.468 (0.337, 0.603) | 94.91% | p=0.645 | p<0.001 |  |
|  |  | Combined method | 2 (33, 102) | 0.324 (0.240, 0.420) | 0.00% | p<0.001 | p=0.777 |  |
|  | Quality group | Low Risk | 185 (13297, 45643) | 0.298 (0.272, 0.324) | 95.97% | p<0.001 | p<0.001 | p=0.003 |
|  |  | Some Risk | 25 (792, 4048) | 0.346 (0.246, 0.461) | 95.70% | p=0.010 | p<0.001 |  |
|  |  | High Risk | 6 (174, 1375) | 0.099 (0.022, 0.352) | 98.02% | p=0.007 | p<0.001 |  |
|  | Year group | 2020_2023 | 132 (8518, 25997) | 0.323 (0.293, 0.354) | 94.67% | p<0.001 | p<0.001 | p=0.007 |
|  |  | 2013_2019 | 84 (5745, 25069) | 0.246 (0.206, 0.291) | 96.99% | p<0.001 | p<0.001 |  |
| erythromycin | Overall | NA | 217 (15548, 47934) | 0.350 (0.324, 0.378) | 95.76% | p<0.001 | p<0.001 | NA |
|  | countries | Australia | 1 (8, 32) | 0.250 (0.130, 0.426) | 0.00% | p=0.007 | p>0.999 | p<0.001 |
|  |  | Nigeria | 2 (87, 231) | 0.377 (0.317, 0.442) | 0.00% | p<0.001 | p=0.348 |  |
|  |  | Iran | 13 (347, 831) | 0.431 (0.314, 0.556) | 88.71% | p=0.277 | p<0.001 |  |
|  |  | Serbia | 4 (433, 1653) | 0.264 (0.229, 0.301) | 43.31% | p<0.001 | p=0.152 |  |
|  |  | Brazil | 6 (141, 1268) | 0.095 (0.037, 0.220) | 95.03% | p<0.001 | p<0.001 |  |
|  |  | Cameroon | 1 (1, 17) | 0.059 (0.008, 0.320) | 0.00% | p=0.007 | p>0.999 |  |
|  |  | Portugal | 3 (78, 1016) | 0.116 (0.052, 0.237) | 86.48% | p<0.001 | p<0.001 |  |
|  |  | Argentina | 2 (44, 269) | 0.085 (0.008, 0.526) | 91.62% | p=0.061 | p<0.001 |  |
|  |  | Poland | 1 (12, 42) | 0.286 (0.170, 0.439) | 0.00% | p=0.007 | p>0.999 |  |
|  |  | Malaysia | 2 (18, 135) | 0.128 (0.038, 0.355) | 82.95% | p=0.004 | p=0.015 |  |
|  |  | Taiwan | 15 (1907, 3176) | 0.608 (0.555, 0.659) | 87.04% | p<0.001 | p<0.001 |  |
|  |  | Switzerland | 2 (1266, 3918) | 0.173 (0.032, 0.574) | 98.86% | p=0.099 | p<0.001 |  |
|  |  | Japan | 7 (190, 1017) | 0.252 (0.132, 0.429) | 91.95% | p=0.008 | p<0.001 |  |
|  |  | Indonesia | 1 (10, 53) | 0.189 (0.105, 0.316) | 0.00% | p<0.001 | p>0.999 |  |
|  |  | Tanzania | 2 (2, 3) | 0.576 (0.087, 0.951) | 30.91% | p=0.822 | p=0.229 |  |
|  |  | Ethiopia | 8 (96, 546) | 0.147 (0.080, 0.255) | 85.75% | p<0.001 | p<0.001 |  |
|  |  | Palestinian Territories | 1 (7, 24) | 0.292 (0.146, 0.498) | 0.00% | p=0.048 | p>0.999 |  |
|  |  | Namibia | 1 (0, 18) | 0.026 (0.002, 0.310) | 0.00% | p=0.012 | p>0.999 |  |
|  |  | Bosnia & Herzegovina | 1 (6, 17) | 0.353 (0.168, 0.596) | 0.00% | p=0.232 | p>0.999 |  |
|  |  | Canada | 3 (825, 3273) | 0.211 (0.015, 0.823) | 99.53% | p=0.365 | p<0.001 |  |
|  |  | Hong Kong SAR China | 2 (73, 111) | 0.644 (0.233, 0.915) | 94.16% | p=0.515 | p<0.001 |  |
|  |  | Unknown | 1 (8, 9) | 0.889 (0.500, 0.985) | 0.00% | p=0.050 | p>0.999 |  |
|  |  | Germany | 4 (137, 521) | 0.233 (0.149, 0.346) | 75.01% | p<0.001 | p=0.007 |  |
|  |  | Saudi Arabia | 4 (147, 360) | 0.444 (0.243, 0.665) | 91.42% | p=0.628 | p<0.001 |  |
|  |  | Ireland | 2 (75, 342) | 0.219 (0.179, 0.266) | 0.00% | p<0.001 | p=0.665 |  |
|  |  | Kuwait | 1 (6, 9) | 0.667 (0.333, 0.889) | 0.00% | p=0.327 | p>0.999 |  |
|  |  | Qatar | 1 (96, 196) | 0.490 (0.420, 0.560) | 0.00% | p=0.775 | p>0.999 |  |
|  |  | Thailand | 3 (35, 292) | 0.101 (0.034, 0.261) | 83.75% | p<0.001 | p=0.002 |  |
|  |  | Mozambique | 2 (40, 136) | 0.261 (0.130, 0.456) | 69.50% | p=0.018 | p=0.070 |  |
|  |  | Egypt | 3 (93, 221) | 0.417 (0.135, 0.766) | 95.65% | p=0.665 | p<0.001 |  |
|  |  | Syria | 1 (7, 10) | 0.700 (0.376, 0.900) | 0.00% | p=0.220 | p>0.999 |  |
|  |  | United States | 2 (416, 881) | 0.472 (0.439, 0.505) | 0.00% | p=0.099 | p=0.502 |  |
|  |  | Hungary | 1 (40, 96) | 0.417 (0.323, 0.517) | 0.00% | p=0.104 | p>0.999 |  |
|  |  | India | 3 (10, 34) | 0.303 (0.169, 0.480) | 0.00% | p=0.030 | p=0.479 |  |
|  |  | Sri Lanka | 2 (12, 63) | 0.154 (0.036, 0.469) | 59.43% | p=0.034 | p=0.116 |  |
|  |  | Iceland | 1 (9, 98) | 0.092 (0.048, 0.167) | 0.00% | p<0.001 | p>0.999 |  |
|  |  | Slovenia | 1 (29, 171) | 0.170 (0.120, 0.233) | 0.00% | p<0.001 | p>0.999 |  |
|  |  | Mexico | 1 (0, 17) | 0.028 (0.002, 0.322) | 0.00% | p=0.013 | p>0.999 |  |
|  |  | Italy | 1 (17, 54) | 0.315 (0.206, 0.449) | 0.00% | p=0.008 | p>0.999 |  |
|  |  | Colombia | 1 (60, 671) | 0.089 (0.070, 0.113) | 0.00% | p<0.001 | p>0.999 |  |
|  |  | China | 1 (17, 27) | 0.630 (0.438, 0.788) | 0.00% | p=0.183 | p>0.999 |  |
|  |  | France | 2 (3184, 8893) | 0.187 (0.036, 0.588) | 97.14% | p=0.114 | p<0.001 |  |
|  |  | Pakistan | 1 (5, 12) | 0.417 (0.185, 0.692) | 0.00% | p=0.566 | p>0.999 |  |
|  |  | Trinidad & Tobago | 1 (22, 72) | 0.306 (0.210, 0.421) | 0.00% | p=0.001 | p>0.999 |  |
|  |  | United Arab Emirates | 1 (90, 158) | 0.570 (0.491, 0.645) | 0.00% | p=0.081 | p>0.999 |  |
|  |  | South Africa | 1 (1, 69) | 0.014 (0.002, 0.096) | 0.00% | p<0.001 | p>0.999 |  |
|  |  | Vietnam | 1 (202, 265) | 0.762 (0.707, 0.810) | 0.00% | p<0.001 | p>0.999 |  |
|  |  | Zimbabwe | 1 (13, 43) | 0.302 (0.184, 0.454) | 0.00% | p=0.012 | p>0.999 |  |
|  | continents | Oceania | 1 (8, 32) | 0.250 (0.130, 0.426) | 0.00% | p=0.007 | p>0.999 | p<0.001 |
|  |  | Africa | 23 (346, 1334) | 0.218 (0.156, 0.296) | 86.29% | p<0.001 | p<0.001 |  |
|  |  | Asia | 61 (3189, 6825) | 0.434 (0.382, 0.487) | 92.45% | p=0.015 | p<0.001 |  |
|  |  | NA | 95 (5184, 16370) | 0.424 (0.372, 0.478) | 96.39% | p=0.006 | p<0.001 |  |
|  |  | Europe | 25 (5313, 16922) | 0.226 (0.195, 0.260) | 93.16% | p<0.001 | p<0.001 |  |
|  |  | Americas | 16 (1508, 6451) | 0.149 (0.085, 0.247) | 98.44% | p<0.001 | p<0.001 |  |
|  | AST category | Disk Diffusion | 114 (9528, 28652) | 0.347 (0.311, 0.385) | 95.98% | p<0.001 | p<0.001 | p=0.024 |
|  |  | MIC | 72 (4256, 13433) | 0.342 (0.293, 0.394) | 96.25% | p<0.001 | p<0.001 |  |
|  |  | Other | 12 (648, 1219) | 0.528 (0.465, 0.591) | 72.04% | p=0.378 | p<0.001 |  |
|  |  | Combined method | 2 (25, 102) | 0.255 (0.164, 0.373) | 20.34% | p<0.001 | p=0.263 |  |
|  | Quality group | Low Risk | 191 (14181, 42765) | 0.359 (0.330, 0.389) | 96.06% | p<0.001 | p<0.001 | p=0.055 |
|  |  | Some Risk | 23 (1087, 4014) | 0.337 (0.253, 0.432) | 94.90% | p=0.001 | p<0.001 |  |
|  |  | High Risk | 7 (280, 1155) | 0.182 (0.076, 0.373) | 96.84% | p=0.003 | p<0.001 |  |
|  | Year group | 2020_2023 | 129 (7612, 20745) | 0.388 (0.352, 0.425) | 94.82% | p<0.001 | p<0.001 | p=0.003 |
|  |  | 2013_2019 | 92 (7936, 27189) | 0.299 (0.259, 0.343) | 96.97% | p<0.001 | p<0.001 |  |
| vancomycin | Overall | NA | 93 (604, 45009) | 0.014 (0.010, 0.020) | 87.35% | p<0.001 | p<0.001 | NA |
|  | countries | Australia | 1 (0, 32) | 0.015 (0.001, 0.201) | 0.00% | p=0.003 | p>0.999 | p<0.001 |
|  |  | Nigeria | 2 (132, 231) | 0.175 (0.002, 0.962) | 91.44% | p=0.525 | p<0.001 |  |
|  |  | Iran | 11 (44, 687) | 0.071 (0.028, 0.166) | 80.51% | p<0.001 | p<0.001 |  |
|  |  | Brazil | 5 (0, 834) | 0.004 (0.001, 0.014) | 0.00% | p<0.001 | p=0.732 |  |
|  |  | Cameroon | 1 (0, 17) | 0.028 (0.002, 0.322) | 0.00% | p=0.013 | p>0.999 |  |
|  |  | Portugal | 2 (0, 773) | 0.001 (0.000, 0.010) | 0.00% | p<0.001 | p=0.641 |  |
|  |  | Japan | 5 (0, 541) | 0.006 (0.002, 0.019) | 0.00% | p<0.001 | p=0.919 |  |
|  |  | Norway | 1 (0, 10) | 0.045 (0.003, 0.448) | 0.00% | p=0.035 | p>0.999 |  |
|  |  | Indonesia | 1 (0, 53) | 0.009 (0.001, 0.131) | 0.00% | p=0.001 | p>0.999 |  |
|  |  | Kenya | 1 (14, 58) | 0.241 (0.148, 0.367) | 0.00% | p<0.001 | p>0.999 |  |
|  |  | Taiwan | 11 (0, 2286) | 0.004 (0.002, 0.009) | 0.00% | p<0.001 | p=0.694 |  |
|  |  | Palestinian Territories | 1 (13, 24) | 0.542 (0.346, 0.725) | 0.00% | p=0.683 | p>0.999 |  |
|  |  | Namibia | 1 (0, 18) | 0.026 (0.002, 0.310) | 0.00% | p=0.012 | p>0.999 |  |
|  |  | Bosnia & Herzegovina | 1 (0, 17) | 0.028 (0.002, 0.322) | 0.00% | p=0.013 | p>0.999 |  |
|  |  | Canada | 3 (23, 3273) | 0.005 (0.001, 0.042) | 71.11% | p<0.001 | p=0.031 |  |
|  |  | Hong Kong SAR China | 1 (0, 65) | 0.008 (0.000, 0.110) | 0.00% | p<0.001 | p>0.999 |  |
|  |  | Germany | 3 (1, 414) | 0.011 (0.001, 0.107) | 62.78% | p<0.001 | p=0.068 |  |
|  |  | Ethiopia | 6 (27, 389) | 0.085 (0.038, 0.181) | 65.94% | p<0.001 | p=0.012 |  |
|  |  | Botswana | 1 (13, 15) | 0.867 (0.595, 0.966) | 0.00% | p=0.014 | p>0.999 |  |
|  |  | Serbia | 1 (0, 432) | 0.001 (0.000, 0.018) | 0.00% | p<0.001 | p>0.999 |  |
|  |  | Kuwait | 1 (0, 9) | 0.050 (0.003, 0.475) | 0.00% | p=0.042 | p>0.999 |  |
|  |  | Qatar | 1 (0, 196) | 0.003 (0.000, 0.039) | 0.00% | p<0.001 | p>0.999 |  |
|  |  | Thailand | 2 (0, 192) | 0.005 (0.001, 0.036) | 0.00% | p<0.001 | p=0.892 |  |
|  |  | Egypt | 3 (14, 221) | 0.034 (0.001, 0.681) | 91.52% | p=0.110 | p<0.001 |  |
|  |  | Syria | 1 (10, 10) | 0.955 (0.552, 0.997) | 0.00% | p=0.035 | p>0.999 |  |
|  |  | Saudi Arabia | 4 (3, 171) | 0.032 (0.004, 0.211) | 69.42% | p=0.001 | p=0.020 |  |
|  |  | India | 2 (1, 59) | 0.032 (0.006, 0.145) | 0.00% | p<0.001 | p=0.391 |  |
|  |  | Sri Lanka | 2 (0, 63) | 0.017 (0.002, 0.110) | 0.00% | p<0.001 | p=0.656 |  |
|  |  | Iceland | 1 (0, 98) | 0.005 (0.000, 0.076) | 0.00% | p<0.001 | p>0.999 |  |
|  |  | Slovenia | 1 (0, 171) | 0.003 (0.000, 0.045) | 0.00% | p<0.001 | p>0.999 |  |
|  |  | Mexico | 2 (0, 591) | 0.005 (0.000, 0.132) | 66.70% | p=0.002 | p=0.083 |  |
|  |  | Switzerland | 1 (0, 3554) | 0.000 (0.000, 0.002) | 0.00% | p<0.001 | p>0.999 |  |
|  |  | Colombia | 1 (4, 671) | 0.006 (0.002, 0.016) | 0.00% | p<0.001 | p>0.999 |  |
|  |  | China | 1 (0, 27) | 0.018 (0.001, 0.230) | 0.00% | p=0.005 | p>0.999 |  |
|  |  | United States | 2 (29, 279) | 0.098 (0.007, 0.642) | 96.75% | p=0.121 | p<0.001 |  |
|  |  | France | 2 (0, 8893) | 0.000 (0.000, 0.026) | 76.86% | p<0.001 | p=0.038 |  |
|  |  | Pakistan | 1 (1, 12) | 0.083 (0.012, 0.413) | 0.00% | p=0.022 | p>0.999 |  |
|  |  | Trinidad & Tobago | 1 (0, 72) | 0.007 (0.000, 0.100) | 0.00% | p<0.001 | p>0.999 |  |
|  |  | United Arab Emirates | 1 (4, 158) | 0.025 (0.010, 0.065) | 0.00% | p<0.001 | p>0.999 |  |
|  |  | Malaysia | 1 (0, 103) | 0.005 (0.000, 0.072) | 0.00% | p<0.001 | p>0.999 |  |
|  |  | South Africa | 2 (0, 197) | 0.005 (0.001, 0.036) | 0.00% | p<0.001 | p=0.759 |  |
|  |  | Iraq | 1 (0, 16) | 0.029 (0.002, 0.336) | 0.00% | p=0.015 | p>0.999 |  |
|  |  | Vietnam | 1 (0, 244) | 0.002 (0.000, 0.032) | 0.00% | p<0.001 | p>0.999 |  |
|  |  | Zimbabwe | 1 (13, 43) | 0.302 (0.184, 0.454) | 0.00% | p=0.012 | p>0.999 |  |
|  | continents | Oceania | 1 (0, 32) | 0.015 (0.001, 0.201) | 0.00% | p=0.003 | p>0.999 | p=0.001 |
|  |  | Africa | 19 (214, 1225) | 0.085 (0.039, 0.176) | 91.41% | p<0.001 | p<0.001 |  |
|  |  | Asia | 51 (76, 4991) | 0.018 (0.010, 0.034) | 82.64% | p<0.001 | p<0.001 |  |
|  |  | NA | 87 (252, 18614) | 0.011 (0.007, 0.018) | 74.73% | p<0.001 | p<0.001 |  |
|  |  | Europe | 14 (6, 14427) | 0.004 (0.001, 0.016) | 77.49% | p<0.001 | p<0.001 |  |
|  |  | Americas | 14 (56, 5720) | 0.008 (0.002, 0.028) | 92.46% | p<0.001 | p<0.001 |  |
|  | AST category | Disk Diffusion | 91 (536, 24047) | 0.026 (0.016, 0.043) | 91.04% | p<0.001 | p<0.001 | p=0.002 |
|  |  | MIC | 62 (38, 11268) | 0.007 (0.004, 0.011) | 67.90% | p<0.001 | p<0.001 |  |
|  |  | Other | 14 (12, 6139) | 0.009 (0.004, 0.020) | 56.24% | p<0.001 | p=0.005 |  |
|  |  | Combined method | 2 (0, 102) | 0.013 (0.002, 0.085) | 0.00% | p<0.001 | p=0.432 |  |
|  | Quality group | Low Risk | 164 (583, 40610) | 0.014 (0.009, 0.020) | 89.37% | p<0.001 | p<0.001 | p=0.914 |
|  |  | Some Risk | 17 (15, 2925) | 0.018 (0.005, 0.058) | 80.52% | p<0.001 | p<0.001 |  |
|  |  | High Risk | 5 (6, 1474) | 0.015 (0.002, 0.103) | 78.19% | p<0.001 | p=0.001 |  |
|  | Year group | 2020_2023 | 117 (199, 21339) | 0.017 (0.012, 0.026) | 83.40% | p<0.001 | p<0.001 | p=0.302 |
|  |  | 2013_2019 | 69 (405, 23670) | 0.010 (0.005, 0.022) | 92.82% | p<0.001 | p<0.001 |  |
| ceftriaxone | Overall | NA | 77 (724, 30196) | 0.062 (0.039, 0.097) | 91.54% | p<0.001 | p<0.001 | NA |
|  | countries | Nigeria | 1 (0, 33) | 0.015 (0.001, 0.196) | 0.00% | p=0.003 | p>0.999 | p=0.069 |
|  |  | Iran | 6 (61, 412) | 0.117 (0.020, 0.458) | 94.33% | p=0.032 | p<0.001 |  |
|  |  | Japan | 4 (32, 20096) | 0.007 (0.000, 0.455) | 95.55% | p=0.042 | p<0.001 |  |
|  |  | Taiwan | 5 (0, 1278) | 0.004 (0.001, 0.013) | 8.54% | p<0.001 | p=0.358 |  |
|  |  | Tanzania | 1 (0, 1) | 0.250 (0.013, 0.891) | 0.00% | p=0.501 | p>0.999 |  |
|  |  | Namibia | 1 (0, 18) | 0.026 (0.002, 0.310) | 0.00% | p=0.012 | p>0.999 |  |
|  |  | Ethiopia | 5 (37, 276) | 0.156 (0.080, 0.283) | 68.39% | p<0.001 | p=0.013 |  |
|  |  | Qatar | 1 (0, 196) | 0.003 (0.000, 0.039) | 0.00% | p<0.001 | p>0.999 |  |
|  |  | Thailand | 2 (0, 192) | 0.005 (0.001, 0.036) | 0.00% | p<0.001 | p=0.892 |  |
|  |  | Mozambique | 1 (0, 101) | 0.005 (0.000, 0.073) | 0.00% | p<0.001 | p>0.999 |  |
|  |  | Syria | 1 (7, 10) | 0.700 (0.376, 0.900) | 0.00% | p=0.220 | p>0.999 |  |
|  |  | United States | 1 (0, 803) | 0.001 (0.000, 0.010) | 0.00% | p<0.001 | p>0.999 |  |
|  |  | China | 1 (0, 27) | 0.018 (0.001, 0.230) | 0.00% | p=0.005 | p>0.999 |  |
|  |  | Trinidad & Tobago | 1 (4, 72) | 0.056 (0.021, 0.139) | 0.00% | p<0.001 | p>0.999 |  |
|  |  | United Arab Emirates | 1 (6, 158) | 0.038 (0.017, 0.082) | 0.00% | p<0.001 | p>0.999 |  |
|  |  | Italy | 1 (0, 87) | 0.006 (0.000, 0.084) | 0.00% | p<0.001 | p>0.999 |  |
|  |  | Malawi | 1 (0, 55) | 0.009 (0.001, 0.127) | 0.00% | p<0.001 | p>0.999 |  |
|  |  | Mexico | 2 (7, 592) | 0.026 (0.000, 0.945) | 94.86% | p=0.272 | p<0.001 |  |
|  |  | Malaysia | 1 (0, 103) | 0.005 (0.000, 0.072) | 0.00% | p<0.001 | p>0.999 |  |
|  |  | Vietnam | 1 (0, 33) | 0.015 (0.001, 0.196) | 0.00% | p=0.003 | p>0.999 |  |
|  |  | Zimbabwe | 1 (20, 43) | 0.465 (0.323, 0.613) | 0.00% | p=0.648 | p>0.999 |  |
|  |  | India | 1 (9, 9) | 0.950 (0.525, 0.997) | 0.00% | p=0.042 | p>0.999 |  |
|  | continents | Africa | 11 (57, 527) | 0.105 (0.049, 0.212) | 79.15% | p<0.001 | p<0.001 | p=0.102 |
|  |  | Asia | 24 (115, 22514) | 0.027 (0.010, 0.071) | 92.08% | p<0.001 | p<0.001 |  |
|  |  | NA | 38 (541, 5601) | 0.104 (0.053, 0.192) | 92.32% | p<0.001 | p<0.001 |  |
|  |  | Americas | 4 (11, 1467) | 0.016 (0.001, 0.201) | 92.45% | p=0.003 | p<0.001 |  |
|  |  | Europe | 1 (0, 87) | 0.006 (0.000, 0.084) | 0.00% | p<0.001 | p>0.999 |  |
|  | AST category | MIC | 26 (129, 22943) | 0.015 (0.006, 0.037) | 91.39% | p<0.001 | p<0.001 | p<0.001 |
|  |  | Disk Diffusion | 38 (530, 2824) | 0.141 (0.077, 0.244) | 91.92% | p<0.001 | p<0.001 |  |
|  |  | Other | 7 (37, 4063) | 0.038 (0.007, 0.176) | 87.43% | p<0.001 | p<0.001 |  |
|  |  | Combined method | 2 (0, 172) | 0.006 (0.001, 0.040) | 0.00% | p<0.001 | p=0.991 |  |
|  | Quality group | Low Risk | 68 (665, 10017) | 0.058 (0.035, 0.094) | 91.92% | p<0.001 | p<0.001 | p=0.709 |
|  |  | Some Risk | 9 (59, 20165) | 0.098 (0.016, 0.417) | 91.18% | p=0.021 | p<0.001 |  |
|  |  | High Risk | 1 (0, 14) | 0.033 (0.002, 0.366) | 0.00% | p=0.019 | p>0.999 |  |
|  | Year group | 2020_2023 | 46 (446, 26087) | 0.097 (0.055, 0.165) | 92.62% | p<0.001 | p<0.001 | p=0.013 |
|  |  | 2013_2019 | 32 (278, 4109) | 0.031 (0.015, 0.062) | 85.31% | p<0.001 | p<0.001 |  |
| amoxicillin | Overall | NA | 10 (43, 9837) | 0.035 (0.006, 0.178) | 91.79% | p<0.001 | p<0.001 | NA |
|  | continents | NA | 6 (14, 851) | 0.050 (0.004, 0.435) | 90.68% | p=0.031 | p<0.001 | p=0.276 |
|  |  | Americas | 2 (27, 220) | 0.051 (0.000, 0.896) | 92.31% | p=0.259 | p<0.001 |  |
|  |  | Europe | 1 (0, 8757) | 0.000 (0.000, 0.001) | 0.00% | p<0.001 | p>0.999 |  |
|  |  | Africa | 1 (2, 9) | 0.222 (0.056, 0.579) | 0.00% | p=0.118 | p>0.999 |  |
|  | AST category | Disk Diffusion | 7 (43, 9367) | 0.087 (0.014, 0.392) | 92.05% | p=0.016 | p<0.001 | p=0.316 |
|  |  | MIC | 1 (0, 150) | 0.003 (0.000, 0.051) | 0.00% | p<0.001 | p>0.999 |  |
|  |  | Other | 1 (0, 148) | 0.003 (0.000, 0.051) | 0.00% | p<0.001 | p>0.999 |  |
|  | Year group | 2020_2023 | 6 (33, 911) | 0.042 (0.005, 0.262) | 90.23% | p=0.003 | p<0.001 | p=0.867 |
|  |  | 2013_2019 | 4 (10, 8926) | 0.024 (0.000, 0.664) | 94.65% | p=0.098 | p<0.001 |  |
| cefuroxime | Overall | NA | 17 (53.3, 4806) | 0.030 (0.012, 0.070) | 82.72% | p<0.001 | p<0.001 | NA |
|  | continents | NA | 6 (18, 214) | 0.092 (0.021, 0.323) | 77.24% | p=0.004 | p<0.001 | p<0.001 |
|  |  | Europe | 3 (0, 3707) | 0.002 (0.000, 0.022) | 59.40% | p<0.001 | p=0.085 |  |
|  |  | Asia | 5 (0, 574) | 0.006 (0.002, 0.021) | 0.00% | p<0.001 | p=0.657 |  |
|  |  | Americas | 3 (35.3, 311) | 0.117 (0.062, 0.209) | 54.53% | p<0.001 | p=0.111 |  |
|  | AST category | Disk Diffusion | 12 (26, 4091) | 0.037 (0.011, 0.115) | 84.15% | p<0.001 | p<0.001 | p=0.674 |
|  |  | MIC | 2 (0, 169) | 0.006 (0.001, 0.041) | 0.00% | p<0.001 | p=0.825 |  |
|  |  | Other | 2 (27.3, 459) | 0.021 (0.000, 0.567) | 88.45% | p=0.067 | p=0.003 |  |
|  |  | Combined method | 1 (0, 87) | 0.006 (0.000, 0.084) | 0.00% | p<0.001 | p>0.999 |  |
|  | Year group | 2013_2019 | 7 (4, 604) | 0.011 (0.002, 0.063) | 75.55% | p<0.001 | p<0.001 | p=0.125 |
|  |  | 2020_2023 | 10 (49.3, 4202) | 0.053 (0.019, 0.139) | 84.79% | p<0.001 | p<0.001 |  |
| cefotaxime | Overall | NA | 33 (367, 6595) | 0.032 (0.017, 0.060) | 0.00% | p<0.001 | p>0.999 | NA |
| meropenem | Overall | NA | 13 (24, 26329) | 0.007 (0.003, 0.017) | 0.00% | p<0.001 | p>0.999 | NA |
| imipenem | Overall | NA | 11 (26, 384) | 0.065 (0.023, 0.166) | 58.66% | p<0.001 | p=0.007 | NA |
|  | countries | Japan | 3 (0, 155) | 0.011 (0.002, 0.053) | 0.00% | p<0.001 | p=0.769 | p<0.001 |
|  |  | Syria | 1 (0, 10) | 0.045 (0.003, 0.448) | 0.00% | p=0.035 | p>0.999 |  |
|  |  | Trinidad & Tobago | 1 (5, 72) | 0.069 (0.029, 0.156) | 0.00% | p<0.001 | p>0.999 |  |
|  |  | India | 1 (9, 9) | 0.950 (0.525, 0.997) | 0.00% | p=0.042 | p>0.999 |  |
|  | continents | NA | 6 (12, 138) | 0.073 (0.020, 0.227) | 51.50% | p<0.001 | p=0.067 | p=0.998 |
|  |  | Asia | 5 (9, 174) | 0.061 (0.004, 0.530) | 80.50% | p=0.060 | p<0.001 |  |
|  |  | Americas | 1 (5, 72) | 0.069 (0.029, 0.156) | 0.00% | p<0.001 | p>0.999 |  |
|  | AST category | Disk Diffusion | 9 (26, 229) | 0.105 (0.037, 0.266) | 68.36% | p<0.001 | p=0.001 | p=0.047 |
|  |  | MIC | 3 (0, 155) | 0.011 (0.002, 0.053) | 0.00% | p<0.001 | p=0.769 |  |
|  | Quality group | Low Risk | 10 (17, 299) | 0.057 (0.024, 0.133) | 54.63% | p<0.001 | p=0.019 | p=0.233 |
|  |  | Some Risk | 2 (9, 85) | 0.259 (0.000, 0.999) | 93.52% | p=0.793 | p<0.001 |  |
|  | Year group | 2013_2019 | 4 (0, 124) | 0.020 (0.005, 0.078) | 0.00% | p<0.001 | p=0.715 | p=0.132 |
|  |  | 2020_2023 | 8 (26, 260) | 0.101 (0.031, 0.284) | 74.01% | p<0.001 | p<0.001 |  |
| azithromycin | Overall | NA | 21 (3580, 21334) | 0.410 (0.280, 0.554) | 97.29% | p=0.218 | p<0.001 | NA |
|  | countries | Japan | 3 (3029, 20015) | 0.242 (0.084, 0.527) | 96.48% | p=0.073 | p<0.001 | p=0.241 |
|  |  | Brazil | 1 (5, 55) | 0.091 (0.038, 0.200) | 0.00% | p<0.001 | p>0.999 |  |
|  |  | Taiwan | 2 (182, 415) | 0.116 (0.001, 0.933) | 95.50% | p=0.394 | p<0.001 |  |
|  |  | Iran | 1 (37, 43) | 0.860 (0.722, 0.936) | 0.00% | p<0.001 | p>0.999 |  |
|  |  | Ethiopia | 2 (10, 110) | 0.091 (0.050, 0.161) | 0.00% | p<0.001 | p=0.785 |  |
|  |  | Egypt | 1 (16, 100) | 0.160 (0.100, 0.245) | 0.00% | p<0.001 | p>0.999 |  |
|  |  | Syria | 1 (7, 10) | 0.700 (0.376, 0.900) | 0.00% | p=0.220 | p>0.999 |  |
|  |  | Thailand | 1 (24, 109) | 0.220 (0.152, 0.308) | 0.00% | p<0.001 | p>0.999 |  |
|  |  | Italy | 1 (17, 87) | 0.195 (0.125, 0.292) | 0.00% | p<0.001 | p>0.999 |  |
|  | continents | Asia | 8 (3279, 20592) | 0.337 (0.163, 0.572) | 98.42% | p=0.170 | p<0.001 | p=0.002 |
|  |  | NA | 8 (253, 390) | 0.722 (0.558, 0.842) | 83.87% | p=0.009 | p<0.001 |  |
|  |  | Americas | 1 (5, 55) | 0.091 (0.038, 0.200) | 0.00% | p<0.001 | p>0.999 |  |
|  |  | Africa | 3 (26, 210) | 0.126 (0.083, 0.185) | 13.15% | p<0.001 | p=0.316 |  |
|  |  | Europe | 1 (17, 87) | 0.195 (0.125, 0.292) | 0.00% | p<0.001 | p>0.999 |  |
|  | AST category | MIC | 8 (3200, 20381) | 0.410 (0.233, 0.613) | 96.18% | p=0.386 | p<0.001 | p=0.688 |
|  |  | Disk Diffusion | 7 (100, 331) | 0.378 (0.165, 0.651) | 92.29% | p=0.383 | p<0.001 |  |
|  |  | Other | 5 (263, 535) | 0.536 (0.335, 0.726) | 86.63% | p=0.733 | p<0.001 |  |
|  |  | Combined method | 1 (17, 87) | 0.195 (0.125, 0.292) | 0.00% | p<0.001 | p>0.999 |  |
|  | Quality group | Some Risk | 3 (3041, 19997) | 0.462 (0.142, 0.817) | 97.80% | p=0.858 | p<0.001 | p=0.767 |
|  |  | Low Risk | 18 (539, 1337) | 0.397 (0.270, 0.540) | 93.06% | p=0.156 | p<0.001 |  |
|  | Year group | 2020_2023 | 13 (3235, 20580) | 0.359 (0.211, 0.541) | 96.65% | p=0.126 | p<0.001 | p=0.350 |
|  |  | 2013_2019 | 8 (345, 754) | 0.498 (0.278, 0.718) | 94.51% | p=0.984 | p<0.001 |  |
| clarithromycin | Overall | NA | 11 (480, 1468) | 0.434 (0.303, 0.575) | 93.05% | p=0.062 | p<0.001 | NA |
|  | countries | Japan | 3 (93, 193) | 0.396 (0.131, 0.741) | 94.35% | p=0.575 | p<0.001 | p=0.627 |
|  |  | Canada | 1 (111, 535) | 0.207 (0.175, 0.244) | 0.00% | p<0.001 | p>0.999 |  |
|  |  | Ethiopia | 1 (2, 16) | 0.125 (0.031, 0.386) | 0.00% | p=0.010 | p>0.999 |  |
|  |  | Syria | 1 (7, 10) | 0.700 (0.376, 0.900) | 0.00% | p=0.220 | p>0.999 |  |
|  |  | Italy | 1 (17, 87) | 0.195 (0.125, 0.292) | 0.00% | p<0.001 | p>0.999 |  |
|  | continents | NA | 5 (250, 627) | 0.595 (0.365, 0.789) | 95.17% | p=0.424 | p<0.001 | p=0.227 |
|  |  | Asia | 4 (100, 203) | 0.464 (0.201, 0.749) | 91.82% | p=0.818 | p<0.001 |  |
|  |  | Americas | 1 (111, 535) | 0.207 (0.175, 0.244) | 0.00% | p<0.001 | p>0.999 |  |
|  |  | Africa | 1 (2, 16) | 0.125 (0.031, 0.386) | 0.00% | p=0.010 | p>0.999 |  |
|  |  | Europe | 1 (17, 87) | 0.195 (0.125, 0.292) | 0.00% | p<0.001 | p>0.999 |  |
|  | AST category | MIC | 8 (343, 820) | 0.523 (0.342, 0.698) | 94.66% | p=0.807 | p<0.001 | p=0.367 |
|  |  | Other | 1 (111, 535) | 0.207 (0.175, 0.244) | 0.00% | p<0.001 | p>0.999 |  |
|  |  | Disk Diffusion | 2 (9, 26) | 0.370 (0.037, 0.901) | 86.57% | p=0.703 | p=0.006 |  |
|  |  | Combined method | 1 (17, 87) | 0.195 (0.125, 0.292) | 0.00% | p<0.001 | p>0.999 |  |
|  | Quality group | Low Risk | 10 (337, 857) | 0.471 (0.301, 0.647) | 93.95% | p=0.750 | p<0.001 | p=0.566 |
|  |  | High Risk | 1 (111, 535) | 0.207 (0.175, 0.244) | 0.00% | p<0.001 | p>0.999 |  |
|  |  | Some Risk | 1 (32, 76) | 0.421 (0.316, 0.534) | 0.00% | p=0.170 | p>0.999 |  |
|  | Year group | 2013_2019 | 5 (219, 608) | 0.538 (0.298, 0.762) | 95.09% | p=0.769 | p<0.001 | p=0.296 |
|  |  | 2020_2023 | 7 (261, 860) | 0.368 (0.205, 0.567) | 94.30% | p=0.191 | p<0.001 |  |
| erythrocin | Overall | NA | 7 (300, 554) | 0.597 (0.310, 0.829) | 96.27% | p=0.519 | p<0.001 | NA |
|  | continents | NA | 4 (151, 250) | 0.749 (0.368, 0.938) | 94.18% | p=0.191 | p<0.001 | p=0.189 |
|  |  | Africa | 1 (8, 65) | 0.123 (0.063, 0.227) | 0.00% | p<0.001 | p>0.999 |  |
|  |  | Asia | 1 (24, 103) | 0.233 (0.161, 0.324) | 0.00% | p<0.001 | p>0.999 |  |
|  |  | Americas | 1 (117, 136) | 0.860 (0.791, 0.909) | 0.00% | p<0.001 | p>0.999 |  |
|  | AST category | MIC | 2 (33, 114) | 0.513 (0.070, 0.936) | 90.84% | p=0.969 | p<0.001 | p=0.741 |
|  |  | Disk Diffusion | 4 (261, 433) | 0.580 (0.214, 0.875) | 97.52% | p=0.697 | p<0.001 |  |
|  |  | Other | 1 (6, 7) | 0.857 (0.419, 0.980) | 0.00% | p=0.097 | p>0.999 |  |
|  | Year group | 2020_2023 | 4 (151, 250) | 0.749 (0.368, 0.938) | 94.18% | p=0.191 | p<0.001 | p=0.281 |
|  |  | 2013_2019 | 3 (149, 304) | 0.393 (0.062, 0.864) | 98.12% | p=0.709 | p<0.001 |  |
| tetracycline | Overall | NA | 62 (21931, 28322) | 0.801 (0.771, 0.828) | 80.79% | p<0.001 | p<0.001 | NA |
|  | countries | Australia | 2 (45, 64) | 0.703 (0.580, 0.802) | 0.00% | p=0.002 | p=0.784 | p=0.002 |
|  |  | Nigeria | 1 (33, 33) | 0.985 (0.804, 0.999) | 0.00% | p=0.003 | p>0.999 |  |
|  |  | Iran | 11 (697, 769) | 0.913 (0.840, 0.954) | 80.14% | p<0.001 | p<0.001 |  |
|  |  | Serbia | 2 (1286, 1503) | 0.856 (0.837, 0.872) | 0.00% | p<0.001 | p=0.479 |  |
|  |  | Brazil | 3 (554, 666) | 0.871 (0.654, 0.960) | 94.95% | p=0.003 | p<0.001 |  |
|  |  | Malaysia | 3 (182, 238) | 0.779 (0.653, 0.869) | 73.05% | p<0.001 | p=0.024 |  |
|  |  | Indonesia | 1 (47, 53) | 0.887 (0.770, 0.948) | 0.00% | p<0.001 | p>0.999 |  |
|  |  | Palestinian Territories | 1 (18, 24) | 0.750 (0.544, 0.883) | 0.00% | p=0.020 | p>0.999 |  |
|  |  | Taiwan | 6 (610, 692) | 0.903 (0.779, 0.961) | 91.88% | p<0.001 | p<0.001 |  |
|  |  | Canada | 1 (23, 37) | 0.622 (0.458, 0.761) | 0.00% | p=0.143 | p>0.999 |  |
|  |  | Ethiopia | 5 (204, 276) | 0.748 (0.512, 0.893) | 90.42% | p=0.040 | p<0.001 |  |
|  |  | Saudi Arabia | 3 (230, 300) | 0.766 (0.714, 0.810) | 0.00% | p<0.001 | p=0.713 |  |
|  |  | Thailand | 3 (153, 292) | 0.587 (0.264, 0.849) | 95.93% | p=0.615 | p<0.001 |  |
|  |  | Egypt | 2 (175, 192) | 0.935 (0.614, 0.992) | 88.41% | p=0.018 | p=0.003 |  |
|  |  | Portugal | 1 (478, 555) | 0.861 (0.830, 0.888) | 0.00% | p<0.001 | p>0.999 |  |
|  |  | United States | 2 (765, 881) | 0.905 (0.769, 0.965) | 77.29% | p<0.001 | p=0.036 |  |
|  |  | Hungary | 1 (79, 96) | 0.823 (0.733, 0.887) | 0.00% | p<0.001 | p>0.999 |  |
|  |  | Slovenia | 1 (148, 171) | 0.865 (0.806, 0.909) | 0.00% | p<0.001 | p>0.999 |  |
|  |  | Mexico | 2 (11, 591) | 0.043 (0.000, 0.988) | 96.14% | p=0.417 | p<0.001 |  |
|  |  | Colombia | 1 (593, 671) | 0.884 (0.857, 0.906) | 0.00% | p<0.001 | p>0.999 |  |
|  |  | Ireland | 1 (205, 235) | 0.872 (0.823, 0.909) | 0.00% | p<0.001 | p>0.999 |  |
|  |  | China | 1 (20, 27) | 0.741 (0.547, 0.871) | 0.00% | p=0.017 | p>0.999 |  |
|  |  | France | 2 (7691, 8893) | 0.855 (0.818, 0.886) | 49.98% | p<0.001 | p=0.157 |  |
|  |  | Pakistan | 1 (1, 12) | 0.083 (0.012, 0.413) | 0.00% | p=0.022 | p>0.999 |  |
|  |  | India | 1 (3, 6) | 0.500 (0.168, 0.832) | 0.00% | p>0.999 | p>0.999 |  |
|  |  | Italy | 1 (70, 87) | 0.805 (0.708, 0.875) | 0.00% | p<0.001 | p>0.999 |  |
|  |  | Gambia | 1 (119, 133) | 0.895 (0.830, 0.937) | 0.00% | p<0.001 | p>0.999 |  |
|  |  | Iraq | 1 (9, 16) | 0.562 (0.324, 0.775) | 0.00% | p=0.618 | p>0.999 |  |
|  |  | Vietnam | 1 (234, 244) | 0.959 (0.926, 0.978) | 0.00% | p<0.001 | p>0.999 |  |
|  |  | South Africa | 1 (111, 128) | 0.867 (0.797, 0.916) | 0.00% | p<0.001 | p>0.999 |  |
|  |  | Zimbabwe | 1 (42, 43) | 0.977 (0.853, 0.997) | 0.00% | p<0.001 | p>0.999 |  |
|  | continents | Oceania | 2 (45, 64) | 0.703 (0.580, 0.802) | 0.00% | p=0.002 | p=0.784 | p=0.424 |
|  |  | Africa | 12 (696, 819) | 0.862 (0.767, 0.923) | 87.24% | p<0.001 | p<0.001 |  |
|  |  | Asia | 34 (2215, 2696) | 0.829 (0.762, 0.880) | 91.46% | p<0.001 | p<0.001 |  |
|  |  | NA | 57 (7062, 10292) | 0.776 (0.729, 0.817) | 95.55% | p<0.001 | p<0.001 |  |
|  |  | Europe | 10 (9967, 11605) | 0.816 (0.770, 0.856) | 92.03% | p<0.001 | p<0.001 |  |
|  |  | Americas | 9 (1946, 2846) | 0.807 (0.707, 0.879) | 93.26% | p<0.001 | p<0.001 |  |
|  | AST category | Disk Diffusion | 65 (13710, 16108) | 0.816 (0.788, 0.841) | 87.71% | p<0.001 | p<0.001 | p=0.733 |
|  |  | Other | 9 (551, 626) | 0.852 (0.747, 0.918) | 82.83% | p<0.001 | p<0.001 |  |
|  |  | MIC | 38 (5453, 8210) | 0.802 (0.743, 0.849) | 96.67% | p<0.001 | p<0.001 |  |
|  |  | Combined method | 1 (70, 87) | 0.805 (0.708, 0.875) | 0.00% | p<0.001 | p>0.999 |  |
|  | Quality group | Low Risk | 109 (20449, 25127) | 0.818 (0.795, 0.839) | 92.02% | p<0.001 | p<0.001 | p<0.001 |
|  |  | Some Risk | 13 (1455, 2695) | 0.705 (0.617, 0.780) | 91.08% | p<0.001 | p<0.001 |  |
|  |  | High Risk | 2 (27, 500) | 0.078 (0.019, 0.268) | 92.00% | p<0.001 | p<0.001 |  |
|  | Year group | 2020_2023 | 69 (7176, 8901) | 0.806 (0.768, 0.839) | 91.41% | p<0.001 | p<0.001 | p=0.686 |
|  |  | 2013_2019 | 55 (14755, 19421) | 0.795 (0.745, 0.838) | 97.49% | p<0.001 | p<0.001 |  |
| doxycycline | Overall | NA | 5 (188, 372) | 0.649 (0.371, 0.853) | 93.66% | p=0.293 | p<0.001 | NA |
|  | continents | NA | 4 (165, 344) | 0.583 (0.295, 0.824) | 93.79% | p=0.586 | p<0.001 | p=0.383 |
|  |  | Asia | 1 (23, 28) | 0.821 (0.636, 0.924) | 0.00% | p=0.002 | p>0.999 |  |
|  | AST category | Disk Diffusion | 1 (62, 62) | 0.992 (0.885, 1.000) | 0.00% | p<0.001 | p>0.999 | p=0.022 |
|  |  | MIC | 3 (92, 250) | 0.529 (0.202, 0.833) | 95.30% | p=0.879 | p<0.001 |  |
|  | Year group | 2013_2019 | 3 (119, 150) | 0.839 (0.495, 0.965) | 85.67% | p=0.053 | p<0.001 | p=0.063 |
|  |  | 2020_2023 | 2 (69, 222) | 0.373 (0.114, 0.732) | 95.70% | p=0.504 | p<0.001 |  |
| TMP/SMX | Overall | NA | 29 (371, 5705) | 0.213 (0.107, 0.378) | 93.33% | p=0.007 | p<0.001 | NA |
|  | countries | Australia | 1 (0, 32) | 0.015 (0.001, 0.201) | 0.00% | p=0.003 | p>0.999 | p=0.123 |
|  |  | Tanzania | 1 (0, 1) | 0.250 (0.013, 0.891) | 0.00% | p=0.501 | p>0.999 |  |
|  |  | Bahrain | 1 (0, 123) | 0.004 (0.000, 0.061) | 0.00% | p<0.001 | p>0.999 |  |
|  |  | Germany | 1 (3, 66) | 0.045 (0.015, 0.132) | 0.00% | p<0.001 | p>0.999 |  |
|  |  | Syria | 1 (7, 10) | 0.700 (0.376, 0.900) | 0.00% | p=0.220 | p>0.999 |  |
|  |  | Slovenia | 1 (0, 171) | 0.003 (0.000, 0.045) | 0.00% | p<0.001 | p>0.999 |  |
|  |  | Iran | 1 (7, 7) | 0.938 (0.461, 0.996) | 0.00% | p=0.064 | p>0.999 |  |
|  |  | Switzerland | 1 (0, 3554) | 0.000 (0.000, 0.002) | 0.00% | p<0.001 | p>0.999 |  |
|  |  | Trinidad & Tobago | 1 (42, 72) | 0.583 (0.467, 0.691) | 0.00% | p=0.159 | p>0.999 |  |
|  |  | United Arab Emirates | 1 (122, 158) | 0.772 (0.700, 0.831) | 0.00% | p<0.001 | p>0.999 |  |
|  |  | Saudi Arabia | 2 (88, 220) | 0.478 (0.029, 0.966) | 98.76% | p=0.960 | p<0.001 |  |
|  |  | Mexico | 1 (13, 18) | 0.722 (0.481, 0.879) | 0.00% | p=0.069 | p>0.999 |  |
|  |  | Lesotho | 1 (3, 9) | 0.333 (0.111, 0.667) | 0.00% | p=0.327 | p>0.999 |  |
|  |  | Iraq | 1 (7, 16) | 0.438 (0.225, 0.676) | 0.00% | p=0.618 | p>0.999 |  |
|  | continents | Oceania | 1 (0, 32) | 0.015 (0.001, 0.201) | 0.00% | p=0.003 | p>0.999 | p=0.005 |
|  |  | Europe | 4 (6, 3856) | 0.008 (0.001, 0.062) | 83.47% | p<0.001 | p<0.001 |  |
|  |  | Africa | 3 (16, 24) | 0.583 (0.119, 0.935) | 73.04% | p=0.779 | p=0.024 |  |
|  |  | NA | 13 (63, 1169) | 0.176 (0.040, 0.521) | 92.52% | p=0.063 | p<0.001 |  |
|  |  | Asia | 7 (231, 534) | 0.491 (0.189, 0.799) | 95.71% | p=0.961 | p<0.001 |  |
|  |  | Americas | 2 (55, 90) | 0.615 (0.493, 0.724) | 12.82% | p=0.065 | p=0.284 |  |
|  | AST category | Disk Diffusion | 19 (140, 4749) | 0.226 (0.094, 0.452) | 92.08% | p=0.020 | p<0.001 | p<0.001 |
|  |  | Other | 5 (224, 289) | 0.748 (0.622, 0.843) | 66.27% | p<0.001 | p=0.018 |  |
|  |  | MIC | 4 (4, 601) | 0.009 (0.002, 0.044) | 60.52% | p<0.001 | p=0.055 |  |
|  | Quality group | Low Risk | 27 (361, 5622) | 0.218 (0.105, 0.399) | 93.99% | p=0.004 | p<0.001 | p=0.691 |
|  |  | High Risk | 1 (3, 65) | 0.046 (0.015, 0.134) | 0.00% | p<0.001 | p>0.999 |  |
|  |  | Some Risk | 2 (7, 18) | 0.406 (0.211, 0.636) | 0.00% | p=0.426 | p=0.404 |  |
|  | Year group | 2020_2023 | 14 (222, 4182) | 0.301 (0.150, 0.513) | 89.01% | p=0.065 | p<0.001 | p=0.574 |
|  |  | 2013_2019 | 16 (149, 1523) | 0.178 (0.055, 0.449) | 94.37% | p=0.024 | p<0.001 |  |
| ciprofloxacin | Overall | NA | 42 (502.9, 3558) | 0.179 (0.127, 0.246) | 87.16% | p<0.001 | p<0.001 | NA |
|  | countries | Brazil | 2 (8, 318) | 0.030 (0.011, 0.083) | 53.24% | p<0.001 | p=0.144 | p<0.001 |
|  |  | Japan | 1 (90, 268) | 0.336 (0.282, 0.394) | 0.00% | p<0.001 | p>0.999 |  |
|  |  | Tanzania | 1 (0, 1) | 0.250 (0.013, 0.891) | 0.00% | p=0.501 | p>0.999 |  |
|  |  | Serbia | 2 (49, 150) | 0.327 (0.256, 0.406) | 0.00% | p<0.001 | p=0.786 |  |
|  |  | Taiwan | 2 (48, 105) | 0.443 (0.311, 0.583) | 22.78% | p=0.425 | p=0.255 |  |
|  |  | Germany | 1 (1, 66) | 0.015 (0.002, 0.100) | 0.00% | p<0.001 | p>0.999 |  |
|  |  | Ethiopia | 3 (51, 112) | 0.411 (0.242, 0.604) | 69.14% | p=0.368 | p=0.039 |  |
|  |  | Syria | 1 (5, 10) | 0.500 (0.225, 0.775) | 0.00% | p>0.999 | p>0.999 |  |
|  |  | India | 2 (2, 25) | 0.092 (0.023, 0.305) | 0.00% | p=0.002 | p=0.394 |  |
|  |  | Iran | 1 (0, 7) | 0.063 (0.004, 0.539) | 0.00% | p=0.064 | p>0.999 |  |
|  |  | Pakistan | 1 (2, 12) | 0.167 (0.042, 0.477) | 0.00% | p=0.038 | p>0.999 |  |
|  |  | Trinidad & Tobago | 1 (6, 72) | 0.083 (0.038, 0.173) | 0.00% | p<0.001 | p>0.999 |  |
|  |  | Mexico | 1 (10, 18) | 0.556 (0.330, 0.760) | 0.00% | p=0.638 | p>0.999 |  |
|  |  | Lesotho | 1 (2, 9) | 0.222 (0.056, 0.579) | 0.00% | p=0.118 | p>0.999 |  |
|  |  | United States | 1 (16.9, 221) | 0.076 (0.048, 0.120) | 0.00% | p<0.001 | p>0.999 |  |
|  |  | Saudi Arabia | 1 (4, 138) | 0.029 (0.011, 0.075) | 0.00% | p<0.001 | p>0.999 |  |
|  |  | South Africa | 1 (7, 128) | 0.055 (0.026, 0.110) | 0.00% | p<0.001 | p>0.999 |  |
|  | continents | NA | 20 (201, 1898) | 0.177 (0.097, 0.301) | 91.18% | p<0.001 | p<0.001 | p=0.652 |
|  |  | Americas | 5 (40.9, 629) | 0.092 (0.030, 0.250) | 90.68% | p<0.001 | p<0.001 |  |
|  |  | Asia | 9 (151, 565) | 0.212 (0.115, 0.358) | 83.71% | p<0.001 | p<0.001 |  |
|  |  | Africa | 6 (60, 250) | 0.257 (0.097, 0.525) | 88.97% | p=0.074 | p<0.001 |  |
|  |  | Europe | 3 (50, 216) | 0.177 (0.044, 0.501) | 82.56% | p=0.051 | p=0.003 |  |
|  | AST category | MIC | 6 (104, 1585) | 0.047 (0.006, 0.274) | 96.50% | p=0.004 | p<0.001 | p=0.027 |
|  |  | Disk Diffusion | 27 (292, 1146) | 0.242 (0.173, 0.326) | 83.41% | p<0.001 | p<0.001 |  |
|  |  | Other | 4 (49.9, 377) | 0.163 (0.052, 0.407) | 91.15% | p=0.011 | p<0.001 |  |
|  | Quality group | Low Risk | 36 (430.9, 2279) | 0.175 (0.126, 0.239) | 87.87% | p<0.001 | p<0.001 | p=0.087 |
|  |  | High Risk | 2 (17, 1145) | 0.073 (0.001, 0.919) | 98.66% | p=0.316 | p<0.001 |  |
|  |  | Some Risk | 5 (55, 134) | 0.411 (0.163, 0.714) | 73.95% | p=0.581 | p=0.004 |  |
|  | Year group | 2013_2019 | 20 (132, 2180) | 0.124 (0.061, 0.236) | 91.28% | p<0.001 | p<0.001 | p=0.061 |
|  |  | 2020_2023 | 23 (370.9, 1378) | 0.244 (0.174, 0.331) | 86.94% | p<0.001 | p<0.001 |  |
| levofloxacin | Overall | NA | 62 (3756, 46465) | 0.086 (0.068, 0.108) | 51.53% | p<0.001 | p<0.001 | NA |
|  | countries | Nigeria | 1 (9, 33) | 0.273 (0.148, 0.447) | 0.00% | p=0.012 | p>0.999 | p<0.001 |
|  |  | Japan | 4 (1861, 20132) | 0.221 (0.082, 0.474) | 97.58% | p=0.032 | p<0.001 |  |
|  |  | Brazil | 2 (14, 570) | 0.021 (0.006, 0.068) | 46.68% | p<0.001 | p=0.171 |  |
|  |  | Iran | 10 (66, 647) | 0.121 (0.081, 0.177) | 64.08% | p<0.001 | p=0.003 |  |
|  |  | Portugal | 2 (0, 773) | 0.001 (0.000, 0.010) | 0.00% | p<0.001 | p=0.641 |  |
|  |  | Indonesia | 1 (3, 53) | 0.057 (0.018, 0.161) | 0.00% | p<0.001 | p>0.999 |  |
|  |  | Taiwan | 7 (360, 1260) | 0.209 (0.098, 0.391) | 96.20% | p=0.003 | p<0.001 |  |
|  |  | Palestinian Territories | 1 (2, 24) | 0.083 (0.021, 0.279) | 0.00% | p=0.001 | p>0.999 |  |
|  |  | Bosnia & Herzegovina | 1 (0, 17) | 0.028 (0.002, 0.322) | 0.00% | p=0.013 | p>0.999 |  |
|  |  | Canada | 1 (0, 37) | 0.013 (0.001, 0.178) | 0.00% | p=0.002 | p>0.999 |  |
|  |  | Germany | 3 (61, 510) | 0.068 (0.003, 0.619) | 97.80% | p=0.098 | p<0.001 |  |
|  |  | Saudi Arabia | 4 (19, 416) | 0.050 (0.032, 0.076) | 0.00% | p<0.001 | p=0.478 |  |
|  |  | Italy | 2 (14, 455) | 0.031 (0.018, 0.051) | 0.00% | p<0.001 | p=0.824 |  |
|  |  | Egypt | 1 (0, 100) | 0.005 (0.000, 0.074) | 0.00% | p<0.001 | p>0.999 |  |
|  |  | Syria | 1 (3, 10) | 0.300 (0.100, 0.624) | 0.00% | p=0.220 | p>0.999 |  |
|  |  | United States | 1 (7, 803) | 0.009 (0.004, 0.018) | 0.00% | p<0.001 | p>0.999 |  |
|  |  | Hungary | 1 (3, 96) | 0.031 (0.010, 0.092) | 0.00% | p<0.001 | p>0.999 |  |
|  |  | Iceland | 1 (0, 98) | 0.005 (0.000, 0.076) | 0.00% | p<0.001 | p>0.999 |  |
|  |  | Slovenia | 1 (0, 171) | 0.003 (0.000, 0.045) | 0.00% | p<0.001 | p>0.999 |  |
|  |  | Mexico | 2 (6, 591) | 0.011 (0.005, 0.024) | 0.00% | p<0.001 | p=0.505 |  |
|  |  | Ethiopia | 1 (4, 83) | 0.048 (0.018, 0.121) | 0.00% | p<0.001 | p>0.999 |  |
|  |  | Colombia | 1 (17, 671) | 0.025 (0.016, 0.040) | 0.00% | p<0.001 | p>0.999 |  |
|  |  | Thailand | 1 (2, 109) | 0.018 (0.005, 0.070) | 0.00% | p<0.001 | p>0.999 |  |
|  |  | France | 2 (66, 8893) | 0.007 (0.006, 0.010) | 0.00% | p<0.001 | p=0.608 |  |
|  |  | Trinidad & Tobago | 1 (6, 72) | 0.083 (0.038, 0.173) | 0.00% | p<0.001 | p>0.999 |  |
|  |  | United Arab Emirates | 1 (16, 158) | 0.101 (0.063, 0.159) | 0.00% | p<0.001 | p>0.999 |  |
|  |  | Argentina | 1 (24, 194) | 0.124 (0.084, 0.178) | 0.00% | p<0.001 | p>0.999 |  |
|  |  | Malaysia | 1 (0, 103) | 0.005 (0.000, 0.072) | 0.00% | p<0.001 | p>0.999 |  |
|  |  | Vietnam | 1 (74, 260) | 0.285 (0.233, 0.342) | 0.00% | p<0.001 | p>0.999 |  |
|  | continents | Africa | 3 (13, 216) | 0.061 (0.009, 0.324) | 87.31% | p=0.007 | p<0.001 | p<0.001 |
|  |  | Asia | 34 (2418, 23247) | 0.127 (0.088, 0.178) | 96.27% | p<0.001 | p<0.001 |  |
|  |  | NA | 65 (1101, 8986) | 0.105 (0.075, 0.147) | 94.57% | p<0.001 | p<0.001 |  |
|  |  | Europe | 14 (150, 11078) | 0.017 (0.005, 0.062) | 97.26% | p<0.001 | p<0.001 |  |
|  |  | Americas | 9 (74, 2938) | 0.026 (0.012, 0.057) | 88.73% | p<0.001 | p<0.001 |  |
|  | AST category | MIC | 52 (2405, 29598) | 0.068 (0.048, 0.095) | 95.68% | p<0.001 | p<0.001 | p=0.302 |
|  |  | Disk Diffusion | 46 (965, 14252) | 0.095 (0.059, 0.151) | 96.79% | p<0.001 | p<0.001 |  |
|  |  | Combined method | 4 (28, 383) | 0.045 (0.013, 0.139) | 72.00% | p<0.001 | p=0.013 |  |
|  |  | Other | 9 (66, 549) | 0.135 (0.082, 0.215) | 69.25% | p<0.001 | p=0.001 |  |
|  | Quality group | Low Risk | 110 (1752, 22954) | 0.082 (0.061, 0.109) | 95.73% | p<0.001 | p<0.001 | p=0.971 |
|  |  | Some Risk | 11 (1910, 22766) | 0.092 (0.039, 0.203) | 96.93% | p<0.001 | p<0.001 |  |
|  |  | High Risk | 4 (94, 745) | 0.080 (0.013, 0.371) | 96.53% | p=0.012 | p<0.001 |  |
|  | Year group | 2020_2023 | 75 (2972, 26883) | 0.131 (0.100, 0.170) | 96.02% | p<0.001 | p<0.001 | p<0.001 |
|  |  | 2013_2019 | 50 (784, 19582) | 0.041 (0.024, 0.068) | 96.62% | p<0.001 | p<0.001 |  |
| gentamicin | Overall | NA | 32 (649, 12155) | 0.190 (0.080, 0.389) | 97.29% | p=0.004 | p<0.001 | NA |
|  | countries | Cameroon | 1 (5, 17) | 0.294 (0.128, 0.542) | 0.00% | p=0.100 | p>0.999 | p=0.006 |
|  |  | Portugal | 2 (1, 773) | 0.003 (0.001, 0.013) | 0.00% | p<0.001 | p=0.346 |  |
|  |  | Tanzania | 1 (0, 1) | 0.250 (0.013, 0.891) | 0.00% | p=0.501 | p>0.999 |  |
|  |  | Serbia | 2 (109, 150) | 0.727 (0.650, 0.792) | 0.00% | p<0.001 | p=0.996 |  |
|  |  | Germany | 1 (8, 66) | 0.121 (0.062, 0.224) | 0.00% | p<0.001 | p>0.999 |  |
|  |  | Syria | 1 (7, 10) | 0.700 (0.376, 0.900) | 0.00% | p=0.220 | p>0.999 |  |
|  |  | Ethiopia | 1 (22, 135) | 0.163 (0.110, 0.235) | 0.00% | p<0.001 | p>0.999 |  |
|  |  | Iceland | 1 (0, 98) | 0.005 (0.000, 0.076) | 0.00% | p<0.001 | p>0.999 |  |
|  |  | Taiwan | 1 (0, 14) | 0.033 (0.002, 0.366) | 0.00% | p=0.019 | p>0.999 |  |
|  |  | Iran | 2 (215, 279) | 0.653 (0.093, 0.972) | 98.52% | p=0.669 | p<0.001 |  |
|  |  | France | 1 (24, 8757) | 0.003 (0.002, 0.004) | 0.00% | p<0.001 | p>0.999 |  |
|  |  | Malawi | 1 (0, 9) | 0.050 (0.003, 0.475) | 0.00% | p=0.042 | p>0.999 |  |
|  |  | Mexico | 1 (4, 18) | 0.222 (0.086, 0.465) | 0.00% | p=0.027 | p>0.999 |  |
|  |  | Saudi Arabia | 1 (0, 138) | 0.004 (0.000, 0.055) | 0.00% | p<0.001 | p>0.999 |  |
|  |  | Iraq | 1 (7, 16) | 0.438 (0.225, 0.676) | 0.00% | p=0.618 | p>0.999 |  |
|  |  | South Africa | 1 (0, 128) | 0.004 (0.000, 0.059) | 0.00% | p<0.001 | p>0.999 |  |
|  | continents | NA | 13 (247, 1546) | 0.395 (0.151, 0.706) | 92.01% | p=0.519 | p<0.001 | p=0.353 |
|  |  | Africa | 5 (27, 290) | 0.124 (0.044, 0.302) | 62.21% | p<0.001 | p=0.032 |  |
|  |  | Europe | 7 (142, 9844) | 0.036 (0.002, 0.438) | 99.08% | p=0.034 | p<0.001 |  |
|  |  | Asia | 6 (229, 457) | 0.307 (0.074, 0.711) | 95.13% | p=0.353 | p<0.001 |  |
|  |  | Americas | 1 (4, 18) | 0.222 (0.086, 0.465) | 0.00% | p=0.027 | p>0.999 |  |
|  | AST category | Disk Diffusion | 20 (222, 10301) | 0.109 (0.033, 0.306) | 97.47% | p=0.001 | p<0.001 | p=0.301 |
|  |  | MIC | 7 (348, 1659) | 0.402 (0.060, 0.877) | 97.82% | p=0.743 | p<0.001 |  |
|  |  | Other | 2 (19, 36) | 0.527 (0.365, 0.684) | 0.00% | p=0.746 | p=0.334 |  |
|  | Quality group | Low Risk | 26 (557, 10897) | 0.174 (0.064, 0.394) | 97.63% | p=0.007 | p<0.001 | p=0.215 |
|  |  | Some Risk | 3 (78, 99) | 0.681 (0.204, 0.947) | 86.19% | p=0.484 | p<0.001 |  |
|  |  | High Risk | 3 (14, 1159) | 0.044 (0.000, 0.863) | 96.94% | p=0.220 | p<0.001 |  |
|  | Year group | 2013_2019 | 19 (491, 11574) | 0.142 (0.031, 0.466) | 98.31% | p=0.034 | p<0.001 | p=0.456 |
|  |  | 2020_2023 | 13 (158, 581) | 0.298 (0.168, 0.473) | 84.41% | p=0.025 | p<0.001 |  |
| linezolid | Overall | NA | 45 (17, 18117) | 0.008 (0.006, 0.011) | 0.00% | p<0.001 | p>0.999 | NA |
| daptomycin | Overall | NA | 22 (4, 10690) | 0.003 (0.002, 0.007) | 5.23% | p<0.001 | p=0.390 | NA |
| tigecycline | Overall | NA | 30 (0, 3066) | 0.007 (0.004, 0.012) | 0.00% | p<0.001 | p=0.996 | NA |
| nitrofurantoin | Overall | NA | 12 (73, 627) | 0.124 (0.055, 0.258) | 83.52% | p<0.001 | p<0.001 | NA |
|  | countries | Bahrain | 1 (0, 123) | 0.004 (0.000, 0.061) | 0.00% | p<0.001 | p>0.999 | p=0.902 |
|  |  | Iran | 1 (0, 7) | 0.063 (0.004, 0.539) | 0.00% | p=0.064 | p>0.999 |  |
|  |  | Trinidad & Tobago | 1 (33, 72) | 0.458 (0.347, 0.574) | 0.00% | p=0.480 | p>0.999 |  |
|  |  | Saudi Arabia | 2 (20, 220) | 0.041 (0.001, 0.772) | 89.73% | p=0.157 | p=0.002 |  |
|  |  | Mexico | 1 (3, 18) | 0.167 (0.055, 0.409) | 0.00% | p=0.011 | p>0.999 |  |
|  |  | Lesotho | 1 (2, 9) | 0.222 (0.056, 0.579) | 0.00% | p=0.118 | p>0.999 |  |
|  |  | Iraq | 1 (7, 16) | 0.438 (0.225, 0.676) | 0.00% | p=0.618 | p>0.999 |  |
|  | continents | Europe | 1 (3, 65) | 0.046 (0.015, 0.134) | 0.00% | p<0.001 | p>0.999 | p=0.750 |
|  |  | NA | 3 (5, 97) | 0.105 (0.001, 0.907) | 86.42% | p=0.343 | p<0.001 |  |
|  |  | Asia | 5 (27, 366) | 0.075 (0.016, 0.287) | 83.11% | p=0.002 | p<0.001 |  |
|  |  | Americas | 2 (36, 90) | 0.317 (0.103, 0.651) | 78.08% | p=0.280 | p=0.033 |  |
|  |  | Africa | 1 (2, 9) | 0.222 (0.056, 0.579) | 0.00% | p=0.118 | p>0.999 |  |
|  | AST category | Disk Diffusion | 9 (43, 464) | 0.083 (0.020, 0.284) | 84.36% | p=0.001 | p<0.001 | p=0.222 |
|  |  | Other | 2 (27, 98) | 0.310 (0.163, 0.510) | 58.67% | p=0.062 | p=0.120 |  |
|  | Quality group | High Risk | 1 (3, 65) | 0.046 (0.015, 0.134) | 0.00% | p<0.001 | p>0.999 | p=0.347 |
|  |  | Low Risk | 10 (63, 546) | 0.113 (0.044, 0.262) | 82.83% | p<0.001 | p<0.001 |  |
|  |  | Some Risk | 1 (7, 16) | 0.438 (0.225, 0.676) | 0.00% | p=0.618 | p>0.999 |  |
|  | Year group | 2013_2019 | 7 (28, 442) | 0.072 (0.024, 0.194) | 76.53% | p<0.001 | p<0.001 | p=0.101 |
|  |  | 2020_2023 | 5 (45, 185) | 0.256 (0.073, 0.602) | 80.48% | p=0.158 | p<0.001 |  |
| ceftaroline | Overall | NA | 4 (0, 176) | 0.012 (0.003, 0.048) | 0.00% | p<0.001 | p=0.933 | NA |
| tedizolid | Overall | NA | 4 (0, 5213) | 0.001 (0.000, 0.008) | 49.62% | p<0.001 | p=0.114 | NA |
| cefepime | Overall | NA | 25 (179, 5231) | 0.063 (0.027, 0.142) | 89.76% | p<0.001 | p<0.001 | NA |
|  | countries | Iran | 5 (19, 166) | 0.106 (0.015, 0.481) | 87.21% | p=0.042 | p<0.001 | p=0.336 |
|  |  | Taiwan | 2 (13, 438) | 0.027 (0.006, 0.111) | 81.11% | p<0.001 | p=0.021 |  |
|  |  | Bosnia & Herzegovina | 1 (0, 17) | 0.028 (0.002, 0.322) | 0.00% | p=0.013 | p>0.999 |  |
|  |  | Japan | 2 (77, 117) | 0.581 (0.000, 1.000) | 95.47% | p=0.945 | p<0.001 |  |
|  |  | Syria | 1 (7, 10) | 0.700 (0.376, 0.900) | 0.00% | p=0.220 | p>0.999 |  |
|  |  | Egypt | 1 (11, 92) | 0.120 (0.067, 0.203) | 0.00% | p<0.001 | p>0.999 |  |
|  |  | Thailand | 1 (0, 109) | 0.005 (0.000, 0.068) | 0.00% | p<0.001 | p>0.999 |  |
|  |  | Saudi Arabia | 2 (0, 152) | 0.011 (0.001, 0.091) | 19.96% | p<0.001 | p=0.264 |  |
|  | continents | Asia | 13 (116, 992) | 0.085 (0.023, 0.273) | 90.44% | p<0.001 | p<0.001 | p=0.404 |
|  |  | NA | 10 (31, 4094) | 0.026 (0.005, 0.131) | 88.51% | p<0.001 | p<0.001 |  |
|  |  | Africa | 2 (32, 128) | 0.303 (0.042, 0.811) | 96.00% | p=0.475 | p<0.001 |  |
|  |  | Europe | 1 (0, 17) | 0.028 (0.002, 0.322) | 0.00% | p=0.013 | p>0.999 |  |
|  | AST category | Disk Diffusion | 13 (64, 870) | 0.084 (0.027, 0.231) | 90.86% | p<0.001 | p<0.001 | p=0.974 |
|  |  | MIC | 7 (87, 524) | 0.058 (0.007, 0.333) | 84.33% | p=0.009 | p<0.001 |  |
|  |  | Other | 3 (28, 3634) | 0.056 (0.001, 0.847) | 96.58% | p=0.222 | p<0.001 |  |
|  |  | Combined method | 1 (0, 17) | 0.028 (0.002, 0.322) | 0.00% | p=0.013 | p>0.999 |  |
|  | Quality group | Some Risk | 3 (32, 52) | 0.590 (0.119, 0.939) | 83.71% | p=0.763 | p=0.002 | p=0.008 |
|  |  | Low Risk | 23 (147, 5179) | 0.044 (0.018, 0.100) | 88.73% | p<0.001 | p<0.001 |  |
|  | Year group | 2020_2023 | 20 (169, 4839) | 0.079 (0.028, 0.201) | 91.79% | p<0.001 | p<0.001 | p=0.281 |
|  |  | 2013_2019 | 6 (10, 392) | 0.044 (0.025, 0.074) | 0.00% | p<0.001 | p=0.528 |  |
| moxifloxacin | Overall | NA | 20 (110, 1431) | 0.063 (0.033, 0.120) | 86.10% | p<0.001 | p<0.001 | NA |
|  | countries | Brazil | 2 (0, 318) | 0.004 (0.001, 0.029) | 0.00% | p<0.001 | p=0.437 | p<0.001 |
|  |  | Iran | 1 (8, 87) | 0.092 (0.047, 0.173) | 0.00% | p<0.001 | p>0.999 |  |
|  |  | Hungary | 1 (3, 96) | 0.031 (0.010, 0.092) | 0.00% | p<0.001 | p>0.999 |  |
|  |  | Mexico | 1 (0, 17) | 0.028 (0.002, 0.322) | 0.00% | p=0.013 | p>0.999 |  |
|  |  | Taiwan | 1 (41, 92) | 0.446 (0.348, 0.548) | 0.00% | p=0.298 | p>0.999 |  |
|  | continents | NA | 13 (58, 756) | 0.082 (0.040, 0.162) | 79.04% | p<0.001 | p<0.001 | p=0.033 |
|  |  | Europe | 2 (3, 161) | 0.025 (0.009, 0.070) | 0.00% | p<0.001 | p=0.348 |  |
|  |  | Americas | 3 (0, 335) | 0.008 (0.002, 0.038) | 0.00% | p<0.001 | p=0.401 |  |
|  |  | Asia | 2 (49, 179) | 0.226 (0.037, 0.690) | 95.77% | p=0.234 | p<0.001 |  |
|  | AST category | MIC | 7 (25, 500) | 0.060 (0.016, 0.198) | 85.47% | p<0.001 | p<0.001 | p=0.894 |
|  |  | Disk Diffusion | 6 (19, 297) | 0.081 (0.046, 0.138) | 24.37% | p<0.001 | p=0.251 |  |
|  |  | Other | 2 (23, 136) | 0.075 (0.003, 0.690) | 82.29% | p=0.137 | p=0.018 |  |
|  | Quality group | Low Risk | 17 (67, 1256) | 0.057 (0.029, 0.110) | 80.22% | p<0.001 | p<0.001 | p=0.087 |
|  |  | High Risk | 1 (0, 65) | 0.008 (0.000, 0.110) | 0.00% | p<0.001 | p>0.999 |  |
|  |  | Some Risk | 2 (43, 110) | 0.267 (0.057, 0.689) | 82.49% | p=0.273 | p=0.017 |  |
|  | Year group | 2013_2019 | 7 (9, 686) | 0.018 (0.005, 0.062) | 63.94% | p<0.001 | p=0.011 | p=0.013 |
|  |  | 2020_2023 | 13 (101, 745) | 0.109 (0.053, 0.208) | 86.71% | p<0.001 | p<0.001 |  |
| oxacillin | Overall | NA | 9 (41, 1069) | 0.062 (0.012, 0.261) | 85.61% | p=0.001 | p<0.001 | NA |
|  | countries | Cameroon | 1 (0, 17) | 0.028 (0.002, 0.322) | 0.00% | p=0.013 | p>0.999 | p=0.152 |
|  |  | Japan | 2 (2, 677) | 0.005 (0.001, 0.036) | 51.20% | p<0.001 | p=0.152 |  |
|  |  | Thailand | 1 (0, 100) | 0.005 (0.000, 0.074) | 0.00% | p<0.001 | p>0.999 |  |
|  |  | Argentina | 1 (24, 194) | 0.124 (0.084, 0.178) | 0.00% | p<0.001 | p>0.999 |  |
|  | continents | Africa | 1 (0, 17) | 0.028 (0.002, 0.322) | 0.00% | p=0.013 | p>0.999 | p=0.130 |
|  |  | Asia | 3 (2, 777) | 0.006 (0.002, 0.019) | 3.63% | p<0.001 | p=0.354 |  |
|  |  | NA | 4 (15, 81) | 0.385 (0.014, 0.965) | 85.80% | p=0.807 | p<0.001 |  |
|  |  | Americas | 1 (24, 194) | 0.124 (0.084, 0.178) | 0.00% | p<0.001 | p>0.999 |  |
|  | AST category | Disk Diffusion | 3 (6, 29) | 0.233 (0.007, 0.925) | 80.28% | p=0.529 | p=0.006 | p=0.080 |
|  |  | MIC | 3 (2, 360) | 0.009 (0.003, 0.026) | 0.00% | p<0.001 | p=0.904 |  |
|  |  | Combined method | 1 (24, 194) | 0.124 (0.084, 0.178) | 0.00% | p<0.001 | p>0.999 |  |
|  | Quality group | Some Risk | 1 (0, 17) | 0.028 (0.002, 0.322) | 0.00% | p=0.013 | p>0.999 | p=0.716 |
|  |  | High Risk | 2 (11, 209) | 0.283 (0.000, 0.998) | 95.41% | p=0.805 | p<0.001 |  |
|  |  | Low Risk | 6 (30, 843) | 0.043 (0.005, 0.279) | 83.69% | p=0.005 | p<0.001 |  |
|  | Year group | 2013_2019 | 4 (17, 232) | 0.319 (0.008, 0.965) | 91.48% | p=0.715 | p<0.001 | p=0.114 |
|  |  | 2020_2023 | 5 (24, 837) | 0.016 (0.002, 0.121) | 80.28% | p<0.001 | p<0.001 |  |
| teicoplanin | Overall | NA | 8 (3, 1274) | 0.007 (0.003, 0.021) | 0.00% | p<0.001 | p=0.882 | NA |
| Q/D | Overall | NA | 15 (67, 1260) | 0.014 (0.003, 0.065) | 0.00% | p<0.001 | p=0.472 | NA |
| chloramphenicol | Overall | NA | 57 (500, 10245) | 0.072 (0.048, 0.107) | 93.25% | p<0.001 | p<0.001 | NA |
|  | countries | Nigeria | 1 (8, 33) | 0.242 (0.126, 0.415) | 0.00% | p=0.005 | p>0.999 | p<0.001 |
|  |  | Iran | 5 (94, 343) | 0.247 (0.088, 0.525) | 85.08% | p=0.072 | p<0.001 |  |
|  |  | Brazil | 2 (19, 570) | 0.034 (0.022, 0.052) | 0.00% | p<0.001 | p=0.425 |  |
|  |  | Portugal | 2 (3, 773) | 0.005 (0.000, 0.062) | 68.86% | p<0.001 | p=0.073 |  |
|  |  | Taiwan | 3 (41, 451) | 0.197 (0.048, 0.543) | 93.27% | p=0.081 | p<0.001 |  |
|  |  | Namibia | 1 (0, 18) | 0.026 (0.002, 0.310) | 0.00% | p=0.012 | p>0.999 |  |
|  |  | Ethiopia | 4 (9, 193) | 0.062 (0.021, 0.167) | 52.71% | p<0.001 | p=0.096 |  |
|  |  | Serbia | 1 (0, 432) | 0.001 (0.000, 0.018) | 0.00% | p<0.001 | p>0.999 |  |
|  |  | Egypt | 1 (1, 100) | 0.010 (0.001, 0.068) | 0.00% | p<0.001 | p>0.999 |  |
|  |  | Iceland | 1 (0, 98) | 0.005 (0.000, 0.076) | 0.00% | p<0.001 | p>0.999 |  |
|  |  | Canada | 2 (30, 3236) | 0.007 (0.001, 0.038) | 91.10% | p<0.001 | p<0.001 |  |
|  |  | Thailand | 1 (2, 109) | 0.018 (0.005, 0.070) | 0.00% | p<0.001 | p>0.999 |  |
|  |  | Pakistan | 1 (0, 12) | 0.038 (0.002, 0.403) | 0.00% | p=0.026 | p>0.999 |  |
|  |  | Saudi Arabia | 1 (0, 14) | 0.033 (0.002, 0.366) | 0.00% | p=0.019 | p>0.999 |  |
|  |  | India | 1 (0, 6) | 0.071 (0.004, 0.577) | 0.00% | p=0.081 | p>0.999 |  |
|  |  | Gambia | 1 (4, 133) | 0.030 (0.011, 0.077) | 0.00% | p<0.001 | p>0.999 |  |
|  |  | Malaysia | 1 (0, 103) | 0.005 (0.000, 0.072) | 0.00% | p<0.001 | p>0.999 |  |
|  |  | Vietnam | 1 (22, 42) | 0.524 (0.375, 0.668) | 0.00% | p=0.758 | p>0.999 |  |
|  |  | South Africa | 1 (21, 128) | 0.164 (0.109, 0.239) | 0.00% | p<0.001 | p>0.999 |  |
|  |  | Zimbabwe | 2 (17, 86) | 0.150 (0.017, 0.647) | 89.08% | p=0.147 | p=0.002 |  |
|  | continents | Africa | 12 (67, 727) | 0.091 (0.049, 0.161) | 78.75% | p<0.001 | p<0.001 | p<0.001 |
|  |  | Asia | 14 (159, 1080) | 0.141 (0.065, 0.280) | 91.83% | p<0.001 | p<0.001 |  |
|  |  | NA | 23 (222, 3329) | 0.096 (0.056, 0.160) | 91.15% | p<0.001 | p<0.001 |  |
|  |  | Americas | 4 (49, 3806) | 0.016 (0.007, 0.039) | 87.64% | p<0.001 | p<0.001 |  |
|  |  | Europe | 4 (3, 1303) | 0.004 (0.001, 0.018) | 42.09% | p<0.001 | p=0.159 |  |
|  | AST category | MIC | 15 (181, 1388) | 0.093 (0.051, 0.162) | 90.11% | p<0.001 | p<0.001 | p=0.740 |
|  |  | Disk Diffusion | 37 (308, 6940) | 0.080 (0.047, 0.134) | 93.82% | p<0.001 | p<0.001 |  |
|  |  | Other | 2 (11, 1587) | 0.030 (0.000, 0.936) | 98.26% | p=0.267 | p<0.001 |  |
|  | Quality group | Low Risk | 51 (413, 9650) | 0.060 (0.039, 0.090) | 92.65% | p<0.001 | p<0.001 | p=0.005 |
|  |  | Some Risk | 6 (87, 595) | 0.329 (0.086, 0.719) | 95.60% | p=0.397 | p<0.001 |  |
|  | Year group | 2020_2023 | 37 (260, 4313) | 0.096 (0.061, 0.149) | 89.78% | p<0.001 | p<0.001 | p=0.099 |
|  |  | 2013_2019 | 20 (240, 5932) | 0.044 (0.020, 0.093) | 95.85% | p<0.001 | p<0.001 |  |
| cefditoren | Overall | NA | 4 (44, 20636) | 0.003 (0.000, 0.238) | 95.23% | p=0.014 | p<0.001 | NA |
|  | continents | Asia | 3 (44, 20576) | 0.002 (0.000, 0.510) | 96.52% | p=0.052 | p<0.001 | p=0.834 |
|  |  | NA | 1 (0, 60) | 0.008 (0.001, 0.118) | 0.00% | p<0.001 | p>0.999 |  |
|  | Quality group | Some Risk | 1 (0, 19899) | 0.000 (0.000, 0.000) | 0.00% | p<0.001 | p>0.999 | p<0.001 |
|  |  | Low Risk | 2 (0, 537) | 0.003 (0.000, 0.022) | 5.84% | p<0.001 | p=0.303 |  |
|  |  | High Risk | 1 (44, 200) | 0.220 (0.168, 0.283) | 0.00% | p<0.001 | p>0.999 |  |
|  | Year group | 2020_2023 | 2 (0, 20376) | 0.000 (0.000, 0.006) | 71.23% | p<0.001 | p=0.062 | p=0.019 |
|  |  | 2013_2019 | 2 (44, 260) | 0.060 (0.002, 0.661) | 83.58% | p=0.115 | p=0.014 |  |
| norfloxacins | Overall | NA | 9 (168, 865) | 0.157 (0.084, 0.274) | 87.99% | p<0.001 | p<0.001 | NA |
|  | countries | Brazil | 2 (119, 318) | 0.375 (0.323, 0.429) | 0.00% | p<0.001 | p=0.430 | p<0.001 |
|  |  | Bahrain | 1 (0, 123) | 0.004 (0.000, 0.061) | 0.00% | p<0.001 | p>0.999 |  |
|  |  | Iran | 1 (0, 7) | 0.063 (0.004, 0.539) | 0.00% | p=0.064 | p>0.999 |  |
|  |  | Trinidad & Tobago | 1 (7, 72) | 0.097 (0.047, 0.190) | 0.00% | p<0.001 | p>0.999 |  |
|  |  | Taiwan | 1 (0, 92) | 0.005 (0.000, 0.080) | 0.00% | p<0.001 | p>0.999 |  |
|  |  | Argentina | 1 (25, 194) | 0.129 (0.089, 0.184) | 0.00% | p<0.001 | p>0.999 |  |
|  | continents | Americas | 4 (151, 584) | 0.214 (0.102, 0.395) | 93.36% | p=0.004 | p<0.001 | p=0.005 |
|  |  | Asia | 3 (0, 222) | 0.011 (0.002, 0.058) | 11.91% | p<0.001 | p=0.321 |  |
|  |  | NA | 2 (17, 59) | 0.290 (0.189, 0.419) | 0.00% | p=0.002 | p=0.504 |  |
|  | AST category | MIC | 1 (18, 55) | 0.327 (0.217, 0.461) | 0.00% | p=0.012 | p>0.999 | p=0.555 |
|  |  | Disk Diffusion | 5 (24, 261) | 0.112 (0.038, 0.289) | 76.33% | p<0.001 | p=0.002 |  |
|  |  | Combined method | 1 (25, 194) | 0.129 (0.089, 0.184) | 0.00% | p<0.001 | p>0.999 |  |
|  | Quality group | Low Risk | 8 (168, 773) | 0.185 (0.102, 0.310) | 87.84% | p<0.001 | p<0.001 | p=0.026 |
|  |  | Some Risk | 1 (0, 92) | 0.005 (0.000, 0.080) | 0.00% | p<0.001 | p>0.999 |  |
|  | Year group | 2020_2023 | 5 (65, 462) | 0.161 (0.080, 0.299) | 84.67% | p<0.001 | p<0.001 | p=0.984 |
|  |  | 2013_2019 | 4 (103, 403) | 0.107 (0.019, 0.432) | 81.16% | p=0.025 | p=0.001 |  |
| AMC | Overall | NA | 6 (31, 2033) | 0.196 (0.023, 0.713) | 75.79% | p=0.696 | p<0.001 | NA |
|  | continents | Africa | 1 (0, 17) | 0.028 (0.002, 0.322) | 0.00% | p=0.013 | p>0.999 | p=0.930 |
|  |  | NA | 4 (25, 1988) | 0.280 (0.004, 0.973) | 92.66% | p=0.683 | p<0.001 |  |
|  |  | Asia | 1 (5, 10) | 0.500 (0.225, 0.775) | 0.00% | p>0.999 | p>0.999 |  |
|  |  | Americas | 1 (1, 18) | 0.056 (0.008, 0.307) | 0.00% | p=0.006 | p>0.999 |  |
|  | AST category | Disk Diffusion | 5 (11, 53) | 0.276 (0.075, 0.641) | 67.39% | p=0.221 | p=0.015 | p=0.030 |
|  |  | Other | 1 (20, 20) | 0.976 (0.713, 0.999) | 0.00% | p=0.009 | p>0.999 |  |
|  | Quality group | Some Risk | 2 (1, 19) | 0.146 (0.005, 0.847) | 67.90% | p=0.319 | p=0.078 | p=0.237 |
|  |  | High Risk | 1 (20, 20) | 0.976 (0.713, 0.999) | 0.00% | p=0.009 | p>0.999 |  |
|  |  | Low Risk | 4 (10, 1994) | 0.082 (0.004, 0.675) | 91.64% | p=0.132 | p<0.001 |  |
|  | Year group | 2013_2019 | 4 (25, 61) | 0.374 (0.031, 0.919) | 85.18% | p=0.732 | p<0.001 | p=0.439 |
|  |  | 2020_2023 | 3 (6, 1972) | 0.064 (0.001, 0.903) | 93.18% | p=0.285 | p<0.001 |  |
| cefoxitine | Overall | NA | 4 (14, 184) | 0.186 (0.031, 0.622) | 81.58% | p=0.142 | p<0.001 | NA |
|  | continents | Africa | 1 (3, 17) | 0.176 (0.058, 0.427) | 0.00% | p=0.015 | p>0.999 | p=0.216 |
|  |  | NA | 2 (11, 29) | 0.598 (0.034, 0.984) | 83.71% | p=0.836 | p=0.013 |  |
|  |  | Asia | 1 (0, 138) | 0.004 (0.000, 0.055) | 0.00% | p<0.001 | p>0.999 |  |
|  | Quality group | Some Risk | 1 (3, 17) | 0.176 (0.058, 0.427) | 0.00% | p=0.015 | p>0.999 | p=0.980 |
|  |  | Low Risk | 3 (11, 167) | 0.189 (0.006, 0.895) | 87.68% | p=0.428 | p<0.001 |  |
|  | Year group | 2013_2019 | 3 (9, 161) | 0.175 (0.005, 0.897) | 87.60% | p=0.413 | p<0.001 | p=0.941 |
|  |  | 2020_2023 | 1 (5, 23) | 0.217 (0.093, 0.428) | 0.00% | p=0.011 | p>0.999 |  |
| norfloxacin | Overall | NA | 4 (18, 499) | 0.096 (0.006, 0.648) | 91.42% | p=0.123 | p<0.001 | NA |
|  | Year group | 2013_2019 | 2 (13, 57) | 0.176 (0.001, 0.975) | 91.88% | p=0.562 | p<0.001 | p=0.700 |
|  |  | 2020_2023 | 2 (5, 442) | 0.037 (0.000, 0.966) | 94.75% | p=0.334 | p<0.001 |  |
| ofloxacin | Overall | NA | 6 (98, 292) | 0.273 (0.049, 0.731) | 94.61% | p=0.332 | p<0.001 | NA |
|  | countries | Taiwan | 3 (8, 174) | 0.058 (0.021, 0.151) | 50.73% | p<0.001 | p=0.131 | p=0.009 |
|  |  | Syria | 1 (5, 10) | 0.500 (0.225, 0.775) | 0.00% | p>0.999 | p>0.999 |  |
|  | continents | Asia | 4 (13, 184) | 0.112 (0.025, 0.382) | 84.01% | p=0.011 | p<0.001 | p=0.012 |
|  |  | NA | 2 (85, 108) | 0.784 (0.697, 0.852) | 0.00% | p<0.001 | p=0.835 |  |
|  | AST category | Disk Diffusion | 4 (13, 93) | 0.268 (0.062, 0.668) | 81.04% | p=0.248 | p=0.001 | p=0.081 |
|  |  | Other | 1 (2, 93) | 0.022 (0.005, 0.082) | 0.00% | p<0.001 | p>0.999 |  |
|  |  | MIC | 1 (83, 106) | 0.783 (0.695, 0.851) | 0.00% | p<0.001 | p>0.999 |  |
|  | Quality group | LOW Risk | 5 (96, 290) | 0.203 (0.029, 0.689) | 95.65% | p=0.216 | p<0.001 | p=0.330 |
|  |  | Some Risk | 1 (2, 2) | 0.833 (0.194, 0.990) | 0.00% | p=0.299 | p>0.999 |  |
|  | Year group | 2013_2019 | 2 (6, 81) | 0.084 (0.032, 0.200) | 24.88% | p<0.001 | p=0.249 | p=0.306 |
|  |  | 2020_2023 | 4 (92, 211) | 0.422 (0.061, 0.892) | 93.70% | p=0.800 | p<0.001 |  |
| amikacin | Overall | NA | 9 (652, 9033) | 0.196 (0.076, 0.422) | 73.85% | p<0.001 | p<0.001 | NA |
|  | continents | NA | 4 (68, 79) | 0.553 (0.053, 0.964) | 81.77% | p=0.893 | p<0.001 | p=0.632 |
|  |  | Africa | 1 (1, 15) | 0.067 (0.009, 0.352) | 0.00% | p=0.011 | p>0.999 |  |
|  |  | Asia | 3 (8, 164) | 0.120 (0.017, 0.522) | 81.12% | p=0.060 | p=0.005 |  |
|  |  | Europe | 1 (572, 8757) | 0.065 (0.060, 0.071) | 0.00% | p<0.001 | p>0.999 |  |
|  |  | Americas | 1 (3, 18) | 0.167 (0.055, 0.409) | 0.00% | p=0.011 | p>0.999 |  |
|  | AST category | Disk Diffusion | 8 (581, 8951) | 0.111 (0.050, 0.229) | 62.75% | p<0.001 | p=0.009 | p<0.001 |
|  |  | MIC | 1 (66, 66) | 0.993 (0.892, 1.000) | 0.00% | p<0.001 | p>0.999 |  |
|  |  | Other | 1 (5, 16) | 0.312 (0.136, 0.567) | 0.00% | p=0.144 | p>0.999 |  |
|  | Quality group | Low Risk | 7 (580, 8949) | 0.097 (0.043, 0.201) | 60.74% | p<0.001 | p=0.018 | p=0.004 |
|  |  | Some Risk | 3 (72, 84) | 0.771 (0.109, 0.989) | 85.70% | p=0.473 | p<0.001 |  |
|  | Year group | 2020_2023 | 5 (10, 39) | 0.283 (0.160, 0.449) | 0.00% | p=0.012 | p=0.750 | p=0.541 |
|  |  | 2013_2019 | 5 (642, 8994) | 0.158 (0.028, 0.547) | 88.70% | p=0.078 | p<0.001 |  |
| nalidixicacid | Overall | NA | 3 (102, 135) | 0.749 (0.420, 0.925) | 3.10% | p=0.764 | p=0.356 | NA |
| Abbreviation: K, Number of reports; n, Number of resistant isolates; N, Number of total isolates; LCI, 95% Lower Limit Confidence Interval; HCI, 95% Higher Limit Confidence Interval; P1, P-value of difference from zero resistance rate; P2, P-value of heterogeneity between reports; P3, P-value of difference between groups; NA, Not Applicable; TMP/SMX, trimethoprim.sulfamethoxazole; SAM, ampicillin.sulbactam; Q/D, quinupristin.dalfopristin; AMC, amoxycillin.clavulanate. | | | | | | | | |

**Supplementary Table 3: Risk of Bias Assessment for Included Studies**

| ID | Author | Were the criteria for inclusion in the sample clearly defined? | Were the study subjects and the setting described in detail? | Were objective, standard criteria used for measurement of the condition? | Were confounding factors identified? | Were strategies to deal with confounding factors stated? | Were the outcomes measured in a valid and reliable way? | Was appropriate statistical analysis used? |
| --- | --- | --- | --- | --- | --- | --- | --- | --- |
| 1 | A. G. B. Stewart | Low Risk | Low Risk | Low Risk | Low Risk | Low Risk | Low Risk | Low Risk |
| 2 | M. M. Bob-Manuel | Low Risk | Low Risk | Low Risk | Low Risk | Low Risk | Low Risk | Low Risk |
| 3 | S. R. Abotorabi | Low Risk | Some Risk | Low Risk | Some Risk | Some Risk | High Risk | Low Risk |
| 4 | Y. W. Gomi | Low Risk | Some Risk | Some Risk | Low Risk | Low Risk | Low Risk | High Risk |
| 5 | D. S. Miloshevski | Low Risk | Low Risk | Low Risk | Low Risk | Low Risk | Low Risk | Low Risk |
| 6 | N. S. G. Ngom | Low Risk | Low Risk | Low Risk | Low Risk | Low Risk | Low Risk | Low Risk |
| 7 | R. E. F. Mendes | Low Risk | Low Risk | Low Risk | High Risk | High Risk | Low Risk | Low Risk |
| 8 | D. G. Kekic | Low Risk | Low Risk | Low Risk | Low Risk | Low Risk | Low Risk | Low Risk |
| 9 | J. A. N. Karlowsky | Low Risk | Some Risk | Low Risk | Low Risk | Low Risk | Low Risk | High Risk |
| 10 | L. R. S. Duncan | Low Risk | Some Risk | Low Risk | Low Risk | Low Risk | Low Risk | High Risk |
| 11 | L. R. S. Duncan | Low Risk | Some Risk | Low Risk | Low Risk | Low Risk | Low Risk | High Risk |
| 12 | S. M. N. Mousavi | Low Risk | Low Risk | Low Risk | Low Risk | Low Risk | Low Risk | Low Risk |
| 13 | C. T. Matani | Some Risk | Some Risk | Low Risk | Low Risk | High Risk | High Risk | Low Risk |
| 14 | F. A. F. d. O. Santana | Low Risk | Low Risk | Low Risk | Low Risk | Low Risk | Low Risk | Low Risk |
| 15 | G. L.-C. de Figueiredo Sanches | Low Risk | Low Risk | Low Risk | Low Risk | Low Risk | Low Risk | High Risk |
| 16 | M. N. Laczeski | Low Risk | Low Risk | Low Risk | Low Risk | Low Risk | High Risk | High Risk |
| 17 | S. T. Belard | Low Risk | Low Risk | Low Risk | Low Risk | Low Risk | Low Risk | Low Risk |
| 18 | N. M. K. Nkembe | Low Risk | Low Risk | Low Risk | Low Risk | Low Risk | Some Risk | High Risk |
| 19 | B. A. S. M. Pimentel | Low Risk | Low Risk | Low Risk | Some Risk | Some Risk | Low Risk | Low Risk |
| 20 | V. G. A. Dutra | Low Risk | Some Risk | Some Risk | Some Risk | Low Risk | Low Risk | Low Risk |
| 21 | M. A. K. Shadbad | Low Risk | Low Risk | Low Risk | Low Risk | Low Risk | Low Risk | Low Risk |
| 22 | E. R. P.-R. Martins | Low Risk | Low Risk | Low Risk | Low Risk | Low Risk | Low Risk | Low Risk |
| 23 | E. J. B. Saad | High Risk | High Risk | High Risk | High Risk | High Risk | High Risk | High Risk |
| 24 | M. J.-K. Frej-Mądrzak | Some Risk | Some Risk | Some Risk | Some Risk | Some Risk | Low Risk | Low Risk |
| 25 | M. M. Majigo | Low Risk | Low Risk | Low Risk | Low Risk | Low Risk | Low Risk | Low Risk |
| 26 | E. S. Shipitsyna | Some Risk | Some Risk | Some Risk | Some Risk | Some Risk | Some Risk | Some Risk |
| 27 | B. J. C. Metcalf | Low Risk | Low Risk | Low Risk | Some Risk | Some Risk | Low Risk | Low Risk |
| 28 | K. L. C. Hon | Low Risk | Low Risk | Low Risk | Low Risk | Low Risk | Low Risk | Low Risk |
| 29 | A. E. A. A. H. El-Gendy | Low Risk | Low Risk | Low Risk | Low Risk | Low Risk | Low Risk | Low Risk |
| 30 | F. N. Z. Sulung | Low Risk | Low Risk | Low Risk | Low Risk | Low Risk | Low Risk | Low Risk |
| 31 | P. T. Wang | Low Risk | Low Risk | Low Risk | Low Risk | Low Risk | Low Risk | Low Risk |
| 32 | S. L. Wang | Low Risk | Low Risk | Low Risk | Low Risk | Low Risk | Low Risk | Low Risk |
| 33 | S. F. Teatero | Low Risk | Low Risk | Low Risk | Low Risk | Low Risk | Low Risk | Low Risk |
| 34 | P. M. Wang | Low Risk | Low Risk | Low Risk | Low Risk | Low Risk | Low Risk | Low Risk |
| 35 | H. C. M. Slotved | Low Risk | Some Risk | Low Risk | Some Risk | Some Risk | Low Risk | High Risk |
| 36 | Y. H. C. Wang | Low Risk | Low Risk | Low Risk | Low Risk | Low Risk | Low Risk | Low Risk |
| 37 | S. R.-F. Fröhlicher | Some Risk | Some Risk | Some Risk | Some Risk | Some Risk | Low Risk | Low Risk |
| 38 | T. O. Ikebe | Low Risk | Low Risk | Low Risk | Low Risk | Low Risk | Some Risk | Low Risk |
| 39 | E. H. S. Størdal | Low Risk | Low Risk | Low Risk | Low Risk | Low Risk | Some Risk | Low Risk |
| 40 | F. F. Alp | Low Risk | Low Risk | Low Risk | Low Risk | Low Risk | Low Risk | Low Risk |
| 41 | J. B. Ngonzi | Low Risk | Low Risk | Low Risk | Low Risk | Low Risk | Low Risk | Low Risk |
| 42 | H. G. H. Bae | Low Risk | Low Risk | Low Risk | Some Risk | Some Risk | Low Risk | Low Risk |
| 43 | M. K. Kitamura | Low Risk | Low Risk | Low Risk | Low Risk | Low Risk | Low Risk | Low Risk |
| 44 | K. A. Cooper | Some Risk | Some Risk | Some Risk | Some Risk | Some Risk | Some Risk | High Risk |
| 45 | T. V. Alemán | Low Risk | Low Risk | Low Risk | Low Risk | Low Risk | Low Risk | Low Risk |
| 46 | S. J. K. Choi | Low Risk | Low Risk | Low Risk | Low Risk | Low Risk | Low Risk | High Risk |
| 47 | T. B. Rasamiravaka | Some Risk | Some Risk | Some Risk | Some Risk | Some Risk | High Risk | Low Risk |
| 48 | D. G. Safari | Low Risk | Low Risk | Low Risk | Low Risk | Low Risk | Low Risk | Low Risk |
| 49 | T. F. Motallebirad | Low Risk | Low Risk | Low Risk | Low Risk | Low Risk | Low Risk | Low Risk |
| 50 | R. A.-Z. Dube | Low Risk | Low Risk | Low Risk | Low Risk | Low Risk | Low Risk | Low Risk |
| 51 | S. C. O. Jisuvei | Low Risk | Low Risk | Low Risk | Low Risk | Low Risk | Low Risk | Low Risk |
| 52 | O. K. A. Husen | Low Risk | Low Risk | Low Risk | Low Risk | Low Risk | Low Risk | Low Risk |
| 53 | A. M. Bitew | Low Risk | Low Risk | Low Risk | Low Risk | Low Risk | Low Risk | Low Risk |
| 54 | M. S. P. Abdallah | Low Risk | Low Risk | Low Risk | Low Risk | Low Risk | Low Risk | Low Risk |
| 55 | M. A. Misic | Low Risk | Low Risk | Low Risk | Low Risk | Low Risk | Low Risk | Low Risk |
| 56 | S. M. Sahraee | Low Risk | Low Risk | Low Risk | Low Risk | Low Risk | Low Risk | Low Risk |
| 57 | Z. L. T. Woldu | Some Risk | Some Risk | Low Risk | Low Risk | Low Risk | High Risk | High Risk |
| 58 | G. C. A. Soares | Low Risk | Low Risk | Low Risk | Low Risk | Low Risk | Low Risk | Low Risk |
| 59 | Y. P. Ge | Low Risk | Low Risk | Low Risk | Low Risk | Low Risk | Low Risk | Low Risk |
| 60 | O. O. A. Ojo | Low Risk | Low Risk | Low Risk | Low Risk | Low Risk | Low Risk | Low Risk |
| 61 | E. A. K. Al Romaihi | Low Risk | Low Risk | Low Risk | Low Risk | Low Risk | Low Risk | High Risk |
| 62 | N. A. J. Al-Tulaibawi | Low Risk | Low Risk | Low Risk | Low Risk | Low Risk | Low Risk | Low Risk |
| 63 | M. A. Qadi | Low Risk | Low Risk | Low Risk | Low Risk | Low Risk | Low Risk | Low Risk |
| 64 | E. L. M. Haimbodi | Low Risk | Low Risk | Low Risk | Low Risk | Low Risk | Low Risk | High Risk |
| 65 | K. B. B. O. Luiz FB Alves, R. R. | Low Risk | Low Risk | Low Risk | Low Risk | Low Risk | Low Risk | Low Risk |
| 66 | C. E. Njoku, C. Agbakwuru, A. | Low Risk | Low Risk | Low Risk | Low Risk | Low Risk | High Risk | Low Risk |
| 67 | H. S. Jiang, M. Kui | Low Risk | Low Risk | Low Risk | Low Risk | Low Risk | Low Risk | Low Risk |
| 68 | F. S. Numanović | Low Risk | Low Risk | Low Risk | Low Risk | Low Risk | Low Risk | Low Risk |
| 69 | N. K. Nagano | Low Risk | Low Risk | Low Risk | Low Risk | Low Risk | Low Risk | High Risk |
| 70 | S. M. Teatero | Low Risk | Low Risk | Low Risk | Low Risk | Low Risk | Low Risk | High Risk |
| 71 | D. G. R. Jamrozy | Some Risk | Some Risk | Some Risk | Some Risk | Some Risk | Some Risk | Some Risk |
| 72 | F. N. Khodaei | Low Risk | Low Risk | Low Risk | Low Risk | Low Risk | Low Risk | Low Risk |
| 73 | X. C. Wang | Low Risk | Low Risk | Low Risk | Low Risk | Low Risk | Low Risk | Low Risk |
| 74 | M. B. Novosak | Low Risk | Low Risk | Low Risk | Low Risk | Low Risk | Low Risk | Low Risk |
| 75 | B. S. Wu | Low Risk | Low Risk | Low Risk | Low Risk | Low Risk | Low Risk | Low Risk |
| 76 | M. H. Malek-Jafarian | Low Risk | Low Risk | Low Risk | Low Risk | Low Risk | Some Risk | High Risk |
| 77 | Y. Y. K. Li | Low Risk | Low Risk | Low Risk | Low Risk | Low Risk | High Risk | High Risk |
| 78 | J. F. Liu, Z. Yu | Low Risk | Low Risk | Low Risk | Low Risk | Low Risk | Low Risk | Low Risk |
| 79 | D. L. R. Horn | Low Risk | Low Risk | Low Risk | Low Risk | Some Risk | Some Risk | Low Risk |
| 80 | X. O. G. Li | Low Risk | Low Risk | Low Risk | Low Risk | Low Risk | Some Risk | Some Risk |
| 81 | G. W. Li | Low Risk | Low Risk | Low Risk | Some Risk | Low Risk | Low Risk | Low Risk |
| 82 | A. F. Ali Hamad | Low Risk | Low Risk | Low Risk | Low Risk | Low Risk | Low Risk | Low Risk |
| 83 | J. G. Yayan | Low Risk | Low Risk | Low Risk | Low Risk | Low Risk | Low Risk | Low Risk |
| 84 | M. T. Gizachew | Low Risk | Low Risk | Low Risk | Low Risk | Low Risk | Low Risk | Low Risk |
| 85 | L. D. Mudzikati | Low Risk | Low Risk | Low Risk | Low Risk | Low Risk | Low Risk | Low Risk |
| 86 | K. B. Venkatnarayan | High Risk | High Risk | Some Risk | Some Risk | High Risk | High Risk | High Risk |
| 87 | M. M. Dehdashtian | Low Risk | Low Risk | Low Risk | Low Risk | Low Risk | Low Risk | Low Risk |
| 88 | R. I. Creti | Low Risk | High Risk | High Risk | High Risk | High Risk | High Risk | Low Risk |
| 89 | D. C. Guo | Low Risk | Low Risk | Low Risk | Low Risk | Low Risk | Low Risk | Low Risk |
| 90 | M. J. Ghamari | Low Risk | Low Risk | Low Risk | Low Risk | Low Risk | Low Risk | High Risk |
| 91 | M. A. Alzayer | Low Risk | Low Risk | Low Risk | Low Risk | Low Risk | Low Risk | Low Risk |
| 92 | N. B. Gharabeigi | Low Risk | Low Risk | Low Risk | Low Risk | Low Risk | Low Risk | Low Risk |
| 93 | S. P. Dehbashi | Low Risk | Low Risk | Low Risk | Low Risk | Low Risk | Some Risk | Low Risk |
| 94 | R. B. B. A. Schuab | Low Risk | Low Risk | Low Risk | Low Risk | Low Risk | Some Risk | Low Risk |
| 95 | I. P. Gajic | Low Risk | Low Risk | Low Risk | Low Risk | Low Risk | Low Risk | Low Risk |
| 96 | A. L. Bergal | Low Risk | Low Risk | Low Risk | Low Risk | Low Risk | Low Risk | Low Risk |
| 97 | K. S. Shrestha | Low Risk | Low Risk | Low Risk | Low Risk | Low Risk | Low Risk | Low Risk |
| 98 | M. T. Gizachew | Low Risk | Low Risk | Low Risk | Low Risk | Low Risk | Low Risk | Low Risk |
| 99 | H. C. Jiang | Low Risk | Low Risk | Low Risk | Low Risk | Low Risk | Low Risk | Low Risk |
| 100 | S. V. Simoni | Low Risk | Low Risk | Low Risk | Low Risk | Low Risk | Low Risk | Low Risk |
| 101 | B. L.-M. Moltó-García | Low Risk | Low Risk | Low Risk | Low Risk | Low Risk | Low Risk | Low Risk |
| 102 | M. U. Kawaguchiya | Low Risk | Some Risk | Some Risk | Some Risk | Low Risk | Low Risk | Low Risk |
| 103 | J. J. Li, W. Gao | Low Risk | Low Risk | Low Risk | Low Risk | Low Risk | Low Risk | Low Risk |
| 104 | M. H. H. Tsai | Low Risk | Low Risk | Low Risk | Low Risk | Low Risk | Low Risk | Low Risk |
| 105 | B. C. Lu | Low Risk | Some Risk | Some Risk | Low Risk | Low Risk | Low Risk | Some Risk |
| 106 | Z. J. Liu | Low Risk | Low Risk | Low Risk | Low Risk | Low Risk | Low Risk | Some Risk |
| 107 | A. O. Dobrut | Low Risk | Low Risk | Low Risk | Low Risk | Low Risk | Low Risk | Low Risk |
| 108 | K. A. B. Al Benwan | Low Risk | Low Risk | Low Risk | Low Risk | Low Risk | Low Risk | Low Risk |
| 109 | G. L. P | Low Risk | Some Risk | Some Risk | Some Risk | Some Risk | Some Risk | Low Risk |
| 110 | Y. Y. Qiu, J | Low Risk | Low Risk | Low Risk | Low Risk | Low Risk | Low Risk | Low Risk |
| 111 | M. A. Ali | Low Risk | Low Risk | Low Risk | Low Risk | Low Risk | Low Risk | Low Risk |
| 112 | B. W. Lu | Low Risk | Low Risk | Low Risk | Low Risk | Low Risk | Low Risk | Low Risk |
| 113 | W. T.-U. Hiriote | Low Risk | Low Risk | Low Risk | Low Risk | Low Risk | Low Risk | Low Risk |
| 114 | P. F. Liu | Low Risk | Low Risk | Low Risk | Low Risk | Low Risk | Low Risk | Low Risk |
| 115 | L. M. Madrid | Low Risk | Low Risk | Low Risk | Low Risk | Low Risk | Low Risk | Low Risk |
| 116 | S. M. Rostami | Low Risk | Low Risk | Low Risk | Low Risk | Low Risk | Low Risk | Low Risk |
| 117 | S. A. Shabayek | Low Risk | Low Risk | Low Risk | Low Risk | Low Risk | Low Risk | Low Risk |
| 118 | H. K. Houri | Low Risk | Low Risk | Low Risk | Low Risk | Low Risk | Low Risk | Low Risk |
| 119 | H. K. Moroi | Low Risk | Low Risk | Some Risk | Some Risk | Low Risk | Low Risk | Low Risk |
| 120 | A. M. Shafi | Some Risk | Some Risk | Some Risk | Some Risk | Some Risk | High Risk | High Risk |
| 121 | Y. Z. H. Yan | Some Risk | Some Risk | Some Risk | Some Risk | Some Risk | Some Risk | High Risk |
| 122 | A. M. J. Ebrahem | Low Risk | Low Risk | Low Risk | Low Risk | Some Risk | Some Risk | High Risk |
| 123 | L. A. K. Al Abbas | Low Risk | Low Risk | Low Risk | Low Risk | Low Risk | Low Risk | High Risk |
| 124 | X. H. Vuillemin | Low Risk | Low Risk | Low Risk | Low Risk | Low Risk | Some Risk | Low Risk |
| 125 | E. H. Graux | Low Risk | Low Risk | Low Risk | Low Risk | Low Risk | Low Risk | Low Risk |
| 126 | J. Z. Zhang | Low Risk | Low Risk | Low Risk | Low Risk | Low Risk | Low Risk | Low Risk |
| 127 | B. K. Sigaúque | Some Risk | Some Risk |  |  |  | High Risk | Low Risk |
| 128 | I. A.-A. Al-Subol | Low Risk | Low Risk | Low Risk | Low Risk | Low Risk | Low Risk | Low Risk |
| 129 | S. N. A. Alani | Low Risk | Low Risk | Low Risk | Some Risk | Some Risk | Some Risk | Low Risk |
| 130 | D. R. Kaminska | Low Risk | Low Risk | Low Risk | Low Risk | Low Risk | Low Risk | Low Risk |
| 131 | E. F. Lopes, T. Machado, M. P. | Low Risk | Low Risk | Low Risk | Low Risk | Low Risk | Low Risk | Low Risk |
| 132 | K. K. F. Gao | Low Risk | Low Risk | Low Risk | Low Risk | Low Risk | Low Risk | Low Risk |
| 133 | R. R. A. El-Lakany | Low Risk | Low Risk | Low Risk | Low Risk | Low Risk | Low Risk | Low Risk |
| 134 | M. I. B. Renteria | Low Risk | Low Risk | Low Risk | Low Risk | Low Risk | Low Risk | Low Risk |
| 135 | D. J. A. Biedenbach | Low Risk | Low Risk | Low Risk | Low Risk | Low Risk | Low Risk | High Risk |
| 136 | J. A. W. Karlowsky | Low Risk | Low Risk | Low Risk | Low Risk | Low Risk | Low Risk | High Risk |
| 137 | J. A. W. Karlowsky | Low Risk | Low Risk | Low Risk | Low Risk | Low Risk | Low Risk | High Risk |
| 138 | D. S. Piérard, G. G. | Low Risk | Low Risk | Low Risk | Low Risk | Low Risk | Low Risk | High Risk |
| 139 | J. A. B. Karlowsky | Low Risk | Low Risk | Low Risk | Low Risk | Low Risk | Low Risk | High Risk |
| 140 | N. V. Mohamed | Low Risk | Low Risk | Low Risk | Low Risk | Low Risk | Low Risk | High Risk |
| 141 | P. R. Hsueh | Low Risk | Some Risk | Low Risk | Low Risk | Low Risk | Low Risk | Low Risk |
| 142 | W. T. L. Lee, M. C. | Low Risk | Some Risk | Low Risk | Low Risk | Low Risk | Low Risk | Low Risk |
| 143 | S. T. Kardos | Low Risk | Some Risk | Low Risk | Low Risk | Low Risk | Low Risk | Low Risk |
| 144 | M. M. Emaneini | Low Risk | Some Risk | Low Risk | Low Risk | Low Risk | Low Risk | High Risk |
| 145 | W. Y. Girma | Low Risk | Low Risk | Low Risk | Low Risk | Low Risk | Low Risk | Low Risk |
| 146 | K. E. S. Proudmore | Low Risk | Some Risk | Low Risk | Low Risk | Low Risk | Low Risk | High Risk |
| 147 | A. M. K. Mohamed | Low Risk | Some Risk | Low Risk | Low Risk | Low Risk | Low Risk | High Risk |
| 148 | R. O. A. Biobaku Oluwafunmilola | Low Risk | Low Risk | Low Risk | Low Risk | Low Risk | Low Risk | Low Risk |
| 149 | M. D. Gogoi | Low Risk | Low Risk | Some Risk | Some Risk | Low Risk | Low Risk | Low Risk |
| 150 | G. N. K. Dilrukshi | Low Risk | Some Risk | Low Risk | Some Risk | Some Risk | Low Risk | Low Risk |
| 151 | Y. J. Dong | Low Risk | Low Risk | Some Risk | Low Risk | Some Risk | Some Risk | Low Risk |
| 152 | B. C. A. Iweriebor | Low Risk | Low Risk | Low Risk | Low Risk | Low Risk | Low Risk | High Risk |
| 153 | Y. G. Leykun | Low Risk | Low Risk | Some Risk | Low Risk | Some Risk | Some Risk | Low Risk |
| 154 | A. G. Kumalo | Low Risk | Low Risk | Low Risk | Low Risk | Low Risk | Low Risk | Low Risk |
| 155 | E. S. M. Björnsdóttir | Low Risk | Some Risk | Low Risk | Low Risk | Low Risk | Low Risk | Low Risk |
| 156 | R. D. Baldan | Low Risk | Some Risk | Low Risk | Low Risk | Low Risk | Low Risk | Low Risk |
| 157 | L. M. J. Warrier | Low Risk | Some Risk | Some Risk | Some Risk | Some Risk | Low Risk | Low Risk |
| 158 | S. S. Wataradee | Low Risk | Low Risk | Low Risk | Low Risk | Low Risk | Low Risk | Low Risk |
| 159 | H. M. L. Kang | Low Risk | Low Risk | Low Risk | Low Risk | Low Risk | Low Risk | Low Risk |
| 160 | M. M. Doumith | Low Risk | Some Risk | Some Risk | Some Risk | Some Risk | Some Risk | High Risk |
| 161 | A. N. C. Williams | Low Risk | Low Risk | Low Risk | Low Risk | Low Risk | Low Risk | High Risk |
| 162 | T. G. Perme | Low Risk | Some Risk | Low Risk | Low Risk | Low Risk | Low Risk | Low Risk |
| 163 | U. B. P. Khan | Low Risk | Low Risk | Low Risk | Low Risk | Low Risk | Low Risk | High Risk |
| 164 | E. R. Campisi, R. Ji | Some Risk | Some Risk | Some Risk | Some Risk | Some Risk | Some Risk | High Risk |
| 165 | G. D. C. R.-M. Palacios-Saucedo | Low Risk | Low Risk | Low Risk | Low Risk | Low Risk | Low Risk | Low Risk |
| 166 | Y. W. Zhou | Low Risk | Low Risk | Low Risk | Low Risk | Low Risk | Low Risk | Low Risk |
| 167 | G. I. Gherardi | Low Risk | Some Risk | Some Risk | Some Risk | Some Risk | Low Risk | High Risk |
| 168 | O. H. M. A. Feuerschuette. | Low Risk | Low Risk | Low Risk | Low Risk | Low Risk | Low Risk | Low Risk |
| 169 | H. R. Saffar | Low Risk | Low Risk | Low Risk | Low Risk | Low Risk | Low Risk | Low Risk |
| 170 | S. G. Hadavand | Low Risk | Low Risk | Low Risk | Low Risk | Low Risk | Low Risk | Low Risk |
| 171 | C. G. S. Carvalhaes | Low Risk | Low Risk | Low Risk | Low Risk | Low Risk | Low Risk | Low Risk |
| 172 | M. M. W. Ali | Low Risk | Low Risk | Low Risk | Low Risk | Low Risk | Low Risk | Low Risk |
| 173 | M. T. Ábrók | Low Risk | Low Risk | Low Risk | Low Risk | Low Risk | Low Risk | High Risk |
| 174 | T. C. Ikebe | Low Risk | Low Risk | Low Risk | Low Risk | Low Risk | Low Risk | High Risk |
| 175 | H. Z. Shen | Low Risk | Low Risk | Low Risk | Low Risk | Low Risk | Low Risk | High Risk |
| 176 | S. N. Jones | Low Risk | Low Risk | Low Risk | Low Risk | Low Risk | Low Risk | Low Risk |
| 177 | X. S. M. Guan | Low Risk | Low Risk | Low Risk | Low Risk | Low Risk | Low Risk | Low Risk |
| 178 | A. H. Alhhazmi | Low Risk | Low Risk | Low Risk | Low Risk | Low Risk | Low Risk | Low Risk |
| 179 | X. L. Zhang | Low Risk | Low Risk | Low Risk | Low Risk | Low Risk | Low Risk | Low Risk |
| 180 | C. D. Florindo | Low Risk | Low Risk | Low Risk | Low Risk | Low Risk | Low Risk | Low Risk |
| 181 | A. T. Ma, L. A. Corsiatto | Low Risk | Low Risk | Low Risk | Low Risk | Low Risk | Low Risk | Low Risk |
| 182 | M. D. P. C.-R. Crespo-Ortiz | Low Risk | Low Risk | Low Risk | Low Risk | Low Risk | Low Risk | Low Risk |
| 183 | Y. T. Kao | Low Risk | Low Risk | Low Risk | Low Risk | Low Risk | Low Risk | Low Risk |
| 184 | K. C. Hayes | Low Risk | Low Risk | Low Risk | Low Risk | Low Risk | Low Risk | Low Risk |
| 185 | S. A. Teatero | Low Risk | Low Risk | Low Risk | Low Risk | Low Risk | Low Risk | Low Risk |
| 186 | O. P. Tulyaprawat | Low Risk | Low Risk | Low Risk | Low Risk | Low Risk | Low Risk | Low Risk |
| 187 | L. K. Zhang | Low Risk | Low Risk | Low Risk | Low Risk | Low Risk | Low Risk | Low Risk |
| 188 | Y. D. Guo | Low Risk | Low Risk | Low Risk | Low Risk | Low Risk | Low Risk | High Risk |
| 189 | M. K. Nabavinia | Low Risk | Low Risk | Low Risk | Low Risk | Low Risk | Low Risk | Low Risk |
| 190 | X. M. D. Li | Low Risk | Low Risk | Low Risk | Low Risk | Low Risk | Low Risk | Low Risk |
| 191 | N. A. E. R. Fahim | Low Risk | Low Risk | Low Risk | Low Risk | Low Risk | Low Risk | Low Risk |
| 192 | A. A. E. M. El Shahaway | Low Risk | Low Risk | Low Risk | Low Risk | Low Risk | Low Risk | Low Risk |
| 193 | S. H. Jalalifar | Low Risk | Low Risk | Low Risk | Low Risk | Low Risk | Low Risk | Low Risk |
| 194 | S. K. Panahi | Low Risk | Low Risk | Low Risk | Low Risk | Low Risk | Low Risk | Low Risk |
| 195 | T. F. Motallebirad | Low Risk | Low Risk | Low Risk | Low Risk | Low Risk | Low Risk | Low Risk |
| 196 | L. R. S. Burcham | Low Risk | Low Risk | Low Risk | Low Risk | Low Risk | Some Risk | Low Risk |
| 197 | K. E. Evangelia | Low Risk | Low Risk | Low Risk | Low Risk | Low Risk | Low Risk | High Risk |
| 198 | M. A. M. Malita | Low Risk | Low Risk | Low Risk | Low Risk | Low Risk | Low Risk | High Risk |
| 199 | G. G. Goudarzi | Low Risk | Low Risk | Low Risk | Low Risk | Low Risk | Low Risk | Low Risk |
| 200 | S. K. Asghar | Low Risk | Some Risk | Low Risk | Low Risk | Low Risk | Low Risk | Low Risk |
| 201 | E. M. A. J. Felemban | Low Risk | Low Risk | Low Risk | Low Risk | Low Risk | Low Risk | High Risk |
| 202 | A. K. D. Brigtsen | Low Risk | Low Risk | Low Risk | Low Risk | Low Risk | Low Risk | Low Risk |
| 203 | W. Y. Mulu | Low Risk | Low Risk | Low Risk | Low Risk | Low Risk | Low Risk | Low Risk |
| 204 | W. Z. Ji | Low Risk | Low Risk | Low Risk | Low Risk | Low Risk | Low Risk | Low Risk |
| 205 | P. E. H. Akpaka | Low Risk | Low Risk | Low Risk | Low Risk | Low Risk | Low Risk | Low Risk |
| 206 | D. N. L. Sapugahawatte | Low Risk | Low Risk | Low Risk | Low Risk | Low Risk | Low Risk | Low Risk |
| 207 | J. F. C. Hsu | Low Risk | Low Risk | Low Risk | Low Risk | Low Risk | Low Risk | Low Risk |
| 208 | I. A. J. Yoon | Low Risk | Low Risk | Low Risk | Low Risk | Low Risk | Low Risk | Low Risk |
| 209 | J. W. Jiao | Low Risk | Low Risk | Low Risk | Low Risk | Low Risk | Low Risk | Low Risk |
| 210 | S. T. S. D. AlZuheiri | Low Risk | Low Risk | Low Risk | Low Risk | Low Risk | Low Risk | Low Risk |
| 211 | X. D. Li | Low Risk | Low Risk | Low Risk | Low Risk | Low Risk | Low Risk | Low Risk |
| 212 | N. K. Hirai | Low Risk | Low Risk | Low Risk | Low Risk | Low Risk | Low Risk | High Risk |
| 213 | C. F. Li | Some Risk | Some Risk | Low Risk | Low Risk | Low Risk | Some Risk | Low Risk |
| 214 | L. Y. Z. Guo | Low Risk | Low Risk | Low Risk | Low Risk | Low Risk | Low Risk | Low Risk |
| 215 | H. K. Lee | Low Risk | Low Risk | Low Risk | Low Risk | Low Risk | Low Risk | Low Risk |
| 216 | P. B. Mathur | Some Risk | Low Risk | Low Risk | Low Risk | Low Risk | Low Risk | High Risk |
| 217 | S. P. Kernéis, C. Barnier | Low Risk | Low Risk | Low Risk | Low Risk | Low Risk | Low Risk | Low Risk |
| 218 | Y. H. C. Wang | Low Risk | Low Risk | Low Risk | Low Risk | Low Risk | Low Risk | Low Risk |
| 219 | J. F. L. Hsu | Low Risk | Low Risk | Low Risk | Low Risk | Low Risk | Low Risk | Low Risk |
| 220 | S. J. T. Zeng | Low Risk | High Risk | Low Risk | Low Risk | Low Risk | Some Risk | Low Risk |
| 221 | M. J. Emaneini | Low Risk | Low Risk | Low Risk | Low Risk | Low Risk | Low Risk | Low Risk |
| 222 | Y. Y. Tang | Low Risk | Some Risk | Some Risk | Some Risk | Some Risk | Some Risk | Low Risk |
| 223 | M. E. S. D. Suhaimi | Low Risk | Some Risk | Low Risk | Low Risk | Low Risk | Low Risk | Low Risk |
| 224 | G. B. Piccinelli | Low Risk | Low Risk | Low Risk | Low Risk | Low Risk | Low Risk | Low Risk |
| 225 | A. H. Al-Matary | Some Risk | Low Risk | Low Risk | Low Risk | Low Risk | Some Risk | Low Risk |
| 226 | B. W. Chang, A. Hosoya | Low Risk | Low Risk | Low Risk | Low Risk | Low Risk | Low Risk | Low Risk |
| 227 | C. L. Hays | Low Risk | Low Risk | Low Risk | Low Risk | Low Risk | Low Risk | Low Risk |
| 228 | R. K. S. Flamm | Low Risk | Low Risk | Low Risk | Low Risk | Low Risk | Low Risk | Low Risk |
| 229 | M. R. Navidini | Low Risk | Low Risk | Low Risk | Low Risk | Low Risk | Low Risk | Low Risk |
| 230 | O. E. Swann | Low Risk | Low Risk | Low Risk | Low Risk | Low Risk | Low Risk | Low Risk |
| 231 | Y. Z. Zhou | Low Risk | Low Risk | Low Risk | Low Risk | Low Risk | Low Risk | Low Risk |
| 232 | M. M. Majigo | Low Risk | Low Risk | Low Risk | Low Risk | Low Risk | Low Risk | Low Risk |
| 233 | S. Ahmad | Low Risk | Low Risk | Low Risk | Low Risk | Low Risk | Low Risk | High Risk |
| 234 | M. K. M. Mwei | Some Risk | Some Risk | Low Risk | Low Risk | Low Risk | Some Risk | Low Risk |
| 235 | E. K. Foster-Nyarko | Some Risk | Some Risk | Low Risk | Low Risk | Low Risk | Some Risk | Low Risk |
| 236 | M. W. Morozumi | Some Risk | Some Risk | Low Risk | Low Risk | Low Risk | Some Risk | Low Risk |
| 237 | L. A. Vigliarolo | Some Risk | Some Risk | Low Risk | Low Risk | Low Risk | Some Risk | Low Risk |
| 238 | R. N. Morfin-Otero | Some Risk | Some Risk | Low Risk | Low Risk | Low Risk | Some Risk | Low Risk |
| 239 | N. I. Eskandarian | Some Risk | Some Risk | Low Risk | Low Risk | Low Risk | Some Risk | Low Risk |
| 240 | F. A. Lagunas-Rangel | Some Risk | Some Risk | Low Risk | Low Risk | Low Risk | Some Risk | Low Risk |
| 241 | P. S. Mubanga | Low Risk | Low Risk | Low Risk | Low Risk | Low Risk | Low Risk | Low Risk |
| 242 | P. M. Bhola | Low Risk | Low Risk | Low Risk | Low Risk | Low Risk | Low Risk | Low Risk |
| 243 | S. C. S. Melo | Some Risk | Low Risk | Low Risk | Low Risk | Low Risk | Low Risk | High Risk |
| 244 | B. M. Balkhi | Low Risk | Low Risk | Low Risk | Low Risk | Low Risk | Low Risk | Low Risk |
| 245 | M. D. Said | Low Risk | Low Risk | Low Risk | Low Risk | Low Risk | Low Risk | Low Risk |
| 246 | A. A. Mohamed | Low Risk | Low Risk | Low Risk | Low Risk | Low Risk | Low Risk | Low Risk |
| 247 | A. M. Tesfaye | Low Risk | High Risk | Low Risk | Low Risk | Low Risk | Some Risk | Low Risk |
| 248 | C. D. C. Minotti | Low Risk | High Risk | Low Risk | Low Risk | Low Risk | Some Risk | Low Risk |
| 249 | V. D. Van Du | Low Risk | Low Risk | Low Risk | Low Risk | Low Risk | Low Risk | Low Risk |
| 250 | H. F. Guo | Low Risk | Low Risk | Low Risk | Low Risk | Low Risk | Low Risk | Low Risk |
| 251 | M. Z. Dashtizade | Low Risk | Low Risk | Low Risk | Low Risk | Low Risk | Low Risk | Low Risk |
| 252 | N. K. Dilrukshi | Low Risk | Low Risk | Low Risk | Low Risk | Low Risk | Low Risk | Low Risk |
| 253 | J. Y. M. Bolukaoto | Low Risk | Low Risk | Low Risk | Low Risk | Low Risk | Low Risk | Low Risk |
| 254 | J. W. Tan | Low Risk | Low Risk | Low Risk | Low Risk | Low Risk | Low Risk | Low Risk |
| 255 | M. K. Zakerifar | Low Risk | Low Risk | Low Risk | Low Risk | Low Risk | Low Risk | High Risk |
| 256 | Z. Q. Cheng | Some Risk | Some Risk | Low Risk | Low Risk | Low Risk | Some Risk | Low Risk |
| 257 | R. M. Mudzana | Some Risk | Some Risk | Low Risk | Low Risk | Low Risk | Some Risk | Low Risk |
| 258 | Y. Y. H. Yu | Some Risk | Some Risk | Low Risk | Low Risk | Low Risk | Some Risk | Low Risk |
| 259 | X. T. Zhang | Some Risk | Some Risk | Low Risk | Low Risk | Low Risk | Some Risk | Low Risk |
| 260 | Y. H. Fujiya | Some Risk | Some Risk | Low Risk | Low Risk | Low Risk | Some Risk | Low Risk |
| 261 | M. C. B. J. Perim | Some Risk | Some Risk | Low Risk | Low Risk | Low Risk | Some Risk | Low Risk |
| 262 | G. F. A.-S. Karim | Some Risk | Some Risk | Low Risk | Low Risk | Low Risk | Some Risk | Low Risk |
| 263 | R. E. F. Mendes | Low Risk | Low Risk | Low Risk | Low Risk | Low Risk | Low Risk | Low Risk |
| 264 | M. I. Mukesi | Low Risk | Low Risk | Low Risk | Low Risk | Low Risk | Low Risk | Low Risk |
| 265 | M. A. F. Pfaller | Low Risk | Low Risk | Low Risk | Low Risk | Low Risk | Low Risk | Low Risk |
| 266 | M. H. J. C. Ko | Some Risk | Low Risk | Low Risk | Low Risk | Low Risk | Low Risk | High Risk |

**Supplementary Table 4: Antibiotic Resistance Across Countries: Highest and Lowest Resistance Values**

| Antibiotic | Country with Highest Resistance | Resistance Value (%) | Country with Lowest Resistance | Resistance Value (%) |
| --- | --- | --- | --- | --- |
| Clindamycin | Nigeria | 76.2 | Iceland | 1.0 |
| Erythromycin | Unknown | 88.9 | South Africa | 1.4 |
| Vancomycin | Switzerland | 95.5 | Syria | 0.0 |
| Ceftriaxone | United States | 95.0 | India | 0.1 |
| Azithromycin | Brazil | 86.0 | Iran | 9.1 |
| Erythrocin | Americas | 86.0 | Africa | 12.3 |
| Clarithromycin | Syria | 70.0 | Ethiopia | 12.5 |
| Tetracycline | Nigeria | 98.5 | Pakistan | 8.3 |
| Doxycycline | Asia | 82.1 | NA | 58.3 |
| TMP/SMX | Iran | 93.8 | Switzerland | 0.0 |
| Ciprofloxacin | Taiwan | 44.3 | Germany | 1.5 |
| Levofloxacin | Vietnam: (0.233, 0.342) | 28.5 | Slovenia | 0.3 |
| Gentamicin | Serbia | 72.7 | Iceland | 0.5 |
| nitrofurantoin | Trinidad & Tobago | 45.8 | Bahrain | 0.4 |
| cefepime | Syria | 70.0 | Taiwan | 44.6 |
| moxifloxacin | Brazil | 44.6 | Taiwan | 0.4 |
| Oxacillin | NA | 96.5 | Japan | 0.5 |
| Teicoplanin | NA | 2.1 | NA | 0.7 |
| Q/D | NA | 6.5 | NA | 0.3 |
| Chloramphenicol | Vietnam | 52.4 | Portugal | 0.5 |
| Cefditoren | Asia | 66.1 | Asia | 0.2 |
| Norfloxacin | Iran | 54.5 | Taiwan | 0.5 |
| AMC | Other | 97.6 | NA | 0.4 |
| Cefoxitine | NA | 89.5 | Asia | 0.5 |
| Norfloxacin | Brazil | 97.5 | Taiwan | 0.5 |
| Ofloxacin | NA | 85.1 | Taiwan | 0.5 |
| Amikacin | Europe | 99 | Europe | 0 |
| Nalidixic acid | Iran | 92.5 | Iran | 42 |


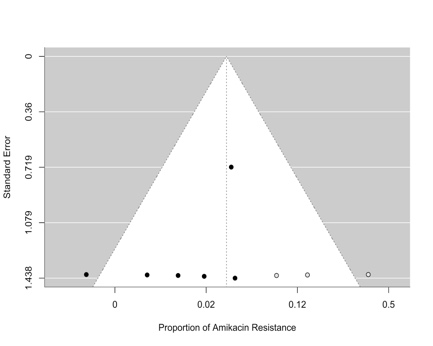

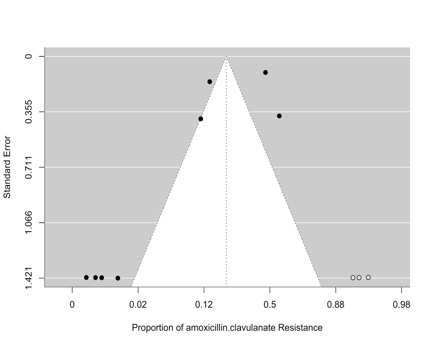

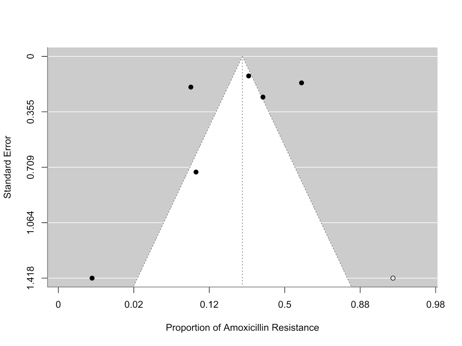

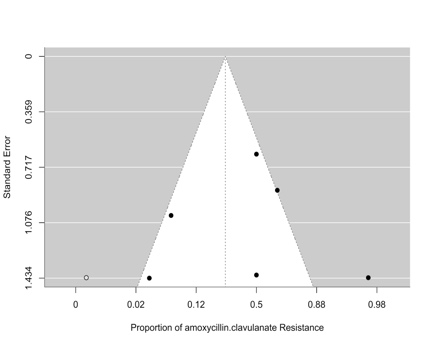

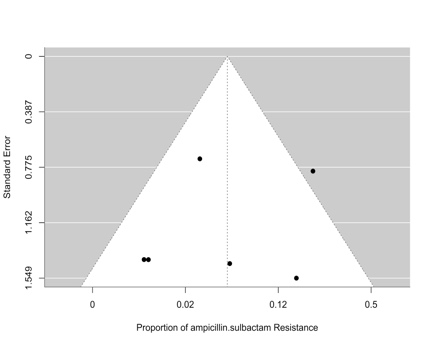

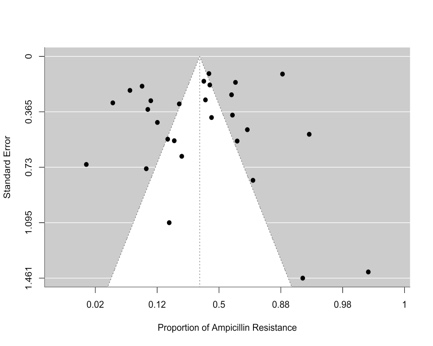

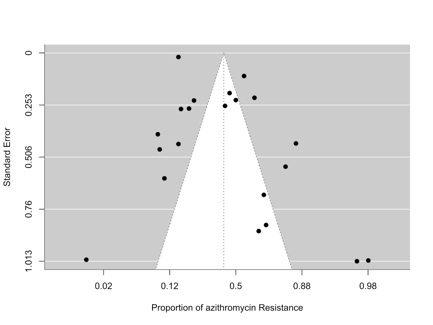

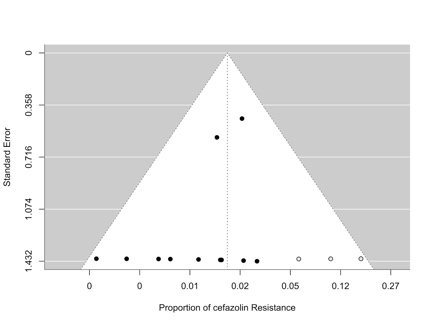

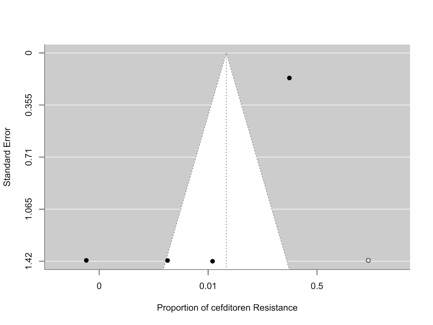

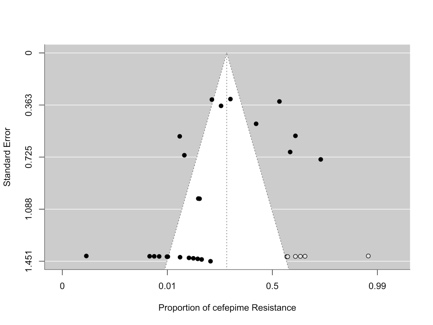

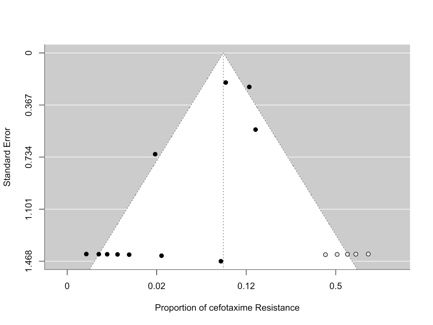

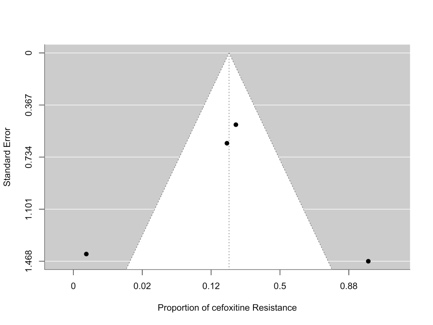

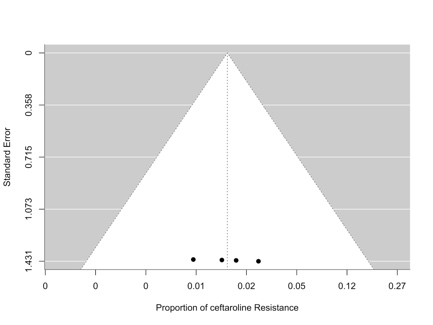

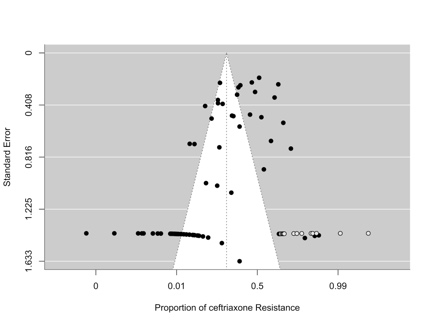

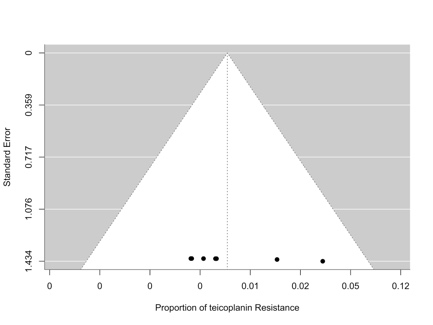

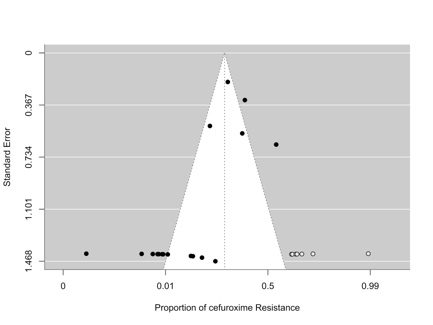

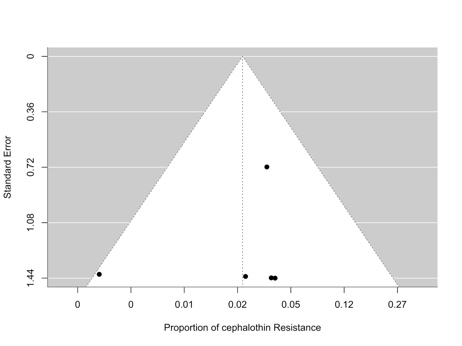

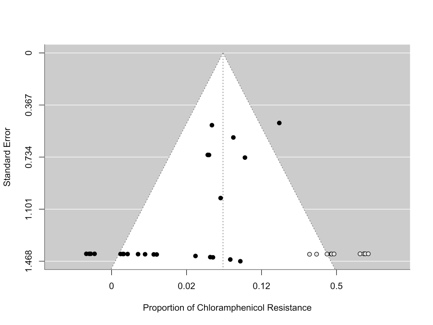

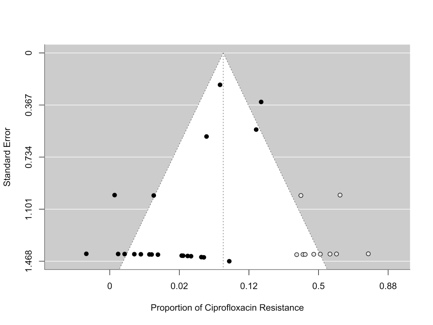

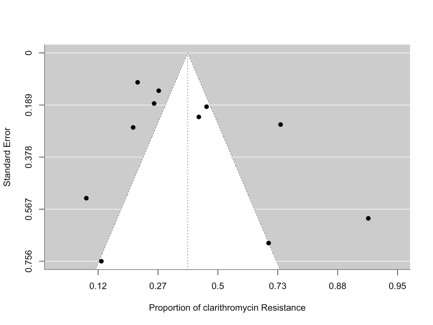

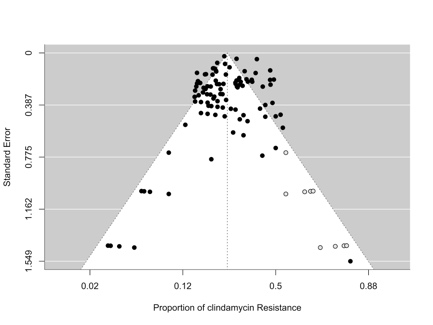

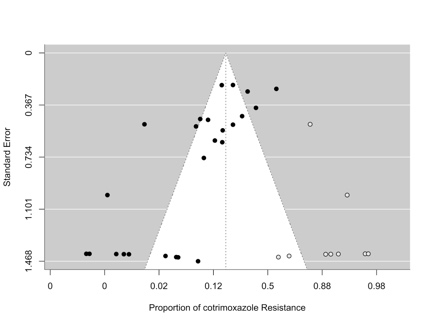

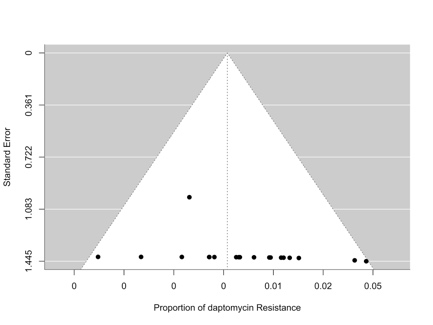

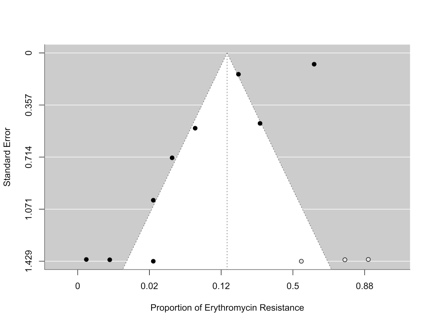


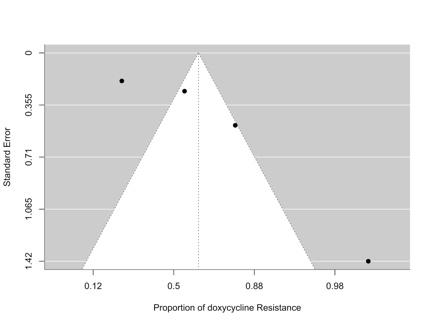

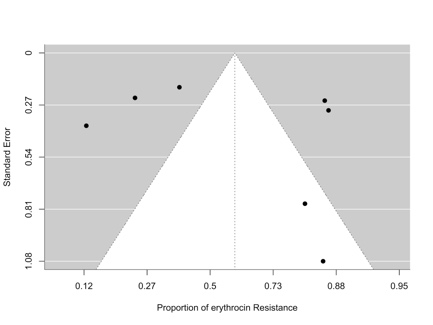

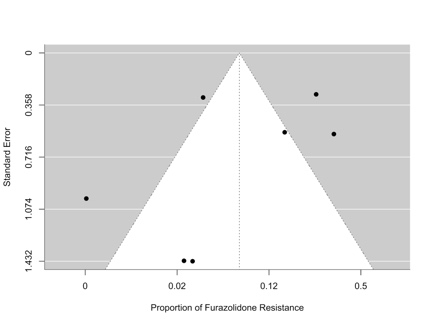

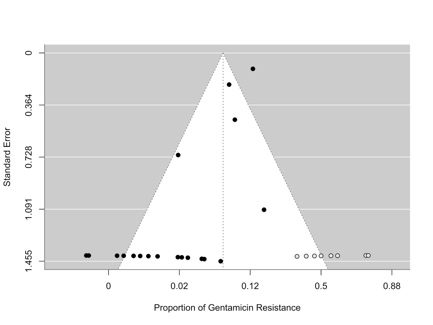


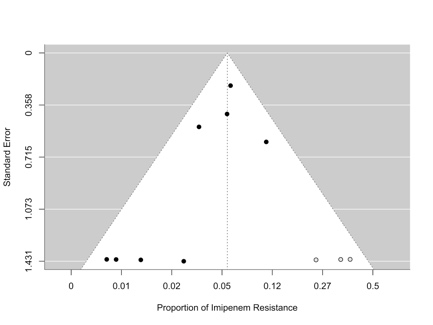

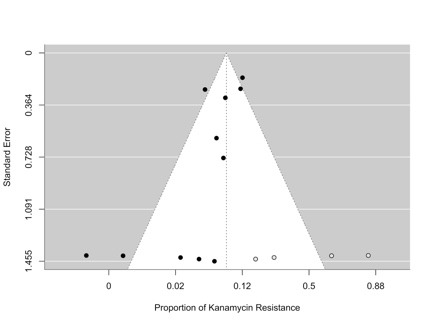

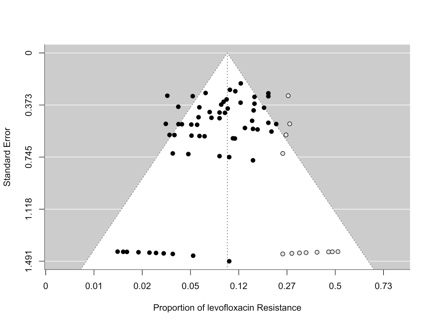

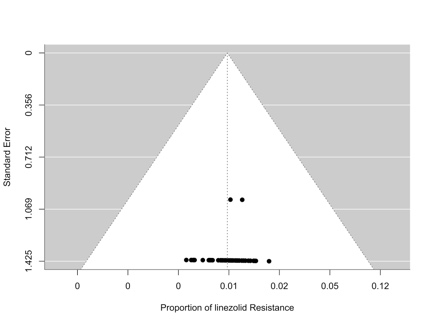


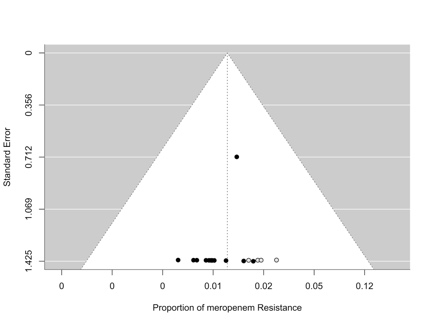

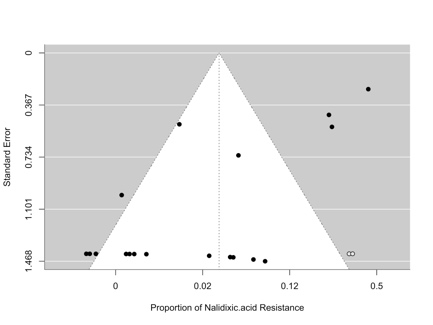

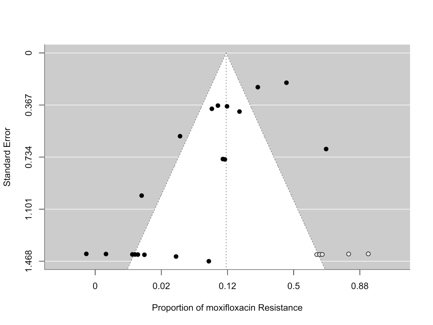

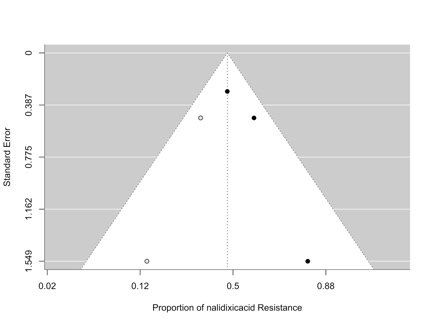


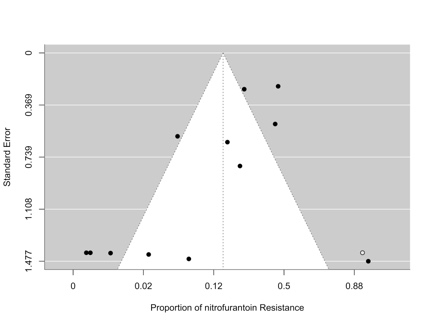

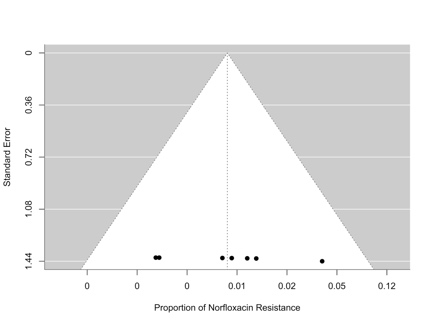

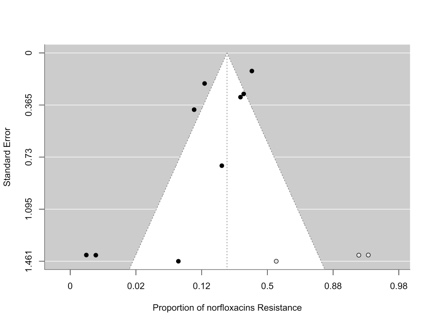

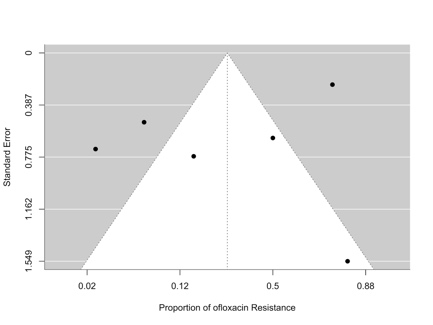

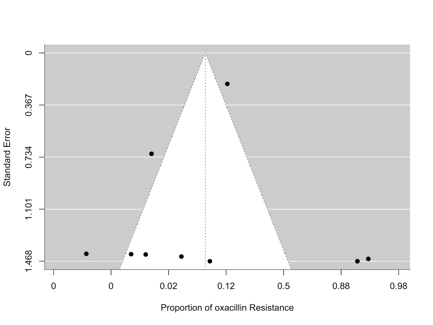

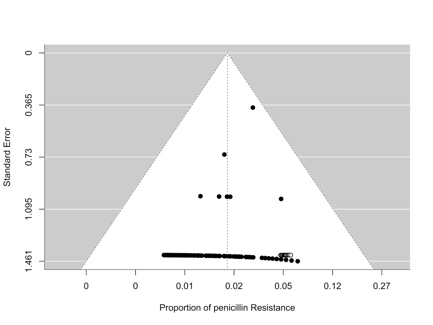

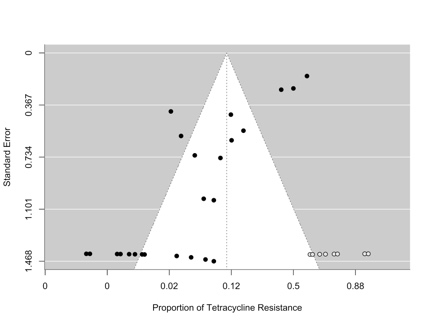

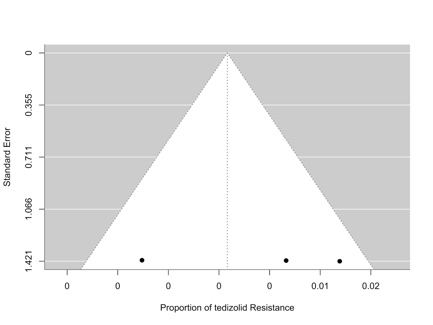


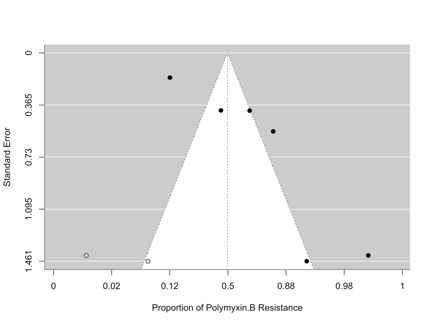

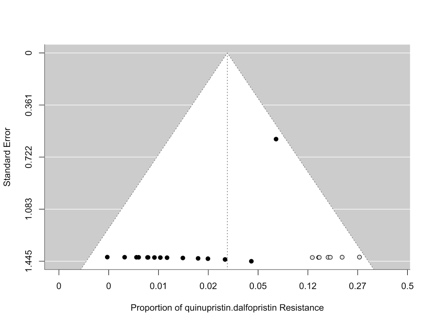

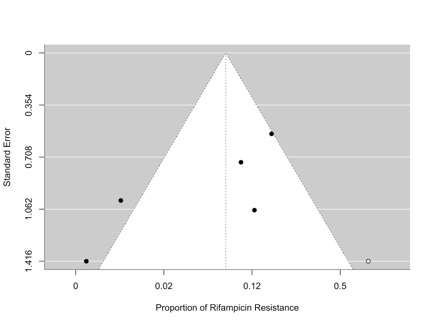

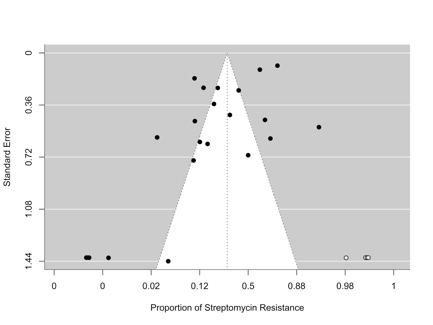


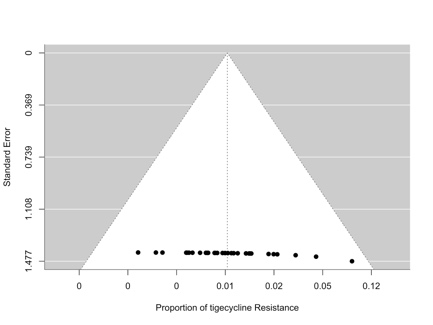

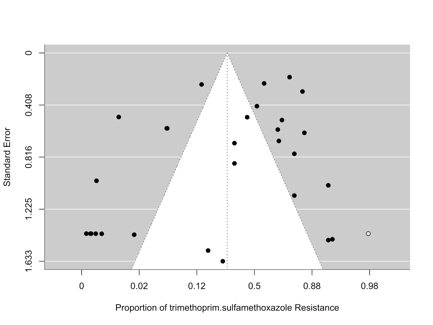

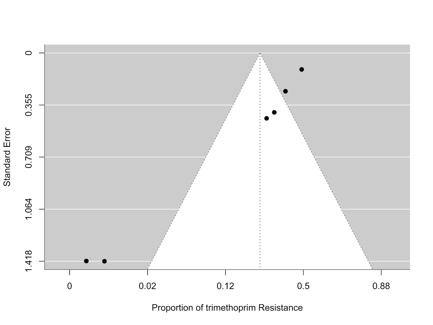

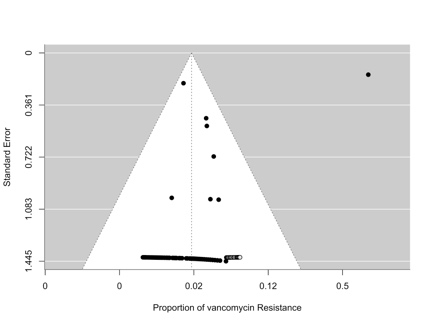


**Supplementary Figure 1:** Funnel Plots for Assessing Publication Bias in Antibiotic Resistance Studies.

# References

1. Stewart AG, Burnard D, Sowden D, McMillan D. Whole genome sequencing for antimicrobial resistance mechanisms, virulence factors and clonality in invasive Streptococcus agalactiae blood culture isolates recovered in Australia. Pathology. 2020;52(6):694-9.

2. Bob-Manuel M, McGee L, Igunma JA, Alex-Wele MA, Obunge OK, Wariso KT. Whole genome sequence based capsular typing and antimicrobial resistance prediction of Group B streptococcal isolates from colonized pregnant women in Nigeria. BMC genomics. 2021;22:1-6.

3. Abotorabi S, Rasooli Z, Pakniat H, Baloo F. Virulence Determinants, Serotypes, and Antimicrobial Resistance of Rectovaginal Isolates of Streptococcus agalactiae. Iranian Journal of Pathology. 2023;18(1):82.

4. Gomi Y, Wang L, Matsushima H, Kawabe A, Kikugawa A, Takagi A, et al. Variations in antibiotic susceptibility of group B Streptococcus in Japanese women: A long-term population-based cohort study. Taiwanese Journal of Obstetrics and Gynecology. 2019;58(6):805-7.

5. Miloshevski DS, V. Miloshevska, I. Vaginal colonization with group B streptococcus (streptococcus agalactiae). Acta Microbiologica Hellenica. 2015;60(3):172.

6. Ngom NS, Gassama O, Dieng A, Diakhaby EB, Ndiaye SML, Tine A, et al. Vaginal Carriage of Group B Streptococcus (GBS) in Pregnant Women, Antibiotic Sensitivity and Associated Risk Factors in Dakar, Senegal. Microbiology Insights. 2023;16:11786361231174419.

7. Mendes RE, Farrell DJ, Sader HS, Streit JM, Jones RN. Update of the telavancin activity in vitro tested against a worldwide collection of Gram-positive clinical isolates (2013), when applying the revised susceptibility testing method. Diagnostic Microbiology and Infectious Disease. 2015;81(4):275-9.

8. Kekic D, Gajic I, Opavski N, Kojic M, Vukotic G, Smitran A, et al. Trends in molecular characteristics and antimicrobial resistance of group B streptococci: A multicenter study in Serbia, 2015–2020. Scientific reports. 2021;11(1):540.

9. Karlowsky JA, Nichol K, Zhanel GG. Telavancin: mechanisms of action, in vitro activity, and mechanisms of resistance. Clinical Infectious Diseases. 2015;61(suppl_2):S58-S68.

10. Duncan LR, Sader HS, Smart JI, Flamm RK, Mendes RE. Telavancin activity in vitro tested against a worldwide collection of Gram-positive clinical isolates (2014). Journal of global antimicrobial resistance. 2017;10:271-6.

11. Mousavi SM, Nasaj M, Hosseini SM, Arabestani MR. Survey of strain distribution and antibiotic resistance pattern of group B streptococci (Streptococcus agalactiae) isolated from clinical specimens. GMS hygiene and infection control. 2016;11.

12. Matani CT, M. Matteini, A. Catalani, C. Messeri, D. Catalani, C. Streptococcus agalactiae:Prevalence of antimicrobial resistance in vaginal and rectal swabs in Italian pregnant women. Infezioni in Medicina. 2016;24(3):217-21.

13. Santana FAF, de Oliveira TVL, de Souza Filho MB, da Silva LSC, de Brito BB, de Melo FF, et al. Streptococcus agalactiae: Identification methods, antimicrobial susceptibility, and resistance genes in pregnant women. World Journal of Clinical Cases. 2020;8(18):3988.

14. de Figueiredo Sanches G, Lannes-Costa PS, Cristoforêto MC, Doran KS, Mattos-Guaraldi AL, Nagao PE. Streptococcus agalactiae strains isolated from cancer patients in Rio de Janeiro, Brazil. Brazilian Journal of Microbiology. 2021;52(1):303-10.

15. Laczeski MEN, M. G. Zapata, P. D. Vergara, M. I. First study of SCPB gene of streptococcus agalactiae in Misiones, Argentina. Biocell. 2014;38:132.

16. Belard S, Toepfner N, Capan-Melser M, Mombo-Ngoma G, Zoleko-Manego R, Groger M, et al. Streptococcus agalactiae serotype distribution and antimicrobial susceptibility in pregnant women in Gabon, Central Africa. Scientific reports. 2015;5(1):17281.

17. Nkembe NM, Kamga HG, Baiye WA, Chafa AB, Njotang PN. Streptococcus agalactiae prevalence and antimicrobial susceptibility pattern in vaginal and anorectal swabs of pregnant women at a tertiary hospital in Cameroon. BMC research notes. 2018;11:1-6.

18. Pimentel B, Martins C, Mendonça J, Miranda P, Sanches G, Mattos-Guaraldi A, et al. Streptococcus agalactiae infection in cancer patients: a five-year study. European journal of clinical microbiology & infectious diseases. 2016;35:927-33.

19. Dutra VG, Alves VM, Olendzki AN, Dias CA, de Bastos AF, Santos GO, et al. Streptococcus agalactiae in Brazil: serotype distribution, virulence determinants and antimicrobial susceptibility. BMC infectious diseases. 2014;14:1-9.

20. Shadbad MA, Kafil HS, Rezaee MA, Farzami MR, Dehkharghani AD, Sadeghi J, et al. Streptococcus agalactiae clinical isolates in Northwest Iran: antibiotic susceptibility, molecular typing, and biofilm formation. GMS hygiene and infection control. 2020;15.

21. Martins ER, Pedroso-Roussado C, Melo-Cristino J, Ramirez M, Infections PGftSoS. Streptococcus agalactiae causing neonatal infections in Portugal (2005–2015): diversification and emergence of a CC17/PI-2b multidrug resistant sublineage. Frontiers in microbiology. 2017;8:499.

22. Saad EJ, Baenas DF, Boisseau CS, García MJ, Núñez SA, Sanchez PE, et al. Bacteriemia por Streptococcus agalactiae en pacientes adultos y mujeres no embarazadas de dos hospitales universitarios. Revista argentina de microbiología. 2018;50(3):280-4.

23. Frej-Mądrzak M, Jama-Kmiecik A, Sarowska J, Teryks-Wołyniec D, Gryboś A, Gryboś M, et al. Streptococcus agalactiae and Chlamydia trachomatis detection in women without symptoms of infection. Advances in Clinical and Experimental Medicine. 2020;29(6):707-13.

24. Majigo MM, J. Luoga, A. Juma, J. Joachim, A. Spectrum and antimicrobial susceptibility patterns of pathogen causing neonatal sepsis at Regional Referral Hospital, Dar es Salaam Tanzania. Tanzania Journal of Health Research. 2022;23:167.

25. Shipitsyna E, Shalepo K, Zatsiorskaya S, Krysanova A, Razinkova M, Grigoriev A, et al. Significant shifts in the distribution of vaccine capsular polysaccharide types and rates of antimicrobial resistance of perinatal group B streptococci within the last decade in St. Petersburg, Russia. European Journal of Clinical Microbiology & Infectious Diseases. 2020;39(8):1487-93.

26. Metcalf B, Chochua S, Gertz Jr R, Hawkins P, Ricaldi J, Li Z, et al. Short-read whole genome sequencing for determination of antimicrobial resistance mechanisms and capsular serotypes of current invasive Streptococcus agalactiae recovered in the USA. Clinical Microbiology and Infection. 2017;23(8):574. e7-. e14.

27. Hon KL, Chow TC, Cheung TS, Lam WT, Hung LT, So KW, et al. Severe Group A and Group B Streptococcus Diseases at a Pediatric ICU: Are they Still Sensitive to the Penicillins? Current Clinical Pharmacology. 2020;15(2):125-31.

28. El-Gendy A-EA, Hassan SET, Gertz B, Bernard B, Ahmed MM, Elzohry HA, et al. Serotyping and Antibiotic Susceptibility of Invasive Streptococcus agalactiae in Egyptian Patients with or without Diabetes Mellitus. The American Journal of Tropical Medicine and Hygiene. 2021;105(6):1684.

29. Sulung F, Nik Zuraina NMN, Singh KKB, Hussin A, Hassan SA. Serotypes, virulence and antimicrobial susceptibility of Group B Streptococci isolated from infections in non-pregnant adults in Malaysia. Journal of Taibah University for Science. 2023;17(1):2241706.

30. Wang P, Tong J-j, Ma X-h, Song F-l, Fan L, Guo C-m, et al. Serotypes, antibiotic susceptibilities, and multi-locus sequence type profiles of Streptococcus agalactiae isolates circulating in Beijing, China. PLoS One. 2015;10(3):e0120035.

31. Wang S, Li L, Wu B, Wu W. Serotype, genotype, and clinical manifestations of Group B Streptococcus (GBS) isolated from neonates in China. Iranian Journal of Pediatrics. 2018;28(1).

32. Teatero S, Ferrieri P, Martin I, Demczuk W, McGeer A, Fittipaldi N. Serotype distribution, population structure, and antimicrobial resistance of group B Streptococcus strains recovered from colonized pregnant women. Journal of clinical microbiology. 2017;55(2):412-22.

33. Wang P, Ma Z, Tong J, Zhao R, Shi W, Yu S, et al. Serotype distribution, antimicrobial resistance, and molecular characterization of invasive group B Streptococcus isolates recovered from Chinese neonates. International Journal of Infectious Diseases. 2015;37:115-8.

34. Slotved H-C, Møller JK, Khalil MR, Nielsen SY. The serotype distribution of Streptococcus agalactiae (GBS) carriage isolates among pregnant women having risk factors for early-onset GBS disease: a comparative study with GBS causing invasive infections during the same period in Denmark. BMC infectious diseases. 2021;21:1-8.

35. Wang Y-H, Chen C-L, Hou J-N, Wang Y-R, Lin T-Y, Wang M-H, et al. Serotype distribution and resistance genes associated with macrolide and fluoroquinolone resistance in Streptococcus agalactiae isolates from a hospital in southern Taiwan. Biomedical Journal. 2015;38(3).

36. Fröhlicher S, Reichen-Fahrni G, Müller M, Surbek D, Droz SC, Spellerberg B, et al. Serotype distribution and antimicrobial susceptibility of group B streptococci in pregnant women: results from a Swiss tertiary centre. Swiss medical weekly. 2014;144:w13935.

37. Ikebe T, Okuno R, Uchitani Y, Takano M, Yamaguchi T, Otsuka H, et al. Serotype distribution and antimicrobial resistance of Streptococcus agalactiae isolates in nonpregnant adults with streptococcal toxic shock syndrome in Japan in 2014 to 2021. Microbiology Spectrum. 2023;11(2):e04987-22.

38. Størdal EH, Solevåg AL, Bjørnholt JV, Rønnestad A, Stensvold HJ. Sepsis treatment options identified by 10‐year study of microbial isolates and antibiotic susceptibility in a level‐four neonatal intensive care unit. Acta Paediatrica. 2022;111(3):519-26.

39. Alp F, Findik D, Dagi HT, Arslan U, Pekin AT, Yilmaz SA. Screening and genotyping of group B streptococcus in pregnant and non-pregnant women in Turkey. The Journal of Infection in Developing Countries. 2016;10(03):222-6.

40. Ngonzi J, Bebell LM, Bazira J, Fajardo Y, Nyehangane D, Boum Y, et al. Risk Factors for Vaginal Colonization and Relationship between Bacterial Vaginal Colonization and In‐Hospital Outcomes in Women with Obstructed Labor in a Ugandan Regional Referral Hospital. International Journal of Microbiology. 2018;2018(1):6579139.

41. Bae HG, Hong J, Kim Y-J, Lee K-R, Lee K, Choi SJ, et al. A retrospective national study on colonization rate and antimicrobial susceptibility of Streptococcus agalactiae in pregnant Korean women, 2018–2020. Yonsei medical journal. 2022;63(8):717.

42. Kitamura M, Kimura K, Ido A, Seki T, Banno H, Jin W, et al. Relatively high rates of cefotaxime-and ceftriaxone-non-susceptible isolates among group B streptococci with reduced penicillin susceptibility (PRGBS) in Japan. Journal of Antimicrobial Chemotherapy. 2019;74(4):931-4.

43. Cooper K, Abbott F, Gould I. Reduced penicillin susceptibility of group B Streptococcus: an assessment of emergence in Grampian, Scotland. British Journal of Biomedical Science. 2016;73(1):25-7.

44. Alemán T, Vielot NA, Herrera R, Velasquez R, Berrios T, Toval-Ruíz C, et al. Rectovaginal Colonization with Serotypes of Group B Streptococci with Reduced Penicillin Susceptibility among Pregnant Women in León, Nicaragua. Pathogens. 2022;11(4):415.

45. Choi SJ, Kang J, Uh Y. Recent epidemiological changes in group B streptococcus among pregnant Korean women. Annals of laboratory medicine. 2021;41(4):380-5.

46. Rasamiravaka TB, E. Ranaivosoa, M. K. Rasamindrakotroka, A. Profile and antimicrobial resistance to newly available drugs of urinary tract pathogens among Malagasy pregnant women. Tropical Biomedicine. 2016;33(1):135-40.

47. Safari D, Gultom SM, Tafroji W, Azzahidah A, Soesanti F, Khoeri MM, et al. Prevalence, serotype and antibiotic susceptibility of Group B Streptococcus isolated from pregnant women in Jakarta, Indonesia. PLoS One. 2021;16(5):e0252328.

48. Motallebirad T, Fazeli H, Ghahiri A, Shokri D, Jalalifar S, Moghim S, et al. Prevalence, population structure, distribution of serotypes, pilus islands and resistance genes among erythromycin-resistant colonizing and invasive Streptococcus agalactiae isolates recovered from pregnant and non-pregnant women in Isfahan, Iran. Bmc Microbiology. 2021;21:1-11.

49. Dube R, Al-Zuheiri STS, Syed M, Harilal L, Zuhaira DAL, Kar SS. Prevalence, clinico-bacteriological profile, and antibiotic resistance of symptomatic urinary tract infections in pregnant women. Antibiotics. 2022;12(1):33.

50. Jisuvei SC, Osoti A, Njeri MA. Prevalence, antimicrobial susceptibility patterns, serotypes and risk factors for group B streptococcus rectovaginal isolates among pregnant women at Kenyatta National Hospital, Kenya; a cross-sectional study. BMC infectious diseases. 2020;20:1-9.

51. Husen O, Kannaiyan Abbai M, Aliyo A, Daka D, Gemechu T, Tilahun D, et al. Prevalence, antimicrobial susceptibility pattern and associated factors of group B Streptococcus among pregnant women attending antenatal Care at Bule Hora University Teaching Hospital, Southern Ethiopia. Infection and Drug Resistance. 2023:4421-33.

52. Bitew A, Mengist A, Belew H, Aschale Y, Reta A. The prevalence, antibiotic resistance pattern, and associated factors of bacterial vaginosis among women of the reproductive age group from felege hiwot referral hospital, Ethiopia. Infection and Drug Resistance. 2021:2685-96.

53. Abdallah MS, Philemon R, Kadri A, Al-Hinai A, Saajan AM, Gidabayda JG, et al. Prevalence, aetiological agents, and antimicrobial sensitivity pattern of bacterial meningitis among children receiving care at KCMC referral hospital in Tanzania. The East African Health Research Journal. 2018;2(1):1.

54. Mišić M, Arsović A, Čukić J, Rosić M, Tošić-Pajić J, Manojlović N, et al. Učestalost rezistencije na makrolide i linkozamide kod ambulantnih i bolničkih izolata stafilokoka i streptokoka u jugoistočnoj Srbiji. Srpski arhiv za celokupno lekarstvo. 2018;146(7-8):384-90.

55. Sahraee S, Milani F, Roushan ZA, Ch MH, Rostami S, Shoja S, et al. The prevalence of rectovaginal colonization and antibiotic susceptibility pattern of Streptococcus agalactiae in pregnant women in Al-Zahra hospital, Rasht, Iran. Infectious Diseases in Clinical Practice. 2019;27(3):143-7.

56. Woldu ZL, Teklehaimanot TG, Waji ST, Gebremariam MY. The prevalence of Group B Streptococus recto-vaginal colonization and antimicrobial susceptibility pattern in pregnant mothers at two hospitals of Addis Ababa, Ethiopia. Reproductive health. 2014;11:1-4.

57. Soares GCT, Alviano DS, Santos GdS, Alviano CS, Mattos-Guaraldi AL, Nagao PE. Prevalence of Group B Streptococcus serotypes III and V in pregnant women of Rio de Janeiro, Brazil. Brazilian Journal of Microbiology. 2013;44:869-72.

58. Ge Y, Pan F, Bai R, Mao Y, Ji W, Wang F, et al. Prevalence of group B streptococcus colonization in pregnant women in Jiangsu, East China. BMC Infectious Diseases. 2021;21(1):492.

59. Ojo OO, Awonuga D, Dedeke IOF, Nwadike VU, Adenaya OR, Odelola OI. Prevalence of Group B Streptococcus Colonisation and Antimicrobial Susceptibility Pattern among Pregnant Women Attending a Tertiary Health Facility in Ogun State, Southwest Nigeria. Journal of West African College of Surgeons. 2019;9(3):8-14.

60. Al Romaihi EAK, S. Saeed, N. Barakat, A. M. El Moez Azam, N. F. A. Haji, S. Prevalence of antimicrobial resistance in uropathogens among patients visiting primary health centers: Implications for empiric therapy. Bahrain Medical Bulletin. 2018;40(4):207-11.

61. Al-Tulaibawi NAJ. Prevalence and sensitivity of bacterial urinary tract infection among adult diabetic patients in misan province, Iraq. Journal of Pure and Applied Microbiology. 2019;13(2):847-53.

62. Qadi M, AbuTaha A, Al-Shehab Ry, Sulaiman S, Hamayel A, Hussein A, et al. Prevalence and risk factors of Group B Streptococcus Colonization in pregnant women: a pilot study in Palestine. Canadian Journal of Infectious Diseases and Medical Microbiology. 2021;2021(1):8686550.

63. Haimbodi EL, Mukesi M, Moyo SR. Prevalence and molecular characterization of group B streptococcus in pregnant women from hospitals in Ohangwena and Oshikoto regions of Namibia. BMC microbiology. 2021;21:1-9.

64. O. Luiz FBd, Alves KB, Barros RR. Prevalence and long-term persistence of beta-haemolytic streptococci throat carriage among children and young adults. Journal of medical microbiology. 2019;68(10):1526-33.

65. Njoku C, Emechebe C, Agbakwuru A. prevalence and determinants of anogenital colonization by Group B Streptococcus infection among HIV positive and negative women in Calabar, Nigeria. Int J Women’s Health Reprod Sci. 2017;6(1):11-7.

66. Jiang H, Su M, Kui L, Huang H, Qiu L, Li L, et al. Prevalence and antibiotic resistance profiles of cerebrospinal fluid pathogens in children with acute bacterial meningitis in Yunnan province, China, 2012-2015. PloS one. 2017;12(6):e0180161.

67. Numanović F, Smajlović J, Gegić M, Delibegović Z, Bektaš S, Halilović E, et al. Presence and resistance of Streptococcus agalactiae in vaginal specimens of pregnant and adult non-pregnant women and association with other aerobic bacteria. Med Glas (Zenica). 2017;14(1):98-105.

68. Nagano N, Koide S, Hayashi W, Taniguchi Y, Tanaka H, Maeyama Y, et al. Population-level transition of capsular polysaccharide types among sequence type 1 group B Streptococcus isolates with reduced penicillin susceptibility during a long-term hospital epidemic. International journal of antimicrobial agents. 2019;53(3):203-10.

69. Teatero S, McGeer A, Li A, Gomes J, Seah C, Demczuk W, et al. Population structure and antimicrobial resistance of invasive serotype IV group B Streptococcus, Toronto, Ontario, Canada. Emerging infectious diseases. 2015;21(4):585.

70. Jamrozy DB, M. W. de Goffau, M. C. van de Beek, D. Kuijpers, T. W. Parkhill, J. van der Ende, A. Bentley, S. D. Increasing incidence of group B streptococcus neonatal infections in the Netherlands is associated with clonal expansion of CC17 and CC23. Scientific reports. 2020;10(1):9539.

71. Khodaei F, Najafi M, Hasani A, Kalantar E, Sharifi E, Amini A, et al. Pilus–encoding islets in S. agalactiae and its association with antibacterial resistance and serotype distribution. Microbial pathogenesis. 2018;116:189-94.

72. Wang X, Cao X, Li S, Ou Q, Lin D, Yao Z, et al. Phenotypic and molecular characterization of Streptococcus agalactiae colonized in Chinese pregnant women: predominance of ST19/III and ST17/III. Research in microbiology. 2018;169(2):101-7.

73. Novosak M, Bobadilla F, Delgado O, Vergara M, Laczeski M. Phenotypic and genotypic characterization of resistance to macrolides and lincosamides in Streptococcus agalactiae isolated from pregnant women in Misiones, Argentina. Microbial Drug Resistance. 2020;26(12):1472-81.

74. Wu B, Su J, Li L, Wu W, Wu J, Lu Y, et al. Phenotypic and genetic differences among group B Streptococcus recovered from neonates and pregnant women in Shenzhen, China: 8-year study. BMC microbiology. 2019;19:1-9.

75. Malek-Jafarian MH, F. S. Ahmadi, A. R. Pattern of infection and antibiotic activity among Streptococcus agalactiae isolates from adults in Mashhad, Iran. Rep Biochem Mol Biol. 2015;3(2):89-93.

76. Li Y, Kong C, To WW. Pathogens in preterm prelabour rupture of membranes and erythromycin for antibiotic prophylaxis: a retrospective analysis. Hong Kong Medical Journal. 2019;25(4):287.

77. Liu JF, Z. Yu, Y. Ding, Y. Liu, Z. Zhang, C. He, H. Geng, H. Chen, W. Zhao, G. Liu, Q. Wang, B. Sun, X. Wang, S. Sun, R. Fu, D. Liu, X. Huang, L. Li, J. Xing, X. Wang, X. Gao, Y. Zhu, R. Han, M. Peng, F. Geng, M. Deng, L. Pathogens distribution and antimicrobial resistance in bloodstream infections in twenty-five neonatal intensive care units in China, 2017–2019. Antimicrob Resist Infect Control. 2021;10(1).

78. Horn DL, Roberts EA, Shen J, Chan JD, Bulger EM, Weiss NS, et al. Outcomes of β-hemolytic streptococcal necrotizing skin and soft-tissue infections and the impact of clindamycin resistance. Clinical Infectious Diseases. 2021;73(11):e4592-e8.

79. Horn DLR, E. A. Shen, J. Chan, J. D. Bulger, E. M. Weiss, N. S. Lynch, J. B. Bryson-Cahn, C. Robinson, B. R. H. Outcomes of β-Hemolytic Streptococcal Necrotizing Skin and Soft-tissue Infections and the Impact of Clindamycin Resistance. Clin Infect Dis. 2021;73(11):E4592-E8.

80. Li X, Gao W, Jia Z, Yao K, Yang J, Tong J, et al. Characterization of Group B Streptococcus Recovered from Pregnant Women and Newborns Attending in a Hospital in Beijing, China. Infection and Drug Resistance. 2023:2549-59.

81. Li G, Wei Y, Guo Y, Gong H, Lian J, Xu G, et al. Omadacycline efficacy against Streptococcus agalactiae isolated in China: correlation between resistance and virulence gene and Biofilm formation. Computational Intelligence and Neuroscience. 2022;2022(1):7636983.

82. Hamad AA, Farrag AA, El-Waseif AA, Almawla SOG. Novel molecular diagnosis of Cyl E, Spb 1 and bib A virulence genes of Streptococcus agalactiae from pregnant women. Materials Today: Proceedings. 2023;80:2347-52.

83. Yayan J, Ghebremedhin B, Rasche K. No resistance to penicillin, cefuroxime, cefotaxime, or vancomycin in Pneumococcal pneumonia. International Journal of Medical Sciences. 2015;12(12):980.

84. Gizachew M, Tiruneh M, Moges F, Adefris M, Tigabu Z, Tessema B. Newborn colonization and antibiotic susceptibility patterns of Streptococcus agalactiae at the University of Gondar Referral Hospital, Northwest Ethiopia. BMC pediatrics. 2018;18:1-11.

85. Mudzikati L, Dramowski A. Neonatal septicaemia: prevalence and antimicrobial susceptibility patterns of common pathogens at Princess Marina Hospital, Botswana. Southern African Journal of Infectious Diseases. 2015;30(3):96-101.

86. Venkatnarayan K, Bej P, Thapar R. Neonatal Sepsis: A Profile of a Changing Spectrum. Journal of Nepal Paediatric Society. 2014;34(3).

87. Dehdashtian M, Moosavian M, Boghrati M, Arshadi M, Malakian A, Aramesh MR. Neonatal nasopharyngeal bacterial colonization: Prevalence, antimicrobial resistance, and concomitant early-onset sepsis. Jundishapur Journal of Microbiology. 2021;14(5).

88. Creti R, Imperi M, Berardi A, Pataracchia M, Recchia S, Alfarone G, et al. Neonatal group B streptococcus infections: prevention strategies, clinical and microbiologic characteristics in 7 years of surveillance. The Pediatric Infectious Disease Journal. 2017;36(3):256-62.

89. Guo D, Cao X, Li S, Ou Q, Lin D, Yao Z, et al. Neonatal colonization of group B Streptococcus in China: prevalence, antimicrobial resistance, serotypes, and molecular characterization. American Journal of Infection Control. 2018;46(3):e19-e24.

90. Ghamari M, Jabalameli F, Emaneini M, Beigverdi R. Multiple-locus variable-number tandem repeat analysis for genotyping of erythromycin-resistant group B streptococci in Iran. New microbes and new infections. 2022;45:100957.

91. Alzayer M, Alkhulaifi MM, Alyami A, Aldosary M, Alageel A, Garaween G, et al. Molecular typing and antimicrobial resistance of group B Streptococcus clinical isolates in Saudi Arabia. Journal of Global Antimicrobial Resistance. 2023;35:244-51.

92. Gharabeigi N, Bafroee AST, Amini K. Molecular serotyping and antibiotic resistance profile of group B Streptococcus strains isolated from iranian pregnant women with urinary tract infection. Iranian Journal of Medical Sciences. 2023;48(6):542.

93. Dehbashi SP, M. R. Mahmoudi, M. Mashhadi, R. Molecular identification of streptococcus agalactiae using gbsl805 gene and determination of the antibiotic susceptibility pattern of isolates. Journal of Babol University of Medical Sciences. 2015;16(14):29-35.

94. Schuab RB, Arêas GP, Souza VC, Barros RR. Molecular epidemiology of Streptococcus agalactiae recovered from significant bacteriuria. Infectious Diseases. 2015;47(9):637-42.

95. Gajic I, Plainvert C, Kekic D, Dmytruk N, Mijac V, Tazi A, et al. Molecular epidemiology of invasive and non-invasive group B Streptococcus circulating in Serbia. International Journal of Medical Microbiology. 2019;309(1):19-25.

96. Bergal A, Loucif L, Benouareth D, Bentorki A, Abat C, Rolain J-M. Molecular epidemiology and distribution of serotypes, genotypes, and antibiotic resistance genes of Streptococcus agalactiae clinical isolates from Guelma, Algeria and Marseille, France. European Journal of Clinical Microbiology & Infectious Diseases. 2015;34:2339-48.

97. Shrestha K, Sah AK, Singh N, Parajuli P, Adhikari R. Molecular characterization of Streptococcus agalactiae isolates from pregnant women in Kathmandu City. Journal of tropical medicine. 2020;2020(1):4046703.

98. Gizachew M, Tiruneh M, Moges F, Adefris M, Tigabu Z, Tessema B. Molecular characterization of Streptococcus agalactiae isolated from pregnant women and newborns at the University of Gondar Comprehensive Specialized Hospital, Northwest Ethiopia. BMC Infectious Diseases. 2020;20:1-9.

99. Jiang H, Chen M, Li T, Liu H, Gong Y, Li M. Molecular characterization of Streptococcus agalactiae causing community-and hospital-acquired infections in Shanghai, China. Frontiers in microbiology. 2016;7:1308.

100. Simoni S, Vincenzi C, Brenciani A, Morroni G, Bagnarelli P, Giovanetti E, et al. Molecular characterization of Italian isolates of fluoroquinolone-resistant Streptococcus agalactiae and relationships with chloramphenicol resistance. Microbial Drug Resistance. 2018;24(3):225-31.

101. Moltó-García B, del Carmen Liébana-Martos M, Cuadros-Moronta E, Rodríguez-Granger J, Sampedro-Martínez A, Rosa-Fraile M, et al. Molecular characterization and antimicrobial susceptibility of hemolytic Streptococcus agalactiae from post-menopausal women. Maturitas. 2016;85:5-10.

102. Kawaguchiya M, Urushibara N, Aung MS, Shimada S, Nakamura M, Ito M, et al. Molecular characterization and antimicrobial resistance of Streptococcus agalactiae isolated from pregnant women in Japan, 2017–2021. IJID regions. 2022;4:143-5.

103. Li J, Ji W, Gao K, Zhou H, Zhang L, Mu X, et al. Molecular characteristics of group B Streptococcus isolates from infants in southern mainland China. BMC Infectious Diseases. 2019;19:1-8.

104. Tsai M-H, Hsu J-F, Lai M-Y, Lin L-C, Chu S-M, Huang H-R, et al. Molecular characteristics and antimicrobial resistance of group B Streptococcus strains causing invasive disease in neonates and adults. Frontiers in microbiology. 2019;10:264.

105. Lu B, Chen X, Wang J, Wang D, Zeng J, Li Y, et al. Molecular characteristics and antimicrobial resistance in invasive and noninvasive Group B Streptococcus between 2008 and 2015 in China. Diagnostic microbiology and infectious disease. 2016;86(4):351-7.

106. Liu Z, Jiang X, Li J, Ji W, Zhou H, Gong X, et al. Molecular characteristics and antibiotic resistance mechanisms of clindamycin-resistant Streptococcus agalactiae isolates in China. Frontiers in Microbiology. 2023;14:1138039.

107. Dobrut A, Ochońska D, Brzozowska E, Górska S, Kaszuba-Zwoinska J, Gołda-Cępa M, et al. Molecular characteristic, antibiotic resistance, and detection of highly immunoreactive proteins of Group B Streptococcus strains isolated from urinary tract infections in polish adults. Frontiers in Microbiology. 2022;13:809724.

108. Al Benwan K, Al Banwan D. Microbiological Profiles and Inflammatory Biomarkers of Bacteremia in Children in a Teaching Hospital in Kuwait: An 8-Year Retrospective Study. Medical Principles and Practice. 2024;33(1):21-30.

109. Wilkie GL, Prabhu M, Ona S, Easter SR, Tuomala RE, Riley LE, et al. Microbiology and antibiotic resistance in peripartum bacteremia. Obstetrics & Gynecology. 2019;133(2):269-75.

110. Qiu Y, Yang J, Chen Y, Yang J, Zhu Q, Zhu C, et al. Microbiological profiles and antimicrobial resistance patterns of pediatric bloodstream pathogens in China, 2016–2018. European Journal of Clinical Microbiology & Infectious Diseases. 2021;40:739-49.

111. Ali M, Alamin MA, A. Ali G, Alzubaidi K, Ali B, Ismail A, et al. Microbiological and clinical characteristics of invasive Group B Streptococcal blood stream infections in children and adults from Qatar. BMC Infectious Diseases. 2022;22(1):881.

112. Lu B, Wu J, Chen X, Gao C, Yang J, Li Y, et al. Microbiological and clinical characteristics of Group B Streptococcus isolates causing materno-neonatal infections: high prevalence of CC17/PI-1 and PI-2b sublineage in neonatal infections. Journal of medical microbiology. 2018;67(11):1551-9.

113. Hiriote WT-U, S. Tor-Udom, P. Maternal to child group B Streptococcus transmission rate at Thammasat University hospital, Thailand. Southeast Asian Journal of Tropical Medicine and Public Health. 2017;48(4):841-9.

114. Liu P, Feng Q, Liang Y, Wang X, Xiao Z, Huang L, et al. Maternal group B streptococcal rectovaginal colonization after intrapartum antibiotic prophylaxis. Children. 2022;9(12):1848.

115. Madrid L, Maculuve SA, Vilajeliu A, Sáez E, Massora S, Cossa A, et al. Maternal carriage of group B Streptococcus and Escherichia coli in a district hospital in Mozambique. The Pediatric infectious disease journal. 2018;37(11):1145-53.

116. Rostami S, Moeineddini L, Ghandehari F, Khorasani MR, Shoaei P, Ebrahimi N. Macrolide-resistance, capsular genotyping and associated factors of group B Streptococci colonized pregnant women in Isfahan, Iran. Iranian Journal of Microbiology. 2021;13(2):183.

117. Shabayek S, Abdalla S. Macrolide-and tetracycline-resistance determinants of colonizing group B streptococcus in women in Egypt. Journal of medical microbiology. 2014;63(10):1324-7.

118. Houri H, Kazemian H, Ebrahim-Saraie HS, Taji A, Tayebi Z, Heidari H. Linezolid activity against clinical Gram-positive cocci with advanced antimicrobial drug resistance in Iran. Journal of global antimicrobial resistance. 2017;10:200-3.

119. Moroi H, Kimura K, Kotani T, Tsuda H, Banno H, Jin W, et al. Isolation of group B Streptococcus with reduced β-lactam susceptibility from pregnant women. Emerging microbes & infections. 2019;8(1):2-7.

120. Abdel-Shafi S, Al-Mohammadi A-R, Hamdi S, Moustafa AH, Enan G. Biological Characterization and Inhibition of Streptococcus pyogenes ZUH1 Causing Chronic Cystitis by Crocus sativus Methanol Extract, Bee Honey Alone or in Combination with Antibiotics: An In Vitro Study. Molecules. 2019;24(16):2903.

121. Yan YZH, H. Lu, T. Y. Fan, H. Q. Hu, Y. Li, G. Zhang, X. H. Shi, Y. Xia, R. Investigation of serotype distribution and resistance genes profile in group B Streptococcus isolated from pregnant women: a Chinese multicenter cohort study. Apmis. 2016;124(9):794-9.

122. Ebrahem AM, Jameel SK, Abbas AH. I nvestigation of Biofilm Formation and Antibiotic Resistant of Bacteria Isolated from Septic Neonates. J Med Chem Sci. 2023;6(4):816-26.

123. Al Abbas LA, Khaddam W, Shreibati F. INVESTIGATING BACTERIA ISOLATED FROM DIABETIC FOOT ULCERS AND STUDYING THEIR SENSITIVITY TO ANTIBIOTICS–SYRIA. Bulletin of Pharmaceutical Sciences Assiut. 2022;45(1):451-8.

124. Vuillemin X, Hays C, Plainvert C, Dmytruk N, Louis M, Touak G, et al. Invasive group B Streptococcus infections in non-pregnant adults: a retrospective study, France, 2007–2019. Clinical Microbiology and Infection. 2021;27(1):129. e1-. e4.

125. Graux E, Hites M, Martiny D, Maillart E, Delforge M, Melin P, et al. Invasive group B Streptococcus among non-pregnant adults in Brussels-Capital Region, 2005–2019. European Journal of Clinical Microbiology & Infectious Diseases. 2021;40(3):515-23.

126. Zhang JZ, R. Dong, Y. Zheng, Y. Invasive group B streptococcal infection in infants in Shenzhen, China. Int J Clin Exp Med. 2015;8(2):2939-43.

127. Sigaúque B, Kobayashi M, Vubil D, Nhacolo A, Chaúque A, Moaine B, et al. Invasive bacterial disease trends and characterization of group B streptococcal isolates among young infants in southern Mozambique, 2001–2015. PLoS One. 2018;13(1):e0191193.

128. Al-Subol I, Abdul-Aziz M, Almikhlafy AA, Alqahtani T. An Initial Survey on the Prevalence of Group B Streptococcus (GBS) among Yemeni Pregnant Women in Sana’a City. Infectious Diseases in Obstetrics and Gynecology. 2022;2022(1):6279343.

129. Alani SN, AlMeani SAL. Inhibition of Streptococcus Agalactiae Biofilm Formation in Response to Purified Phytochemical Antimicrobial Materials. Journal of Pharmaceutical Negative Results. 2022;13(4):601-7.

130. Kaminska D, Ratajczak M, Szumała-Kąkol A, Dlugaszewska J, Nowak-Malczewska DM, Gajecka M. Increasing resistance and changes in distribution of serotypes of Streptococcus agalactiae in Poland. Pathogens. 2020;9(7):526.

131. Lopes EF, T. Machado, M. P. Carriço, J. Cristino, J. M. Ramirez, M. Martins, E. Increase of macrolide resistance among group B Streptococcus invasive disease in non-pregnant adults in Portugal (2009- 2015) was driven by a capsular variant of a single clone. International Journal of Antimicrobial Agents. 2017;50:S116.

132. Gao K, Fu J, Guan X, Zhu S, Zeng L, Xu X, et al. Incidence, bacterial profiles, and antimicrobial resistance of culture-proven neonatal sepsis in south China. Infection and Drug Resistance. 2019:3797-805.

133. EL-Lakany RR, Abdelmaged ES, Shams M, Hassan R, Rizk DE. Incidence of virulence determinants among Streptococcus agalactiae isolated from pregnant women and association with their serotypes. Egyptian Journal of Basic and Applied Sciences. 2023;10(1):650-70.

134. Renteria M, Biedenbach D, Bouchillon S, Hoban D, Raghubir N, Sajben P, et al. In vitro activity of tigecycline against isolates collected from complicated skin and skin structure infections and intra-abdominal infections in Africa and Middle East countries: TEST 2007–2012. Diagnostic Microbiology and Infectious Disease. 2014;79(1):54-9.

135. Biedenbach DJ, Arhin FF, Moeck G, Lynch TF, Sahm DF. In vitro activity of oritavancin and comparator agents against staphylococci, streptococci and enterococci from clinical infections in Europe and North America, 2011–2014. International journal of antimicrobial agents. 2015;46(6):674-81.

136. Karlowsky JA, Walkty AJ, Baxter MR, Arhin FF, Moeck G, Adam HJ, et al. In vitro activity of Oritavancin against gram-positive pathogens isolated in Canadian hospital laboratories from 2011 to 2015. Diagnostic Microbiology and Infectious Disease. 2017;87(4):349-56.

137. Karlowsky J, Walkty A, Baxter M, Adam H, Zhanel G. In vitro activity of oritavancin against Gram-positive pathogens isolated in Canadian hospital laboratories from 2011 to 2013. Diagnostic Microbiology and Infectious Disease. 2014;80(4):311-5.

138. Piérard D, Stone GG. In vitro activity of ceftaroline and comparators against bacterial isolates collected globally from patients with skin infections. Journal of global antimicrobial resistance. 2021;26:4-10.

139. Karlowsky JA, Biedenbach DJ, Bouchillon SK, Hackel M, Iaconis JP, Sahm DF. In vitro activity of Ceftaroline against bacterial pathogens isolated from patients with skin and soft tissue and respiratory tract infections in African and Middle Eastern countries: AWARE global surveillance program 2012–2014. Diagnostic microbiology and infectious disease. 2016;86(2):194-9.

140. Mohamed N, Valdez RR, Fandiño C, Baudrit M, Falci DR, Murillo JDC. In vitro activity of ceftaroline against bacterial isolates causing skin and soft tissue and respiratory tract infections collected in Latin American countries, ATLAS program 2016–2020. Journal of Global Antimicrobial Resistance. 2024;36:4-12.

141. Hsueh PR. In vitro activities of tedizolid and linezolid against Grampositive cocci associated with acute bacterial skin and skin structure infections and pneumonia. International Journal of Antimicrobial Agents. 2015;45:S70.

142. Lee W-T, Lai M-C. High prevalence of Streptococcus agalactiae from vaginas of women in Taiwan and its mechanisms of macrolide and quinolone resistance. Journal of Microbiology, Immunology and Infection. 2015;48(5):510-6.

143. Kardos S, Tóthpál A, Laub K, Kristóf K, Ostorházi E, Rozgonyi F, et al. High prevalence of group B streptococcus ST17 hypervirulent clone among non-pregnant patients from a Hungarian venereology clinic. BMC infectious diseases. 2019;19:1-10.

144. Emaneini MM, A. Beigvierdi, R. Fooladi, A. A. Asadi, F. Jabalameli, F. Taherikalani, M. High Incidence of Macrolide and Tetracycline Resistance among Streptococcus Agalactiae Strains Isolated from Clinical Samples in Tehran, Iran. Maedica (Bucur). 2014;9(2):157-61.

145. Girma W, Yimer N, Kassa T, Yesuf E. Group B Streptococcus recto-vaginal colonization in near-term pregnant women, Southwest Ethiopia. Ethiopian Journal of Health Sciences. 2020;30(5).

146. Proudmore K, Swe MNN, Leitch M, Clayfield K, Hennessy J, Baird R. Group B streptococcus in the Northern Territory in 2023: clindamycin down but not out. Communicable Diseases Intelligence. 2023;47.

147. Mohamed AM, Khan MA, Faiz A, Ahmad J, Khidir EB, Basalamah MA, et al. Group B Streptococcus colonization, antibiotic susceptibility, and serotype distribution among Saudi pregnant women. Infection & chemotherapy. 2020;52(1):70.

148. Biobaku Oluwafunmilola R, Olaleye Atinuke O, Adefusi Olorunwa F, Adeyemi Babalola A, Onipede Anthony O, Loto Olabisi M, et al. Group B streptococcus colonization and HIV in pregnancy: A cohort study in Nigeria. Journal of Neonatal-Perinatal Medicine. 2017;10(1):91-7.

149. Gogoi M, Das MK, Das JK, Barman N, Das P, Devi U. Group B Streptococcus Colonising the Genital Tract of Pregnant Women from Dibrugarh, Assam: Circulating Serotypes, Susceptibility Pattern and Phylogenetic Analysis. J Clin Diagn Res. 2021;15(4):13-8.

150. Dilrukshi G, Kottahachchi J, Dissanayake D, Pathiraja R, Karunasingha J, Sampath M, et al. Group B Streptococcus colonisation and their antimicrobial susceptibility among pregnant women attending antenatal clinics in tertiary care hospitals in the Western Province of Sri Lanka. Journal of Obstetrics and Gynaecology. 2021;41(1):1-6.

151. Dong Y, Jiang S-Y, Zhou Q, Cao Y. Group B Streptococcus causes severe sepsis in term neonates: 8 years experience of a major Chinese neonatal unit. World Journal of Pediatrics. 2017;13:314-20.

152. Iweriebor BC, Afolabi KO, Stofile PZ, Obi LC. Group B Streptococcus agalactiae resistant to recommended intrapartum antibiotic prophylaxis isolated from the rectovaginal area of 35-37 weeks pregnant women. Malaysian Journal of Microbiology. 2023;19(5).

153. Leykun Y, Genet C, Mulu W. Group B streptococci vaginal-recto colonization, vertical transmission to newborns, antimicrobial susceptibility profile and associated factors in selected health facilities of Bahir Dar city: a cross-sectional study. Infection and Drug Resistance. 2021:5457-72.

154. Kumalo A, Gebre B, Shiferaw S, Wolde W, Shonde T. Group B Streptococci recto-vaginal colonization, antimicrobial susceptibility pattern, and associated factors among pregnant women at selected health facilities of Wolaita Sodo Town, Southern Ethiopia. Frontiers in Microbiology. 2023;14:1277928.

155. Björnsdóttir ES, Martins ER, Erlendsdottir H, Haraldsson G, Melo-Cristino J, Ramirez M, et al. Group B streptococcal neonatal and early infancy infections in Iceland, 1976–2015. The Pediatric Infectious Disease Journal. 2019;38(6):620-4.

156. Baldan R, Droz S, Casanova C, Knabben L, Huang DJ, Brülisauer C, et al. Group B streptococcal colonization in elderly women. BMC infectious diseases. 2021;21:1-8.

157. Warrier LM, Joy S, Bashir RA. Group B Streptococcal colonization among pregnant women and neonates in a tertiary care hospital in South India. Indian Journal of Pediatrics. 2022;89(12):1187-94.

158. Wataradee S, Samngamnim S, Boonserm T, Ajariyakhajorn K. Genotypic and antimicrobial susceptibility of Streptococcus agalactiae causing bovine mastitis in the central region of Thailand. Frontiers in Veterinary Science. 2023;10:1250436.

159. Kang HM, Lee HJ, Lee H, Jo DS, Lee HS, Kim TS, et al. Genotype characterization of group B Streptococcus isolated from infants with invasive diseases in South Korea. The Pediatric Infectious Disease Journal. 2017;36(10):e242-e7.

160. Doumith M, Mushtaq S, Martin V, Chaudhry A, Adkin R, Coelho J, et al. Genomic sequences of Streptococcus agalactiae with high-level gentamicin resistance, collected in the BSAC bacteraemia surveillance. Journal of Antimicrobial Chemotherapy. 2017;72(10):2704-7.

161. Williams AN, Croxen MA, Demczuk WH, Martin I, Tyrrell GJ. Genomic characterization of emerging invasive Streptococcus agalactiae serotype VIII in Alberta, Canada. European Journal of Clinical Microbiology & Infectious Diseases. 2023;42(6):747-57.

162. Perme T, Golparian D, Bombek Ihan M, Rojnik A, Lučovnik M, Kornhauser Cerar L, et al. Genomic and phenotypic characterisation of invasive neonatal and colonising group B Streptococcus isolates from Slovenia, 2001–2018. BMC Infectious Diseases. 2020;20:1-9.

163. Khan UB, Portal EA, Sands K, Lo S, Chalker VJ, Jauneikaite E, et al. Genomic analysis reveals new integrative conjugal elements and transposons in GBS conferring antimicrobial resistance. Antibiotics. 2023;12(3):544.

164. Campisi E, Rosini R, Ji W, Guidotti S, Rojas-Lopez M, Geng G, et al. Genomic analysis reveals multi-drug resistance clusters in group B Streptococcus CC17 hypervirulent isolates causing neonatal invasive disease in southern mainland China. Frontiers in microbiology. 2016;7:1265.

165. Palacios-Saucedo GdC, Rivera-Morales LG, Vázquez-Guillén JM, Caballero-Trejo A, Mellado-García MC, Flores-Flores AS, et al. Genomic analysis of virulence factors and antimicrobial resistance of group B Streptococcus isolated from pregnant women in northeastern Mexico. PloS one. 2022;17(3):e0264273.

166. Zhou Y, Wang L-Q, Yan Q, Lee C-C, Hsu M-H, Liao W-T, et al. Genomic analysis of group B Streptococcus from neonatal sepsis reveals clonal CC17 expansion and virulence-and resistance-associated traits after intrapartum antibiotic prophylaxis. Clinical Infectious Diseases. 2022;75(12):2153-60.

167. Gherardi G, Imperi M, Palmieri C, Magi G, Facinelli B, Baldassarri L, et al. Genetic diversity and virulence properties of Streptococcus dysgalactiae subsp. equisimilis from different sources. Journal of medical microbiology. 2014;63(1):90-8.

168. Feuerschuette OHM, Alves EV, Scheffer MC, Vilela APP, Barazzetti FH, Feuerschuette HM, et al. Genetic diversity and antimicrobial resistance of invasive, noninvasive and colonizing group B Streptococcus isolates in southern Brazil. Access Microbiology. 2022;4(6):000370.

169. Saffar HR, A. Abdollahi, A. Habibi, S. Baseri, Z. Frequency of inducible clindamycin resistance among gram-positive cocci in a tertiary hospital, Tehran, Iran. Iranian Journal of Microbiology. 2016;8(4):243-8.

170. Hadavand SG, F. Rajabi, L. Davati, A. Zafarghandi, N. Frequency of Group B Streptococcal Colonization in Pregnant Women Aged 35- 37 Weeks in Clinical Centers of Shahed University, Tehran, Iran. Iran J Pathol. 2015;10(2):120-6.

171. Carvalhaes CG, Sader HS, Streit JM, Mendes RE. Five-year analysis of the in vitro activity of tedizolid against a worldwide collection of indicated species causing clinical infections: results from the Surveillance of Tedizolid Activity and Resistance (STAR) programme. JAC-Antimicrobial Resistance. 2022;4(5):dlac088.

172. Ali MM, Woldeamanuel Y, Asrat D, Fenta DA, Beall B, Schrag S, et al. Features of Streptococcus agalactiae strains recovered from pregnant women and newborns attending different hospitals in Ethiopia. BMC infectious diseases. 2020;20:1-9.

173. Ábrók M, Tigyi P, Kostrzewa M, Burián K, Deák J. Evaluation of the results of Group B Streptococcus screening by MALDI-TOF MS among pregnant women in a Hungarian Hospital. Pathogens. 2019;9(1):1.

174. Ikebe T, Chiba K, Shima T, Masuda C, Okuno R, Ohya H, et al. Evaluation of streptococcal toxic shock-like syndrome caused by group B streptococcus in adults in Japan between 2009 and 2013. Journal of Infection and Chemotherapy. 2015;21(3):207-11.

175. Shen H, Zhu C, Liu X, Ma D, Song C, Zhou L, et al. The etiology of acute meningitis and encephalitis syndromes in a sentinel pediatric hospital, Shenzhen, China. BMC infectious diseases. 2019;19:1-9.

176. Jones S, Newton P, Payne M, Furfaro L. Epidemiology, antimicrobial resistance, and virulence determinants of group b Streptococcus in an Australian setting. Frontiers in Microbiology. 2022;13:839079.

177. Guan X, Mu X, Ji W, Yuan C, He P, Zhang L, et al. Epidemiology of invasive group B streptococcal disease in infants from urban area of South China, 2011–2014. BMC Infectious Diseases. 2018;18:1-8.

178. Alhhazmi A, Hurteau D, Tyrrell GJ. Epidemiology of invasive group B streptococcal disease in Alberta, Canada, from 2003 to 2013. Journal of clinical microbiology. 2016;54(7):1774-81.

179. Zhang X, Li Y, Tao Y, Ding Y, Shao X, Li W. Epidemiology and drug resistance of neonatal bloodstream infection pathogens in East China children’s Medical Center from 2016 to 2020. Frontiers in Microbiology. 2022;13:820577.

180. Florindo C, Damiao V, Silvestre I, Farinha C, Rodrigues F, Nogueira F, et al. Epidemiological surveillance of colonising group B Streptococcus epidemiology in the Lisbon and Tagus Valley regions, Portugal (2005 to 2012): emergence of a new epidemic type IV/clonal complex 17 clone. Eurosurveillance. 2014;19(23):20825.

181. Ma A, Thompson LA, Corsiatto T, Hurteau D, Tyrrell GJ. Epidemiological characterization of group B Streptococcus infections in Alberta, Canada: an update from 2014 to 2020. Microbiology Spectrum. 2021;9(3):e01283-21.

182. Crespo-Ortiz MdP, Castañeda-Ramirez CR, Recalde-Bolaños M, Vélez-Londoño JD. Emerging trends in invasive and noninvasive isolates of Streptococcus agalactiae in a Latin American hospital: a 17-year study. BMC infectious diseases. 2014;14:1-11.

183. Kao Y, Tsai M-H, Lai M-Y, Chu S-M, Huang H-R, Chiang M-C, et al. Emerging serotype III sequence type 17 group B streptococcus invasive infection in infants: the clinical characteristics and impacts on outcomes. BMC Infectious Diseases. 2019;19:1-8.

184. Hayes K, Cotter L, Barry L, O'HALLORAN F. Emergence of the L phenotype in Group B Streptococci in the South of Ireland. Epidemiology & Infection. 2017;145(16):3535-42.

185. Teatero S, Athey TB, Van Caeseele P, Horsman G, Alexander DC, Melano RG, et al. Emergence of serotype IV group B Streptococcus adult invasive disease in Manitoba and Saskatchewan, Canada, is driven by clonal sequence type 459 strains. Journal of clinical microbiology. 2015;53(9):2919-26.

186. Tulyaprawat O, Pharkjaksu S, Shrestha RK, Ngamskulrungroj P. Emergence of multi-drug resistance and its association with uncommon serotypes of Streptococcus agalactiae isolated from non-neonatal patients in Thailand. Frontiers in microbiology. 2021;12:719353.

187. Zhang L, Kang W-J, Zhu L, Xu L-J, Guo C, Zhang X-H, et al. Emergence of invasive serotype Ib sequence type 10 group B streptococcus disease in Chinese infants is driven by a tetracycline-sensitive clone. Frontiers in Cellular and Infection Microbiology. 2021;11:642455.

188. Guo Y, Deng X, Liang Y, Zhang L, Zhao G-P, Zhou Y. The draft genomes and investigation of serotype distribution, antimicrobial resistance of group B Streptococcus strains isolated from urine in Suzhou, China. Annals of clinical microbiology and antimicrobials. 2018;17:1-7.

189. Nabavinia MK, M. B. Sadeh, M. Eslami, G. Vakili, M. Azartoos, N. Mojibiyan, M. Distribution of pilus island and antibiotic resistance genes in streptococcus agalactiae obtained from vagina of pregnant women in Yazd, Iran. Iranian Journal of Microbiology. 2020;12(5):411-6.

190. Li X, Du Z, Tang Z, Wen Q, Cheng Q, Cui Y. Distribution and drug sensitivity of pathogenic bacteria in diabetic foot ulcer patients with necrotizing fasciitis at a diabetic foot center in China. BMC Infectious Diseases. 2022;22(1):396.

191. Fahim NAE, Ragay MB, Salah El-Deen NN, ElMasry SA. Diagnostic performance of two chromogenic media for Streptococcus agalactiae screening in pregnant women. Microbes and Infectious Diseases. 2022;3(4):947-55.

192. El Shahaway AA, El Maghraby HM, Mohammed HA, Abd Elhady RR, Abdelrhman AA. Diagnostic performance of direct latex agglutination, post-enrichment latex agglutination and culture methods in screening of group B streptococci in late pregnancy: a comparative study. Infection and drug resistance. 2019:2583-8.

193. Jalalifar S, Havaei SA, Motallebirad T, Moghim S, Fazeli H, Esfahani BN. Determination of surface proteins profile, capsular genotyping, and antibiotic susceptibility patterns of Group B Streptococcus isolated from urinary tract infection of Iranian patients. BMC research notes. 2019;12:1-6.

194. Panahi S, Khalili MB, Sadeh M, Vakili M. Determination of pilus-islands profile and antibiotic susceptibility of Streptococcus agalactiae isolated from urine of pregnant women. Iranian Journal of Microbiology. 2023;15(2):219.

195. Motallebirad T, Fazeli H, Azadi D, Shokri D, Moghim S, Esfahani BN. Determination of capsular serotypes, antibiotic susceptibility pattern, and molecular mechanism of erythromycin resistance among clinical isolates of Group B Streptococcus in Isfahan, Iran. Advanced Biomedical Research. 2021;10(1):27.

196. Burcham LR, Spencer BL, Keeler LR, Runft DL, Patras KA, Neely MN, et al. Determinants of Group B streptococcal virulence potential amongst vaginal clinical isolates from pregnant women. PLoS One. 2019;14(12):e0226699.

197. Evangelia KE, S. Iliana, T. Evi, C. Ioanna, T. Vassiliki, G. Stavroula, B. Decreased susceptibility to clindamycin, erythromycin and tetracycline of streptococcus agalactiae isolated from vaginal samples. Acta Microbiologica Hellenica. 2015;60(3):180.

198. Malița MA, Manolescu LSC, Pîrvu CF, Costea RC, Marcov EC, Burlibasa M, et al. Cumulative Antibiogram: A Rapid Method to Hinder Transmission of Resistant Bacteria to Oral Cavity of Newborn Babies. Antibiotics. 2023;12(1):80.

199. Goudarzi G, Ghafarzadeh M, Shakib P, Anbari K. Culture and real-time PCR based maternal screening and antibiotic susceptibility for group B Streptococcus: an Iranian experience. Global journal of health science. 2015;7(6):233.

200. Asghar S, Khan JA, Mahmood MS, Arshad MI. A Cross-sectional study of group B streptococcus–associated sepsis, coinfections, and antibiotic susceptibility profile in neonates in Pakistan. Advances in Neonatal Care. 2020;20(4):E59-E69.

201. Felemban EM, Al Juaid DA, Alsanie WF, Hassan MM, Gaber A. Correlation Between Strain Distribution and Antibiotic Resistance Genes Pattern of Streptococcus agalactiae Group B from Patients in Taif, Saudi Arabia. Journal of Pure & Applied Microbiology. 2019;13(1).

202. Brigtsen AK, Dedi L, Melby KK, Holberg-Petersen M, Radtke A, Lyng RV, et al. Comparison of PCR and serotyping of Group B Streptococcus in pregnant women: the Oslo GBS-study. Journal of microbiological methods. 2015;108:31-5.

203. Mulu W, Yimer M, Zenebe Y, Abera B. Common causes of vaginal infections and antibiotic susceptibility of aerobic bacterial isolates in women of reproductive age attending at Felegehiwot referral Hospital, Ethiopia: a cross sectional study. BMC women's health. 2015;15:1-9.

204. Ji W, Zhang L, Guo Z, Xie S, Yang W, Chen J, et al. Colonization prevalence and antibiotic susceptibility of Group B Streptococcus in pregnant women over a 6-year period in Dongguan, China. PloS one. 2017;12(8):e0183083.

205. Akpaka PE, Henry K, Thompson R, Unakal C. Colonization of Streptococcus agalactiae among pregnant patients in Trinidad and Tobago. IJID regions. 2022;3:96-100.

206. Sapugahawatte DN, Li C, Liyanapathirana V, Kandauda C, Gihan C, Zhu C, et al. Colonization of group B Streptococcus in pregnant women and their neonates from a Sri Lankan Hospital. Pathogens. 2022;11(4):386.

207. Hsu J-F, Chen Y-N, Chu S-M, Lee W-J, Huang H-R, Chiang M-C, et al. Clonal complex 12 serotype Ib Streptococcus agalactiae strain causing complicated sepsis in neonates: Clinical features and genetic characteristics. Microbiology Spectrum. 2023;11(1):e03778-22.

208. Yoon IA, Jo DS, Cho EY, Choi EH, Lee HJ, Lee H. Clinical significance of serotype V among infants with invasive group B streptococcal infections in South Korea. International Journal of Infectious Diseases. 2015;38:136-40.

209. Jiao J, Wu W, Shen F, Liu Z, Zhou H, Fan G, et al. Clinical Profile and Risk Factors of Group B Streptococcal Colonization in Mothers from the Eastern District of China. Journal of Tropical Medicine. 2022;2022(1):5236430.

210. AlZuheiri STS, Dube R, Menezes G, Qasem S. Clinical profile and outcome of Group B streptococcal colonization in mothers and neonates in Ras Al Khaimah, United Arab Emirates: A prospective observational study. Saudi Journal of Medicine & Medical Sciences. 2021;9(3):235-40.

211. Li X, Ding X, Shi P, Zhu Y, Huang Y, Li Q, et al. Clinical features and antimicrobial susceptibility profiles of culture-proven neonatal sepsis in a tertiary children's hospital, 2013 to 2017. Medicine. 2019;98(12):e14686.

212. Hirai N, Kasahara K, Nakano R, Ogawa Y, Suzuki Y, Ogawa M, et al. Clinical characteristics and molecular epidemiology of invasive Streptococcus agalactiae infections between 2007 and 2016 in Nara, Japan. Plos one. 2020;15(10):e0240590.

213. Li C, Feng W-y, Lin A-w, Zheng G, Wang Y-c, Han Y-j, et al. Clinical characteristics and etiology of bacterial meningitis in Chinese children> 28 days of age, January 2014–December 2016: a multicenter retrospective study. International Journal of Infectious Diseases. 2018;74:47-53.

214. Guo L-y, Zhang Z-x, Wang X, Zhang P-p, Shi W, Yao K-h, et al. Clinical and pathogenic analysis of 507 children with bacterial meningitis in Beijing, 2010–2014. International Journal of Infectious Diseases. 2016;50:38-43.

215. Lee H, Kim ES, Song K-H, Kim HB, Park JS, Park KU. Clinical and molecular epidemiology of invasive group B Streptococcus infections in adults in a referral center in Korea. European Journal of Clinical Microbiology & Infectious Diseases. 2022;41(12):1407-13.

216. Mathur P, Bhardwaj N, Mathur K, Behera B, Gupta G, Kapil A, et al. Clinical and molecular epidemiology of beta-hemolytic streptococcal infections in India. The Journal of Infection in Developing Countries. 2014;8(03):297-303.

217. Kernéis S, Plainvert C, Barnier J-P, Tazi A, Dmytruk N, Gislain B, et al. Clinical and microbiological features associated with group B Streptococcus bone and joint infections, France 2004–2014. European Journal of Clinical Microbiology & Infectious Diseases. 2017;36:1679-84.

218. Wang Y-H, Chen H-M, Yang Y-H, Yang T-H, Teng C-H, Chen C-L, et al. Clinical and microbiological characteristics of recurrent group B streptococcal infection among non-pregnant adults. International Journal of Infectious Diseases. 2014;26:140-5.

219. Hsu J-F, Lu J-J, Chu S-M, Lee W-J, Huang H-R, Chiang M-C, et al. The Clinical and Genetic Characteristics of Streptococcus agalactiae Meningitis in Neonates. International Journal of Molecular Sciences. 2023;24(20):15387.

220. Zeng S, Tang X, Zhao W, Qiu H, Wang H, Feng Z. Clinical analysis of cases of neonatal Streptococcus agalactiae sepsis. Genet Mol Res. 2016;15(2).

221. Emaneini M, Jabalameli F, Mirsalehian A, Ghasemi A, Beigverdi R. Characterization of virulence factors, antimicrobial resistance pattern and clonal complexes of group B streptococci isolated from neonates. Microbial pathogenesis. 2016;99:119-22.

222. Tang Y, Yu F, Hu Z, Peng L, Jiang Y. Characterization of aerobic vaginitis in late pregnancy in a Chinese population: A STROBE-compliant study. Medicine. 2020;99(25):e20732.

223. Suhaimi ME, Desa MN, Eskandarian N, Pillay SG, Ismail Z, Neela VK, et al. Characterization of a group B Streptococcus infection based on the demographics, serotypes, antimicrobial susceptibility and genotypes of selected isolates from sterile and non-sterile isolation sites in three major hospitals in Malaysia. Journal of infection and public health. 2017;10(1):14-21.

224. Piccinelli G, Biscaro V, Gargiulo F, Caruso A, De Francesco MA. Characterization and antibiotic susceptibility of Streptococcus agalactiae isolates causing urinary tract infections. Infection, Genetics and Evolution. 2015;34:1-6.

225. Al-Matary A, Heena H, AlSarheed AS, Ouda W, AlShahrani DA, Wani TA, et al. Characteristics of neonatal Sepsis at a tertiary care hospital in Saudi Arabia. Journal of infection and public health. 2019;12(5):666-72.

226. Chang B, Wada A, Hosoya M, Oishi T, Ishiwada N, Oda M, et al. Characteristics of group B Streptococcus isolated from infants with invasive infections: a population-based study in Japan. Japanese Journal of Infectious Diseases. 2014;67(5):356-60.

227. Hays C, Louis M, Plainvert C, Dmytruk N, Touak G, Trieu-Cuot P, et al. Changing epidemiology of group B Streptococcus susceptibility to fluoroquinolones and aminoglycosides in France. Antimicrobial agents and chemotherapy. 2016;60(12):7424-30.

228. Flamm RK, Sader HS, Jones RN. Ceftaroline activity tested against contemporary Latin American bacterial pathogens (2011). The Brazilian Journal of Infectious Diseases. 2014;18(2):187-95.

229. Goudarzi M, Navidinia M. Overview perspective of bacterial strategies of resistance to biocides and antibiotics. Archives of Clinical Infectious Diseases. 2019;14(2).

230. Swann O, Everett DB, Furyk JS, Harrison EM, Msukwa MT, Heyderman RS, et al. Bacterial meningitis in Malawian infants< 2 months of age: etiology and susceptibility to World Health Organization first-line antibiotics. The Pediatric infectious disease journal. 2014;33(6):560-5.

231. Zhou Y, Zhou S, Peng J, Min L, Chen Q, Ke J. Bacterial distribution and drug resistance in blood samples of children in Jiangxi Region, 2017–2021. Frontiers in Cellular and Infection Microbiology. 2023;13:1163312.

232. Majigo M, Makupa J, Mwazyunga Z, Luoga A, Kisinga J, Mwamkoa B, et al. Bacterial aetiology of neonatal sepsis and antimicrobial resistance pattern at the regional referral Hospital, Dar es Salam, Tanzania; a call to strengthening antibiotic stewardship program. Antibiotics. 2023;12(4):767.

233. Ahmad S. Asymptomatic group B streptococcal bacteriuria among pregnant women in Saudi Arabia. British journal of biomedical science. 2015;72(3):135-9.

234. Mwei MK, Mchome B, John B, Maro E. Asymptomatic bacteriuria among pregnant women attending antenatal care at Kilimanjaro Christian Medical Centre in Northern Tanzania. Tanzania Journal of Health Research. 2018;20(4).

235. Foster-Nyarko E, Kwambana B, Aderonke O, Ceesay F, Jarju S, Bojang A, et al. Associations between nasopharyngeal carriage of Group B Streptococcus and other respiratory pathogens during early infancy. BMC microbiology. 2016;16:1-9.

236. Morozumi M, Wajima T, Kuwata Y, Chiba N, Sunaoshi K, Sugita K, et al. Associations between capsular serotype, multilocus sequence type, and macrolide resistance in Streptococcus agalactiae isolates from Japanese infants with invasive infections. Epidemiology & Infection. 2014;142(4):812-9.

237. Vigliarolo L, Arias B, Suárez M, Van Haute E, Kovacec V, Lopardo H, et al. Argentinian multicenter study on urinary tract infections due to Streptococcus agalactiae in adult patients. J Infect Dev Ctries. 2019;13(1):77-82.

238. Morfin-Otero R, Noriega ER, Dowzicky MJ. Antimicrobial susceptibility trends among gram-positive and-negative clinical isolates collected between 2005 and 2012 in Mexico: results from the Tigecycline Evaluation and Surveillance Trial. Annals of Clinical Microbiology and Antimicrobials. 2015;14:1-14.

239. Eskandarian N, Ismail Z, Neela V, Van Belkum A, Desa M, Amin Nordin S. Antimicrobial susceptibility profiles, serotype distribution and virulence determinants among invasive, non-invasive and colonizing Streptococcus agalactiae (group B streptococcus) from Malaysian patients. European Journal of Clinical Microbiology & Infectious Diseases. 2015;34:579-84.

240. Lagunas-Rangel FA. Antimicrobial susceptibility profiles of bacteria causing urinary tract infections in Mexico: Single-centre experience with 10 years of results. Journal of global antimicrobial resistance. 2018;14:90-4.

241. Mubanga P, Steinberg WJ, Van Rooyen FC. Antimicrobial susceptibility profile of uropathogens in Maluti Adventist Hospital patients, 2011. African journal of primary health care & family medicine. 2015;7(1):1-5.

242. Bhola P, Mvelase N, Balakrishna Y, Mlisana K, Swe-Han KS. Antimicrobial susceptibility patterns of uropathogens isolated from pregnant women in KwaZulu-Natal Province: 2011-2016. South African Medical Journal. 2020;110(9):872-6.

243. MELO SCCSd, SANTOS NCdS, OLIVEIRA Md, SCODRO RBdL, Cardoso RF, PÁDUA RAF, et al. Antimicrobial susceptibility of Streptococcus agalactiae isolated from pregnant women. Revista do Instituto de Medicina Tropical de São Paulo. 2016;58:83.

244. Balkhi B, Mansy W, Alghadeer S, Alnuaim A, Alshehri A, Somily A. Antimicrobial susceptibility of microorganisms causing urinary tract infections in Saudi Arabia. The Journal of Infection in Developing Countries. 2018;12(04):220-7.

245. Said M, Dangor Y, Mbelle N, Madhi SA, Kwatra G, Ismail F. Antimicrobial susceptibility and serotype distribution of Streptococcus agalactiae recto-vaginal colonizing isolates from pregnant women at a tertiary hospital in Pretoria, South Africa: an observational descriptive study. BioRxiv. 2019:564856.

246. Mohamed AA. Antimicrobial Resistance Rates in Gram-positive Uropathogens in Duhok city, Kurdistan Region of Iraq. medRxiv. 2023:2023.02. 26.23286459.

247. Tesfaye A, Melese A, Derbie A. Antimicrobial resistance profile and associated factors of group B Streptococci colonization among pregnant women attending antenatal clinics in Jigjiga, Southeast Ethiopia. International Journal of Microbiology. 2022;2022(1):9910842.

248. Minotti C, Di Caprio A, Facchini L, Bedetti L, Miselli F, Rossi C, et al. Antimicrobial Resistance Pattern and Empirical Antibiotic Treatments in Neonatal Sepsis: A Retrospective, Single-Center, 12-Year Study. Antibiotics. 2023;12(10):1488.

249. Van Du V, Dung PT, Toan NL, Van Mao C, Bac NT, Van Tong H, et al. Antimicrobial resistance in colonizing group B Streptococcus among pregnant women from a hospital in Vietnam. Scientific Reports. 2021;11(1):20845.

250. Guo H, Fu M, Peng Q, Chen Z, Liu J, Qiu Y, et al. Antimicrobial resistance and molecular characterization of Streptococcus agalactiae from pregnant women in southern China. The Journal of Infection in Developing Countries. 2019;13(09):802-9.

251. Dashtizade M, Zolfaghari MR, Yousefi M, Nazari-Alam A. Antibiotic susceptibility patterns and prevalence of streptococcus agalactiae rectovaginal colonization among pregnant women in Iran. Revista Brasileira de Ginecologia e Obstetrícia. 2020;42(08):454-9.

252. Dilrukshi N, Kottahachchi J, Dissanayake T, Fernando N. Antibiotic Sensitivity of Group B Streptococcus from Pregnant Mothers and Its Association with Resistance Genes. Medical Principles and Practice. 2023;32(2):126-32.

253. Bolukaoto JY, Monyama CM, Chukwu MO, Lekala SM, Nchabeleng M, Maloba MR, et al. Antibiotic resistance of Streptococcus agalactiae isolated from pregnant women in Garankuwa, South Africa. BMC research notes. 2015;8:1-7.

254. Tan J, Wang Y, Gong X, Li J, Zhong W, Shan L, et al. Antibiotic resistance in neonates in China 2012–2019: a multicenter study. Journal of Microbiology, Immunology and Infection. 2022;55(3):454-62.

255. Zakerifar M, Kaboosi H, Goli HR, Rahmani Z, Peyravii Ghadikolaii F. Antibiotic resistance genes and molecular typing of Streptococcus agalactiae isolated from pregnant women. BMC Pregnancy and Childbirth. 2023;23(1):43.

256. Cheng Z, Qu P, Ke P, Yang X, Zhou Q, Lan K, et al. Antibiotic resistance and molecular epidemiological characteristics of Streptococcus agalactiae isolated from pregnant women in Guangzhou, South China. Canadian Journal of Infectious Diseases and Medical Microbiology. 2020;2020(1):1368942.

257. Mudzana R, Mavenyengwa RT, Gudza-Mugabe M. Analysis of virulence factors and antibiotic resistance genes in group B streptococcus from clinical samples. BMC infectious diseases. 2021;21:1-11.

258. Yu YYH, Q. K. Liu, A. C. Analysis of pathogens, drug resistance, sensitive antibiotic treatment and risk factors of early-onset sepsis in very low birth weight infants. American Journal of Translational Research. 2021;13(11):12939-48.

259. Zhang X, Tan L, Ouyang P, Ma H, Peng J, Shi T, et al. Analysis of distribution and antibiotic resistance of Gram-positive bacteria isolated from a tertiary-care hospital in southern China: an 8-year retrospective study. Frontiers in Microbiology. 2023;14:1220363.

260. Fujiya Y, Hayakawa K, Gu Y, Yamamoto K, Mawatari M, Kutsuna S, et al. Age-related differences in clinical characteristics of invasive group G streptococcal infection: Comparison with group A and group B streptococcal infections. PLoS One. 2019;14(3):e0211786.

261. Perim MC, Borges JdC, Celeste SRC, Orsolin EdF, Mendes RR, Mendes GO, et al. Aerobic bacterial profile and antibiotic resistance in patients with diabetic foot infections. Revista da Sociedade Brasileira de Medicina Tropical. 2015;48(5):546-54.

262. Karim GF, AL-Salihi SS, Atya QM, Abass KS. Aerobic and anaerobic bacteria in tonsils of different ages with recurrent tonsillitis. Indian J Public Health Res Dev. 2019;10(9):132-6.

263. Mendes RE, Farrell DJ, Sader HS, Flamm RK, Jones RN. Activity of oritavancin against Gram-positive clinical isolates responsible for documented skin and soft-tissue infections in European and US hospitals (2010–13). Journal of Antimicrobial Chemotherapy. 2015;70(2):498-504.

264. Mukesi M, Iweriebor BC, Obi LC, Nwodo UU, Moyo SR, Okoh AI. The activity of commercial antimicrobials, and essential oils and ethanolic extracts of Olea europaea on Streptococcus agalactiae isolated from pregnant women. BMC complementary and alternative medicine. 2019;19:1-9.

265. Pfaller MA, Flamm RK, Jones RN, Farrell DJ, Mendes RE. Activities of tedizolid and linezolid determined by the reference broth microdilution method against 3,032 Gram-positive bacterial isolates collected in Asia-Pacific, Eastern Europe, and Latin American countries in 2014. Antimicrobial Agents and Chemotherapy. 2016;60(9):5393-9.

266. Ko MH-J, Chang H-Y, Li S-T, Jim W-T, Chi H, Hsu C-H, et al. An 18-year retrospective study on the epidemiology of early-onset neonatal sepsis-emergence of uncommon pathogens. Pediatrics & Neonatology. 2021;62(5):491-8.
